# Safety, pharmacokinetics, and neutralisation activity of PGDM1400LS, VRC07-523LS, and PGT121.414.LS infused intravenously or subcutaneously (HVTN 140/HPTN 101 part B): a phase 1, randomised trial

**DOI:** 10.1016/S2352-3018(25)00356-X

**Published:** 2026-05-11

**Authors:** Sharana Mahomed, Carmen A Paez, Yunda Huang, Kelly E Seaton, Chenchen Yu, Kevin Gillespie, Maurine D Miner, Shelly T Karuna, Theresa Gamble, Jack Heptinstall, Elize Domin, Haili Tang, Lu Zhang, Fei Gao, Margaret Yacovone, Hans M L Spiegel, Julie Dumond, Maija A Anderson, Estelle Piwowar-Manning, Bonnie J Dye, India Tindale, Lori Proulx-Burns, Meg Trahey, Simbarashe Takuva, Azwidihwi Takalani, Veronique C Bailey, Spyros A Kalams, Hyman Scott, Josphat Kosgei, Sinéad Delany-Moretlwe, Sheetal Kassim, Fatima Laher, Z Mike Chirenje, Yeukai Musodza, Felix Mhlanga, Nonhlanhla Mkhize, Joshua A Weiner, Margaret E Ackerman, M Juliana McElrath, Michael N Pensiero, Lucio Gama, Dan H Barouch, Lawrence Corey, Myron S Cohen, David C Montefiori, Georgia D Tomaras, Marc Siegel, Colleen F Kelley

**Affiliations:** **Centre for the AIDS Programme of Research in South Africa, Doris Duke Medical Research Institute, University of KwaZulu Natal, Durban, South Africa** (S Mahomed MD PhD); **Department of Surgery** (K E Seaton PhD, J Heptinstall BS, E Domin BS, H Tang MS, L Zhang MS, Prof D C Montefiori PhD, Prof G D Tomaras PhD), **Department of Integrative Immunobiology** (Prof G D Tomaras), **Department of Molecular Genetics and Microbiology** (Prof G D Tomaras), **Duke Center for Human Systems Immunology, Durham, NC, USA; Vaccine and Infectious Disease Division, Fred Hutchinson Cancer Center, Seattle, WA, USA** (C A Paez MD, Y Huang PhD, C Yu MS, K Gillespie MS, M D Miner PhD, S T Karuna MD, F Gao PhD, M A Anderson MN, I Tindale MSc, L Proulx-Burns MN, M Trahey PhD, S Takuva MBChB, Prof M J McElrath MD, Prof L Corey MD); **FHI 360, Durham, NC, USA** (T Gamble PhD, B J Dye MPH); **Department of Medical Microbiology, School of Laboratory Medicine & Medical Science, University of KwaZulu-Natal, Durban, South Africa** (M D Miner); **National Institute of Allergy and Infectious Diseases, Rockville, MD, USA** (M Yacovone MD, M N Pensiero PhD); **Kelly Government Solutions, Rockville, MD, USA** (H M L Spiegel MD); **Eshelman School of Pharmacy, University of North Carolina at Chapel Hill, Chapel Hill, NC, USA** (J Dumond PharmD, Prof M S Cohen MD); **Department of Pathology, School of Medicine, Johns Hopkins University, Baltimore, MD, USA** (E Piwowar-Manning BS); **Hutchinson Centre Research Institute of South Africa, Chris Hani Baragwanath Academic Hospital, Johannesburg, South Africa** (A Takalani FCPHM, V C Bailey MBChB); **Department of Medicine,** (Prof S A Kalams MD); **Department of Pathology, Microbiology, and Immunology** (Prof S A Kalams MD), **Division of Infectious Diseases, Vanderbilt University Medical Center, Nashville, TN, USA); San Francisco Department of Public Health, San Francisco, CA, USA** (Prof H Scott MD); **Kenya Medical Research Institute–Walter Reed Project, Kericho, Kenya** (J Kosgei MBChB); **Wits Reproductive Health and HIV Institute, University of the Witwatersrand, Johannesburg, South Africa** (Prof S Delany-Moretlwe MD); **Desmond Tutu HIV Centre, University of Cape Town, Cape Town, South Africa** (S Kassim MBChB); **Perinatal HIV Research Unit, Faculty of Health Sciences, University of the Witwatersrand, Johannesburg, South Africa** (F Laher MBBCh); **Institute of Global Health, University of California San Francisco, San Francisco, CA, USA** (Prof Z M Chirenje MD); **University of Zimbabwe Clinical Trials Research Center, Harare, Zimbabwe** (Y Musodza MSc, F Mhlanga MBChB); **National Institute for Communicable Diseases, Durban, South Africa** (N Mkhize PhD); **Thayer School of Engineering, Dartmouth College, Hanover, NH, USA** (J A Weiner PhD, Prof M E Ackerman PhD); **Vaccine Research Centre, National Institute of Allergy and Infectious Diseases, Bethesda, MD, USA** (L Gama PhD); **Center for Virology and Vaccine Research, Beth Israel Deaconess Medical Center, Harvard Medical School, Boston, MA, USA** (Prof D H Barouch MD); **George Washington University School of Medicine and Health Sciences, Washington, DC, USA** (Prof M Siegel MD); **Division of Infectious Diseases, Department of Medicine, Emory University School of Medicine, Atlanta, GA, USA** (Prof C F Kelley MD)

## Abstract

**Background:**

Passive immunisation with a combination of broadly neutralising monoclonal antibodies (mAbs) presents a potential HIV-1 prevention modality. This study (HVTN 140/HPTN 101) sought to evaluate PGDM1400LS (targeting V3-glycan) administered alone (part A, previously reported), and in combination (part B) with VRC07-523LS (targeting CD4 binding site) and PGT121.414.LS (targeting V2-apex) in healthy adults without HIV-1.

**Methods:**

The study was a phase 1, multicentre, randomised, open-label trial done across the USA (five sites), Kenya (one site), South Africa (four sites), and Zimbabwe (three sites). Part B participants were randomly assigned to five groups (n=16 each), all receiving the three mAbs at months 0 and 4: infusion group T6 (20 mg/kg each mAb intravenously), T7 (20 mg/kg each mAb subcutaneously), T8 (1·4 g each mAb intravenously), T9 (1·4 g each mAb subcutaneously), and T10 (40 mg/kg each mAb intravenously). Primary endpoints were safety and tolerability throughout the study, and pharmacokinetics and neutralising activity on days 0, 3, 6, 28, 56, 112, 168, 224, and 280. Serum mAb concentrations over time were assessed via a validated anti-idiotype binding assay and analysed with two-compartment population pharmacokinetics models. Serum neutralisation titres were assessed by a validated TZM-bl assay. The study is registered at ClinicalTrials.gov (NCT05184452) and is complete.

**Findings:**

For the 80 part B participants enrolled from March 21 to Oct 5, 2022, the median age was 27·0 years, with 47·5% females. Most participants had mild-to-moderate solicited local and systemic symptoms. No serious adverse events were reported. On the basis of pharmacokinetics data from part A and part B combined, the estimated elimination half-life of PGDM1400LS was 53 days (51·4–55·3). Subcutaneous administration of PGDM1400LS exhibited bioavailability of 73·0% (67·5–78·5%) relative to intravenous administration of the same dose. PGT121.414. LS had an estimated elimination half-life of 65 days (61·8–68·1) with subcutaneous bioavailability of 77·7% (71·2–84·1), and VRC07–523LS had an estimated elimination half-life of 44 days (41·6–45·7) with a subcutaneous bioavailability of 80·1% (71·4–88·8). Both weight-based and fixed-dose regimens showed similar pharmacokinetic profiles. Observed neutralisation titres were consistent with those predicted on the basis of concentrations and in vitro neutralisation. No treatment-induced anti-drug antibody responses were detected.

**Interpretation:**

The mAb combination of PGDM1400LS, PGT121.414.LS, and VRC07-523LS was safe and well tolerated, with no antagonistic pharmacokinetic interactions or loss of neutralisation activity. These findings support further evaluation of this combination in future efficacy trials.

## Introduction

The global HIV epidemic remains a major public health challenge, with around 1·3 million new infections in 2023 according to UNAIDS and WHO. Advances in prevention science continue to contribute to the decline in HIV incidence, but available modalities are insufficient to end the HIV epidemic. There remains a product gap in the market and passive antibodies might help address this gap.^1^

Broadly neutralising monoclonal antibodies (mAbs) are a potential approach for HIV prevention. These antibodies, capable of neutralising a wide range of HIV-1 strains across multiple clades, might be effective for prevention of HIV infection when administered passively.^2^ The Antibody Mediated Prevention (AMP) trials showed that although the single mAb VRC01 alone did not achieve overall efficacy in preventing HIV acquisition, it showed 75% efficacy against strains with high neutralisation sensitivity to VRC01.^3^ This proof-of-concept study emphasised the likely need for combining multiple, highly potent, and broadly neutralising antibodies targeting different HIV-1 epitopes to effectively prevent viral resistance and achieve comprehensive neutralisation coverage.

Advancements in antibody engineering have led to the development of longer lasting mAbs. The LS modification (M428L/N434S substitutions) in the fragment crystalisable (Fc) region of mAbs is designed to extend mAb half-life in vivo by enhancing binding to the neonatal Fc receptor.^4^ Among these LS version mAbs are: PGDM1400LS, which targets the V2 apex of the HIV-1 envelope; VRC07-523LS, which targets the CD4 binding site; and PGT121.414.LS, which targets the V3-glycan supersite. In-vitro models indicate that administration of combination mAbs results in more potent and broader neutralisation than use of any single mAb alone. In clinical testing, combinations of mAbs including VRC07-523LS and PGT121.414.LS have shown safety and favourable pharmacokinetic profiles.^5–7^ PGDM1400 has also shown an excellent safety profile in humans when administered as a single agent, in a dual combination with PGT121, and in a triple cocktail with PGT121 plus VRC07-523LS.^8^

HIV Vaccine Trials Network (HVTN) 140/HIV Prevention Trials Network (HPTN) 101 assessed the safety, tolerability, pharmacokinetics, and neutralisation activity of PGDM1400LS when administered alone (part A) and in combination with VRC07-523LS and PGT121.414.LS (part B) in healthy adults. Part A of the trial was a first-in-human evaluation of PGDM1400LS, involving 15 participants who received dose-escalated intravenous or subcutaneous infusions. Part A data showed that PGDM1400LS was safe and well tolerated.^9^ Additionally, PGDM1400LS retained neutralisation breadth and potency in vivo, and the LS modification effectively increased its serum half-life by two to three times versus the parental PGDM1400.^9^

This Article presents findings from part B, which enrolled an additional 80 participants to evaluate first-inhuman combination regimens of PGDM1400LS, VRC07-523LS, and PGT121.414.LS. HVTN 140/HPTN 101 explored both intravenous and subcutaneous routes of administration, as well as weight-based and fixed-dose strategies, to address the potential for simplified dosing in real-world settings.

## Methods

### Study design and participants

This study was a multicentre, randomised, open-label trial done across the USA (five clinical research sites [CRSs]), Kenya (one CRS), South Africa (four CRSs), and Zimbabwe (three CRSs; appendix p 8), with approval obtained from the national regulatory authorities overseeing each country and from the institutional review boards and ethics committees overseeing each site. The protocol in its entirety can be found in the appendix (p 40). Partial eligibility criteria are listed in the appendix (p 8) with full criteria found in the protocol (appendix). All participants provided written consent.

In part B of this study, 80 healthy adults aged 18–50 years were randomly assigned into five groups of 16 participants each from March 21 to Oct 5, 2022. Participants received two rounds of a three-antibody combination infusion (PGDM1400LS, VRC07-523LS, and PGT121.414.LS) administered sequentially in this order, at months 0 and 4. Groups varied in dosage and administration route: T6 received 20 mg/kg of each antibody intravenously; T7 received 20 mg/kg of each antibody subcutaneously; T8 and T9 received a 1·4 g fixed dose of each antibody intravenously and subcutaneously, respectively; and T10 received 40 mg/kg intravenously of each antibody. The study is registered at ClinicalTrials.gov (NCT05184452).

### Randomisation and masking

In part B, T6 (intravenous 20 mg/kg), T7 (subcutaneous 20 mg/kg), T8 (intravenous 1·4 g fixed dose), and T9 (subcutaneous 1·4 g fixed dose) were randomly assigned and enrolled simultaneously after review of part A safety data. There was no randomisation for T10 (intravenous 40 mg/kg) and enrolment was opened after review of T6, T7, T8, and T9 safety data. Randomisation assignments were computer-generated and provided to the CRS pharmacist through a web-based randomisation system. Participants and CRS staff were unmasked to participant group assignments. Laboratory staff remained masked to group assignments within part B during sample analysis.

### Procedures

PGDM1400LS, VRC07-523LS, and PGT121.414.LS were administered sequentially in that order on the day of enrolment and again 4 months later. For intravenous infusions, each mAb was administered over approximately 30–60 min at the first infusion visit, and 15–30 min at the second visit. Subcutaneous infusions were administered at a rate of approximately 15 mL/h per infusion site at the first visit, potentially increasing to 20 mL/h at the second visit as tolerated. Subcutaneous administrations used two to three infusion sites for each mAb on the abdomen, arms, or thighs; no hyaluronidase was used to support administration. Participants and CRS study clinicians decided whether to administer the subcutaneous infusions at 1, 2, or 3 anatomical locations on the basis of participant preference and tolerability, and CRS study clinician assessment. Total infusion times ranged from approximately 1·5 h to 3 h for intravenous administrations and from 1 h to more than 3 h for subcutaneous administration depending on group assignment and number of infusion sites.

Participants were observed in the clinic for about 60 min after the last infusion at each visit. During this time, they completed an acceptability questionnaire and were monitored for solicited local and systemic adverse events. Participants tracked adverse events in a diary for 3 days following each infusion visit.

Safety was evaluated through solicited and unsolicited adverse events. Safety assessments, including physical examinations, vital signs, and laboratory tests, were done at scheduled visits throughout the 10-month study period. Solicited systemic symptoms were reported as related or not related. Unsolicited adverse events were documented for the entire study duration. Infusion-related reactions were defined as a constellation of signs and symptoms that affect multiple systems, which were deemed by the CRS investigators to be related and had a temporal relationship to study product administration. All adverse events were graded according to the Division of AIDS Table for Grading the Severity of Adult and Pediatric Adverse Events, corrected version 2.1, July, 2017, with specific exceptions (appendix p 9).

Safety laboratory assessments were done at prespecified timepoints throughout the study. The HVTN 140/HPTN 101 protocol safety review team and HVTN safety monitoring board continuously monitored safety reports throughout the study duration.

Serum samples were tested against mAb-specific viruses that are known to be sensitive to the specific mAb in vitro, and a diverse panel of 11 HIV-1 strains derived from AMP placebo recipients who acquired HIV-1 during the AMP study, selected as being sensitive to multiple epitope classes of mAbs in vitro (appendix pp 9–10).^9^ Neutralising antibodies against HIV-1 were evaluated by measuring reductions in Tat-regulated luciferase (Luc) reporter gene expression in TZM-bl cells as reported previously^10,11^ and in the appendix (pp 6–7).

The mAb-specific viruses used in this assay included: 6540.v4.c1, sensitive to neutralisation by PGDM1400LS, but resistant to PGT121.414.LS and VRC07-523LS; CNE55.N160K, sensitive to VRC07-523LS neutralisation, but resistant to PGT121.414.LS and PGDM1400LS; and CH505TF.N334S.N160A.N280D.1, sensitive to PGT121.414.LS neutralisation, but resistant to both PGDM1400LS and VRC07-523LS (appendix p 9).

The combination predicted serum 80% inhibitory dose titre (PT_80_) was calculated by use of an additive model as described previously.^12^

Serum concentrations of PGDM1400LS, VRC07-523LS, and PGT121.414.LS were measured by validated anti-idiotype binding antibody multiplex assay to quantify each mAb individually as described previously^9,13,14^ and in the appendix (pp 6–7).

To assess the potential development of anti-drug antibodies, serum samples were analysed by use of a tiered approach, as described previously^15^ and in the appendix (pp 6–7).

### Outcomes

The primary endpoints were safety and tolerability of PGDM1400LS, VRC07-523LS, and PGT121.414.LS when administered in sequence intravenously or subcutaneously; the serum concentrations and pharmacokinetics of PGDM1400LS, VRC07-523LS, and PGT121.414.LS after each three-mAb administration; and the individual mAb-specific serum neutralising activity after each three-mAb administration of PGDM1400LS, VRC07-523LS, and PGT121.414.LS. Secondary endpoints included: serum concentrations of PGDM1400LS, VRC07-523LS, and PGT121.414.LS; serum neutralisation titres against a panel of viruses; and anti-drug antibodies.

### Statistical analysis

Safety data, including adverse events and laboratory abnormalities, were summarised by treatment group by use of descriptive statistics. For pharmacokinetics and neutralisation titre data, summary statistics including geometric mean and associated 95% CIs were calculated for each mAb at each timepoint. To assess the combined effect of the three mAbs, we analysed the magnitude and breadth of neutralisation against a panel of AMP placebo pseudoviruses.^16^

A population pharmacokinetics analysis was done on the serum concentration data, including those collected after single administration of PGDM1400LS in part A^9^ and those after repeated triple administrations of PGDM1400LS, PGT121.414.LS, and VRC07-523LS in part B. All data were analysed according to participants’ assigned treatment in the modified intention-to-treat cohort, which included all enrolled participants receiving at least the first infusion. For each mAb, serum concentrations after intravenous or subcutaneous administration exhibited bi-exponential decay and were described by an open two-compartment disposition model with first-order elimination from the central compartment.

Prediction of prevention efficacy for the triple administrations of PGDM1400LS, PGT121.414.LS, and VRC07-523LS was done according to steps described in previously published work^17,18^ on the basis of data from this trial and previous trials of the three mAbs.^19^ Briefly, serum concentrations of each single mAb over time were first simulated by use of the popPK models of the three mAbs as described by Huang and colleagues.^19^ For each individual in an AMP-like simulated trial, the corresponding predicted PT_80_ titre of each mAb against the panel of viruses acquired by placebo recipients in the AMP trials^3^ was calculated by use of their simulated serum concentration divided by the 80% inhibitory concentration (IC_80_) of the mAb against each virus. The prevention efficacy of the mAb combination over time was then predicted on the basis of the conservative assumption that the PT_80_ values needed for a fixed amount of protection are double in humans compared to the non-human challenge study.^20^ Pharmacokinetic modelling was done with Monolix (version 2020R1, Lixoft SAS, Antony, France); all other analyses used R (version 4.0.4).

### Role of the funding source

The funder of the study had a role in study design, data interpretation, and writing of the report.

## Results

**Figure 1:**
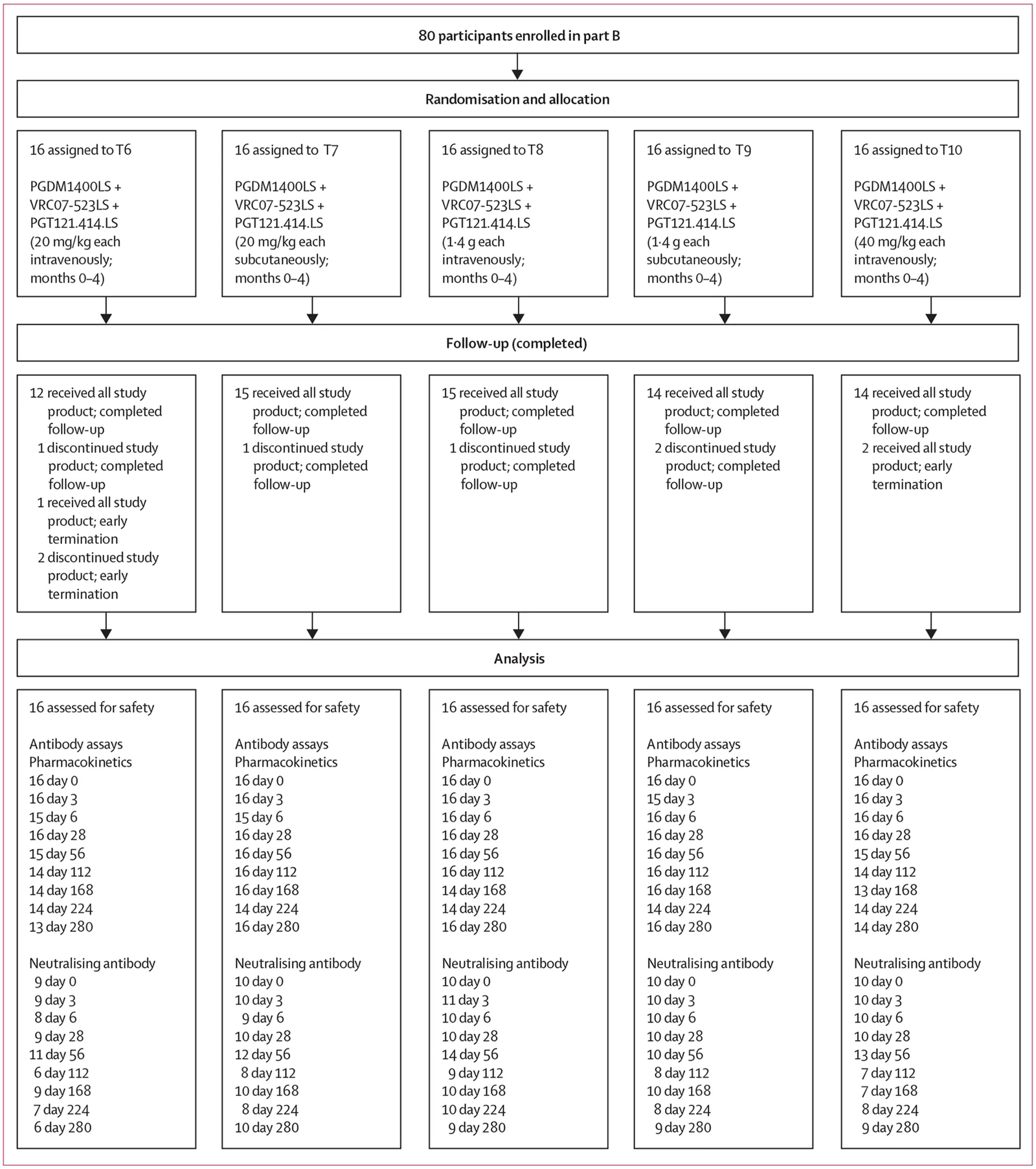
Trial profile Part B evaluated PGDM1400LS in combination with VRC07-523LS and PGT121.414.LS. Enrolment was restricted to a maximum of one participant per day across these groups for the first eight participants. After safety review, enrolment resumed without restrictions for the remaining participants in infusion groups T6 to T9. T10 (intravenous 40 mg/kg) was enrolled after review of safety data from T6 to T9. T6 etc=infusion group.

Study enrolment was completed in October, 2022, with a total of 95 participants (figure 1). Of these, 80 participants were enrolled in part B. Follow-up study visits in part B were completed on July 19, 2023. Participant demographics are shown in table 1.

Four participants terminated the study early: two participants could not be contacted, one withdrew their consent because they were unable to make the time commitment for the study, and one relocated. Five participants discontinued the study product: two had unsolicited adverse events deemed by the investigator to be related to study product (one pre-syncopal episode [infusion-related reactions] and one delayed urticaria at subcutaneous infusion sites [mAb reaction]; table 2); one had an unsolicited adverse event (temporomandibular joint syndrome) deemed unrelated to study product but associated with a new pattern of headaches that was deemed related to study product; two reported erythema at the infusion site (one participant reported a grade 2 erythema at the PGT121.414LS infusion site and another participant reported a grade 3 erythema at the PGDM1400LS infusion site and a grade 3 erythema at the VRC07-523LS infusion site).

In part B, most related solicited local adverse events observed were reported as grade 1 to 2 in subcutaneous infusion groups (T7 and T9; table 3, appendix p 11). Overall, related systemic solicited adverse events were predominantly grade 1 to 2 and were reported by a total of 35 (43·8%) participants (table 3). The most common maximum related systemic adverse events reported were malaise or fatigue (53/169=31·4%), headache (43/169=25·4%), and myalgia (21/169=12·4%). There were no significant differences between the local or systemic solicited adverse events reported after the first and second study product administrations.

A total of four unsolicited adverse events deemed related to study product occurred in four participants. These events included a grade 2 infusion site erythema in T7 and three mAb reactions: a localised infusion site urticaria in T9 and two grade 2 infusion-related reactions, one in T6 and another one in T8 (table 2). Overall, these events were promptly managed and resolved without further complications (table 2).

Ten participants reported a total of 47 local unsolicited adverse events deemed not related to study product and attributed to study procedure. These events included infusion site erythema, three grade 1 in T7, seven grade 2 (one in T6, four in T7, and two in T9), and four grade 3 in T7; infusion site induration, four grade 1 (one in T7 and three in T9) and two grade 2 in T9; infusion site pruritus, five grade 1 in T9; infusion site pain, eight grade 1 (two in T6, three in T7, and three in T9) and three grade 2 (one in T7 and two in T9); infusion site bruising, one grade 1 in T10; infusion site inflammation, one grade 1 in T8; and infusion site swelling, six grade 2 (four in T7 and two in T9) and three grade 3 (one in T7 and two in T9).

There were no serious adverse events, no pregnancies, no HIV seroconversions reported and no study safety pauses. One participant reported a single social impact event related to personal relationships, which was categorised as having a minimal effect on the participant’s quality of life.

Of the 80 participants who received the enrolment infusion, 71 (89%) returned for the second infusion and completed the acceptability questionnaire; nine discontinued before dose 2 (one withdrew consent as the participant was unable to make the time commitment to the study, one relocated, two could not be contacted, two reported related unsolicited adverse events, one reported a not related unsolicited adverse event and two reported solicited adverse events; see table 2). Most participants did not report any discomfort (59 [74%] of 80, post-first mAb combination and 57 [80%] of 71, post-second mAb combination). Additionally, most of the participants who had some discomfort reported it as mild (15 [71%] of 21, post-first mAb combination and 11 [79%] of 14, post-second mAb combination). All participants who had discomfort assessed the degree of discomfort as acceptable.

Only two participants reported that they would not be willing to receive intravenous or subcutaneous combination infusions to protect themselves from getting a serious disease, such as HIV, if they were at high likelihood for acquisition (one [1%] of 80, post-first mAb combination and one [1%[ of 71, post-second mAb combination). Most participants stated that they would recommend receiving the intravenous or subcutaneous infusion to a friend who is at high likelihood of acquiring HIV (73 [91%] of 80, post-first mAb combination and 65 [92%] of 71, post-second mAb combination; appendix p 13).

For each mAb, descriptively, higher doses resulted in higher initial mAb concentrations, but all eventually declined over time. For the same dosages, concentrations were lower after subcutaneous administration compared with intravenous owing to less than 100% subcutaneous bioavailability of these mAbs. Peak concentrations were observed at the first timepoint post-intravenous administration whereas at the second timepoint around day 6 post-subcutaneous administrations. Across all groups, PGT121.414.LS generally displayed higher and more sustained concentrations compared with the other mAbs (appendix p 22).

**Figure 2:**
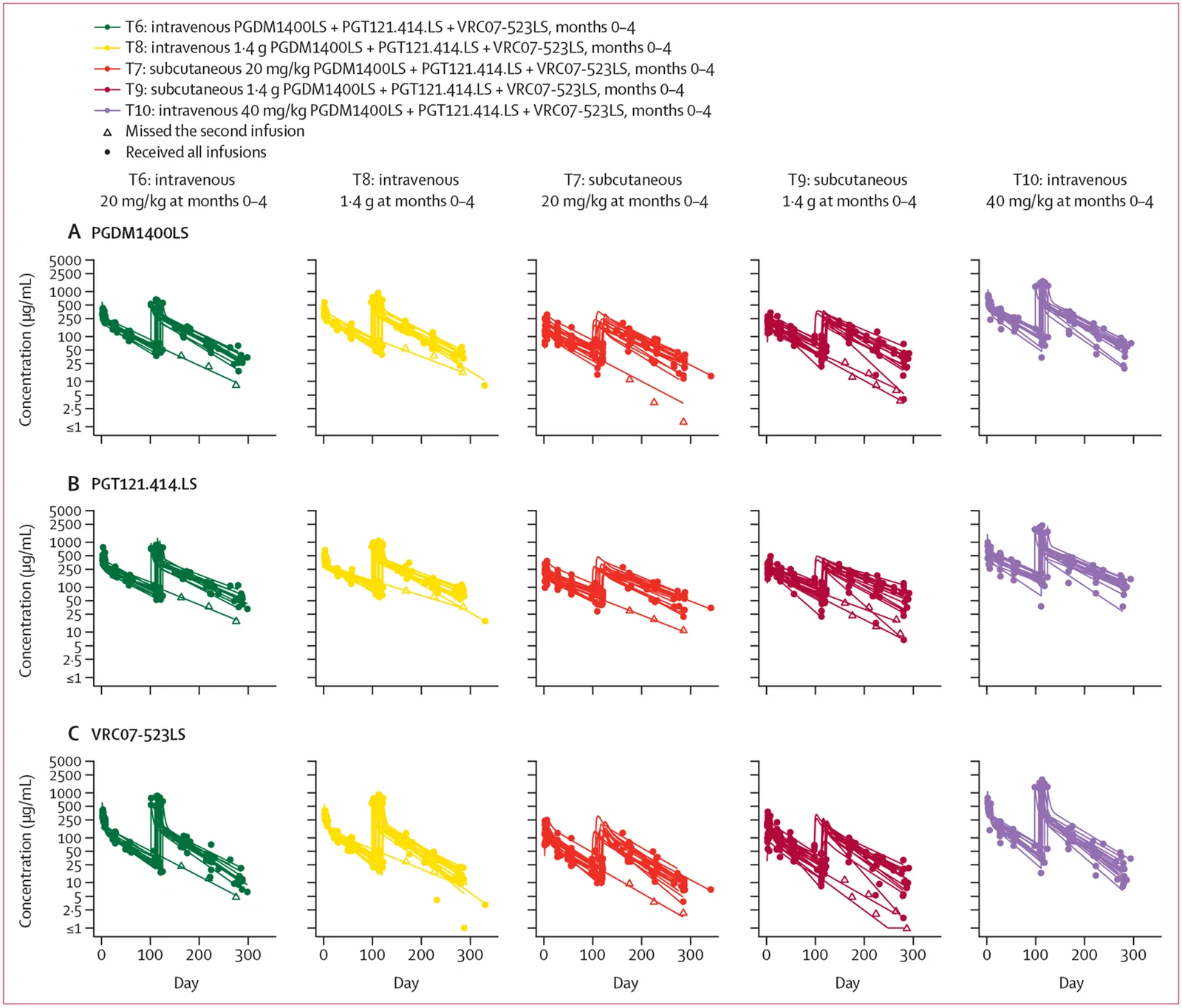
Observed and pharmacokinetic model-predicted individual-level concentrations over time for PGDM1400LS, PGT121.414.LS, and VRC07-523LS Observed (symbol) and predicted (line) serum concentrations of PGDM1400LS (A), PGT121.414.LS (B), and VRC07-523LS (C) are shown for each of the five treatment groups for each participant. Filled circles=observed concentrations from participants who received all intended study product administrations. Open triangles=subsequently observed concentrations from participants who missed the previous study product administration. T6 etc=infusion group.

For PGDM1400LS, two popPK models, one with and one without incorporating the effect of single versus combination administration were considered. The former model provided a better model fit and was deemed the final model that describes PGDM1400LS PK based on data from both part A and part B (appendix p 15). For PGT121.414LS and VRC07-523LS, only one popPK model for combination administration was considered as no single administration data for these two mAbs were available from this study (appendix pp 17–18). Based on these pharmacokinetics models, the population-level (mean) estimates of clearance (L/day), central volume (L), intercompartmental clearance (L/day), and peripheral compartment volume (L) were 0·13, 2·40, 0·42, and 4·69, respectively, for VRC07-523LS; 0·05, 1·66, 0·43, and 2·75, respectively, for PGT121.414. LS; and 0·07, 2·54, 0·46, and 2·80, respectively, for PGDM1400LS (when co-administered). For all three mAbs, the model diagnostic results suggested that the final popPK models provided a reliable description of the pharmacokinetic data (appendix p 23). Reasonable prediction of mAb concentrations for each individual were achieved (figure 2), with the observed data falling within the 90% prediction intervals of the simulated data (appendix p 25).

On the basis of the pharmacokinetic models reparametrised in terms of volume of distribution, rate constant, distribution, and elimination half-lives (appendix pp 19–21), the estimated elimination half-life of PGDM1400LS was 53 days (95% CI 51·4–55·4) and was not significantly influenced by co-administration with VRC07-523LS and PGT121.414.LS. Compared with intravenous administration, the bioavailability of PGDM1400LS administered subcutaneously was 78·3% (95% CI 68·4–88·3). The estimated elimination half-life of PGT121.414.LS was 65 days (61·8–68·1), with subcutaneous bioavailability of 75·1% (66·6–83·6). The estimated elimination half-life of VRC07-523LS was 44 days (95% CI 41·6–45·8), with subcutaneous bioavailability of 73·8% (63·3–84·3).

The median baseline weight across all treatment groups was 65·8 kg, with weights ranging from 44·3 kg to 110·8 kg across all treatment groups, falling within a consistent range of 65–71 kg (appendix p 26). Similar pharmacokinetic patterns (appendix p 27) and distributions of pharmacokinetic parameter estimates (appendix p 30) were observed between the weight-based and fixed-dose intravenous and subcutaneous regimens (T6 *vs* T8, T7 *vs* T9).

Potent neutralisation was observed against the three mAb-specific viruses across all five groups receiving the combination of PGDM1400LS, PGT121.414. LS, and VRC07-523LS, administered either intravenously or subcutaneously. For example, for the 20 mg/kg, 20 mg/kg, and 20 mg/kg intravenous group (T6), median ID_80_ serum titres against the PGDM1400LS-specific virus were 4039 at 2 months post-first administration, 6031 at 2 months post-second administration, and 504 at 6 months post-second administration (appendix p 31). Median ID_80_ titres against the PGT121.414.LS-specific virus were 9311, 14051, and 2210, whereas against the VRC07-523LS-specific virus, the titres were 76, 85, and 23 at the same timepoints (appendix p 31). The relatively lower VRC07-523LS-specific ID_80_ titres were attributed to the virus’s inherent reduced sensitivity and the lower expected concentrations of VRC07-523LS (appendix p 29). The same pattern was seen for ID_50_ (appendix p 34).

The triple combination regimens showed broad and potent neutralisation against the panel of 11 AMP placebo viruses at both 2 months post-first and 2 months post-second administration (appendix pp 35–37). In the 20 mg/kg, 20 mg/kg, and 20 mg/kg intravenous group (T6), median ID_80_ titres across viruses and participants were 5913 and 4832 at these respective timepoints, whereas in the 40 mg/kg, 40 mg/kg, and 40 mg/kg intravenous group (T10), the titres were 11 391 and 9673. The 1·4 g, 1·4 g, and 1·4 g intravenous group (T8) exhibited ID_80_ titres similar to the T6 group, but lower titres were observed in the subcutaneous groups (T7 and T9) owing to the anticipated less-than-100% bioavailability of mAbs with subcutaneous administration.

**Figure 3:**
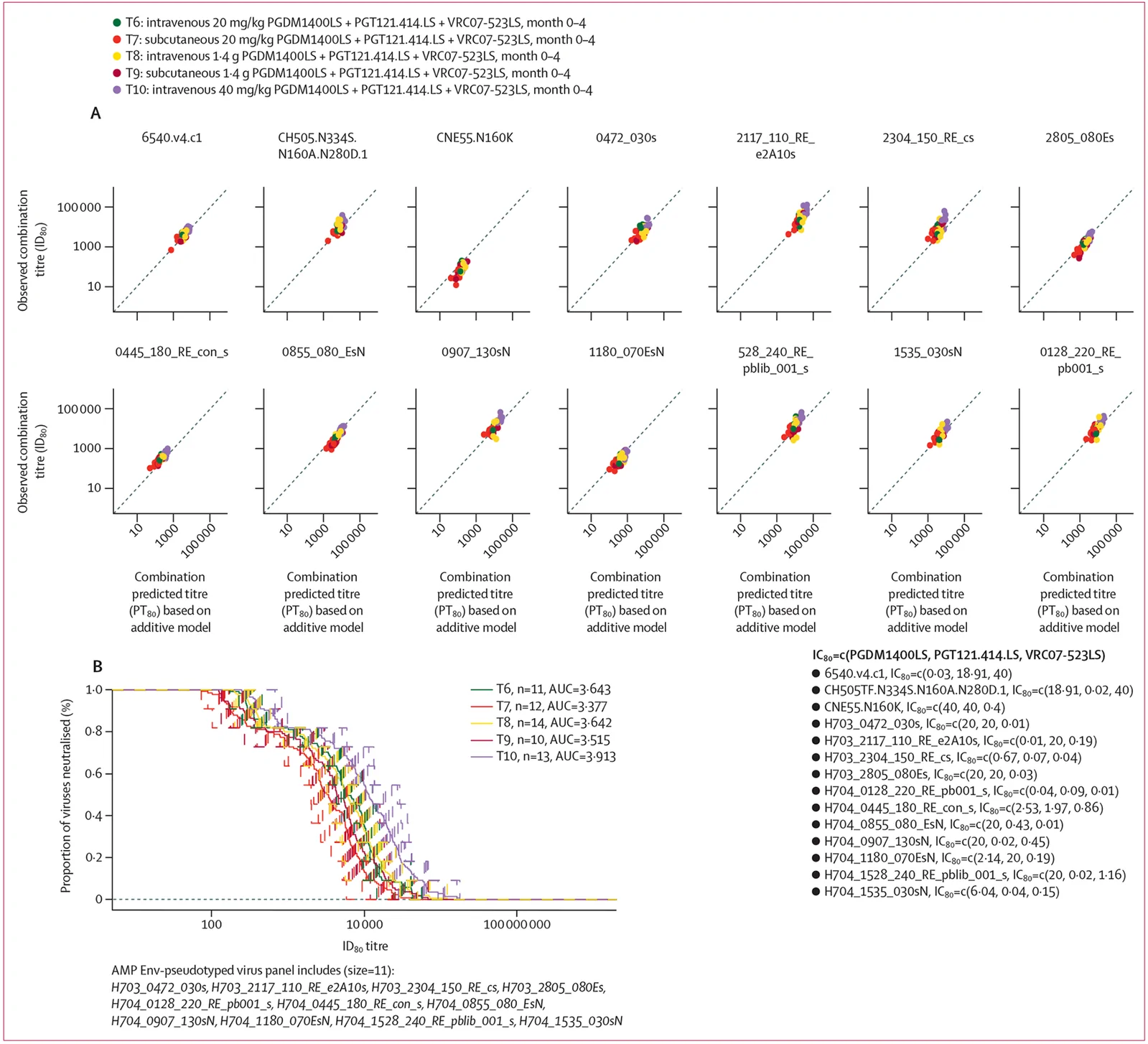
Observed combination serum ID_80_ titre versus PT_80_ against three mAb-specific viruses and a multi-clade panel of 11 envelope pseudo-typed viruses at month 2 and individual participant and group-mean magnitude-breadth curves against the multi-clade panel at month 2 (A) PT_80_ of the combination antibody was calculated under the additive neutralisation interaction model by adding the PT_80_ of each mAb against a given virus with specific in-vitro neutralisation potency indicated by IC_80_. (B) Individual participant (dashed curves) and group mean (solid curves). AUC is calculated for the group-mean magnitude-breadth curve. AUC=area under the curve. ID_80_=80% inhibitory dose titre. mAb=monclonal antibody. PT_80_=predicted combination serum 80% inhibitory dose titre. T6 etc=infusion group.

In addition, the triple mAb combinations maintained expected neutralisation activity against both the mAb-specific viruses and the 11 AMP placebo viruses following intravenous or subcutaneous administration, as shown by the concordance between the combination PT_80_ and the observed combination ID_80_ (figure 3A). As anticipated, the mean magnitude-breadth area under the receiver operating characteristic curve was lowest in the subcutaneous infusion groups (T7 and T9) and higher in the intravenous infusion groups (T6, T8, and T10; figure 3B).

The prevention efficacy of the triple-mAb combination administered intravenously at a 6-monthly fixed dosing of 3·2 g, 1·6 g, and 1·6 g of VRC07-523LS, PGDM1400LS, and PGT121.414.LS was predicted to be 89·4% (95% CI 83·8–95) against the 47 clade C viruses and 90·3% (83·3–97·2) against the 70 predominantly clade B viruses isolated from AMP placebo recipients (appendix p 38). As an internal validation, the predicted prevention efficacy of the VRC01 regimen used in AMP (30 mg/kg intravenous) was 26% against the clade C viruses and 32% against the predominantly clade B viruses, closely assimilating what was observed in AMP.^3^

Only one individual exhibited a tier II-confirmed positive antigrug antibodies response to PGDM1400LS at a post-baseline visit (1·05% in T7, 20 mg/kg subcutaneous combination; appendix p 39). However, this response was also present at baseline, and the post-infusion antidrug antibodies titre at day 280 was lower than the baseline titre, supporting that this response was treatment independent.

## Discussion

This phase 1 study of the triple mAb combination (PGDM1400LS, PGT121.414.LS, and VRC07-523LS) showed safety and promising pharmacokinetic profiles and neutralisation. All three mAbs maintained their expected neutralisation activity against the mAb-specific viruses and the 11 contemporaneous viruses, regardless of whether the mAbs were administered intravenously or subcutaneously. By targeting three distinct epitopes on the HIV-1 envelope, this mAb combination strategy is expected to offer broader protection against diverse HIV-1 strains and reduce the risk of viral escape. Based on these encouraging data, a phase 2a qualifying trial HVTN 206/HPTN 114 of combination mAb regimens of these three mAbs is ongoing (NCT06812494). Findings from that study will further inform the design and planning of future combination mAb efficacy trials.^21^

Findings from this study, together with those from others^7,8,22–24^ contribute to the growing body of evidence supporting the potential of mAb regimens for HIV prevention. PGDM1400 is one of two V2-targeting antibodies in clinical trials, alongside CAP256-VRC26.25^25^ and its half-life-optimised version, CAP256V2LS.^26^ Although this class of mAb has poor neutralisation potency effectiveness against clade B viruses, it offers superior neutralisation potency against clade C and CRF01_AE viruses compared with other mAb classes.^27^

In this study, the extended half-lives for all three mAbs, particularly notable for PGDM1400LS (53 days) and PGT121.414.LS (65 days), provide a strong rationale for a 6-monthly intravenous administration schedule, surpassing the preferred dosing interval for mAbs as specified in the Preferred Product Characteristics.^28^ The slightly shorter half-life for VRC07-523LS might be compensated via a higher relative dose in the combination as illustrated in Huang and colleagues’ study^18^ and implemented in the ongoing HVTN 206/HPTN 114 trial (NCT06812494) by use of 6·4 g for VRC07-523LS and 3·2 g for the other two mAbs to potentially achieve high efficacy at around 90%. Subcutaneous administration of antibodies might pose substantial implementation challenges as large volumes and long infusion times are required to administer the required dose to achieve sufficient neutralisation potency for HIV prevention. In addition, this study also provided valuable data on the similar pharmacokinetics patterns observed between weight-based and fixed-dose regimens that support the potential implementation of simplified fixed-dose strategies.^29^

We aim to use this exact mAb combination in upcoming efficacy trials to assess its potential in preventing HIV-1 acquisition. In addition to its demonstrated safety, extended half-life, and complementary epitope targeting, a major rationale for advancing this combination into efficacy testing is to address a key scientific question: whether the PT_80_ threshold for HIV protection, established in the AMP trial via a single CD4 binding site mAb (VRC01), can be applied to mAb combinations that target distinct epitopes, including the V3-glycan and V2-apex? If PT_80_ is not directly transferable to such combinations, this would necessitate a recalibration of this widely referenced correlate of protection.^17^ Resolving this question is particularly important for the field of HIV vaccine development, where the overarching goal is to elicit similarly potent antibody combinations through active immunisation.

There are several limitations of this study. First, although the trial was designed to allow delineation of local adverse events and infusion-related reactions that might occur after the administration of each single mAb, it was challenging to pinpoint the exact mAb responsible for systemic symptoms that might occur after the completion of the combination mAb administration. In this regard, data from single mAb administration groups provide the most reliable assessment for mAb-specific systemic symptoms as reported previously for each of the three mAbs.^7,9,30^ Second, although this study was done in both the USA and Africa, the sample size was too small to allow statistically powered assessments of the effect of region on mAb pharmacokinetics while controlling for other known demographic, clinical, and host genetic factors that might modulate pharmacokinetic^31^ In the future, cross-protocol analyses of data pooled from multiple multi-region clinical trials might be done to assess the region effect. Third, the range of bodyweight observed in this study was limited to 44–111 kg; it did not cover a wider range of possible weights. Therefore, the observed similarity of pharmacokinetics profiles between the weight-based and fixed-dose regimens might not apply to extreme bodyweights that are not represented in the population of this study. Fourth, there was no control or placebo group in this study, making the acceptability questionnaire more difficult to interpret. Lastly, it is important to note that the magnitude-breadth virus panels used for neutralisation assessment, while diverse, were selected for sensitivity to multiple mAb epitopes as a means to robustly assess retention of expected neutralising activity post-administration. Therefore, the observed neutralisation breadth in vivo might not fully represent the diversity of circulating HIV-1 strains. Although several analyses have shown the excellent capability of the use of concentration and in-vitro neutralisation data to predict neutralisation in vivo against larger panels of viruses,^32,33^ future studies could consider use of a more representative virus panel to characterise neutralisation potency and breadth of combination mAbs by use of samples from mAb recipients.

Given its favourable safety and pharmacokinetic profile, this broadly neutralising antibody combination might represent a candidate for future efficacy trials. However, the prevention landscape has advanced considerably, with alternative long-acting pre-exposure prophylaxis strategies such as injectable cabotegravir and lenacapavir showing very high levels of protection. Any role for broadly neutralising antibody-based prevention must therefore be evaluated relative to these established and emerging benchmarks.

In summary, this study strongly supports the ongoing clinical evaluation of the combination of PGDM1400LS, PGT121.414.LS, and VRC07-523LS in HVTN 206/HPTN 114. The extended half-lives, high bioavailability, potent neutralisation activity, and safety suggest a promising clinical profile. The potential for a 6-month dosing frequency could greatly improve adherence and feasibility in real-world prevention settings compared with more frequent regimens. Additionally, the similar fixed and weight-based dosing profiles offer the opportunity to streamline commercial planning and clinical protocols and minimise dosing errors, which is particularly beneficial in resource-limited settings. Future efficacy trials should aim to optimise dosing strategies, including the exploration of fixed-dose regimens that effectively balance pharmacokinetic considerations with practical implementation as we reported in 2025.^19^ Crucially, these trials will also address a key scientific question: whether the PT_80_ correlate of protection established from the AMP study using a single CD4-binding site mAb is applicable to multi-mAb combinations targeting distinct epitopes? As vaccines remain the most scalable and sustainable long-term strategy to control the HIV epidemic, confirming whether PT_80_ can serve as a universal benchmark or must be revised for antibody combinations, is essential to guide current vaccine design strategies and inform future immunogen development.

## Supplementary Material

MMC1

## Figures and Tables

**Table 1:** Baseline characteristics of participants

	T6 (n=16)	T7 (n=16)	T8 (n=16)	T9 (n=16)	T10 (n=16)	Total (n=80)
Sex						
Male	7 (44%)	7 (44%)	7 (44%)	10 (63%)	11 (69%)	42 (53%)
Female	9 (56%)	9 (56%)	9 (56%)	6 (38%)	5 (31%)	38 (48%)

Ethnicity						
Hispanic or Latinx	2 (13%)	1 (6%)	1 (6%)	0	1 (6 %)	5 (6 %)
Not Hispanic or Latinx	14 (88%)	15 (94%)	15 (94%)	16 (100%)	15 (94%)	75 (94%)

Race						
White	4 (25 %)	2 (13%)	3 (19%)	2 (13%)	4 (25%)	15 (19%)
Black or African American	12 (75%)	13 (81%)	11 (69%)	13 (81%)	10 (63%)	59 (74%)
Asian	0	0	1 (6%)	0	0	1 (1%)
Native Hawaiian or other Pacific Islander	0	0	0	0	0	0
American Indian or Alaska Native	0	0	0	0	0	0
Multiracial	0	0	1 (6%)	1 (6%)	1 (6%)	3 (4%)
Other	0	1 (6%)	0	0	1 (6%)	2 (3%)

Age, years						
<18	0	0	0	0	0	0
18–20	3 (19%)	1 (6%)	3 (19%)	3 (19%)	2 (13%)	12 (15%)
21–30	9 (56 %)	9 (56%)	7 (44%)	8 (50%)	4 (25%)	37 (46%)
31–40	2 (13%)	3 (19%)	2 (13%)	2 (13%)	7 (44%)	16 (20%)
41–50	2 (13%)	3 (19%)	4 (25 %)	3 (19%)	3 (19%)	15 (19%)
>50	0	0	0	0	0	0
Median	26	28	26	26	31	27
Range	18–46	18–46	18–45	18–48	19–45	18–48

Weight, kg	65 (44·3–99·1)	65 (47·5–110·8)	70 (52·5–101·1)	58 (48 ·4–103·7)	71 (55·7–96 ·1)	66 (44·3–110·8)

Study product administration frequencies						
Visit 2—enrolment	16 (100)	16 (100)	16 (100)	16 (100)	16 (100)	80 (100)
Visit 7—day 112	13 (81%)	15 (94%)	15 (94%)	14 (88%)	14 (88%)	71 (89%)

Data are n (%) or median (IQR). T6 etc=infusion group.

**Table 2:** Unsolicited adverse events, infusion-related reactions, and antibody reactions reported during the study

	Related unsolicited adverse event or antibody reaction	Infusion visit day	Onset*	Symptoms	Duration*	Treatment	Next mAb combination administration visit	Recommendations after investigator and protocol safety review team and safety monitoring board review and additional notes
Participant 1 (group T6): 20 mg/kg intravenous PGDM1400LS and VRC07-523LS and PGT121.414.LS each	Infusion-related reaction	0	2 h after combination mAb intravenous administration	Pre-syncope, moderate diaphoresis, headache, chills, and myalgia	Resolved the same day (time was not reported)	Benadryl and ibuprofen orally	112	Site principal investigator decided to discontinue subsequent mAb combination administrations; participant remained on study for follow-up visits
Participant 2 (group T7): 20 mg/kg subcutaneous PGDM1400LS and VRC07-523LS and PGT121.414.LS each	Unsolicited adverse event; infusion site erythema	112	6 days after combination mAb subcutaneous administration	Moderate erythema at PGDM1400LS infusion site (measured 5 cm × 4 cm)	10 days	Hydrocortisone cream	NA	Protocol safety review team and safety monitoring board reviewed this safety event and combination mAb subcutaneous infusions were administered as scheduled in the remaining participants in group 7
Participant 3 (group T8): 1·4 g intravenous PGDM1400LS and VRC07-523LS and PGT121.414.LS each	Infusion related reaction	112	60 min after combination mAb intravenous administration	Mild generalised pruritus and non-exertional dyspnoea; moderate unexplained diaphoresis, headache, chills, fatigue or malaise, and myalgia	72 h	Paracetamol and ibuprofen orally	NA	Protocol safety review team and safety monitoring board reviewed this safety event and combination mAb intravenous infusions were administered as scheduled in the remining participants in group 8
Participant 4 (group T9): 1·4 g subcutaneous PGDM1400LS and VRC07-523LS and PGT121.414.LS each	Antibody reaction (mAb reaction); delayed urticaria at subcutaneous infusion sites	0	7 days after combination mAb subcutaneous administration	Mild infusion site urticaria and localised pruritus in infusion sites of all three antibodies	8 days	Loratadine orally, benadryl orally, and hydrocortisone cream	112	Site principal investigator decided to discontinue subsequent mAb combination administrations; participant remained on study for follow-up visits

mAb=monoclonal antibody. NA=not applicable. T6 etc=infusion group.

* Times are approximate; ranges reflect duration of different symptoms.

**Table 3:** Maximum local and systemic solicited adverse events summary

	T6		T7		T8		T9		T10		Total	
	Participants	Events	Participants	Events	Participants	Events	Participants	Events	Participants	Events	Participants	Events
**Local**												

Induration												
Mild	··	··	1	3	··	··	2	6	··	··	3	9
Moderate	··	··	··	··	··	··	2	4	··	··	2	4
Severe	··	··	2	2	··	··	2	3	··	··	4	5
Erythema												
Mild	··	··	1	1	··	··	1	2	··	··	2	3
Moderate	··	··	1	4	··	··	5	14	··	··	6	18
Severe	··	··	2	4	··	··	··	··	··	··	2	4
Pain or tenderness												
Mild	1	4	6	31	3	3	12	82	2	5	24	125
Moderate	··	··	1	5	··	··	1	4	··	··	2	9
Injection site pruritus												
Mild	··	··	4	6	··	··	7	22	··	··	11	28
Moderate	··	··	··	··	··	··	1	2	··	··	1	2

**Systemic**												

Participants with at least one related solicited adverse event	8	51	5	14	10	46	8	39	4	19	35	169
Malaise or fatigue												
Mild	4	12	4	5	4	11	5	11	1	2	18	41
Moderate	2	3	1	2	3	5	··	··	1	1	7	11
Severe	··	··	··	··	··	··	··	··	1	1	1	1
Headache												
Mild	6	15	2	2	3	3	3	7	2	2	16	29
Moderate	2	3	1	1	2	7	··	··	2	3	7	14
Myalgia												
Mild	1	1	2	2	2	6	2	4	1	1	8	14
Moderate	2	3	1	1	2	2	··	··	1	1	6	7
Arthralgia												
Mild	1	1	··	··	1	1	3	5	1	3	6	10
Moderate	1	1	··	··	··	··	··	··	··	··	1	1
Chills												
Mild	1	1	··	··	1	1	2	3	2	3	6	8
Moderate	2	2	··	··	2	2	··	··	··	··	4	4
Facial flushing												
Mild	1	1	··	··	··	··	1	1	··	··	2	2
Generalised pruritus												
Mild	··	··	··	··	1	1	··	··	··	··	1	1
Nausea												
Mild	4	6	1	1	1	3	3	6	1	1	10	17
Moderate	··	··	··	··	1	1	1	2	··	··	2	3
Non-exertional dyspnoea												
Mild	··	··	··	··	1	2	··	··	··	··	1	2
Temperature (°C)												
Mild	··	··	··	··	··	··	··	··	1	1	1	1
Unexplained diaphoresis												
Mild	1	1	··	··	··	··	··	··	··	··	1	1
Moderate	1	1	··	··	1	1	··	··	··	··	2	2

T6 etc=infusion group.

## Data Availability

De-identified participant-level and aggregate-level data will be made publicly available via https://dataverse.harvard.edu/dataverse/hvtn140-hptn101.

## Research in context

### Evidence before this study

Passive infusion with a combination of broadly neutralising monoclonal antibodies (mAbs) is a potential HIV prevention intervention owing to its long-lasting protection and broad neutralisation across HIV clades. The Antibody Mediated Prevention (AMP) trials showed the efficacy of the single mAb VRC01 against sensitive HIV strains but highlighted the need for more potent and broadly targeting mAbs and possibly combinations. In addition, advances in antibody engineering, including LS modifications for extended half-life, have improved the pharmacokinetics of mAbs, offering potential for less frequent dosing regimens. However, optimal dosing strategies and administration routes to balance the pharmacokinetics, acceptability, and feasibility remain under investigation. We did a systematic search from Oct 20, 2020, to June 23, 2021, in PubMed and ClinicalTrials.gov, of studies published in English, with the search terms “PGDM1400”, “PGDM1400LS”, “VRC07–523LS”, “PGT121.414.LS”, and “VRC01”, and assessed publications for quality evidence before and during study development. We selected the mAb combination of VRC07-523LS, PGT121.414.LS, and PGDM1400LS on the basis of previous phase 1 data showing that each of these mAbs are safe, well tolerated, and retain potent neutralising activity for up to 6 months. Each mAb targets a distinct epitope (CD4bs, V3-glycan, and V2-apex, respectively), providing broad coverage. This specific multi-epitope combination was chosen on the basis of in-vitro data and modelling predicting complementary coverage, including some double and triple antibody coverage, of circulating global strains. In addition, previous studies have shown excellent safety profiles and retained neutralisation activity of the dual combinations VRC07-523LS and PGT121.414. LS and triple combinations of VRC07-523LS with the non-LS version mAbs PGT121 and PGDM1400. LS mutations extend half-life, enabling infrequent dosing, and triple combinations outperform double combinations in breadth and potency without pharmacokinetic interference or antidrug antibodies. This makes the selected trio LS-modified mAbs promising for future combination AMP (Combo AMP) efficacy trials.

### Added value of this study

This study provides key insights into the safety, tolerability, pharmacokinetics, and neutralisation activity of a combination of three LS-modified mAbs—PGDM1400LS, PGT121.414.LS, and VRC07-523LS—administered via intravenous and subcutaneous routes, using both weight-based and fixed-dose regimens. The study introduces several novel findings that advance the potential use of mAbs for HIV prevention with their efficacy to be evaluated in upcoming Combo AMP trials. Fixed-dose regimens showed similar pharmacokinetic profiles to weight-based strategies, supporting the feasibility of simplified dosing approaches that could enhance implementation, drug formulation, and manufacturing, particularly in resource-limited environments. PGDM1400LS, PGT121.414.LS, and VRC07-523LS exhibited extended elimination half-lives, suggesting the feasibility of biannual dosing schedules in future prevention strategies. Participant acceptability, coupled with minimal infusion-related adverse events, provides support for the practical deployment of mAb-based prevention strategies. Because the study showed robust neutralisation activity against a panel of HIV-1 strains in serum, it reinforces the potential of combining mAbs targeting multiple epitopes to achieve broader and more durable protection.

### Implications of all the available evidence

The findings from this study contribute to the growing body of evidence supporting combination mAbs as a promising HIV prevention modality. The demonstration of favourable safety profiles, extended durability, and practical dosing regimens supports the ongoing clinical development of mAb combinations as a viable alternative or complement to existing pre-exposure prophylaxis options. Importantly, as we move toward a Combo AMP trial using these mAbs, a central question remains: whether the predicted serum 80% inhibitory dose titre (PT_80_) correlate of protection, established for a single CD4 binding site mAb (VRC01), applies to combinations targeting three distinct epitopes. Addressing this will not only refine our understanding of mAb-mediated protection but also establish the validity of PT_80_ as a universal benchmark—an answer that is crucial for not only mAb development but also for ongoing vaccine development efforts aimed at eliciting multiepitope (ie, polyclonal) broadly neutralising responses.

## References

[1] Laher F, Salami T, Hornschuh S, Willingness to use HIV prevention methods among vaccine efficacy trial participants in Soweto, South Africa: discretion is important. BMC Public Health 2020; 20: 1669. 33160341 10.1186/s12889-020-09785-0 PMC7648553

[2] Mahomed S. Broadly neutralizing antibodies for HIV prevention: a comprehensive review and future perspectives. Clin Microbiol Rev 2024; 37: e0015222. 38687039 10.1128/cmr.00152-22 PMC11324036

[3] Corey L, Gilbert PB, Juraska M, , and the HVTN 704/HPTN 085 and HVTN 703/HPTN 081 Study Teams. Two randomized trials of neutralizing antibodies to prevent HIV-1 acquisition. N Engl J Med 2021; 384: 1003–14. 33730454 10.1056/NEJMoa2031738 PMC8189692

[4] Ko SY, Pegu A, Rudicell RS, Enhanced neonatal Fc receptor function improves protection against primate SHIV infection. Nature 2014; 514: 642–45. 25119033 10.1038/nature13612 PMC4433741

[5] Edupuganti S, Hurt CB, Stephenson KE, , and the HVTN 136/HPTN 092 Study Team. Safety, tolerability, pharmacokinetics, and neutralisation activities of the anti-HIV-1 monoclonal antibody PGT121.414.LS administered alone and in combination with VRC07-523LS in adults without HIV in the USA (HVTN 136/HPTN 092): a first-in-human, open-label, randomised controlled phase 1 trial. Lancet HIV 2025; 12: e13–25. 39667379 10.1016/S2352-3018(24)00247-9 PMC11795396

[6] Gaudinski MR, Houser KV, Doria-Rose NA, , and the VRC 605 study team. Safety and pharmacokinetics of broadly neutralising human monoclonal antibody VRC07-523LS in healthy adults: a phase 1 dose-escalation clinical trial. Lancet HIV 2019; 6: e667–79. 31473167 10.1016/S2352-3018(19)30181-X PMC11100866

[7] Sobieszczyk ME, Mannheimer S, Paez CA, , and the HVTN 130/HPTN 089 Study Team. Safety, tolerability, pharmacokinetics, and immunological activity of dual-combinations and triple-combinations of anti-HIV monoclonal antibodies PGT121, PGDM1400, 10-1074, and VRC07-523LS administered intravenously to HIV-uninfected adults: a phase 1 randomised trial. Lancet HIV 2023; 10: e653–62. 37802566 10.1016/S2352-3018(23)00140-6 PMC10629933

[8] Julg B, Stephenson KE, Wagh K, Safety and antiviral activity of triple combination broadly neutralizing monoclonal antibody therapy against HIV-1: a phase 1 clinical trial. Nat Med 2022; 28: 1288–96. 35551291 10.1038/s41591-022-01815-1 PMC9205771

[9] Seaton KE, Paez CA, Yu C, , and the HVTN 140/HPTN 101 study team. Safety, pharmacokinetics, and neutralisation activity of PGDM1400LS, a V2 specific HIV-1 broadly neutralising antibody, infused intravenously or subcutaneously in people without HIV-1 in the USA (HVTN 140/HPTN 101 part A): a first-in-human, phase 1 randomised trial. Lancet HIV 2025; 12: e405–15. 40441807 10.1016/S2352-3018(25)00012-8 PMC12266790

[10] Montefiori DC. Measuring HIV neutralization in a luciferase reporter gene assay. Methods Mol Biol 2009; 485: 395–405. 19020839 10.1007/978-1-59745-170-3_26

[11] Sarzotti-Kelsoe M, Bailer RT, Turk E, Optimization and validation of the TZM-bl assay for standardized assessments of neutralizing antibodies against HIV-1. J Immunol Methods 2014; 409: 131–46. 24291345 10.1016/j.jim.2013.11.022 PMC4040342

[12] Mayer BT, deCamp AC, Huang Y, Optimizing clinical dosing of combination broadly neutralizing antibodies for HIV prevention. PLOS Comput Biol 2022; 18: e1010003. 35385469 10.1371/journal.pcbi.1010003 PMC9084525

[13] Seaton KE, Huang Y, Karuna S, Pharmacokinetic serum concentrations of VRC01 correlate with prevention of HIV-1 acquisition. EBioMedicine 2023; 93: 104590. 37300931 10.1016/j.ebiom.2023.104590 PMC10363420

[14] Wesley MS, Chiong KT, Seaton KE, Validation of a triplex pharmacokinetic assay for simultaneous quantitation of HIV-1 broadly neutralizing antibodies PGT121, PGDM1400, and VRC07-523-LS. Front Immunol 2021; 12: 709994. 34504492 10.3389/fimmu.2021.709994 PMC8422903

[15] Bharadwaj P, Riekofski C, Lin S, Implementation of a three-tiered approach to identify and characterize anti-drug antibodies raised against HIV-specific broadly neutralizing antibodies. J Immunol Methods 2020; 479: 112764. 32070674 10.1016/j.jim.2020.112764 PMC7103756

[16] Huang Y, Gilbert PB, Montefiori DC, Self SG. Simultaneous evaluation of the magnitude and breadth of a left and right censored multivariate response, with application to HIV vaccine development. Stat Biopharm Res 2009; 1: 81–91. 20072667 10.1198/sbr.2009.0008 PMC2805400

[17] Gilbert PB, Huang Y, deCamp AC, Neutralization titer biomarker for antibody-mediated prevention of HIV-1 acquisition. Nat Med 2022; 28: 1924–32. 35995954 10.1038/s41591-022-01953-6 PMC9499869

[18] Huang Y, Zhang B, Zhang L, Dose finding in early-phase human immunodeficiency virus type 1 prevention monoclonal antibody clinical trials. Clin Trials 2025; 22: 442–51. 40616435 10.1177/17407745251347280 PMC12370288

[19] Huang Y, Zhang L, Gelderblom H, , and the VRC 605, HVTN 127/HPTN 087, HVTN 130/HPTN 089, HVTN 136/HPTN 092, and HVTN 140/HPTN 101 study teams. Fixed dosing versus weight-based dosing of HIV-1 prophylactic monoclonal antibodies in adults: a post-hoc, cross-protocol pharmacokinetics modelling study. EBioMedicine 2025; 117: 105804. 40516378 10.1016/j.ebiom.2025.105804 PMC12205768

[20] Pegu A, Borate B, Huang Y, A meta-analysis of passive immunization studies shows that serum-neutralizing antibody titer associates with protection against SHIV challenge. Cell Host Microbe 2019; 26: 336–46. 31513771 10.1016/j.chom.2019.08.014 PMC6755677

[21] Nkolola JP, Barouch DH. Prophylactic HIV-1 vaccine trials: past, present, and future. Lancet HIV 2024; 11: e117–24. 38141639 10.1016/S2352-3018(23)00264-3 PMC11736820

[22] Cohen YZ, Butler AL, Millard K, Safety, pharmacokinetics, and immunogenicity of the combination of the broadly neutralizing anti-HIV-1 antibodies 3BNC117 and 10-1074 in healthy adults: a randomized, phase 1 study. PLoS One 2019; 14: e0219142. 31393868 10.1371/journal.pone.0219142 PMC6687118

[23] Julg B, Walker-Sperling VEK, Wagh K, Safety and antiviral effect of a triple combination of HIV-1 broadly neutralizing antibodies: a phase 1/2a trial. Nat Med 2024; 30: 3534–43. 39266747 10.1038/s41591-024-03247-5 PMC11645281

[24] Mahomed S, Garrett N, Capparelli EV, Safety and pharmacokinetics of monoclonal antibodies VRC07-523LS and PGT121 administered subcutaneously for human immunodeficiency virus prevention. J Infect Dis 2022; 226: 510–20. 35134995 10.1093/infdis/jiac041 PMC9417124

[25] Doria-Rose NA, Bhiman JN, Roark RS, New member of the V1V2-directed CAP256-VRC26 lineage that shows increased breadth and exceptional potency. J Virol 2015; 90: 76–91. 26468542 10.1128/JVI.01791-15 PMC4702551

[26] Mahomed S, Garrett N, Capparelli EV, Safety and pharmacokinetics of escalating doses of neutralising monoclonal antibody CAP256V2LS administered with and without VRC07-523LS in HIV-negative women in South Africa (CAPRISA 012B): a phase 1, dose-escalation, randomised controlled trial. Lancet HIV 2023; 10: e230–43. 37001964 10.1016/S2352-3018(23)00003-6

[27] Karuna ST, Corey L. Broadly neutralizing antibodies for HIV prevention. Annu Rev Med 2020; 71: 329–46. 31986089 10.1146/annurev-med-110118-045506

[28] WHO. WHO preferred product characteristics for monoclonal antibodies for HIV prevention. Geneva: World Health Organization; 2022. https://www.who.int/publications/i/item/9789240045729 (accessed Dec 1, 2024).

[29] Huang Y, Zhang L, Gelderblom HC, , eds. Fixed dosing versus body-weight-based dosing of HIV-1 prophylactic monoclonal antibodies in adults: a post-hoc, cross-protocol pharmacokinetics modelling study. eBiomedicine 2025; 117: 105804. 40516378 10.1016/j.ebiom.2025.105804 PMC12205768

[30] Walsh SR, Gay CL, Karuna ST, , and the HVTN 127/HPTN 087 Study Team. Safety and pharmacokinetics of VRC07-523LS administered via different routes and doses (HVTN 127/HPTN 087): a phase I randomized clinical trial. PLoS Med 2024; 21: e1004329. 38913710 10.1371/journal.pmed.1004329 PMC11251612

[31] Thomas VA, Balthasar JP. Understanding inter-individual variability in monoclonal antibody disposition. Antibodies (Basel) 2019; 8: 56. 31817205 10.3390/antib8040056 PMC6963779

[32] Huang Y, Naidoo L, Zhang L, Pharmacokinetics and predicted neutralisation coverage of VRC01 in HIV-uninfected participants of the Antibody Mediated Prevention (AMP) trials. EBioMedicine 2021; 64: 103203. 33493795 10.1016/j.ebiom.2020.103203 PMC7841500

[33] Huang Y, Zhang Y, Bailer R, Brief report: prediction of serum HIV-1 neutralization titers after passive administration of VRC01. J Acquir Immune Defic Syndr 2020; 83: 434–39. 31855881 10.1097/QAI.0000000000002272 PMC7594341

