# Supplementary material for: Safety, pharmacokinetics, and neutralisation activity of PGDM1400LS, VRC07-523LS, and PGT121.414.LS infused intravenously or subcutaneously (HVTN 140/HPTN 101 part B): a phase 1, randomised trial
Source: Lancet HIV. Author manuscript; Available in PMC 2026 Sep 2. (PMC13537269; doi:10.1016/S2352-3018(25)00356-X)
Supplement: Supplementary file 1 [file NIHMS2176821-supplement-MMC1.pdf]

THE LANCET
HIV

Supplementary appendix

This appendix formed part of the original submission and has been peer reviewed. We post it as supplied by the authors.

Supplement to: Mahomed S, Paez CA, Huang Y, et al. Safety, pharmacokinetics, and neutralisation activity of PGDM1400LS, VRC07-523LS, and PGT121.414.LS infused intravenously or subcutaneously (HVTN 140/HPTN 101 part B): a phase 1, randomised trial. *Lancet HIV* 2026; published online May 11. https://doi.org/10.1016/S2352-3018(25)00356-X.

Supplemental Material for Safety, pharmacokinetics, and neutralization activity of PGDM1400LS, VRC07-523-LS, and PGT121.414.LS infused intravenously or subcutaneously: a Phase 1 randomized trial (HVTN 140/HPTN 101 Part B)

Contents

HVTN 140/HPTN 101 Study Team 3

Supplemental Methods 6

Supplemental Table 1. List of clinical research sites (CRS) and enrollment. 8

Supplemental Table 2. Partial* inclusion/exclusion criteria 8

Supplemental Table 3. AE grading exceptions. 9

Supplemental Table 4. In vitro neutralization IC50 and IC80 concentrations (mcg/mL) of the clinical lot of each mAb against mAb-specific pseudoviruses. 9

Supplemental Table 5. in vitro neutralization IC50 and IC80 concentrations (mcg/mL) of the clinical lot of each mAb against pseudoviruses derived from AMP placebo recipients who acquired HIV. 10

Supplemental Table 6. Maximum local solicited AE in participants by administration route. ... 11

Supplemental Table 7. Summary of procedure acceptability of Part B participants. 13

Supplemental Table 8. PGDM1400LS popPK model (Parameterization: CL, Vc, Q and Vp): parameter estimates, corresponding 95% CIs, and % RSEs from a two-compartment population-level model with (Panel A) and without (Panel B) a fixed effect of single vs. co-administration on the PK parameters. 15

Supplemental Table 9. PGT121.414.LS final popPK model (Parameterization: CL, Vc, Q and Vp): parameter estimates, corresponding 95% CIs, and % RSEs from a two-compartment population-level model. 17

Supplemental Table 10. VRC07-523LS final popPK model (Parameterization: CL, Vc, Q and Vp): parameter estimates, corresponding 95% CIs, and % RSEs from a two-compartment population-level model. 18

Supplemental Table 11. PGDM1400LS popPK model (Parameterization: V, k, eHL and dHL): parameter estimates, corresponding 95% CIs, and % RSEs from a two-compartment population-level model without a fixed effect of single vs. co-administration on the PK parameters. 19

Supplemental Table 12. PGT121.414.LS model (Parameterization: V, k, eHL and dHL): parameter estimates, corresponding 95% CIs, and % RSEs from a two-compartment population-level model. 20

Supplemental Table 13. VRC07-523LS model (Parameterization: V, k, eHL and dHL): parameter estimates, corresponding 95% CIs of PK parameters, and % RSEs from a two-compartment population-level model. 21

Supplemental Figure 1: Geometric mean mAb concentrations over time in Groups T1-T10... 22

Supplemental Figure 2. Goodness of fit Diagnostic plots for the popPK models of PGDM1400LS (first row), PGT121.414.LS (second row) and VRC07-523LS (last row). 23

Supplemental Figure 3. Observed mAb concentrations (dots) with the 90% prediction intervals (shaded areas) by treatment group (T1-T10) for PGDM1400LS, PGT121.414.LS and VRC07-523LS based on the final popPK models for each mAb. 25

Supplemental Figure 4: Body weight distributions in Groups T1-T10. 26

Supplemental Figure 5. PK of PGDM1400LS, PGT121.414LS, VRC07-523LS in the body-weight-based and fixed-dose IV administration regimens. 27

Supplemental Figure 6: Distribution of estimated individual-level distribution and elimination half-lives of PGDM1400LS, PGT121.414.LS and VRC07-523LS from the final popPK model of each mAb. 30

Supplemental Figure 7. Observed serum ID80 neutralization titers over time against PGDM1400LS-specific virus 6540.v4.c1 (Panel A), PGT121.414.LS-specific virus CH505TF.N334S.N160A.N280D.1 (Panel B), and VRC07-523LS-specific virus CNE55.N160K (Panel C). 31

Supplemental Figure 8: Observed ID50 neutralization tiers over time against PGDM1400LS-specific virus 6540.v4.c1 (Panel A), PGT121.414.LS-specific virus CH505TF.N334S.N160A.N280D.1 (Panel B), and VRC07-523LS-specific virus CNE55.N160K (Panel C). 34

Supplemental Figure 9. Observed serum ID80 neutralization tiers at 2 months post first study product administration against separate magnitude-breadth panels of contemporaneous clade C (A) and clade B (B) viruses obtained in the AMP trials. 35

Supplemental Figure 10: Observed ID80 neutralization tiers at 2 months post the 2nd study product administration (month 6) against a panel of contemporaneous clade C viruses (Panel A) and clade B viruses (Panel B) obtained in the AMP trials. 36

Supplemental Figure 11: Observed ID50 neutralization tiers at 2 months post the 2nd study product administration (month 6) against a panel of contemporaneous clade C viruses (Panel A) and clade B viruses (Panel B) obtained in the AMP trials. 37

Supplemental Figure 12. Predicted prevention efficacy of the triple-mAb combination VRC07-523LS + PGDM1400LS + PGT121.414.LS administered intravenously at 3.2g + 1.6g +1.6g (blue) relative to that of eight-weekly VRC01 administered intravenously at 30 mg/kg (red), against clade B and clade C viruses. 38

Supplemental Figure 13: ADA Titer. 39

HVTN 140/HPTN 101 Study Team

First names	Surname
Margaret E.	Ackerman
Maija A	Anderson
Michael	Archibald
Kathryn	Axelrod
Veronique C.	Bailey
Saman	Baral
Dan H.	Barouch
Gail	Broder
Wairimu	Chege
Shawn	Chiambah
Kelvin	Chiong
Z. Mike	Chirenje
Myron S.	Cohen
Lawrence	Corey
Sinéad	Delany-Moretlwe
Elize	Domin
Jaiden	Dumas
Julie	Dumond
Bonnie J.	Dye
Lucio	Gama
Theresa	Gamble
Fei	Gao
Kevin	Gillespie
Chelsea	Greer
Jen	Hanke
Nicholas	Hastings
Jack	Heptinstall
Nate	Holm
Yunda	Huang
Zoe	Jones
Spyros A.	Kalams
Geoffrey	Kamau
Shelly T.	Karuna
Sheetal	Kassim
Colleen	Kelley
Robert	Klitzman

Josphat	Kosgei
Gabriela	Kovacikova
Fatima	Laher
Judith	Lucas
Jonathan	Lucas
Sharana	Mahomed
Anders	McConachie
M. Juliana	McElrath
Tara	McNair
Felix	Mhlanga
Maurine D.	Miner
Nonhlanhla	Mkhize
David C.	Montefiori
Amber	Moser
Yeukai	Musodza
Carmen A.	Paez
Michael N.	Pensiero
Estelle	Piwowar-Manning
Lori	Proulx-Burns
Lynette	Purdue
April	Randhawa
Laurie	Rinn
Sheetal	Sawant
Hyman	Scott
Kelly E.	Seaton
Angelina	Sharak
Marc	Siegel
Hans M.L.	Spiegel
Kenneth R.	Stroop
Shonda E.	Sumner
Azwidihwi	Takalani
Simbarashe	Takuva
Haili	Tang
India	Tindale
Georgia D.	Tomaras
Meg	Trahey
Lenka	Tswenyana
Melissa	Turner

Joshua A.	Weiner
Haven	Wilvich
Margaret	Yacovone
Nicole	Yates
Hannah	Yellin
Ntando	Yola
Chenchen	Yu
Lu	Zhang

Supplemental Methods

Neutralizing Antibody Assays

To optimize resource utilization while maintaining statistical power, we employed a randomized sampling strategy. From the 80 participants in Part B of the study, we randomly selected a subset of 62 individuals for comprehensive neutralization testing. This sample size was determined to be sufficient to provide representative data on the neutralizing activity of the mAb combination across different dosing regimens and administration routes. These measurements were taken from specimens collected at several key visits: pre- Study Product combo-infusion, 3 days, 6 days, 1 month, and 2 months post-first combo-infusion, at the time of the second combo-infusion as well as 2, 4, and 6 months following the second combo-infusion (corresponding to visits 2, 3, 4, 5, 6, 7, 9, 10, and 11). Additionally, neutralization titers against AMP Env-pseudotyped viruses were evaluated only at visits 6 and 9, corresponding to 2 months post-first combo-infusion and 2 months post-second combo-infusion respectively. Additionally, neutralization titers against AMP Env-pseudotyped viruses were evaluated only at 2 months post-first combo-infusion and 2 months post-second combo-infusion.

This assay quantified neutralization titers against specific Env-pseudotyped viruses sensitive to the mAbs present in each treatment group, as well as a magnitude-breadth panel of tier 2 Env-pseudotyped viruses derived from the AMP trials. The serum neutralization titer was defined as the serum dilution that achieved a 50% (ID50) or 80% (ID80) reduction in relative luminescence units (RLUs) compared to virus control wells (cells + virus only), after subtracting the background RLU from wells containing only cells.

Pharmacokinetic Assessments

The endpoint measured was each mAb concentration (µg/mL) in serum samples. Standard curves specific to each antibody were included on every assay plate to ensure precise quantification, with the lower limit of quantification ranging between 50–100 ng/mL. Serum

concentrations for each of the three mAbs were measured over a 280-day period following repeated combo-infusions of PGDM1400LS, PGT121.414.LS, and VRC07-523LS. Collection time points included Day 0, 1 hour post-first combo-infusion, and subsequently on Days 3, 6, 28, 56, 112 (at the second combo-infusion), 1 hour post-second combo-infusion, Days 168 (2 months post-second combo-infusion), 224 (4 months post-second combo-infusion), and 280 (6 months post-second combo-infusion).

Anti-Drug Antibody Assays

The presence of anti-PGDM1400LS, PGT121.414.LS, and VRC07-523LS antibodies in serum were measured by a qualified bridging electrochemiluminescence assays following a previously described method (Bharadwaj P 2020). Assays were conducted in a GCLP compliant laboratory. Samples were tested in duplicate along with a panel of anti-idiotype positive and negative controls and data accepted based on meeting pre-established criteria.

Supplemental Table 1. List of clinical research sites (CRS) and enrollment.

Clinical Research Site	Total Enrolled
Hope Clinic, Atlanta, USA	5
Bridge HIV CRS, San Francisco, USA	6
Groote Schuur CRS, Cape Town, South Africa	8
George Washington University CRS, Washington DC, USA	6
KEMRI CRS, Kericho, Kenya	9
Vanderbilt University CRS, Nashville, USA	5
New Jersey Medical School CRS, Newark, USA	1
Parirenyatwa CRS, Harare, Zimbabwe	3
Seke South CRS, Harare, Zimbabwe	6
Soweto – Bara CRS, Johannesburg, South Africa	10
Spilhaus CRS, Harare, Zimbabwe	6
Ward 21 CRS, Johannesburg, South Africa	9
eThekwini CRS, Durban, South Africa	6
Total	80

Supplemental Table 2. Partial* inclusion/exclusion criteria

**Inclusion criteria**
Age of 18 through 50
Ability and willingness to provide informed consent
Good general health
At low likelihood of HIV acquisition
**Exclusion criteria**
Weight <35 kg or >115 kg
Pregnant or breastfeeding
Clinically significant medical conditions

*Full inclusion/exclusion criteria can be found in the Protocol

Supplemental Table 3. AE grading exceptions.

Unintentional Weight Loss is required to be reported as an AE only if it is considered to be potentially deleterious to the participant's health (see HVTN 140/HPTN 101 SSP)
Infusion Site Erythema or Redness and Infusion Site Induration or Swelling will not consider surface area and interference with usual social and functional activities such that:<br>▪ Grade 1 is: 2.5 to < 5 cm in diameter;<br>▪ Grade 2 is: ≥ 5 to < 10 cm in diameter;<br>▪ Grade 3 is: ≥ 10 cm in diameter OR Ulceration OR Secondary infection OR Phlebitis OR Sterile abscess OR Drainage;<br>▪ Grade 4 is: Potentially life-threatening consequences (eg, abscess, exfoliative dermatitis, necrosis involving dermis or deeper tissue)
Creatinine is required to be reported as an AE only if it is gradable per the increase from local lab ULN/reference range parameter. Do not grade elevated creatinine based on the change from the baseline parameter.
Creatinine clearance or eGFR is required to be reported based only on the reported value or if dialysis is needed. Do not grade Creatinine clearance or eGFR based on the change from the baseline parameter.
Ab reactions not represented in the DAIDS Table to be graded per the "infusion related reaction" row from the Common Terminology Criteria for Adverse Events (CTCAE) from the US DHHS (Version 5.0. Published November 27, 2017, available at https://ctep.cancer.gov/protocoldevelopment/electronic_applications/docs/ctcae_v5_quick_reference_5x7.pdf .

Supplemental Table 4. In vitro neutralization IC50 and IC80 concentrations (mcg/mL) of the clinical lot of each mAb against mAb-specific pseudoviruses.

mAb	Pseudovirus	IC50 (mcg/mL)	IC80 (mcg/mL)
PGT121.414.LS	CH505TF.N334S.N160A.N280D.1	0.01	0.02
PGDM1400LS	6540.v4.c1	0.01	0.03
VRC07-523LS	CNE55.N160K	0.07	0.4

Supplemental Table 5. in vitro neutralization IC50 and IC80 concentrations (mcg/mL) of the clinical lot of each mAb against pseudoviruses derived from AMP placebo recipients who acquired HIV.

IC50/IC80	Pseudovirus	PGDM1400LS	PGT121.414.LS	VRC07-523LS
IC50	H703_0472_030s	0.27	>10.0	< 0.01
(mcg/mL)	H703_2117_110_RE_e2A10s	< 0.01	>10.0	0.06
	H703_2304_150_RE_cs	0.03	0.02	0.01
	H703_2805_080Es	>10.0	>10.0	0.01
	H704_0128_220_RE_pb001_s	0.01	0.02	< 0.01
	H704_0445_180_RE_con_s	0.76	0.56	0.16
	H704_0855_080_EsN	>10.0	0.08	< 0.01
	H704_0907_130sN	>10.0	< 0.01	0.14
	H704_1180_070EsN	0.26	>10.0	0.04
	H704_1528_240_RE_pblib_001_s	>10.0	< 0.01	0.36
	H704_1535_030sN	1.14	0.01	0.05
IC80	H703_0472_030s	>10.0	>10.0	0.01
(mcg/mL)	H703_2117_110_RE_e2A10s	0.01	>10.0	0.19
	H703_2304_150_RE_cs	0.67	0.07	0.04
	H703_2805_080Es	>10.0	>10.0	0.03
	H704_0128_220_RE_pb001_s	0.04	0.09	0.01
	H704_0445_180_RE_con_s	2.53	1.97	0.86
	H704_0855_080_EsN	>10.0	0.43	0.01
	H704_0907_130sN	>10.0	0.02	0.45
	H704_1180_070EsN	2.14	>10.0	0.19
	H704_1528_240_RE_pblib_001_s	>10.0	0.02	1.16
	H704_1535_030sN	6.04	0.04	0.15

Supplemental Table 6. Maximum local solicited AE in participants by administration route.

	IV	SC
Pain or tenderness		
Grade 1	6	20
Grade 2	0	2
Erythema		
Grade 1	0	5
Grade 2	0	6
Grade 3	0	2
Induration		
Grade 1	0	6
Grade 2	0	3
Grade 3	0	4
Erythema and/or induration		
Grade 1	0	6
Grade 2	0	6
Grade 3	0	4
Injection site pruritis		
Grade 1	0	12
Grade 2	0	1

Only participants who experienced gradable solicited AEs are included.

Supplemental Table 7. Summary of procedure acceptability of Part B participants.

A. Month 0, 1st SPA

	Visit	T6	T7	T8	T9	T10
**1. Based on your experience of receiving the [IV infusion/SC infusion], how willing would you be to get [IV infusion/SC infusion] to protect yourself from getting a serious disease, such as HIV, if you were at risk for that disease?**						
Not willing.	2	-	-	-	-	1/16 = 6.2%
Somewhat willing.	2	4/16 = 25.0%	1/16 = 6.2%	2/16 = 12.5%	3/16 = 18.8%	1/16 = 6.2%
Very willing.	2	12/16 = 75.0%	15/16 = 93.8%	14/16 = 87.5%	13/16 = 81.2%	14/16 = 87.5%
**2. Based on your experience to date, if you had a choice of methods to protect yourself from HIV infection, which would you prefer**						
Intramuscular (IM) injections (injections directly into a muscle; for example most vaccines are administered intramuscularly).	2	4/16 = 25.0%	4/16 = 25.0%	8/16 = 50.0%	5/16 = 31.2%	5/16 = 31.2%
Intravenous (IV) infusions.	2	8/16 = 50.0%	1/16 = 6.2%	4/16 = 25.0%	-	7/16 = 43.8%
Other preventative methods such as condoms and oral medications.	2	2/16 = 12.5%	-	3/16 = 18.8%	1/16 = 6.2%	4/16 = 25.0%
Subcutaneous (SC) infusions.	2	1/16 = 6.2%	8/16 = 50.0%	1/16 = 6.2%	7/16 = 43.8%	-
Subcutaneous (SC) injections (injections given just under the skin; examples include insulin and TB tests).	2	1/16 = 6.2%	3/16 = 18.8%	-	3/16 = 18.8%	-
**3. Did you experience any discomfort from the [IV infusion/SC infusion]**						
No.	2	14/16 = 87.5%	12/16 = 75.0%	12/16 = 75.0%	9/16 = 56.2%	12/16 = 75.0%
Yes.	2	2/16 = 12.5%	4/16 = 25.0%	4/16 = 25.0%	7/16 = 43.8%	4/16 = 25.0%
**3b. How would you rate your level of discomfort from the [IV infusion/SC infusion]**						
Answered No to 3.	2	14/16 = 87.5%	12/16 = 75.0%	12/16 = 75.0%	9/16 = 56.2%	12/16 = 75.0%
Mildly uncomfortable.	2	1/16 = 6.2%	4/16 = 25.0%	3/16 = 18.8%	5/16 = 31.2%	2/16 = 12.5%
Moderately uncomfortable.	2	1/16 = 6.2%	-	1/16 = 6.2%	2/16 = 12.5%	2/16 = 12.5%
**3c. This level of discomfort is. . .**						
Acceptable.	2	2/16 = 12.5%	4/16 = 25.0%	5/16 = 31.2%	7/16 = 43.8%	3/16 = 18.8%
Answered No to 3.	2	14/16 = 87.5%	12/16 = 75.0%	11/16 = 68.8%	9/16 = 56.2%	13/16 = 81.2%
**7. Based on today's experience, would you recommend receiving the [IV infusion/SC infusion] to a friend who is at risk for HIV**						
Don't know.	2	1/16 = 6.2%	1/16 = 6.2%	2/16 = 12.5%	2/16 = 12.5%	-
No.	2	1/16 = 6.2%	-	-	-	-
Yes.	2	14/16 = 87.5%	15/16 = 93.8%	14/16 = 87.5%	14/16 = 87.5%	16/16 = 100.0%

B. Month 4, 2nd SPA

	Visit	T6	T7	T8	T9	T10
**1. Based on your experience of receiving the [IV infusion/SC infusion], how willing would you be to get [IV infusion/SC infusion] to protect yourself from getting a serious disease, such as HIV, if you were at risk for that disease?**						
Not willing.	7	1/13 = 7.7%	-	-	-	-
Somewhat willing.	7	2/13 = 15.4%	2/15 = 13.3%	1/15 = 6.7%	1/14 = 7.1%	1/14 = 7.1%
Very willing.	7	10/13 = 76.9%	13/15 = 86.7%	14/15 = 93.3%	13/14 = 92.9%	13/14 = 92.9%
**2. Based on your experience to date, if you had a choice of methods to protect yourself from HIV infection, which would you prefer**						
Don't know.	7	-	-	1/15 = 6.7%	1/14 = 7.1%	-
Intramuscular (IM) injections (injections directly into a muscle; for example most vaccines are administered intramuscularly).	7	5/13 = 38.5%	4/15 = 26.7%	5/15 = 33.3%	6/14 = 42.9%	7/14 = 50.0%
Intravenous (IV) infusions.	7	6/13 = 46.2%	1/15 = 6.7%	4/15 = 26.7%	-	7/14 = 50.0%
Other preventative methods such as condoms and oral medications.	7	1/13 = 7.7%	-	2/15 = 13.3%	1/14 = 7.1%	-
Subcutaneous (SC) infusions.	7	-	7/15 = 46.7%	1/15 = 6.7%	4/14 = 28.6%	-
Subcutaneous (SC) injections (injections given just under the skin; examples include insulin and TB tests).	7	1/13 = 7.7%	3/15 = 20.0%	2/15 = 13.3%	2/14 = 14.3%	-
**3. Did you experience any discomfort from the [IV infusion/SC infusion]**						
No.	7	11/13 = 84.6%	11/15 = 73.3%	13/15 = 86.7%	9/14 = 64.3%	13/14 = 92.9%
Yes.	7	2/13 = 15.4%	4/15 = 26.7%	2/15 = 13.3%	5/14 = 35.7%	1/14 = 7.1%
**3b. How would you rate your level of discomfort from the [IV infusion/SC infusion]**						
Answered No to 3.	7	11/13 = 84.6%	11/15 = 73.3%	13/15 = 86.7%	9/14 = 64.3%	13/14 = 92.9%
Mildly uncomfortable.	7	2/13 = 15.4%	2/15 = 13.3%	2/15 = 13.3%	4/14 = 28.6%	1/14 = 7.1%
Moderately uncomfortable.	7	-	2/15 = 13.3%	-	1/14 = 7.1%	-
**3c. This level of discomfort is...**						
Acceptable.	7	2/13 = 15.4%	4/15 = 26.7%	2/15 = 13.3%	5/14 = 35.7%	1/14 = 7.1%
Answered No to 3.	7	11/13 = 84.6%	11/15 = 73.3%	13/15 = 86.7%	9/14 = 64.3%	13/14 = 92.9%
**7. Based on today's experience, would you recommend receiving the [IV infusion/SC infusion] to a friend who is at risk for HIV**						
Don't know.	7	-	-	2/15 = 13.3%	4/14 = 28.6%	-
Yes.	7	13/13 = 100.0%	15/15 = 100.0%	13/15 = 86.7%	10/14 = 71.4%	14/14 = 100.0%

Supplemental Table 8. PGDM1400LS popPK model (Parameterization: CL, Vc, Q and Vp): parameter estimates, corresponding 95% CIs, and % RSEs from a two-compartment population-level model with (Panel A) and without (Panel B) a fixed effect of single vs. co-administration on the PK parameters. The model in Panel A is considered the final popPK model for PGDM1400LS in this study.

A.

Parameter	Description	Estimate	95% CI	% RSE
**Fixed Effects**				
F	Bioavilability	0.730	(0.675, 0.785)	3.86
ka	Absorption rate constant	0.277	(0.242, 0.312)	6.45
CL (L/day)	Clearance	0.072	(0.067, 0.076)	3.10
V_c (L)	Central volume	2.542	(2.331, 2.754)	4.24
Q (L/day)	Inter-compartmental clearance	0.460	(0.411, 0.508)	5.41
V_p (L)	Peripheral volume	2.803	(2.586, 3.02)	3.95
**Random Effects**				
ω_{CL}	SD, clearance	0.233	(0.197, 0.269)	7.93
ω_{V_c}	SD, central volume	0.322	(0.27, 0.374)	8.22
ω_{V_p}	SD, peripheral volume	0.197	(0.126, 0.267)	18.22
**Correlations**				
ρ_{V_pCl}	Correlation between random effects for V_p and CL	0.637	(0.386, 0.889)	20.10
**Fixed Effects**				
$\beta_{Clsingle}$	Adjusted fixed effect of CL for single- and triple- combinations	-0.305	(-0.441, -0.168)	-22.85
$\beta_{Qsingle}$	Adjusted fixed effect of Q for single- and triple- combinations	-1.595	(-2.667, -0.522)	-34.32
$\beta_{V_psingle}$	Adjusted fixed effect of V_p for single- and triple- combinations	-1.185	(-1.601, -0.768)	-17.94
**Model Parameters**				
σ $(constant)$	SE, additive	3.070	(2.234, 3.906)	13.89
σ $(proportional)$	SE, proportional	0.134	(0.122, 0.146)	4.57

B.

Parameter	Description	Estimate	95% CI	% RSE
**Fixed Effects**				
F	Bioavailability	0.755	(0.707, 0.802)	3.22
ka	Absorption rate constant	0.277	(0.243, 0.311)	6.34
CL (L/day)	Clearance	0.069	(0.065, 0.073)	3.02
V_c (L)	Central volume	2.493	(2.314, 2.672)	3.66
Q (L/day)	Inter-compartmental clearance	0.456	(0.403, 0.509)	5.92
V_p (L)	Peripheral volume	2.615	(2.374, 2.857)	4.71
**Random Effects**				
ω_{CL}	SD, clearance	0.259	(0.218, 0.3)	8.06
ω_{V_C}	SD, central volume	0.295	(0.241, 0.348)	9.19
ω_{V_P}	SD, peripheral volume	0.312	(0.229, 0.395)	13.55
**Correlations**				
$\rho_{V_P Cl}$	Correlation between random effects for V_p and CL	0.735	(0.55, 0.92)	12.85
**Error Model Parameters**				
σ ($constant$)	SE, additive	5.527	(4.565, 6.488)	8.87
σ ($proportional$)	SE, proportional	0.145	(0.135, 0.155)	3.61

RSE: relative standard error; CI: confidence interval; SD: standard deviation.

Supplemental Table 9. PGT121.414.LS final popPK model (Parameterization: CL, Vc, Q and Vp): parameter estimates, corresponding 95% CIs, and % RSEs from a two-compartment population-level model.

Parameter	Description	Estimate	95% CI	% RSE
**Fixed Effects**				
F	Bioavailability	0.777	(0.712, 0.841)	4.22
ka	Absorption rate constant	0.253	(0.225, 0.282)	5.77
CL (L/day)	Clearance	0.050	(0.047, 0.053)	3.18
V_c (L)	Central volume	1.661	(1.526, 1.797)	4.15
Q (L/day)	Inter-compartmental clearance	0.433	(0.38, 0.486)	6.20
V_p (L)	Peripheral volume	2.750	(2.567, 2.932)	3.38
**Random Effects**				
ω_{CL}	SD, clearance	0.232	(0.193, 0.27)	8.54
ω_{V_C}	SD, central volume	0.253	(0.193, 0.314)	12.12
ω_{V_P}	SD, peripheral volume	0.188	(0.117, 0.259)	19.22
**Correlations**				
$\rho_{V_C Cl}$	Correlation between random effects for V_c and CL	0.684	(0.519, 0.849)	12.30
**Error Model Parameters**				
σ $(constant)$	SE, additive	4.881	(3.722, 6.04)	12.12
σ $(proportional)$	SE, proportional	0.165	(0.154, 0.176)	3.48

RSE: relative standard error; CI: confidence interval; SD: standard deviation.

Supplemental Table 10. VRC07-523LS final popPK model (Parameterization: CL, Vc, Q and Vp): parameter estimates, corresponding 95% CIs, and % RSEs from a two-compartment population-level model.

Parameter	Description	Estimate	95% CI	% RSE
**Fixed Effects**				
F	Bioavailability	0.801	(0.714, 0.888)	5.54
ka	Absorption rate constant	0.223	(0.192, 0.254)	7.09
CL (L/day)	Clearance	0.130	(0.121, 0.139)	3.57
V_c (L)	Central volume	2.396	(2.115, 2.677)	5.98
Q (L/day)	Inter-compartmental clearance	0.424	(0.383, 0.464)	4.90
V_p (L)	Peripheral volume	4.694	(4.356, 5.032)	3.67
**Random Effects**				
ω_{CL}	SD, clearance	0.243	(0.199, 0.287)	9.23
ω_{V_C}	SD, central volume	0.395	(0.312, 0.478)	10.68
ω_{V_P}	SD, peripheral volume	0.188	(0.121, 0.256)	18.39
**Correlations**				
$\rho_{V_C Cl}$	Correlation between random effects for V_c and CL	0.651	(0.494, 0.808)	12.32
**Error Model Parameters**				
σ (*constant*)	SE, additive	1.388	(0.918, 1.857)	17.25
σ (*proportional*)	SE, proportional	0.185	(0.168, 0.201)	4.55

RSE: relative standard error; CI: confidence interval; SD: standard deviation.

Supplemental Table 11. PGDM1400LS popPK model (Parameterization: V, k, eHL and dHL): parameter estimates, corresponding 95% CIs, and % RSEs from a two-compartment population-level model without a fixed effect of single vs. co-administration on the PK parameters.

Parameter	Description	Estimate	95% CI	% RSE
**Fixed Effects**				
F	Bioavilability	0.78	(0.69, 0.89)	6.48
ka	Absorption rate constant	0.28	(0.25, 0.31)	4.93
V	Volume of distribution	2.53	(2.42, 2.65)	2.37
k	Rate constant	0.03	(0.03, 0.03)	3.36
dHL	Distribution half-life	1.80	(1.60, 2.01)	5.84
eHL	Elimination half-life	53.34	(51.40, 55.36)	1.89
**Random Effects**				
ω_F	SD, Bioavailability	0.37	(0.28, 0.48)	13.81
ω_V	SD, volume of distribution	0.16	(0.13, 0.20)	11.92
ω_k	SD, rate constant	0.27	(0.23, 0.33)	9.49
ω_{eHL}	SD, elimination half-life	0.14	(0.11, 0.18)	11.82
**Correlations**				
ρ_{eHLV}	Correlation between random effects for eHL and V	0.19	(-0.14, 0.48)	88.06
ρ_{kV}	Correlation between random effects for k and V	-0.60	(-0.77, -0.36)	17.14
ρ_{keHL}	Correlation between random effects for k and eHL	-0.56	(-0.73, -0.33)	18.34
**Error Model Parameters**				
σ (*constant*)	SE, additive	3.30	(2.66, 4.09)	11.05
σ (*proportional*)	SE, proportional	0.13	(0.12, 0.14)	4.33

RSE: relative standard error; CI: confidence interval; SD: standard deviation.

Supplemental Table 12. PGT121.414.LS model (Parameterization: V, k, eHL and dHL): parameter estimates, corresponding 95% CIs, and % RSEs from a two-compartment population-level model.

Parameter	Description	Estimate	95% CI	% RSE
**Fixed Effects**				
F	Bioavilability	0.75	(0.67, 0.84)	5.74
ka	Absorption rate constant	0.25	(0.22, 0.27)	5.22
V	Volume of distribution	1.58	(1.47, 1.69)	3.46
k	Rate constant	0.03	(0.03, 0.03)	2.74
dHL	Distribution half-life	1.38	(1.20, 1.59)	7.21
eHL	Elimination half-life	64.84	(61.77, 68.07)	2.48
**Random Effec ts**				
ω_V	SD, volume of distribution	0.17	(0.13, 0.22)	12.56
ω_k	SD, rate constant	0.17	(0.13, 0.22)	14.25
ω_{dHL}	SD, distribution half-life	0.35	(0.26, 0.48)	16.52
ω_{eHL}	SD, elimination half-life	0.18	(0.14, 0.22)	11.56
**Correlations**				
ρ_{keHL}	Correlation between random effects for k and eHL	-0.73	(-0.88, -0.44)	14.94
**Error Model Parameters**				
σ (*constant*)	SE, additive	2.92	(2.25, 3.80)	13.58
σ (*proportional*)	SE, proportional	0.15	(0.14, 0.16)	3.90

RSE: relative standard error; CI: confidence interval; SD: standard deviation.

Supplemental Table 13. VRC07-523LS model (Parameterization: V, k, eHL and dHL): parameter estimates, corresponding 95% CIs of PK parameters, and % RSEs from a two-compartment population-level model.

Parameter	Description	Estimate	95% CI	% RSE
**Fixed Effects**				
F	Bioavilability	0.74	(0.64, 0.85)	7.28
ka	Absorption rate constant	0.19	(0.17, 0.22)	6.52
V	Volume of distribution	2.13	(2.02, 2.25)	2.74
k	Rate constant	0.06	(0.05, 0.06)	3.48
dHL	Distribution half-life	1.98	(1.82, 2.16)	4.38
eHL	Elimination half-life	43.64	(41.64, 45.75)	2.40
**Random Effects**				
ω_F	SD, Bioavailability	0.41	(0.32, 0.53)	12.73
ω_V	SD, volume of distribution	0.10	(0.07, 0.15)	20.12
ω_k	SD, rate constant	0.21	(0.17, 0.26)	10.57
ω_{eHL}	SD, elimination half-life	0.17	(0.13, 0.21)	11.77
**Correlations**				
ρ_{eHLF}	Correlation between random effects for eHL and F	-0.53	(-0.79, -0.11)	32.93
ρ_{kF}	Correlation between random effects for k and F	0.65	(0.13, 0.89)	29.25
ρ_{keHL}	Correlation between random effects for k and eHL	-0.82	(-0.93, -0.58)	10.12
**Error Model Parameters**				
σ (*constant*)	SE, additive	1.54	(1.14, 2.08)	15.59
σ (*proportional*)	SE, proportional	0.17	(0.16, 0.19)	4.32

RSE: relative standard error; CI: confidence interval; SD: standard deviation.

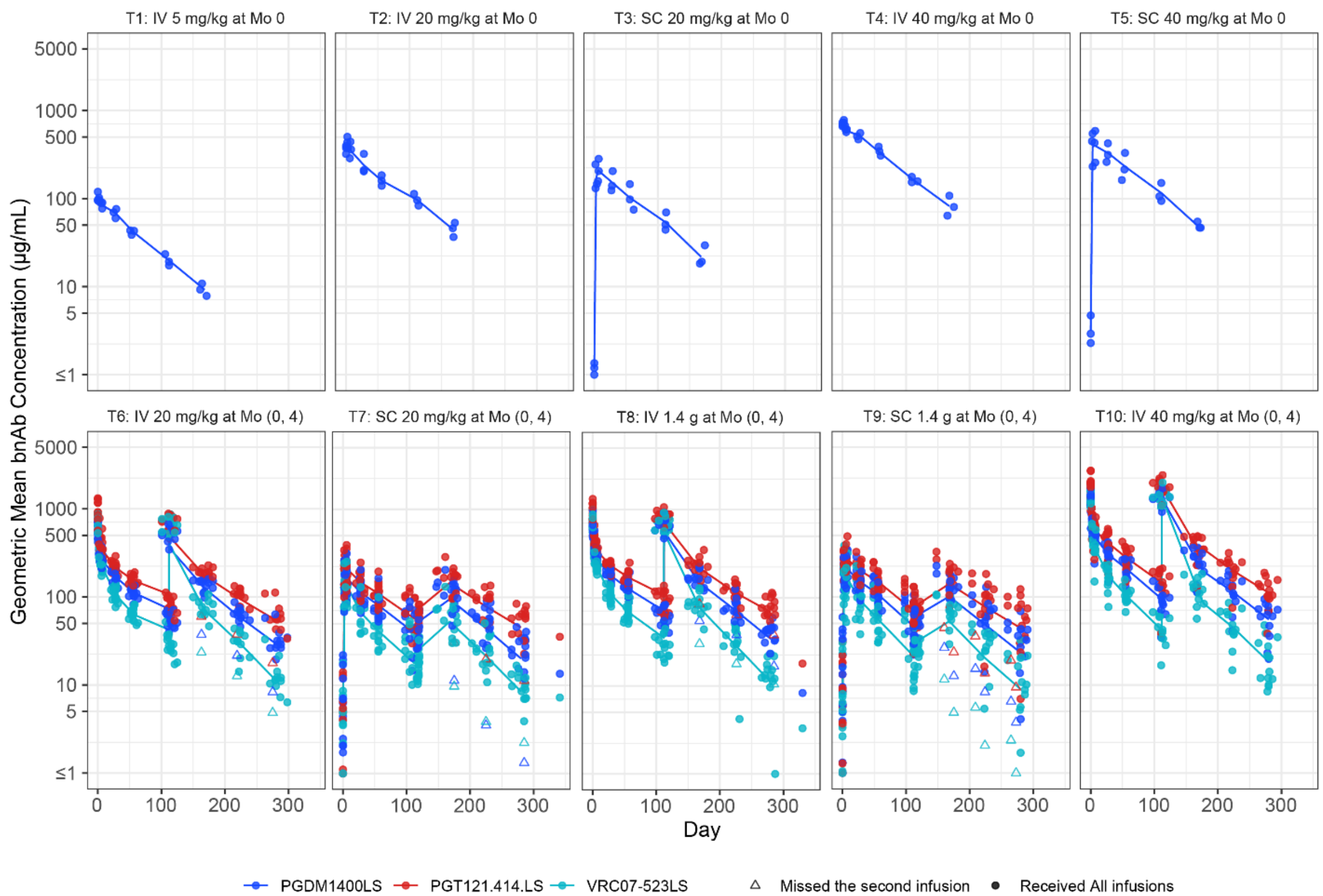

Supplemental Figure 1: Geometric mean mAb concentrations over time in Groups T1-T10. Dots indicate observed concentration levels; solid lines connect the geometric mean concentration levels across time-points for each mAb.

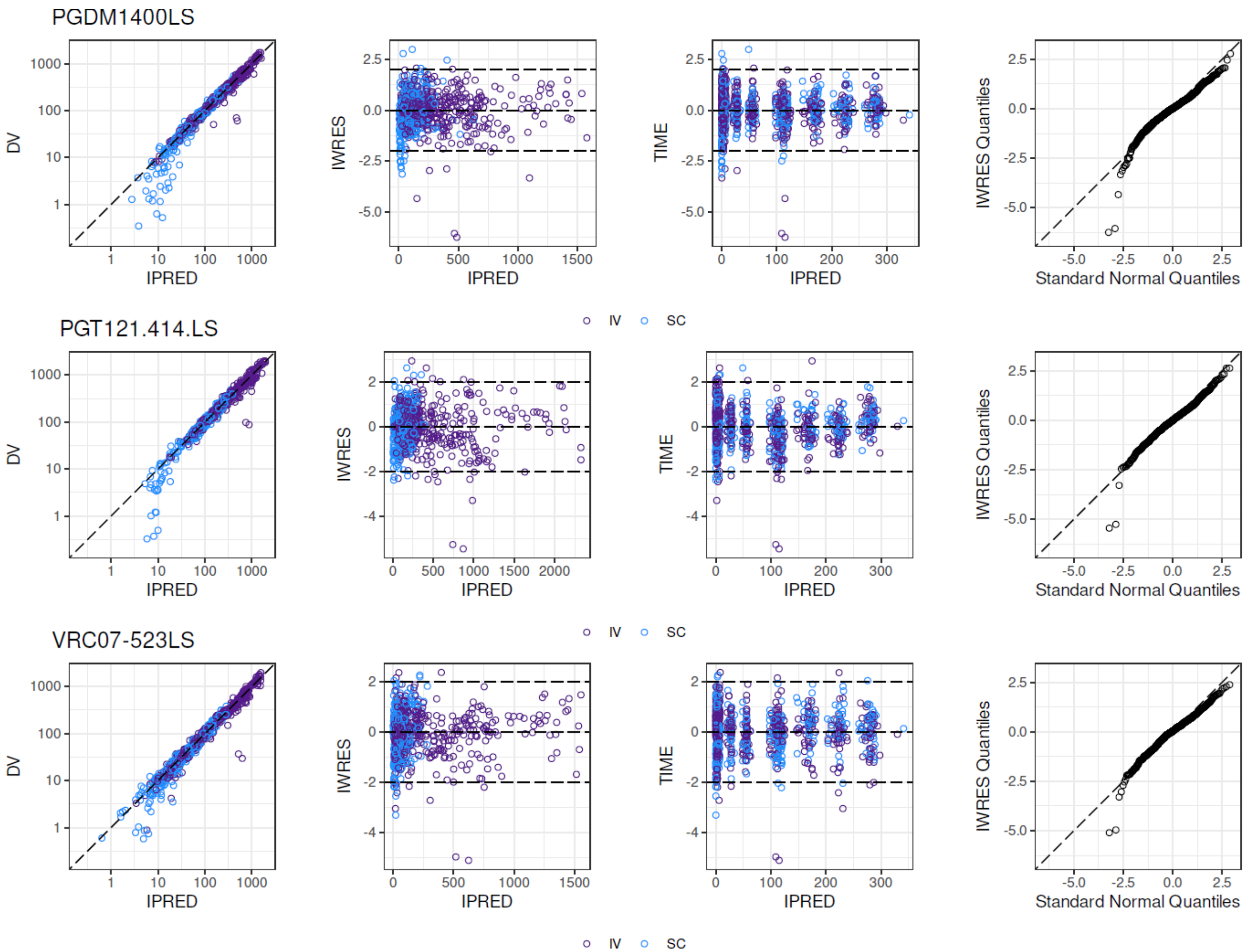

Supplemental Figure 2. Goodness of fit Diagnostic plots for the popPK models of PGDM1400LS (first row), PGT121.414.LS (second row) and VRC07-523LS (last row). The 1st column of the figures shows the observed concentration (DV) vs. the popPK model-predicted individual-level concentrations (IPRED); the 2nd column shows the individual weighted residuals (IWRES), calculated as the weighted difference between mode-predicted and observed concentrations vs. IPRED; the 3rd column shows IWRES vs. time since last infusion; and the column shows the Quantile-Quantile plot of the IWRES last.

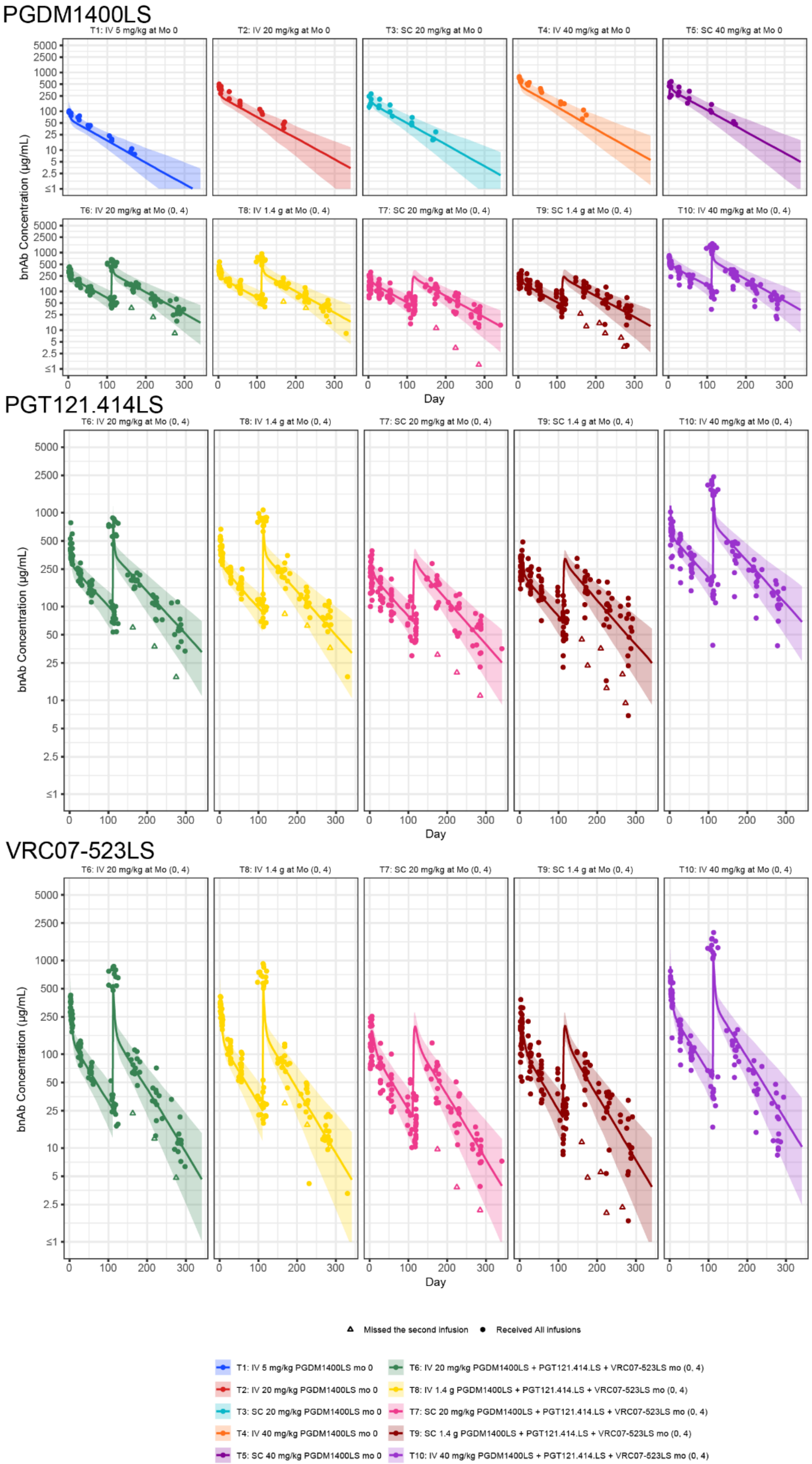
PGDM1400LS
T1: IV 5 mg/kg at Mo 0
T2: IV 20 mg/kg at Mo 0
T3: SC 20 mg/kg at Mo 0
T4: IV 40 mg/kg at Mo 0
T5: SC 40 mg/kg at Mo 0
T6: IV 20 mg/kg at Mo (0, 4)
T8: IV 1.4 g at Mo (0, 4)
T7: SC 20 mg/kg at Mo (0, 4)
T9: SC 1.4 g at Mo (0, 4)
T10: IV 40 mg/kg at Mo (0, 4)
bnAb Concentration (µg/mL)
Day
PGT121.414LS
VRC07-523LS
Missed the second infusion
Received All infusions
T1: IV 5 mg/kg PGDM1400LS mo 0
T2: IV 20 mg/kg PGDM1400LS mo 0
T3: SC 20 mg/kg PGDM1400LS mo 0
T4: IV 40 mg/kg PGDM1400LS mo 0
T5: SC 40 mg/kg PGDM1400LS mo 0
T6: IV 20 mg/kg PGDM1400LS + PGT121.414.LS + VRC07-523LS mo (0, 4)
T8: IV 1.4 g PGDM1400LS + PGT121.414.LS + VRC07-523LS mo (0, 4)
T7: SC 20 mg/kg PGDM1400LS + PGT121.414.LS + VRC07-523LS mo (0, 4)
T9: SC 1.4 g PGDM1400LS + PGT121.414.LS + VRC07-523LS mo (0, 4)
T10: IV 40 mg/kg PGDM1400LS + PGT121.414.LS + VRC07-523LS mo (0, 4)

Supplemental Figure 3. Observed mAb concentrations (dots) with the 90% prediction intervals (shaded areas) by treatment group (T1-T10) for PGDM1400LS, PGT121.414.LS and VRC07-523LS based on the final popPK models for each mAb. Open triangles indicate observations from participants who missed the 2nd infusion.

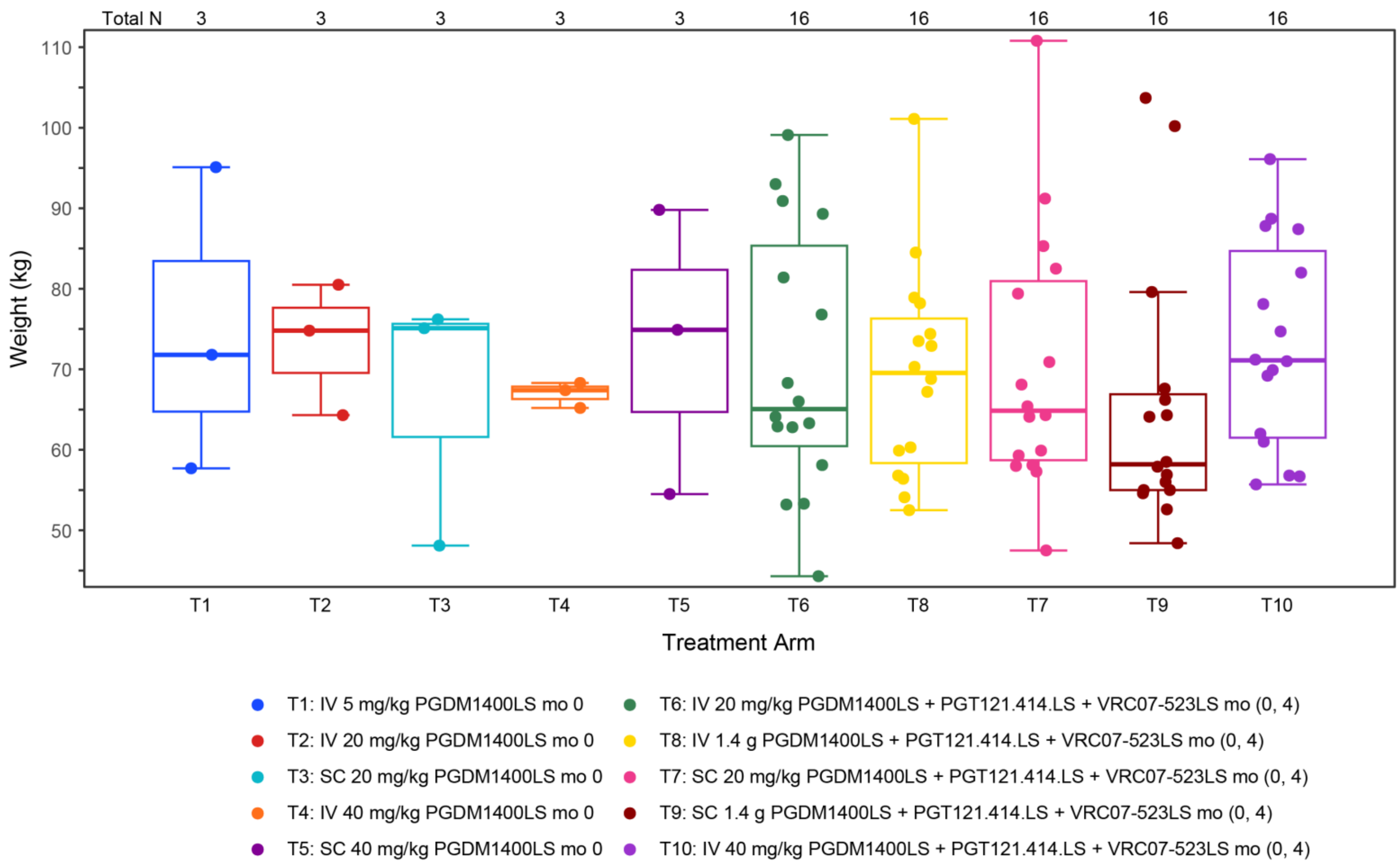

Supplemental Figure 4: Body weight distributions in Groups T1-T10.

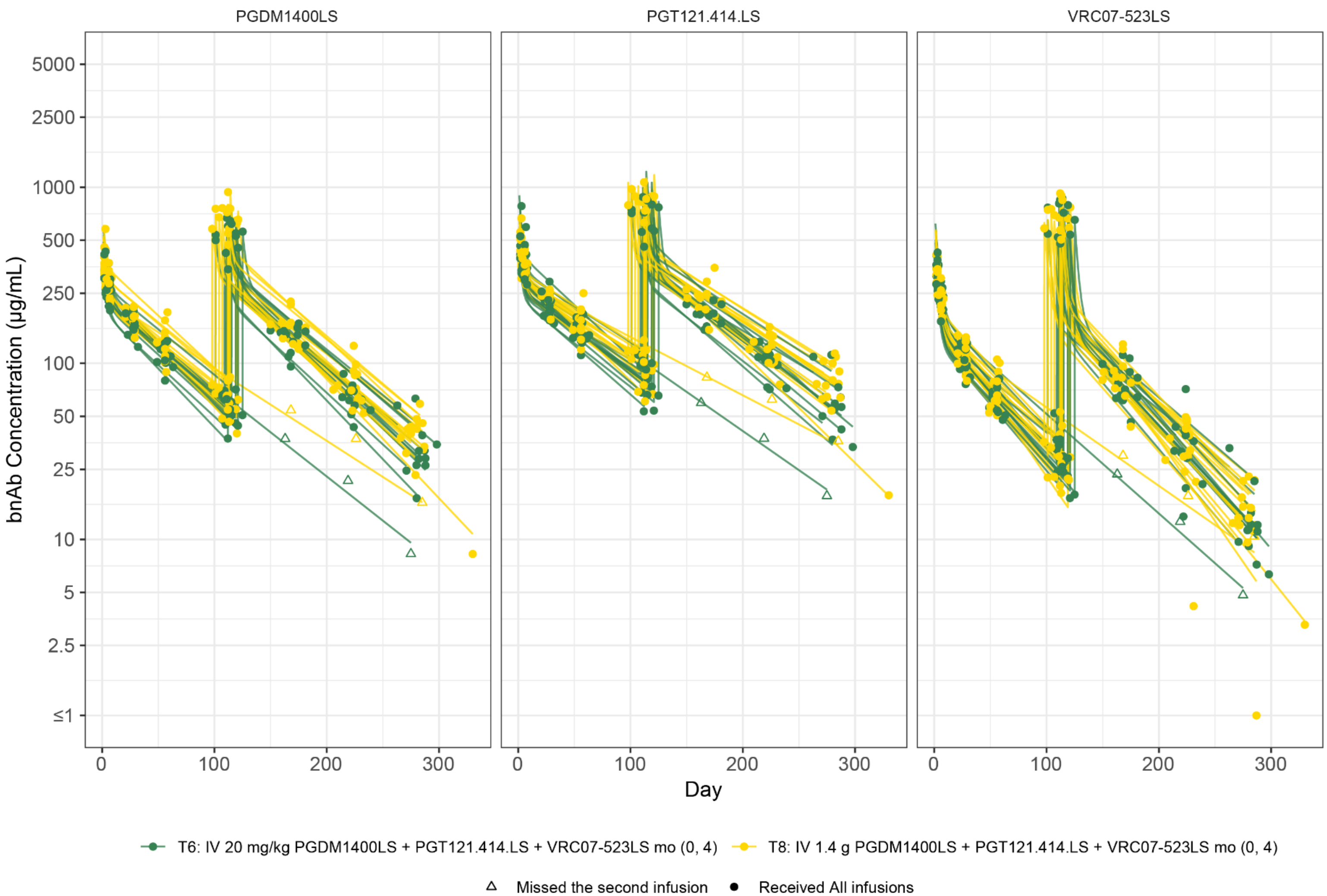

Supplemental Figure 5. PK of PGDM1400LS, PGT121.414LS, VRC07-523LS in the body-weight-based and fixed-dose IV administration regimens. Dots indicate observed mAb concentration levels; solid curves indicate popPK model-

fitted PK patterns for each individual. For PGDM1400LS, the popPK model without the fixed effect of single vs. combination administration was used.

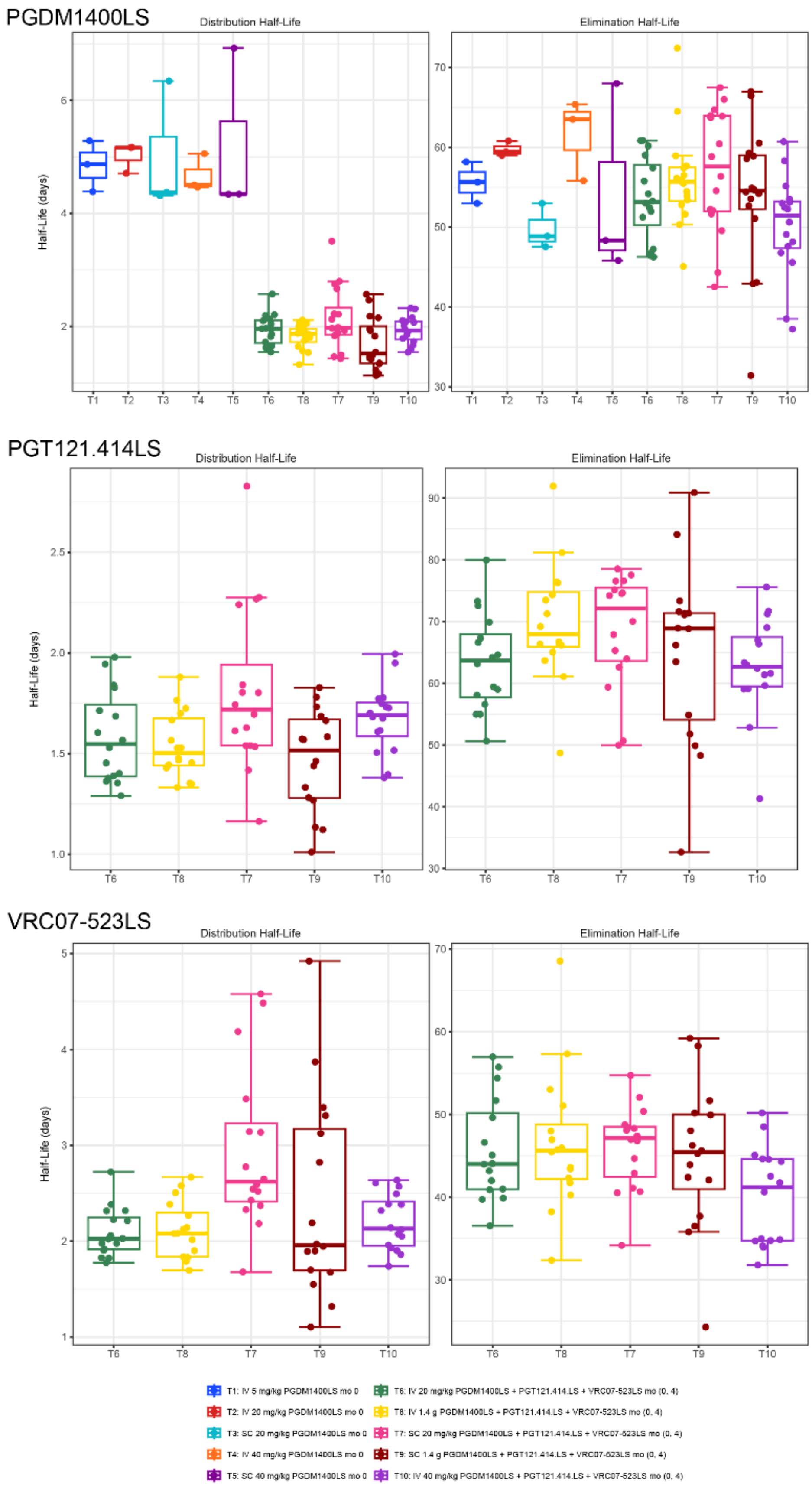

PGDM1400LS
Distribution Half-Life
Elimination Half-Life
Half-Life (days)
PGT121.414LS
Distribution Half-Life
Elimination Half-Life
Half-Life (days)
VRC07-523LS
Distribution Half-Life
Elimination Half-Life
Half-Life (days)
T1: IV 5 mg/kg PGDM1400LS mo 0
T2: IV 20 mg/kg PGDM1400LS mo 0
T3: SC 20 mg/kg PGDM1400LS mo 0
T4: IV 40 mg/kg PGDM1400LS mo 0
T5: SC 40 mg/kg PGDM1400LS mo 0
T6: IV 20 mg/kg PGDM1400LS + PGT121.414.LS + VRC07-523LS mo (0, 4)
T8: IV 1.4 g PGDM1400LS + PGT121.414.LS + VRC07-523LS mo (0, 4)
T7: SC 20 mg/kg PGDM1400LS + PGT121.414.LS + VRC07-523LS mo (0, 4)
T9: SC 1.4 g PGDM1400LS + PGT121.414.LS + VRC07-523LS mo (0, 4)
T10: IV 40 mg/kg PGDM1400LS + PGT121.414.LS + VRC07-523LS mo (0, 4)

Supplemental Figure 6: Distribution of estimated individual-level distribution and elimination half-lives of PGDM1400LS, PGT121.414.LS and VRC07-523LS from the final popPK model of each mAb.

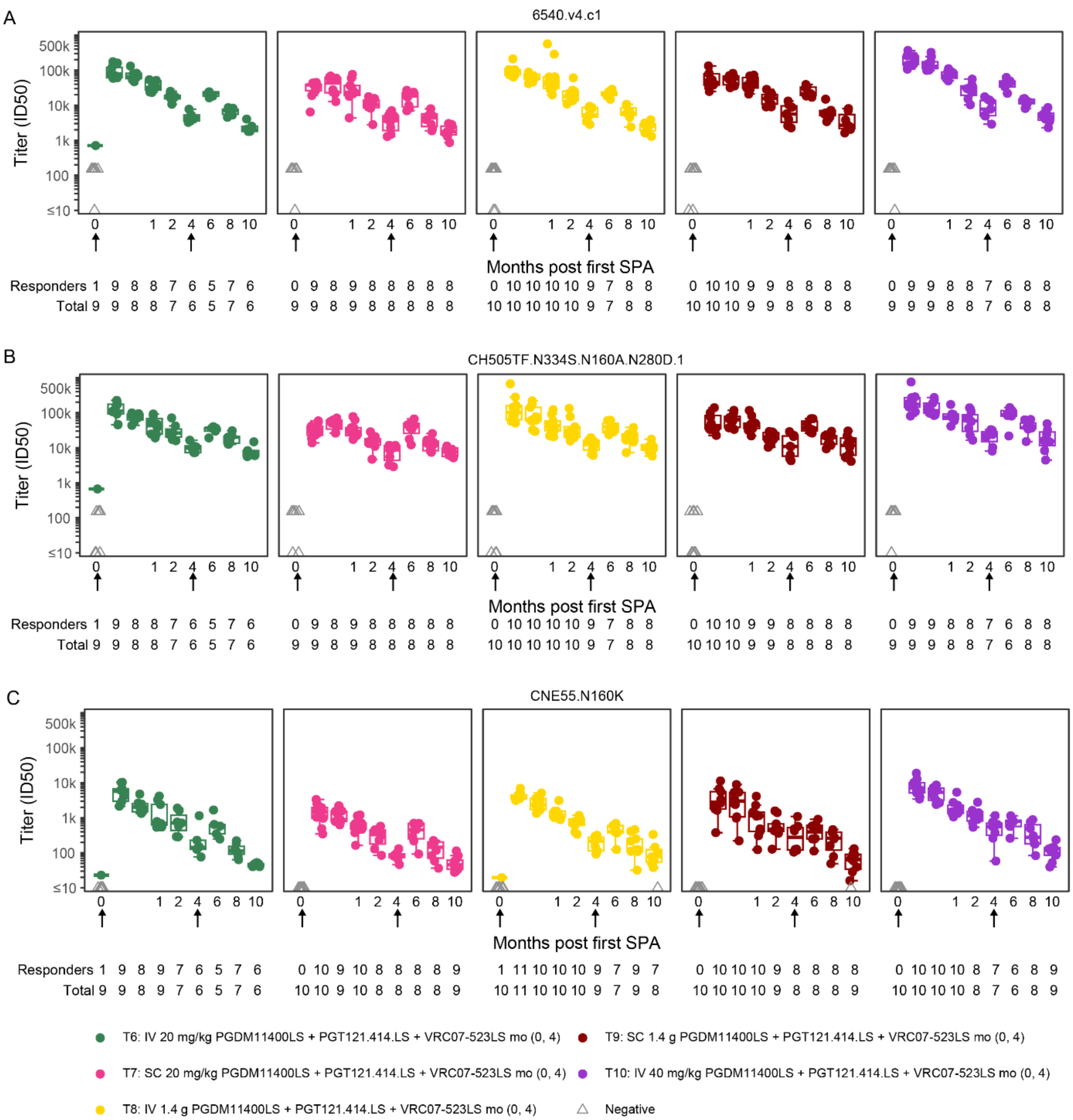

Supplemental Figure 7. Observed serum ID80 neutralization titers over time against PGDM1400LS-specific virus 6540.v4.c1 (Panel A), PGT121.414.LS-specific virus CH505TF.N334S.N160A.N280D.1 (Panel B), and VRC07-523LS-specific virus CNE55.N160K (Panel C). Arrows at month 0 and 4 depict times of antibody infusion. 6540.v4.c1 (A) is sensitive to neutralization by PGDM1400LS and resistant to PGT121.414.LS and VRC07-523LS. CH505TF.N334S.N160A.N280D (B) is sensitive to neutralization by PGT121.414.LS and resistant to PGDM1400LS and VRC07-523LS. CNE55.N160K (C) is sensitive to neutralization by VRC07-523LS and resistant to

PGT121.414.LS and PGDM1400LS. Days sampled between months 0 and 1 include days 3 and 6.

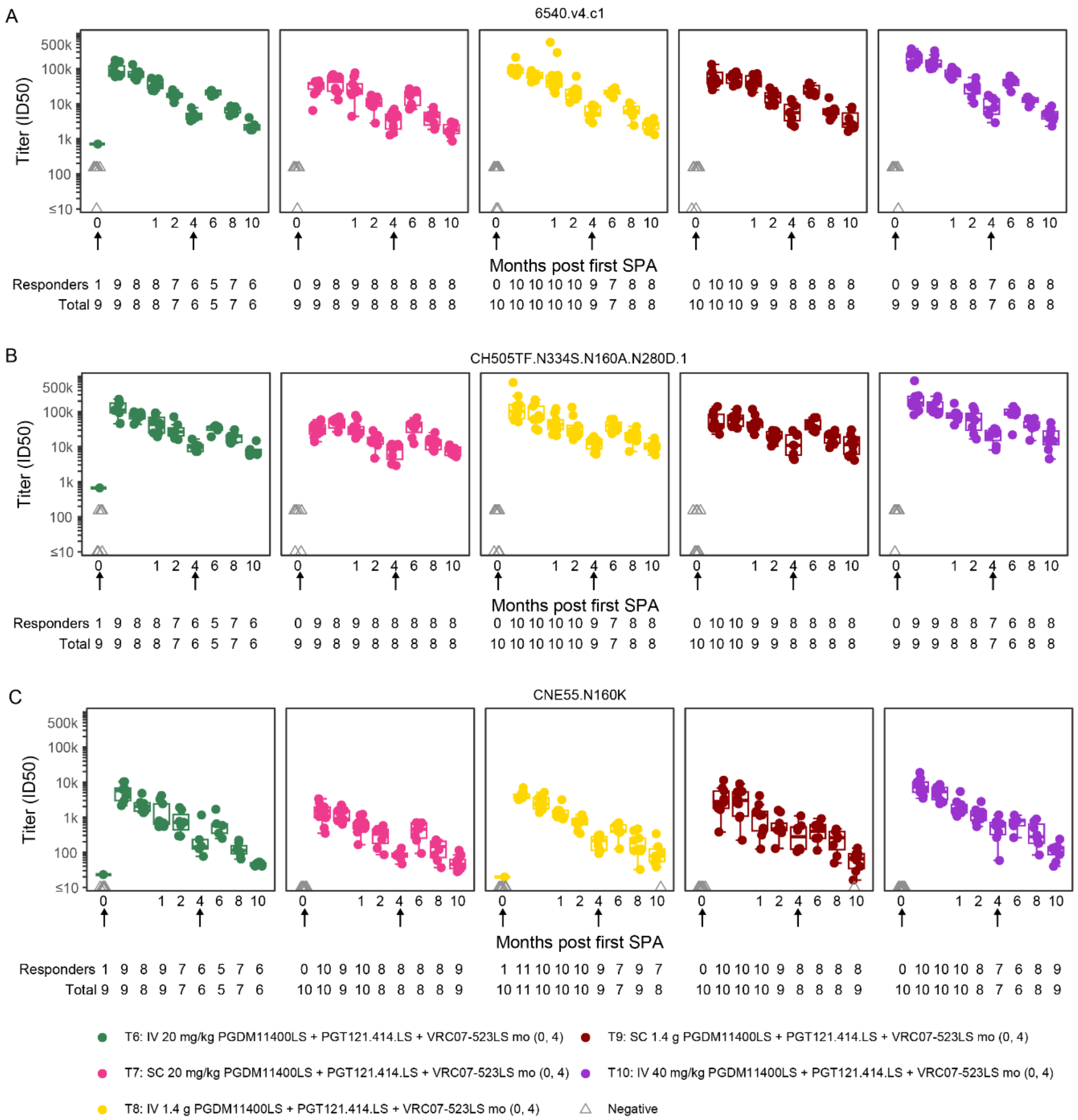

A
6540.v4.c1
Titer (ID50)
500k
100k
10k
1k
100
≤10
0 1 2 4 6 8 10
Months post first SPA
Responders 1 9 8 8 7 6 5 7 6 | 0 9 8 9 8 8 8 8 8 | 0 10 10 10 10 9 7 8 8 | 0 10 10 9 9 8 8 8 8 | 0 9 9 8 8 7 6 8 8
Total 9 9 8 8 7 6 5 7 6 | 9 9 8 9 8 8 8 8 8 | 10 10 10 10 10 9 7 8 8 | 10 10 10 9 9 8 8 8 8 | 9 9 9 8 8 7 6 8 8
B
CH505TF.N334S.N160A.N280D.1
Titer (ID50)
Months post first SPA
Responders 1 9 8 8 7 6 5 7 6 | 0 9 8 9 8 8 8 8 8 | 0 10 10 10 10 9 7 8 8 | 0 10 10 9 9 8 8 8 8 | 0 9 9 8 8 7 6 8 8
Total 9 9 8 8 7 6 5 7 6 | 9 9 8 9 8 8 8 8 8 | 10 10 10 10 10 9 7 8 8 | 10 10 10 9 9 8 8 8 8 | 9 9 9 8 8 7 6 8 8
C
CNE55.N160K
Titer (ID50)
Months post first SPA
Responders 1 9 8 9 7 6 5 7 6 | 0 10 9 10 8 8 8 8 9 | 1 11 10 10 10 9 7 9 7 | 0 10 10 10 9 8 8 8 8 | 0 10 10 10 8 7 6 8 9
Total 9 9 8 9 7 6 5 7 6 | 10 10 9 10 8 8 8 8 9 | 10 11 10 10 10 9 7 9 8 | 10 10 10 10 9 8 8 8 9 | 10 10 10 10 8 7 6 8 9
T6: IV 20 mg/kg PGDM11400LS + PGT121.414.LS + VRC07-523LS mo (0, 4)
T7: SC 20 mg/kg PGDM11400LS + PGT121.414.LS + VRC07-523LS mo (0, 4)
T8: IV 1.4 g PGDM11400LS + PGT121.414.LS + VRC07-523LS mo (0, 4)
T9: SC 1.4 g PGDM11400LS + PGT121.414.LS + VRC07-523LS mo (0, 4)
T10: IV 40 mg/kg PGDM11400LS + PGT121.414.LS + VRC07-523LS mo (0, 4)
Negative

Supplemental Figure 8: Observed ID50 neutralization tiers over time against PGDM1400LS-specific virus 6540.v4.c1 (Panel A), PGT121.414.LS-specific virus CH505TF.N334S.N160A.N280D.1 (Panel B), and VRC07-523LS-specific virus CNE55.N160K (Panel C). Arrows at months 0 and 4 depict times of antibody infusion. 6540.v4.c1 (A) is sensitive to neutralization by PGDM1400LS and resistant to PGT121.414.LS and VRC07-523LS. CH505TF.N334S.N160A.N280D (B) is sensitive to neutralization by PGT121.414.LS and resistant to PGDM1400LS and VRC07-523LS. CNE55.N160K (C) is sensitive to neutralization by VRC07-523LS and resistant to PGT121.414.LS and PGDM1400LS. Days sampled between months 0 and 1 include days 3 and 6.

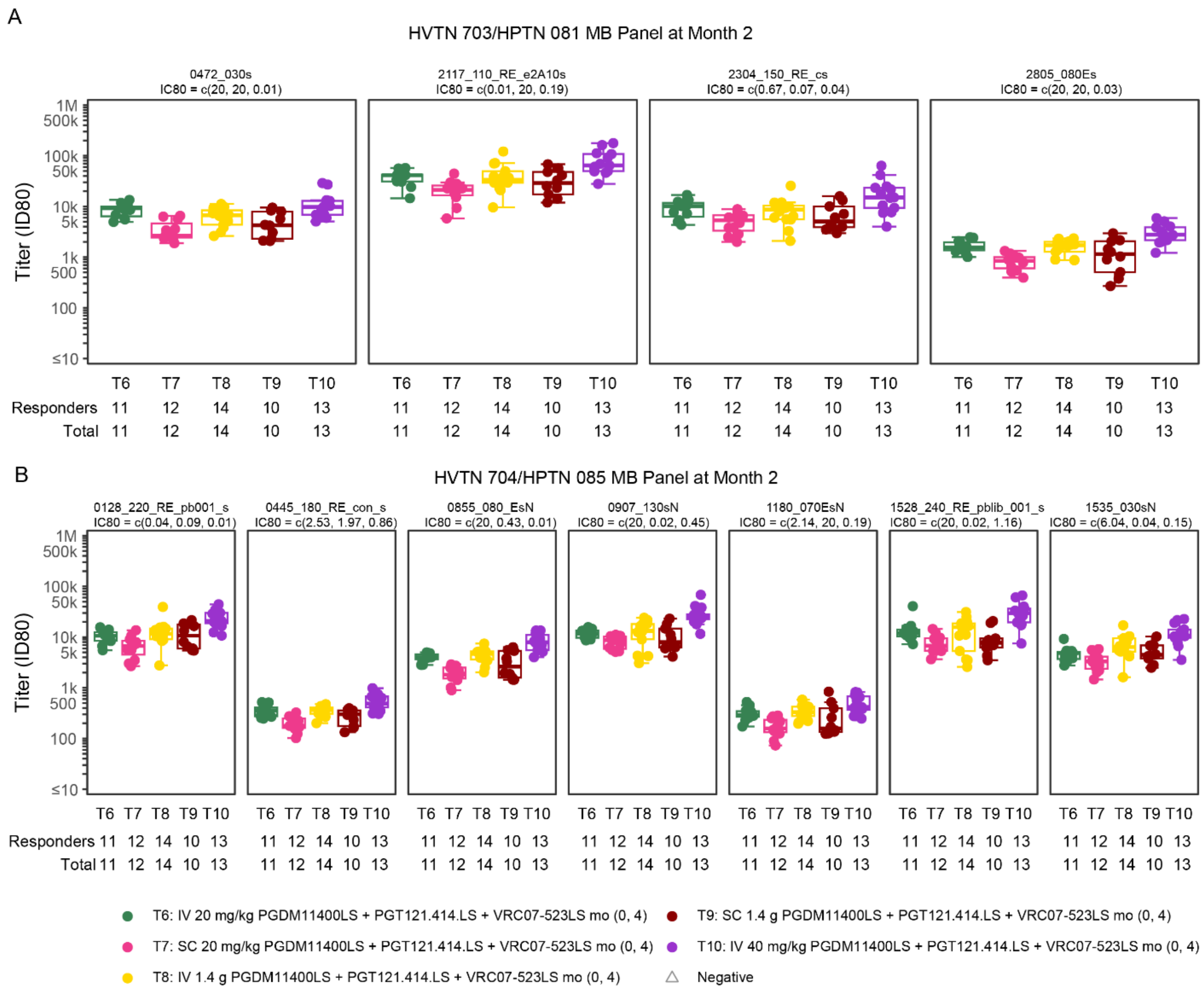

Supplemental Figure 9. Observed serum ID80 neutralization tiers at 2 months post first study product administration against separate magnitude-breadth panels of contemporaneous clade C (A) and clade B (B) viruses obtained in the AMP trials. IC80 values (μg/ml) for each virus for each of the three mAbs is listed below the virus name.

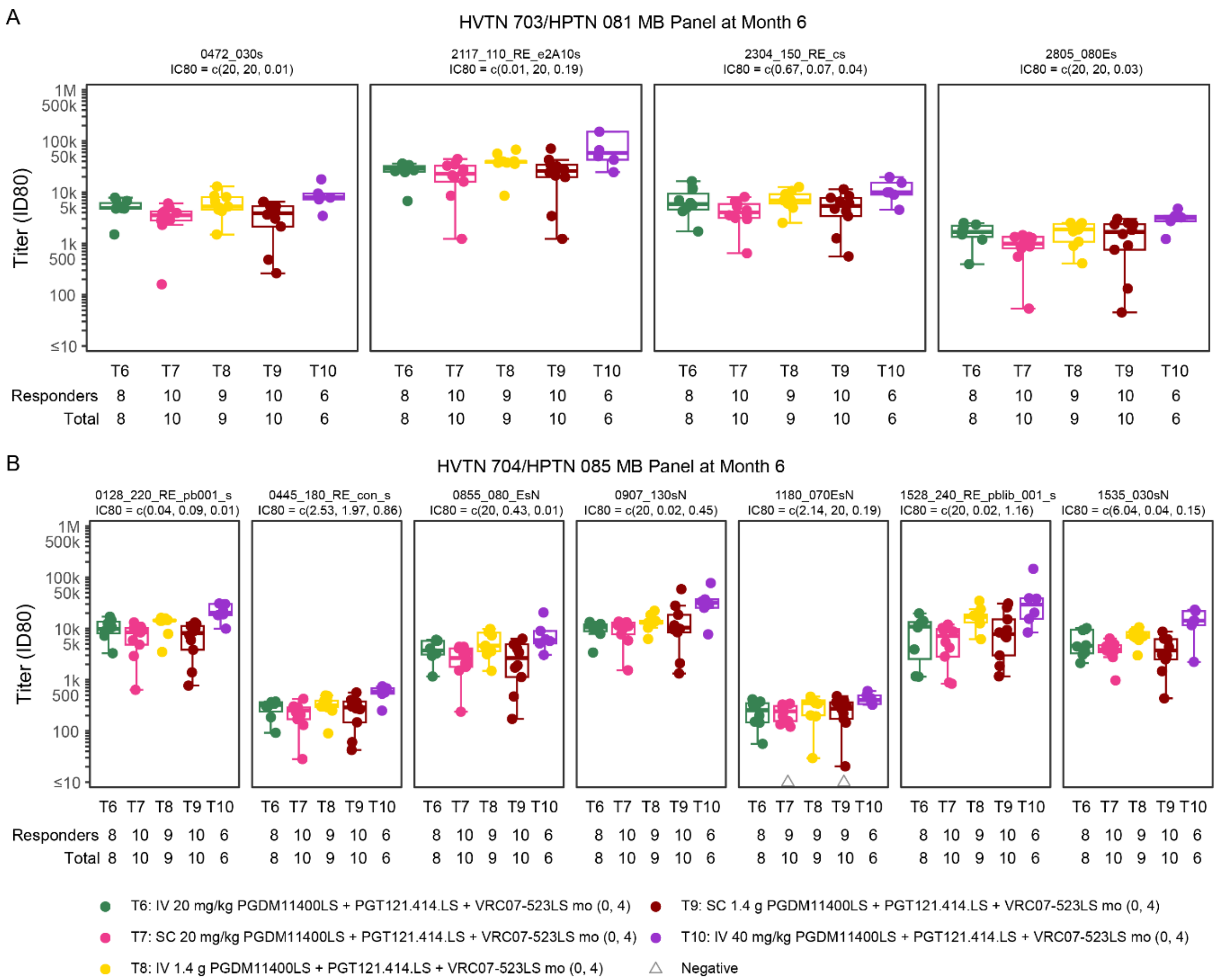

Supplemental Figure 10: Observed ID80 neutralization tiers at 2 months post the 2nd study product administration (month 6) against a panel of contemporaneous clade C viruses (Panel A) and clade B viruses (Panel B) obtained in the AMP trials. IC80 values for each virus for each of the three mAbs is listed below the virus name.

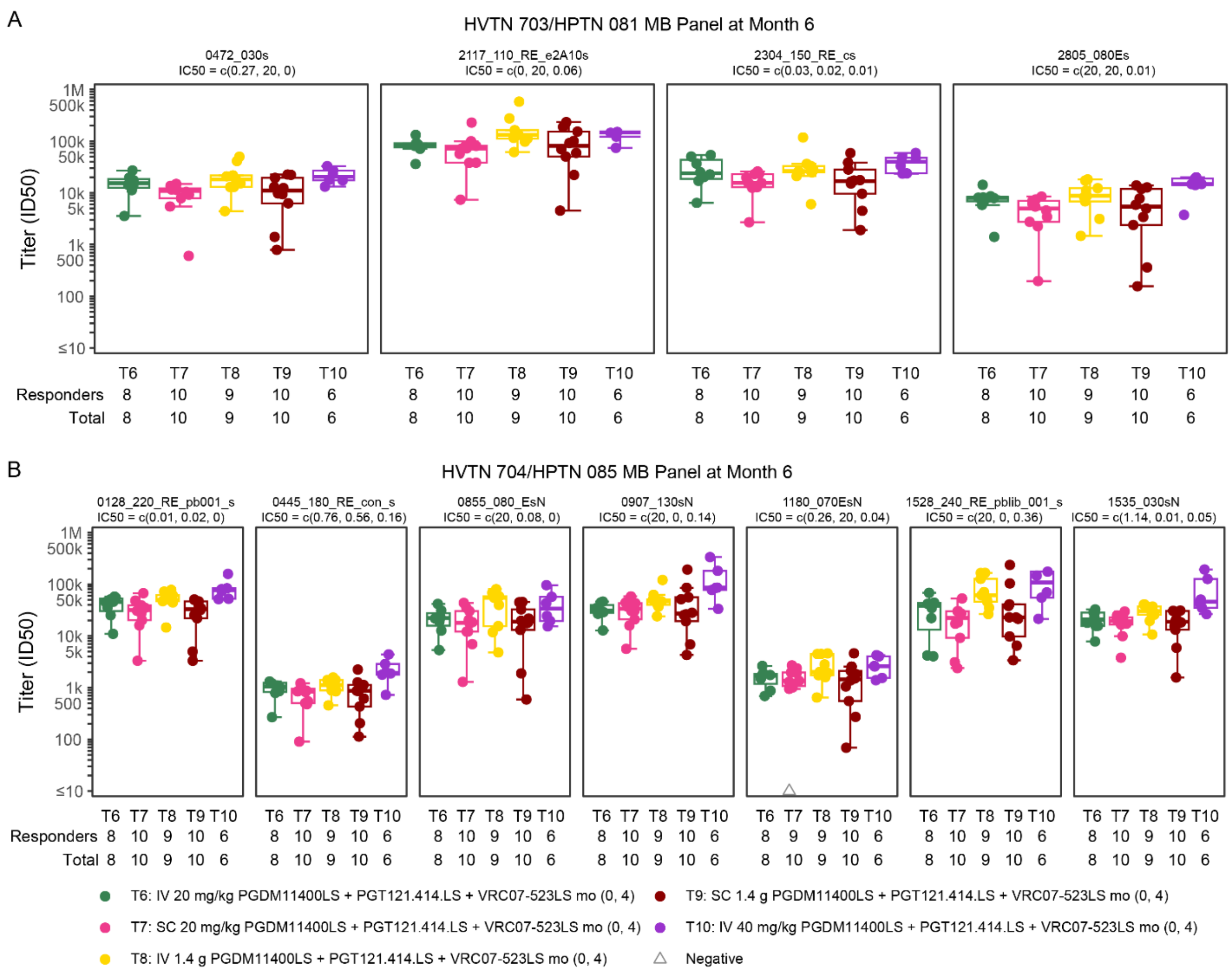

Supplemental Figure 11: Observed ID50 neutralization tiers at 2 months post the 2nd study product administration (month 6) against a panel of contemporaneous clade C viruses (Panel A) and clade B viruses (Panel B) obtained in the AMP trials. IC50 values for each virus for each of the three mAbs is listed below the virus name.

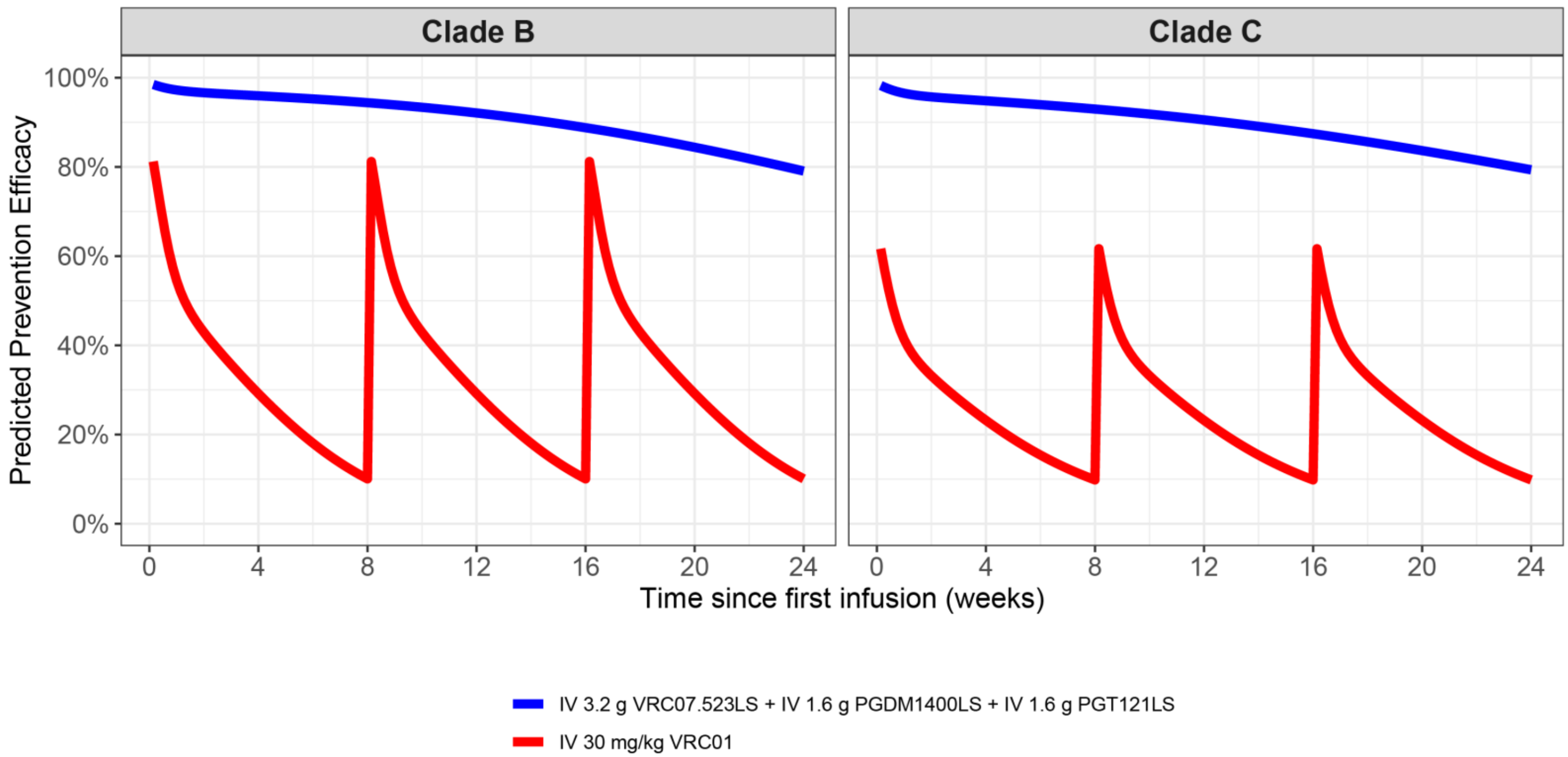

Supplemental Figure 12. Predicted prevention efficacy of the triple-mAb combination VRC07-523LS + PGDM1400LS + PGT121.414.LS administered intravenously at 3.2g + 1.6g +1.6g (blue) relative to that of eight-weekly VRC01 administered intravenously at 30 mg/kg (red), against clade B and clade C viruses. The following three steps were followed to predict the prevention efficacy. Step 1: Given the pharmacodynamic relationship between PT80 and prevention efficacy, and the estimated daily-grid combination PT80 titer over time against each circulating virus for a given individual, the predicted prevention efficacy against each virus is calculated for the individual via plugging in the estimated PT80 titer values to the pharmacodynamics model; Step 2: Given the predicted prevention efficacy over time against each virus, and the distribution of the circulating viruses, predicted prevention efficacy against all possible circulating strains assuming equal exposure over time is obtained for the given individual in an AMP-like trial; Step 3: the same Steps 1 and 2 are repeated for each individual, and the estimated population level PE is obtained.

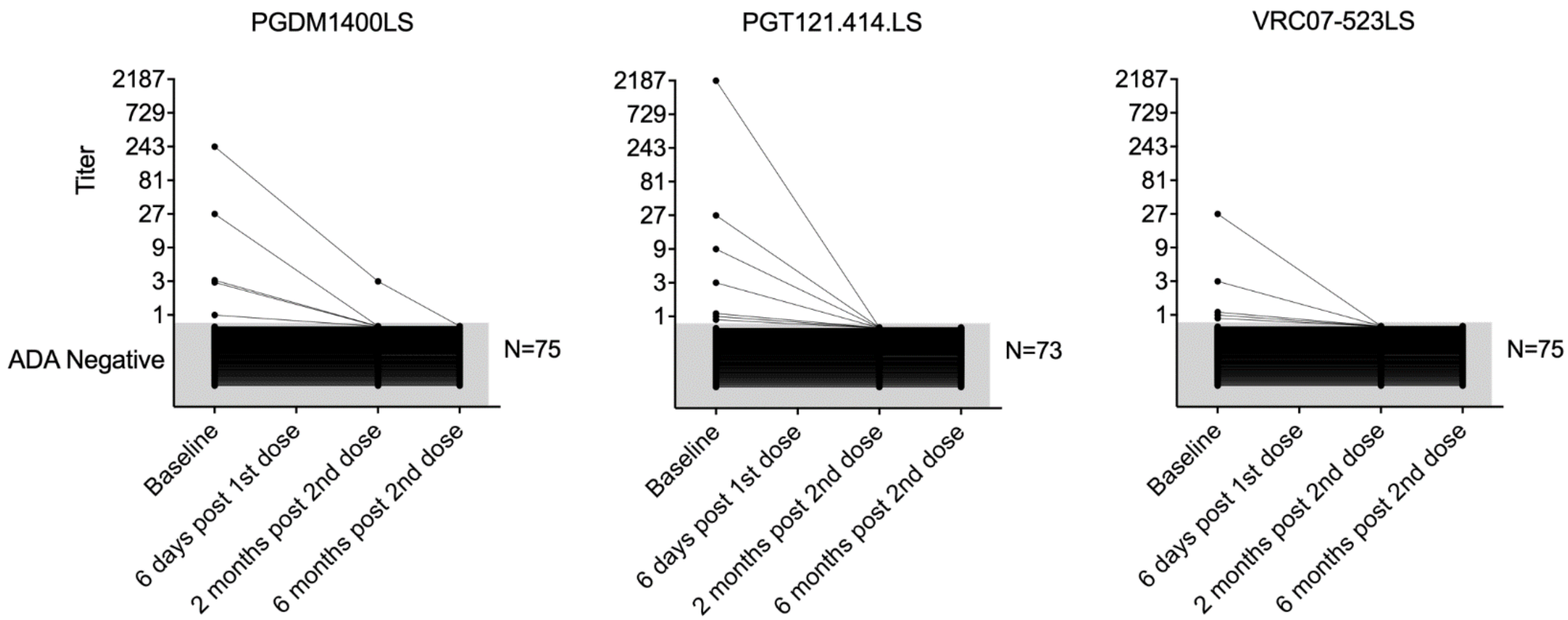

Supplemental Figure 13: ADA Titer. Number of subjects with no ADA indicated as N=#.

PROTOCOL

HVTN 140/HPTN 101

A phase 1 dose-escalation clinical trial to evaluate the safety, tolerability, and pharmacokinetics of PGDM1400LS alone and in combination with VRC07-523LS and PGT121.414.LS in healthy, HIV-uninfected adult participants

PROTOCOL

HVTN 140/HPTN 101

A phase 1 dose-escalation clinical trial to evaluate the safety, tolerability, and pharmacokinetics of PGDM1400LS alone and in combination with VRC07-523LS and PGT121.414.LS in healthy, HIV-uninfected adult participants

DAIDS DOCUMENT ID 38723

IND 154188 HELD BY DAIDS

CLINICAL TRIAL SPONSORED BY

Division of AIDS (DAIDS)
National Institute of Allergy and Infectious Diseases (NIAID)
National Institutes of Health (NIH)
Department of Health and Human Services (DHHS)
Bethesda, Maryland, USA

STUDY PRODUCT(S) PROVIDED BY

DAIDS, NIAID, NIH, DHHS
(Bethesda, Maryland, USA)

Dale and Betty Bumpers Vaccine Research Center (VRC), NIAID, NIH, DHHS
(Bethesda, Maryland, USA)

June 23, 2021
Final
HVTN 140/HPTN 101
Version 1.0

FOR REVIEW ONLY

Contents

1 Overview 5
1.1 Protocol Team 9
2 Ethical considerations 11
3 IRB/EC review considerations 13
3.1 Minimized risks to participants 13
3.2 Reasonable risk/benefit balance 13
3.3 Equitable participant selection 14
3.4 Appropriate informed consent 14
3.5 Adequate safety monitoring 14
3.6 Protect privacy/confidentiality 15
4 Background 16
4.1 Rationale for trial concept 16
4.2 PGDM1400LS 18
4.3 PGT121.414.LS 19
4.4 VRC07-523LS (VRC-HIVMAB075-00-AB) 20
4.5 Trial design rationale 20
4.6 Plans for future product development and testing 26
4.7 Preclinical studies 26
4.8 Clinical trials of PGT121, PGT121.141LS, PGDM1400, VRC07-523LS alone and in combinations 36
4.9 Potential risks of study products and administration 48
5 Objectives and endpoints 51
5.1 Primary objectives and endpoints 51
5.2 Secondary objectives and endpoints 52
5.3 Exploratory objectives 53
6 Statistical considerations 54
6.1 Accrual and sample size calculations 54
6.2 Randomization 59
6.3 Blinding 60
6.4 Statistical analyses 60
7 Selection and withdrawal of participants 66
7.1 Inclusion criteria 66
7.2 Exclusion criteria 69
7.3 Participant departure from study product administration schedule or withdrawal (Part B) 72
8 Study product 75
8.1 Study product regimen 75
8.2 Study product formulation 76
8.3 Preparation of study products 76
8.4 Administration 85
8.5 Acquisition of study products 87
8.6 Pharmacy records 87
8.7 Final disposition of study products 88

FOR REVIEW ONLY

9 Clinical procedures 89
9.1 Informed consent 89
9.2 Pre-enrollment procedures 91
9.3 Enrollment and study product administration visits....93
9.4 Follow-up visits....95
9.5 HIV counseling and testing 96
9.6 Contraception status 97
9.7 Urine testing 98
9.8 Assessments of Solicited AEs 98
9.9 Visit windows and missed visits 100
9.10 Early termination visit....100
9.11 Pregnancy 100
9.12 HIV infection during the study....101

10 Laboratory....102
10.1 CRS laboratory procedures 102
10.2 Total blood volume 102
10.3 PGDM1400LS, VRC07-523LS, and PGT121.414.LS concentrations 102
10.4 Neutralizing antibody assay 103
10.5 ADA detection assays 103
10.6 ADA functional assay 103
10.7 Antibody reaction assays....103
10.8 HVTN Laboratory Center assay portfolio....103
10.9 Exploratory studies....104
10.10 Specimen storage and other use of specimens 104
10.11 Biohazard containment....104

11 Safety monitoring and safety review 106
11.1 Safety monitoring and oversight 106
11.2 Safety reporting 107
11.3 Safety reviews 110
11.4 Safety pause and prompt PSRT AE review....113
11.5 Review of cumulative safety data 115
11.6 Study termination 115

12 Protocol conduct 117
12.1 Social impacts 118
12.2 Emergency communication with study participants 118

13 Version history....119

14 Document references (other than literature citations)....120

15 Acronyms and abbreviations....123

16 Literature cited....126

Appendix A Sample informed consent form for Part A 132

Appendix B Sample informed consent form for Part B 152

Appendix C Approved birth control methods for persons assigned female sex at birth (for sample informed consent form)....173

FOR REVIEW ONLY

Appendix D Sample consent form for use of samples and information in other studies 175
Appendix E Table of procedures (for sample informed consent form) 180
Appendix F Laboratory procedures for Part A 182
Appendix G Laboratory procedures for Part B 184
Appendix H Procedures at HVTN CRS for Part A 186
Appendix I Procedures at HVTN CRS for Part B 188
Appendix J HVTN low risk guidelines for the US 190
Appendix K HVTN low risk guidelines for Southern Africa 193
Appendix L Visit windows 194
Appendix M Protocol Signature Page 195

FOR REVIEW ONLY

1 Overview

Title

A phase 1 dose-escalation clinical trial to evaluate the safety, tolerability, and pharmacokinetics of PGDM1400LS alone and in combination with VRC07-523LS and PGT121.414.LS in healthy, HIV-uninfected adult participants

Primary objective(s)

- To evaluate the safety and tolerability of PGDM1400LS when administered via intravenous (IV) or subcutaneous (SC) routes (Part A) and of PGDM1400LS + VRC07-523LS + PGT121.414.LS when administered in sequence IV or SC (Part B)

- To evaluate the serum concentrations and pharmacokinetics of PGDM1400LS after a single administration (Part A) and of PGDM1400LS + VRC07-523LS + PGT121.414.LS after each three-mAb administration (Part B)

- To evaluate the individual mAb-specific serum neutralizing activity after single product administration of PGDM1400LS (Part A) and after each three-mAb administration of PGDM1400LS, VRC07-523LS and PGT121.414.LS (Part B)

Study products

- **PGDM1400LS**: a human mAb that targets the HIV-1 V2 glycan, centered on N160. It is a derivative of PGDM1400 that was engineered to improve in vivo elimination half-life. It has been developed by the Vaccine Research Program (VRP) of the National Institute of Allergy and Infectious Diseases (NIAID). PGDM1400LS was manufactured under current Good Manufacturing Practice (cGMP) standards at Just-Evotec (Seattle, Washington) under contract to DAIDS's Vaccine Translational Research Branch (VTRB). The drug product was filled and released at the VRC Pilot Plant, operated under contract by Vaccine Clinical Materials Program (VCMP), Leidos Biomedical Research, Inc., Frederick, MD. The drug product is provided at 100 mg/mL as 10 mL glass vials with a 4.75 mL fill volume.

- **PGT121. 414.LS** is a human mAb that targets the HIV-1 V3 glycan, centered on N332. It is a derivative of PGT121 that was engineered for improved manufacturing, stability and in vivo elimination half-life by Just Biotherapeutics in collaboration with Dan Barouch and Collaboration for AIDS Vaccine Discovery (CAVD) investigators. The drug substance was manufactured under cGMP standards at Just Biotherapeutics under contract to DAIDS's VTRB. The drug product was filled and released by the VRC and VCMP, Leidos Biomedical Research, Inc., Frederick, MD. Product is provided at 100 mg/mL as 10 mL glass vials with a 4.75 mL fill volume.

FOR REVIEW ONLY

- **VRC-HIVMAB075-00-AB (VRC07-523LS)** is a human mAb that targets the HIV-1 CD4 binding site. It was developed by the VRC/NIAID/NIH and manufactured under cGMP standards at the VRC Pilot Plant operated under contract by the VCMP, Leidos Biomedical Research, Inc., Frederick, MD. Product is provided at 100 ± 10 mg/mL as 10 mL glass vials with a 6.25 ± 0.1 mL fill volume and 3 mL glass vials with a 2.25 mL ± 0.1 mL fill volume.

Table 1-1 Schema

Study arm	N*	Dose	Route	Month 0	Month 4
**Part A**					
Group 1	3	5 mg/kg	IV	PGDM1400LS	-
Group 2[1]	3	20 mg/kg	IV	PGDM1400LS	-
Group 3[1]	3	20 mg/kg	SC	PGDM1400LS	-
Group 4[2]	3	40 mg/kg	IV	PGDM1400LS	-
Group 5[2]	3	40 mg/kg	SC	PGDM1400LS	-
**Part B****					
Group 6[3]	16	20 mg/kg + 20 mg/kg + 20 mg/kg	IV	PGDM1400LS + VRC07-523LS + PGT121.414.LS	PGDM1400LS + VRC07-523LS + PGT121.414.LS
Group 7[3]	16	20 mg/kg + 20 mg/kg + 20 mg/kg	SC	PGDM1400LS + VRC07-523LS + PGT121.414.LS	PGDM1400LS + VRC07-523LS + PGT121.414.LS
Group 8[3]	16	1.4 g + 1.4 g + 1.4 g	IV	PGDM1400LS + VRC07-523LS + PGT121.414.LS	PGDM1400LS + VRC07-523LS + PGT121.414.LS
Group 9[3]	16	1.4 g + 1.4 g + 1.4 g	SC	PGDM1400LS + VRC07-523LS + PGT121.414.LS	PGDM1400LS + VRC07-523LS + PGT121.414.LS
Group 10[4]	16	40 mg/kg + 40 mg/kg + 40 mg/kg	IV	PGDM1400LS + VRC07-523LS + PGT121.414.LS	PGDM1400LS + VRC07-523LS + PGT121.414.LS
**Total**	**95**				

IV = intravenous infusion; SC = subcutaneous infusion; *additional participants may be enrolled to ensure the availability of safety data from at least 3 participants in each group;** antibodies will be administered sequentially as 3 separate infusions; + sign = "and".

[1]Opening enrollment in Groups 2 & 3 follows review of safety data for participants in Group 1. Details are described in Section 11.3.1.

[2]Opening enrollment in Group 4 & 5 follows review of safety data for participants in Groups 1- 3.

[3]Opening enrollment in Groups 6, 7, 8, and 9 follows review of safety data for participants in Part A.

[4]Opening enrollment in Group 10 follows review of safety data for participants from Groups 1-9.

FOR REVIEW ONLY

Participants

About 95 healthy, HIV-1–uninfected volunteers aged 18 through 50 years

Design

Multicenter, randomized, open-label study

Duration per participant

6 months per participant in Part A and 10 months per participant in Part B of scheduled clinic visits

Estimated total study duration

14 months (includes enrollment, planned safety holds, and follow-up)

Investigational New Drug (IND) sponsor

DAIDS, NIAID, NIH, DHHS (Bethesda, Maryland, USA)

Study product providers

- **PGDM1400LS**: DAIDS, NIAID, NIH, DHHS (Bethesda, Maryland, USA)
- **PGT121.414.LS**: DAIDS, NIAID, NIH, DHHS (Bethesda, Maryland, USA)
- **VRC07-523LS**: Dale and Betty Bumpers Vaccine Research Center (VRC), NIAID, NIH, DHHS (Bethesda, Maryland, USA)

HVTN Leadership and Operations Center (LOC)

HIV Vaccine Trials Network (HVTN) Leadership Group/Core Operations Center, Fred Hutchinson Cancer Research Center (Fred Hutch) (Seattle, Washington, USA)

HVTN Statistical and data management center (SDMC)

Statistical Center for HIV/AIDS Research and Prevention (SCHARP), Fred Hutch (Seattle, Washington, USA)

HVTN Laboratory Center (LC)

HIV diagnostic laboratory

University of Washington Virology Specialty Laboratory (UW-VSL) (Seattle, Washington, USA)

FOR REVIEW ONLY

Endpoint assay laboratories

- Duke University Medical Center (Durham, North Carolina, USA)
- South Africa Immunology Laboratory and National Institute for Communicable Diseases (Johannesburg, South Africa)

Study sites

HVTN and HPTN Clinical Research Sites (CRSs) in the US and sub-Saharan Africa to be specified in the Site Announcement Memo

Safety monitoring

HVTN 140/HPTN 101 Protocol Safety Review Team (PSRT); HVTN Safety Monitoring Board (SMB)

FOR REVIEW ONLY

1.1 Protocol Team

Protocol leadership

HVTN Chairs	Colleen Kelley<br>Emory University<br>404-712-1435<br>colleen.kelley@emory.edu<br><br>Sharana Mahomed<br>Centre for the AIDS Programme of Research in South Africa (CAPRISA)<br>+27 31 655 0610<br>Sharana.Mahomed@caprisa.org	*HPTN Chairs*	Marc Siegel<br>George Washington Medical Faculty Associates<br>202-741-2165<br>msiegel@mfa.gwu.edu
HVTN Protocol Team leaders	Shelly Karuna<br>HVTN LOC, Fred Hutch<br>206-667-4355<br>mkaruna@fredhutch.org<br><br>Simba Takuva<br>HVTN LOC, Fred Hutch<br>+27 11 989 9968<br>stakuva@hcrisa.org.za	*HPTN Protocol Team leader*	Theresa Gamble<br>HPTN LOC, FHI 360<br>919-544-7040 x11350<br>tgamble@fhi360.org
HVTN Laboratory lead	David Montefiori<br>HVTN Laboratory Center<br>919-684-5278<br>david.montefiori@duke.edu	*HPTN Laboratory lead*	Estelle Piwowar-Manning<br>HPTN Laboratory Center, Johns Hopkins University<br>410-614-6736<br>epiwowa@jhmi.edu
DAIDS Medical officer	Peggy Yacovone<br>DAIDS, NIAID<br>240-292-0826<br>margaret.yacovone@nih.gov	*DAIDS Medical officer*	Wairimu Chege<br>DAIDS, NIAID<br>240-292-4786<br>wairimu.chege@nih.gov
HVTN Statistician	Chenchen Yu<br>HVTN SDMC, Fred Hutch<br>206-667-5658<br>cyu@scharp.org	*HPTN Statistician*	Fei Gao<br>HPTN, LOC, FHI 360<br>fgao@fredhutch.org

FOR REVIEW ONLY

Other contributors to the original protocol

HVTN Medical monitor	Shelly Karuna<br>HVTN LOC, Fred Hutch	*DAIDS protocol pharmacist*	Shawn Chiambah<br>Lynette Purdue<br>DAIDS, NIAID
HVTN Regional medical liaison	Simba Takuva<br>HVTN LOC, Fred Hutch	*VRC Consultant*	Lucio Gama<br>Dale and Betty Bumpers Vaccine Research Center
HVTN Clinical safety specialist	Maija Anderson<br>HVTN LOC, Fred Hutch	*HPTN Pharmacologist*	Julie Dumond<br>UNC Chapel Hill
HVTN Clinical trials manager	India Tindale<br>HVTN LOC, Fred Hutch	*HPTN Senior Clinical research manager*	Bonnie Dye<br>HPTN LOC, FHI360
HVTN Laboratory Center representative	Jen Hanke<br>HVTN Laboratory Center, Fred Hutch	*HPTN Laboratory Program representative*	Amber Moser<br>HPTN Laboratory Center
HVTN Statistician consultant	Yunda Huang<br>HVTN SDMC, Fred Hutch	*Statistical research associate*	Chenchen Yu<br>HVTN SDMC, Fred Hutch
SDMC Associate director of lab science	April Randhawa<br>SCHARP, Fred Hutch	*Lead clinical data manager*	Nate Holm<br>SCHARP, Fred Hutch
HVTN Regulatory affairs associate	Laurie Rinn<br>HVTN LOC, Fred Hutch	*DAIDS product lead*	Michael Pensiero<br>DAIDS, NIAID
Community Advisory Board (CAB) members	Lenka Tswenyana<br>PHRU Soweto/Kliptown CAB<br>Melissa Turner<br>George Washington University CAB	*Clinic coordinators*	Geoffrey Kamau<br>The Hope Clinic<br>Emory Vaccine Center<br>Hannah Yellin<br>George Washington University
HVTN Community engagement unit representative	Gail Broder<br>HVTN LOC, Fred Hutch	*HPTN Community program manager*	Jonathan Lucas<br>HPTN LOC, FHI 360
HVTN Community educator/recruiter	Chelsea Greer<br>Emory Vaccine Center CRS	*HPTN Community educator/recruiter*	Ntando Yola<br>Groote Schuur HIV CRS
HVTN Protocol development manager	Meg Trahey<br>HVTN LOC, Fred Hutch	*HPTN Prevention research specialist*	Zoe Jones<br>Nicholas Hastings<br>HPTN LOC, FHI360
HVTN Project coordinator	Anders McConachie<br>Haven Wilvich<br>HVTN LOC, Fred Hutch	*HPTN Ethics Working Group representative*	Robert Klitzman<br>Columbia University

FOR REVIEW ONLY

2 Ethical considerations

It is critical that universally accepted ethical guidelines are followed at all sites involved in the conduct of clinical trials. The HIV Vaccine Trials Network (HVTN) and HIV Prevention Trials Network (HPTN) [hereafter referred to as the "Networks"] have addressed ethical concerns in the following ways:

- Network trials are designed and conducted to enhance the knowledge base necessary to find new methods for the prevention of HIV infection, using methods that are scientifically rigorous and valid, and in accordance with International Council for Harmonisation of Technical Requirements for Pharmaceuticals for Human Use (ICH) and/or other Good Clinical Practice (GCP) guidelines.

- Network scientists and operational staff incorporate the philosophies underlying major codes (1-3), declarations, and other guidance documents relevant to human subjects research into the design and conduct of HIV vaccine and prevention clinical trials.

- Network scientists and operational staff are committed to substantive community input—into the planning, conduct, and follow-up of its research—to help ensure that locally appropriate cultural and linguistic needs of study populations are met. Community Advisory Boards (CAB) are required by DAIDS and supported at all Network research sites to ensure community input, in accordance with Good Participatory Practices (GPP) and all local and national guidelines."

- Network clinical trial staff counsel study participants routinely on how to reduce HIV risk. Participants who become HIV-infected during the trial are provided counseling on notifying their partners and about HIV infection according to local guidelines. Staff members will also counsel them about reducing their risk of transmitting HIV to others.

- The Networks require that all sites develop plans for the care and treatment of participants who acquire HIV infection during a trial. Each plan may be developed in consultation with representatives of host countries, communities from which potential trial participants will be drawn, sponsors, and the Networks. Participants who become HIV-infected during the trial are referred to medical practitioners to manage their HIV infection and to identify potential clinical trials they may want to join. If a program for antiretroviral therapy (ART) provision is not available at a CRS and ART is needed, a privately established fund will be used to pay for access to treatment to the fullest extent possible.

- The Networks provide training so that all participating sites similarly ensure fair participant selection, protect the privacy of research participants, and obtain meaningful informed consent. During the study, participants will have

FOR REVIEW ONLY

their wellbeing monitored, and to the fullest extent possible, their privacy protected. Participants may withdraw from the study at any time.

- Prior to implementation, Network trials are rigorously reviewed by scientists who are not involved in the conduct of the trials under consideration.

- Network trials are reviewed by local and national regulatory bodies and are conducted in compliance with all applicable national and local regulations.

- The Networks design their research to minimize risk and maximize benefit to both study participants and their local communities. For example, Network protocols provide enhancement of participants' knowledge of HIV and HIV prevention, as well as counseling, guidance, and assistance with any social impacts that may result from research participation. Network protocols also include careful medical review of each research participant's health conditions and reactions to study products while in the study.

- Network research aims to benefit local communities by directly addressing the health and HIV prevention needs of those communities and by strengthening the capacity of the communities through training, support, shared knowledge, and equipment. Researchers involved in Network trials are able to conduct other critical research in their local research settings.

- The Networks value the role of in-country Institutional Review Boards (IRBs), Ethics Committees (ECs), and other Regulatory Entities (REs) as custodians responsible for ensuring the ethical conduct of research in each setting.

FOR REVIEW ONLY

3 IRB/EC review considerations

US Food and Drug Administration (FDA) and other US federal regulations require IRBs/ECs/REs to ensure that certain requirements are satisfied on initial and continuing review of research (Title 45, Code of Federal Regulations (CFR), Part 46.111(a) 1-7; 21 CFR 56.111(a) 1-7). The following section highlights how this protocol addresses each of these research requirements. Each Network Investigator welcomes IRB/EC/RE questions or concerns regarding these research requirements.

This trial is being conducted in countries outside of the United States, with funding from the US NIH among others. Due to this, the trial is subject to both US and local regulations and guidelines on the protection of human research subjects and ethical research conduct. Where there is a conflict in regulations or guidelines, the regulation or guideline providing the maximum protection of human research subjects will be followed.

In compliance with international and local (as appropriate) ICH and/or other GCP guidelines, each research location has a locally-based Principal Investigator (PI) who is qualified to conduct (and supervise the conduct of) the research; and the research addresses an important local health need for an HIV prevention method. In addition, the investigators take responsibility for the conduct of the study and the control of the study products, including obtaining all appropriate regulatory and ethical reviews of the research.

3.1 Minimized risks to participants

45 CFR 46.111 (a) 1 and 21 CFR 56.111 (a) 1: Risks to subjects are minimized.

This protocol minimizes risks to participants by (a) correctly and promptly informing participants about risks so that they can join in partnership with the researcher in recognizing and reporting harms; (b) respecting local/national blood draw limits; (c) performing direct observation of participants post study product administration and collecting information regarding side effects for several days post study product administration; (d) having staff properly trained in administering study procedures that may cause physical harm or psychological distress, such as blood draws, study product administrations, HIV testing and counseling and HIV risk reduction counseling; (e) providing HIV risk reduction counseling and checking on contraception use (for persons assigned female at birth); and (f) providing safety monitoring.

3.2 Reasonable risk/benefit balance

45 CFR 46.111(a) 2 and 21 CFR 56.111(a) 2: Risks to subjects are reasonable in relation to anticipated benefits, if any, to subjects, and

FOR REVIEW ONLY

the importance of the knowledge that may reasonably be expected to result.

In all public health research, the risk-benefit ratio may be difficult to assess because the benefits to a healthy participant are not as apparent as they would be in treatment protocols, where a study participant may be ill and may have exhausted all conventional treatment options. However, this protocol is designed to minimize the risks to participants while maximizing the potential value of the knowledge it is designed to generate.

3.3 Equitable participant selection

45 CFR 46.111 (a) 3 and 21 CFR 56.111 (a) 3: Subject selection is equitable

This protocol has specific inclusion and exclusion criteria for investigators to follow in admitting participants into the protocol. Participants are selected because of these criteria and not because of positions of vulnerability or privilege. Investigators are required to maintain screening and enrollment logs to document volunteers who screened into and out of the protocol and for what reasons.

3.4 Appropriate informed consent

45 CFR 46.111 (a) 4 and 5 and 21 CFR 56.111 (a) 4 and 5: Informed consent is sought from each prospective subject or the subject's legally authorized representative as required by 45 CFR 46.116 and 21 CFR Part 50; informed consent is appropriately documented as required by 45 CFR 46.117 and 21 CFR 50.27

The protocol specifies that informed consent must be obtained before any study procedures are initiated and assessed throughout the trial (see Section 9.1). Each CRS is provided training in informed consent by the and is required to have a Standard Operating Procedure (SOP) on the informed consent process. The Networks require a signed consent document for documentation, in addition to chart notes or a consent checklist.

3.5 Adequate safety monitoring

45 CFR 46.111 (a) 6 and 21 CFR 56.111 (a) 6: There is adequate provision for monitoring the data collected to ensure the safety of subjects.

This protocol has extensive safety monitoring in place (see Section 11). Safety is monitored daily by HVTN clinical staff and routinely by the HVTN 140/HPTN

FOR PREVIEW ONLY

101 Protocol Safety Review Team (PSRT). In addition, the HVTN Safety Monitoring Board (SMB) periodically reviews study data.

3.6 Protect privacy/confidentiality

45 CFR 46.111 (a) 7 and 21 CFR 56.111 (a) 7: There are adequate provisions to protect the privacy of subjects and maintain the confidentiality of data.

Privacy refers to an individual's right to be free from unauthorized or unreasonable intrusion into his/her private life and the right to control access to individually identifiable information about him/her. The term "privacy" concerns research participants or potential research participants as individuals whereas the term "confidentiality" is used to refer to the treatment of information about those individuals. This protocol respects the privacy of participants by informing them about who will have access to their personal information and study data (see Appendix A and Appendix B). The privacy of participants is protected by assigning unique identifiers in place of the participant's name on study data and specimens. In the United States, research participants in Network protocols are protected by a Certificate of Confidentiality from the US NIH, which can prevent disclosure of study participation even when that information is requested by subpoena. Participants are told of the use and limits of the certificate in the study consent form. In addition, each staff member at each study CRS in this protocol signs an Agreement on Confidentiality and Use of Data and Specimens with the Networks. In some cases, a comparable confidentiality agreement process may be acceptable. Each study CRS participating in the protocol is required to have a standard operating procedure on how the staff members will protect the confidentiality of study participants.

FOR REVIEW ONLY

4 Background

4.1 Rationale for trial concept

In 2017, more than 35 million people were living with HIV, and 1.8 million people were newly infected with the virus (4). There were 940,000 deaths attributable to HIV infection. One reason that such high rates of HIV-related deaths continue to occur globally – despite the advent of drugs that are highly effective at suppressing HIV replication – is that only about half of people living with HIV have access to antiretroviral therapy (ART). Another reason for continued HIV-related mortality is that ART does not cure HIV infection and must be maintained for a lifetime. Even in the United States (US), only 49% of the 1.1 million people living with HIV have suppressed virus to undetectable levels, despite the fact that most HIV-infected people in the US have access to ART (5). It is clear that antiretroviral therapy is necessary but not sufficient to end the AIDS epidemic, both in the US and globally, and that novel efforts to prevent HIV are critically needed.

The recent discovery of multiple potent and broadly neutralizing antibodies (bnAbs) against HIV has led to the re-emergence of the concept that passive administration of antibodies may be useful for prevention (6). HIV-specific antibodies that target the HIV envelope protein (Env) can prevent simian-human immunodeficiency virus (SHIV) infection in rhesus monkeys and have been shown to reduce HIV RNA levels in humans (7-22). Until recently, however, these antibodies were few in number, targeted a narrow spectrum of HIV strains and Env epitopes, and were not potent enough for practical use. In the last decade, the field has changed dramatically: new developments in high throughput single-cell PCR-amplification and novel soluble Env baits have led to the isolation of new monoclonal antibodies with extraordinary potency and breadth (6, 23, 24). These bnAbs may be effective for prevention of HIV infection when administered passively (6, 25, 26).

One of the bnAbs currently under investigation is PGDM1400, a broadly neutralizing mAb that was identified from African donor 84 of the IAVI Protocol G cohort. PGDM1400 is a human mAb that interacts with glycans in the region of N160 on the V2 loop of gp120 Env (27). The PGDM1400 antibody is exceptionally broad and potent, with 60% global coverage at a median IC80 of < 1 mcg/mL, and is, therefore, 10 to 100-fold more potent than the previous best in class CD4bs antibodies VRC01 and VRC07 as shown in Figure 4-1, top panel (23, 27-29). PGDM1400 has been demonstrated to be safe and well-tolerated in phase 1 trials, and to have a median half-life of about 20 days (Yunda Huang/Dan Barouch, personal communication). In vitro models suggest that administering these mAbs in combination will produce more potent and broader neutralization than administering any one mAb by itself (compare solid and dotted lines in Figure 4-1, bottom panel). Comparison of the solid lines suggests that administering all 3 mAbs in combination may produce neutralization superior to any of 2 of the mAbs in combination.

FOR REVIEW ONLY

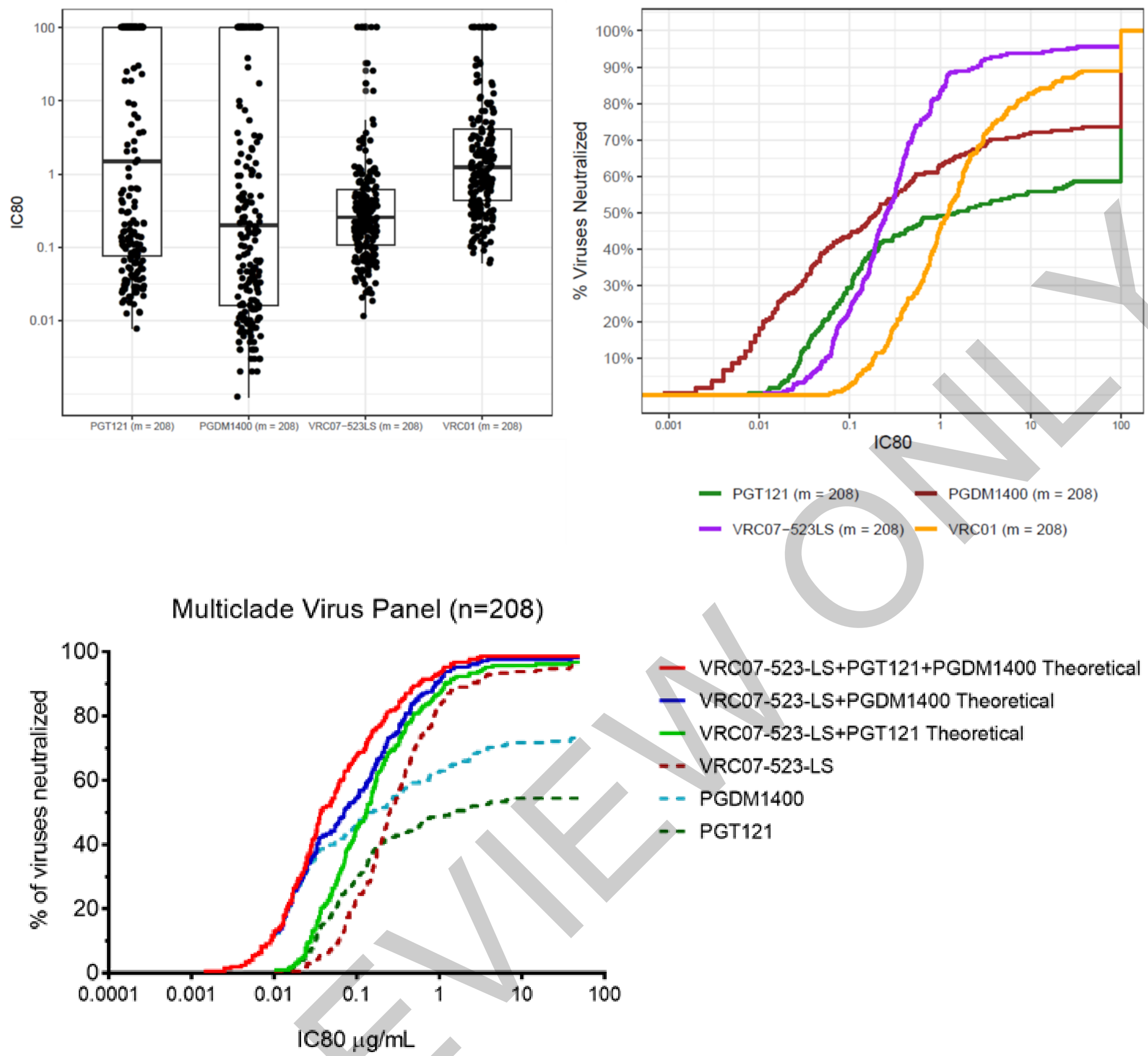

Figure 4-1 Neutralization Sensitivity (IC80) to the 208-virus VRC Multi-Clade panel for PGDM1400, PGT1221, VRC07-523LS and VRC01 (top panel) and predicted breadth and potency curves for PGT121, PGDM1400, VRC07-523LS and combinations of these mAbs against a panel of multiclade HIV isolates (bottom panel).

This half-life of about 20 days limits the use of PGDM1400 in settings where frequent dosing may be a challenge. As a result, PGDM1400 was modified in the laboratory through rational engineering to produce a new bnAb, PGDM1400LS. This modified bnAb is designed to have a longer half-life, while maintaining the potency and breadth of parental PGDM1400. There is no clinical experience with this bnAb alone or in combination with a second or third bnAb, such as a CD4 binding site mAb like VRC07-523LS and/or a V3 binding mAb like PGT121.414.LS.

This first study of the PGDM1400LS mAb is a phase 1, dose-escalation, open-label clinical trial to examine the safety, tolerability, dose, and pharmacokinetics of PGDM1400LS alone and in combination with VRC07-523LS and PGT121.414.LS. The hypothesis is that these regimens will be safe for

FOR REVIEW ONLY

administration to healthy adults and will have a favorable PK profile by the IV and SC routes. Healthy adults 18-50 years of age will be enrolled. In Part A of the study, PGDM1400LS will be administered alone as a single IV infusion at 5, 20, or 40 mg/kg (Groups 1, 2, and 4) and as a single SC infusion at 20 or 40 mg/kg (Groups 3 and 5). In Part B of the study, participants will receive two doses 4 months apart of PGDM1400LS + VRC07-523LS + PGT121.414.LS administered in two dosing approaches: (1) weight-based dosing at 20 or 40 mg/kg each IV or at 20 mg/kg each SC (Groups 6, 7 and 10); or (2) a fixed dose of 1.4 g each, administered IV or SC (Groups 8 and 9). Participants will be followed for 24 weeks after the last study product administration.

4.1.1 Cross-network implementation

DAIDS has requested that its two major prevention trial Networks—the HVTN and the HPTN—work together in the rapid development of HIV-directed bnAbs for both the advancement of vaccine research and HIV prevention purposes. This priority program leverages the historical partnership between the HVTN and the VRC (a major developer of anti-HIV bnAbs) and other commercial developers, the strong portfolio of biomedical-based HIV prevention trials that the HVTN and HPTN have developed over the past 2 decades, the multidisciplinary expertise of investigators in each Network, and the Networks' complementary laboratory and statistical expertise. In addition, the two Networks have complementary CRSs that allow for rapid enrollment of participants, worldwide. The engagement of CRSs from both Networks, particularly in early phase trials, accelerates recruitment, diversifies the trial cohort, and builds capacity for the conduct of future bnAb efficacy trials.

4.2 PGDM1400LS

PGDM1400 is a broadly neutralizing mAb identified from African donor 84 of the IAVI Protocol G cohort that targets a V2 apex epitope region of the human immunodeficiency virus-1 (HIV-1) envelope protein. PGDM1400LS is an engineered variant of PGDM1400 designed to increase its binding affinity for the neonatal Fc receptor (FcRn). The LS designation specifies methionine to leucine (L) and asparagine to serine (S) (M428L/N434S, referred to as LS) changes within the C-terminus of the heavy chain constant region far outside of the antigen-combining site (30). As a result of enhanced FcRn function, PGDM1400LS is anticipated to have an extended half-life in both serum and mucosal tissue compared to PGDM1400. Other than the two amino acid difference, PGDM1400LS is identical to PGDM1400. PGDM1400LS drug substance was manufactured under cGMP standards for NIAID by Just–Evotec, under contract to DAIDS's VTRB. The drug product was filled and released at the VRC Pilot Plant, operated under contract by VCMP.

FOR REVIEW ONLY

4.3 PGT121.414.LS

PGT121.414.LS was produced by Just Biotherapeutics in collaboration with Dan Barouch (Beth Israel Deaconess Medical Center), and collaborative engagement of CAVD investigators. The drug substance was manufactured under cGMP standards at Just Biotherapeutics under contract to DAIDS's VTRB. The drug product was filled and released for the VRC by the VCMP, Leidos Biomedical Research, Inc., Frederick, MD. The PGT121.414.LS mAb is an engineered variant of PGT121. It contains a total of 8 residue modifications to improve various aspects of manufacturing, stability and in vivo elimination half-life. Six of the modifications are in the Fragment crystallizable (Fc) region, providing increased conformational stability leading to improved manufacturing characteristics including low pH stability and an improved storage stability profile. The 2 modifications in the Fc region of each heavy chain are the Xencor Xtend LS modifications helping provide a significantly reduced elimination half-life in vivo (30, 31). The magnitude and breadth of neutralizing activity of PGT121.414.LS and its parent PGT121 in vitro were shown to be nearly equivalent against a multiclade panel of Env-pseudotyped viruses (Figure 4-2).

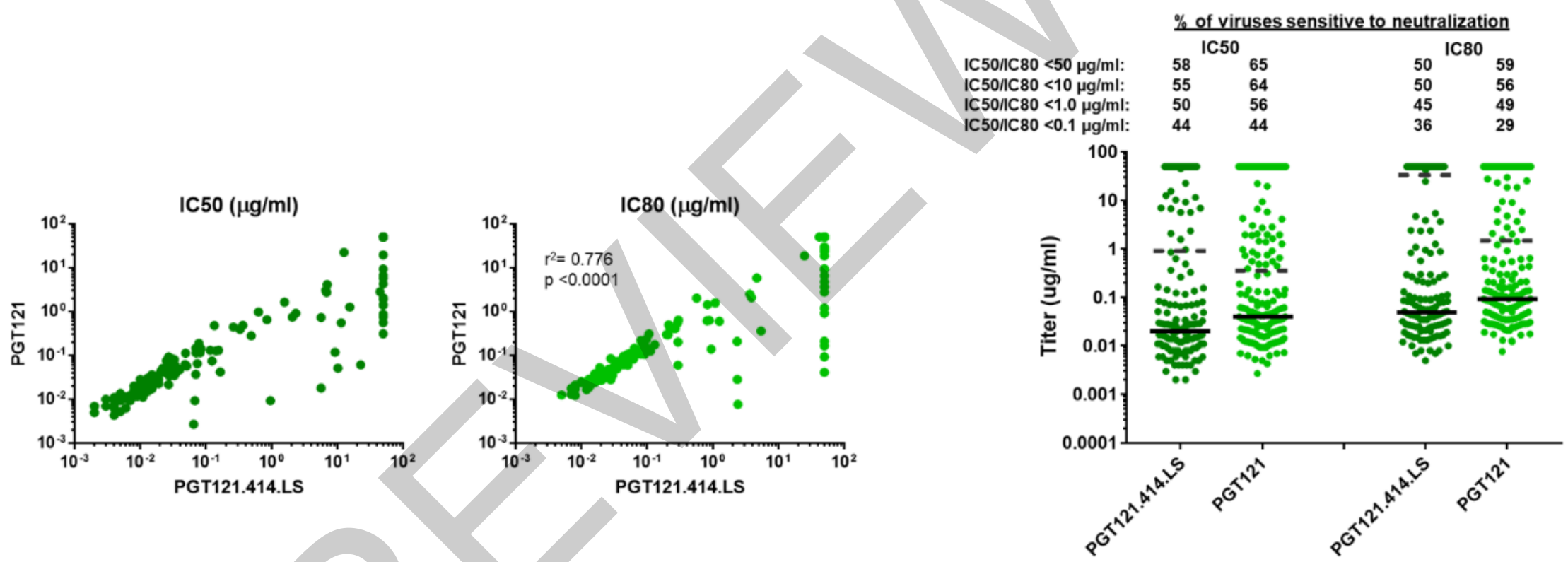

Figure 4-2 Comparison of in vitro neutralizing activity of PGT121 and PGT121.414.LS against a multiclade pseudovirus panel (n=208). 50 mcg/mL was the highest concentration tested and is used as the cut-off for negative neutralization. Dotted line shows median IC50 and IC80 of all viruses (including those not neutralized). Solid line shows median IC50 and IC80 of viruses sensitive to neutralization (excluding those not neutralized). Data courtesy of the Vaccine Research Center, NIH.

The bnAb, PGT121, was identified from African donor 17 of the International AIDS Vaccine Initiative (IAVI) Protocol G cohort. It targets the V3 glycan-dependent epitope region of the HIV-1 virus. This epitope on the gp120 outer domain includes both protein and glycans and is centered on the conserved residue N332 (32-34). Using a 162-pseudovirus panel, representative of all major HIV-1 circulating clades, the PGT121 had a 10-fold higher median neutralizing potency than mAbs PG9, VRC01, or PGV04 and a 100-fold higher potency than 2G12, b12, or 4E10 (23). While PGT121 neutralized a smaller percentage of the panel of pseudoviruses than VRC01 at an IC50 < 50 mcg/mL (63% for PGT121 vs. 93% for VRC01), it exhibited high potency against the sensitive strains, with neutralization of 44% of the 162-virus panel at an IC50 < 0.1 mcg/mL. This percentage is almost twice the neutralization under the same conditions as PG9,

FOR REVIEW ONLY

VRC01, PGV04 and 20–40 times more neutralizing than 2G12, b12, and 4E10- all of which have been investigated previously in passive protection studies (23, 27, 29).

4.4 VRC07-523LS (VRC-HIVMAB075-00-AB)

VRC07-523LS is a human mAb targeting the HIV-1 CD4 binding site, developed by NIAID-NIH at the VRC. A similar antibody, VRC01, also targeting the CD4 binding site, is currently in clinical trials for both HIV-1 prevention (IND 113,611 and IND 125,494) and therapeutic (IND 126,001, IND 126,664, and IND 133,017) indications. VRC01 was originally isolated from a subject infected with HIV-1 for more than 15 years whose immune system controlled the virus without ART (35, 36). Through advances in B-cell immunology, cloning, and structure-guided optimization techniques, numerous HIV-1 neutralizing mAbs, including VRC07 ("07" denotes sequential numbering when discovered), were isolated and subsequently engineered to have potency and breadth greater than those of earlier antibodies (11). This protocol will use VRC07-523LS ("523" denotes sequential numbering when the engineered variant was generated; "LS" denotes 2 amino acid mutations).

The VRC07 (wild-type) heavy chain was identified by 454 deep sequencing based on its similarity to VRC01 and was then engineered to pair it with the VRC01 (wild-type) light chain. The mutations that together define the 523 designation are a glycine-to-histidine mutation at residue 54 of the heavy chain, a deletion of the first 2 amino acids, glutamate and isoleucine, from the light chain, and a valine-to-serine mutation at the third amino acid residue of the light chain (11). The LS designation in VRC07-523LS specifies methionine to leucine (L) and asparagine to serine (S) (M428L/N434S, referred to as LS) changes within the C-terminus of the heavy chain constant region. The LS mutation was introduced by site-directed mutagenesis to increase the binding affinity for the neonatal Fc-receptor (FcRn); this mutation increases the recirculation of functional immunoglobulin G (IgG) (14, 30), thus, increasing plasma half-life. VRC07-523LS was found to be 5- to 8-fold more potent than VRC01, with an IC50 < 50 mcg/mL against 96% of HIV-1 pseudoviruses representing the major circulating HIV-1 clades, and an IC50 < 1 mcg/mL against 92% of HIV-1 viruses tested (11).

4.5 Trial design rationale

4.5.1 Dose (amount and number)

The doses chosen for this trial are based upon observations of the neutralization potency required to achieve protective efficacy in an NHP meta-analysis (37) and in the AMP studies (38) (see Figure 4-3) as well as models of neutralization potency and coverage of bnAb combinations with IV or SC administration (see Figure 4-4). Both the AMP studies and the NHP meta-analysis suggested that achieving over 80% prevention efficacy may require an ID80 of between 200-400

FOR REVIEW ONLY

(Figure 4-3). Achieving those ID80 titers may require mAb doses of at least 60 mg/kg, combined (eg, 20 mg/kg each of 3 bnAbs) and quite possibly higher, particularly for subcutaneous administration (Figure 4-4). Subcutaneous bioavailability has ranged from about 35% of IV bioavailability for PGT121 (Y Huang, personal communication) to about 75% for VRC01 (39).

The highest dose of bnAbs that will be administered in this study, 120 mg/kg, is considerably less than the approved dosages for a variety of intravenous immunoglobulin (IVIG) products currently licensed for use in the US (eg, 200-800 mg/kg Privigen every 2-4 weeks for primary immune deficiency).

This trial is designed to evaluate both SC and IV routes, as SC administration may be more feasible than IV in some settings, and the comparison of these routes will elucidate the bioavailability of PGDM1400LS SC vs. IV and, thus, inform future decisions regarding dose, frequency and route of PGDM1400LS administration. Toward informing future dosing decisions, the trial is also designed to evaluate both weight-based and fixed dosing. The fixed dose of 4.2 g of total mAb (1.4 g of each mAb) would be equivalent to a weight-based dose of 60 mg/kg of total mAb (20 mg/kg each) for a 70 kg participant; 70 kg is the approximate median of participants' baseline weights in the AMP studies (38). A PK model of administration of a 4.2 g fixed dose underscores the comparability of this dose to a 60 mg/kg dose in terms of coverage at an ID80 >200 (see Figure 4-5).

The effect of body weight on drug biodistribution has been observed for some monoclonal antibodies (39-43), but body-weight-based dosing is not clearly necessary in human adults, who have a body size range of generally only 2- to 3-fold (44, 45). When body weight has a significantly less than proportional effect on PK parameters, body-weight-based dosing may over-adjust for the body size effect. Fixed dosing simplifies administration, potentially enhancing safety by reducing dosing errors, and it can be more cost-effective and convenient to manufacture and administer. Of note, the anti-SARS-CoV-2 mAbs in late-stage clinical trials or post-EUA are administered in fixed doses (evaluated at up to 8 g total per administration), as are many mAbs used for oncologic and anti-inflammatory indications. Whether fixed dosing is equivalent to body-weight-based dosing for a given mAb is unknown until studied in the target population (eg, adult humans) and there is a growing call to assess it early in clinical development programs. (44-46).

FOR REVIEW ONLY

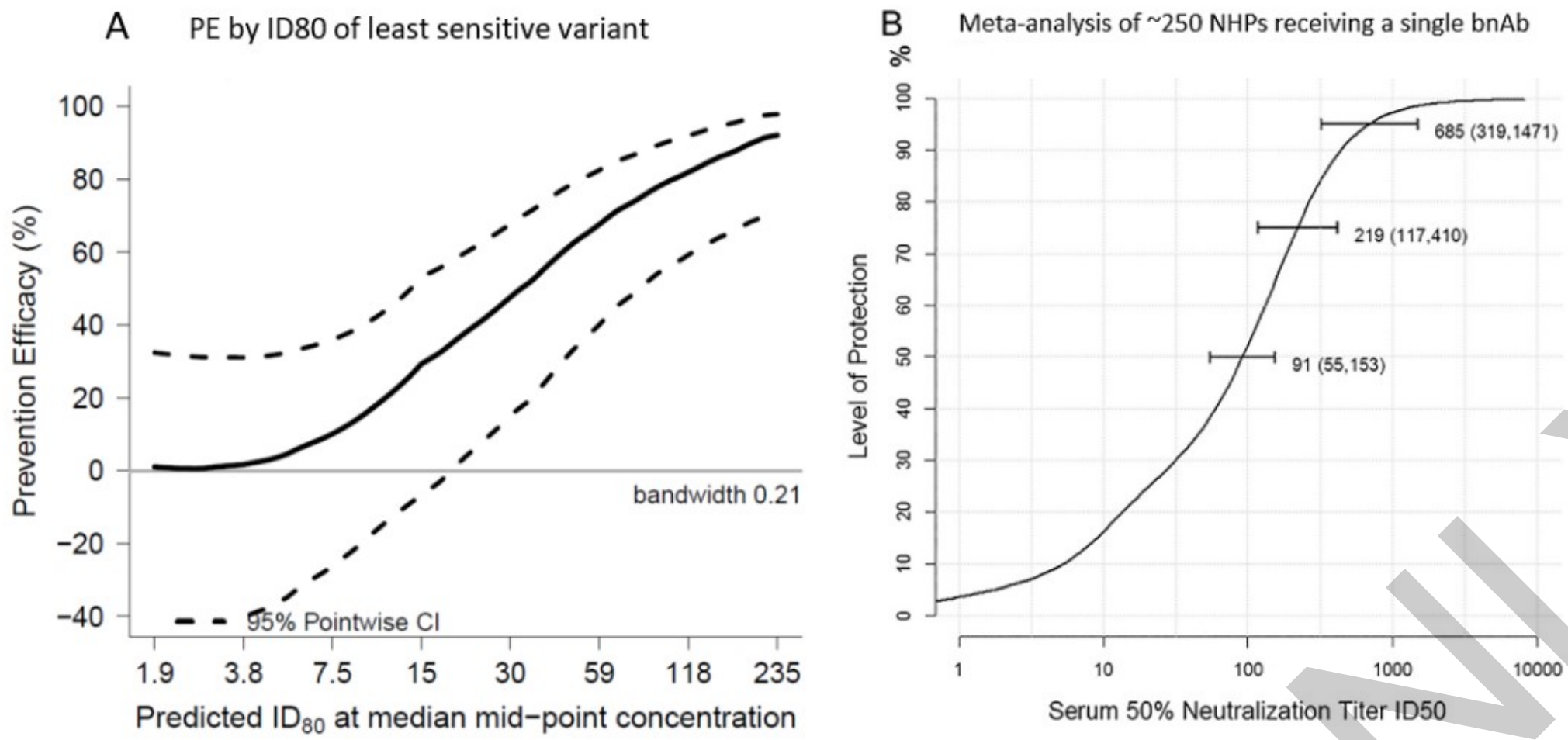

Figure 4-3 Relationship between neutralization titers and level of prevention efficacy. (A) In humans in the AMP studies. predicted ID80 serum neutralizing titer is calculated as the median VRC01 concentration at mid-infusion visits (35.0 µg/ml) divided by IC80 (concentration from BAMA) of the acquired virus (n=162 infected cases) from the AMP trials. The median concentration was computed based on the n=82 non-case VRC01 recipients from both dose arms sampled for PK modeling (Y. Huang, unpublished data). (B) In a meta-analysis of NHP receiving a single bnAb (37).

Ideally, this study will demonstrate that PGDM1400LS alone and in combination with VRC07-523LS and PGT121.414.LS is safe and well-tolerated when given IV and SC, and that the half-life of PGDM1400LS is significantly extended compared to parental PGDM1400, as well as unchanged by co-administration with VRC07-523LS and PGT121.414.LS. The study also aims to discern whether sera from infused participants retain the same neutralizing breadth in vitro as parental PGDM1400 alone and in combination with VRC07-523LS and PGT121.414.LS.

This study will be part of a series of studies that aim to evaluate safety and tolerability of mAbs and whether combining mAbs that target different sites on the virus is additive or synergistic with respect to neutralization breath and potency. These data will be critical for designing future mAb studies.

FOR REVIEW ONLY

A Average coverage in the first week IV, total dose amount 60 mg/kg

Virus Panel	IV VRC01 30mg/kg (reference)	IV PGT121 + PGDM1400 + VRC07-523LS (Total dose = 60 mg/kg)		
		1-Active (min- max)	2-Active (min- max)	3-Active (min- max)
VRC Multi-clade (m=208)	38%	86.7% - 94.1%	58.7% - 73.4%	15.7% - 26.9%
VRC Acute Clade C (m=200)	20%	80.3% - 92.1%	42% - 62.5%	8.7% - 15.8%
CATNAP Clade C (m=228)	22%	80.2% - 91.9%	45.2% - 65%	10.9% - 18.9%
CATNAP Clade B (m=41)	35%	60.9% - 70.6%	19.7% - 27.5%	2.9% - 4.7%

B Average coverage in the first week: SC (bioavailability = 35%), total dose amount 60 mg/kg

Virus Panel	IV VRC01 30mg/kg (reference)	PGT121 + PGDM1400 + VRC07-523LS (Total dose = 60 mg/kg)		
		1-Active (min- max)	2-Active (min- max)	3-Active (min- max)
VRC Multi-clade (m=208)	38%	76% - 89.2%	27.7% - 62%	3.6% - 18.9%
VRC Acute Clade C (m=200)	20%	68.9% - 84.4%	19.6% - 46.6%	3.3% - 10.4%
CATNAP Clade C (m=228)	22%	70.2% - 83.5%	21.9% - 49.6%	4.8% - 12.9%
CATNAP Clade B (m=41)	35%	51% - 63.9%	12.4% - 19.9%	1% - 3.3%

C Average coverage in the first week: SC (bioavailability = 75%), total dose amount 60 mg/kg

Virus Panel	IV VRC01 30mg/kg (reference)	PGT121 + PGDM1400 + VRC07-523LS (Total dose = 60 mg/kg)		
		1-Active (min- max)	2-Active (min- max)	3-Active (min- max)
VRC Multi-clade (m=208)	38%	82.6% - 92.9%	48.2% - 71.3%	9.1% - 25.2%
VRC Acute Clade C (m=200)	20%	75.1% - 90.8%	33% - 58.3%	6.6% - 14.4%
CATNAP Clade C (m=228)	22%	75.8% - 90.4%	35.8% - 61.2%	8.1% - 17.2%
CATNAP Clade B (m=41)	35%	57.4% - 69.4%	16.1% - 26.1%	1.8% - 4.5%

Figure 4-4 Average coverage in the first week for IV bnAb combinations with a total dose of 60 mg/kg (A); average coverage in the first week for SC bnAb combinations [bioavailability = 35% (B) or 75% (C)] with a total dose amount 60 mg/kg. 1-Active, 2-Active, and 3-Active refer to the number of bnAbs in the combination that provide coverage over the viruses in the specified panel at ID80 >200 min-max % coverage across all dose combinations. m= number of viruses in the panel.

FOR REVIEW ONLY

A Average coverage in the first week: IV, total fixed dose 4.2 g

Virus Panel	IV VRC01 30mg/kg	PGT121 + PGDM1400 + VRC07-523LS		
		1-Active	2-Active	3-Active
VRC Multi-clade (m=208)	38%	94.10%	73.40%	26.70%
VRC Acute Clade C (m=200)	20%	92.10%	62.40%	15.20%
CATNAP Clade C (m=228)	22%	91.90%	64.70%	18.70%
CATNAP Clade B (m=41)	35%	70%	27.50%	4.70%

B Average coverage in the first week: SC (bioavailability = 35%), total fixed dose 4.2 g

Virus Panel	IV VRC01 30mg/kg	PGT121 + PGDM1400 + VRC07-523LS		
		1-Active	2-Active	3-Active
VRC Multi-clade (m=208)	38%	86%	59.50%	17.40%
VRC Acute Clade C (m=200)	20%	79.80%	44.70%	9.50%
CATNAP Clade C (m=228)	22%	79.30%	47.60%	11.20%
CATNAP Clade B (m=41)	35%	61.10%	19%	3.20%

C Average coverage in the first week: SC (bioavailability = 75%), total fixed dose 4.2 g

Virus Panel	IV VRC01 30mg/kg	PGT121 + PGDM1400 + VRC07-523LS		
		1-Active	2-Active	3-Active
VRC Multi-clade (m=208)	38%	91.60%	71.10%	24.50%
VRC Acute Clade C (m=200)	20%	89.70%	58%	13.80%
CATNAP Clade C (m=228)	22%	89.10%	60.90%	17.10%
CATNAP Clade B (m=41)	35%	68.60%	25.80%	4.40%

Figure 4-5 Average coverage in the first week for IV (A) or SC [bioavailability = 35% (B) or 75% (C)] bnAb combinations with a total fixed dose of 4.2 g. 1-Active, 2-Active, and 3-Active refer to the number of bnAbs in the combination that provide coverage over the viruses in the specified panel at ID80 >200. m= number of viruses in the panel.

4.5.2 Schedule

There is currently no human PK data on PGDM1400LS and PGT121.414.LS. Therefore, the dosing schedule in Part B was selected based on the PK modeling of PGDM1400, PGT121, and VRC07-523LS in combination, assuming no drug-drug PK interactions. Specifically, the PK modeling shown in Figure 4-6 is based on human data from n=120 HIV-uninfected participants for VRC07-523LS in HVTN 127/HPTN 087, n = 16 HIV-uninfected participants for PGT121 in Barouch 628 (T001), and n= 18 HIV-uninfected participants for PGDM1400 in Barouch 693 (T002). Initiation of testing in Part B at the interval proposed will be supported by PGDM1400LS preclinical PK data, including in FcRn mouse and non-human primate (NHP) models, and PGT121.414.LS PK data from Part A of the ongoing HVTN 136/HPTN 092 clinical trial. The half-lives of the LS variants of PGDM1400 and PGT121 are predicted to be 2-3 fold longer. Predicted serum concentrations following multiple IV infusions at 20 mg/kg every 8 to 20 weeks were generated through Monte Carlo simulations of 1000 hypothetical trial participants based on population PK models of PK data for PGT121, IV PGDM1400, and VRC07-523LS as of June 2018.

FOR REVIEW ONLY

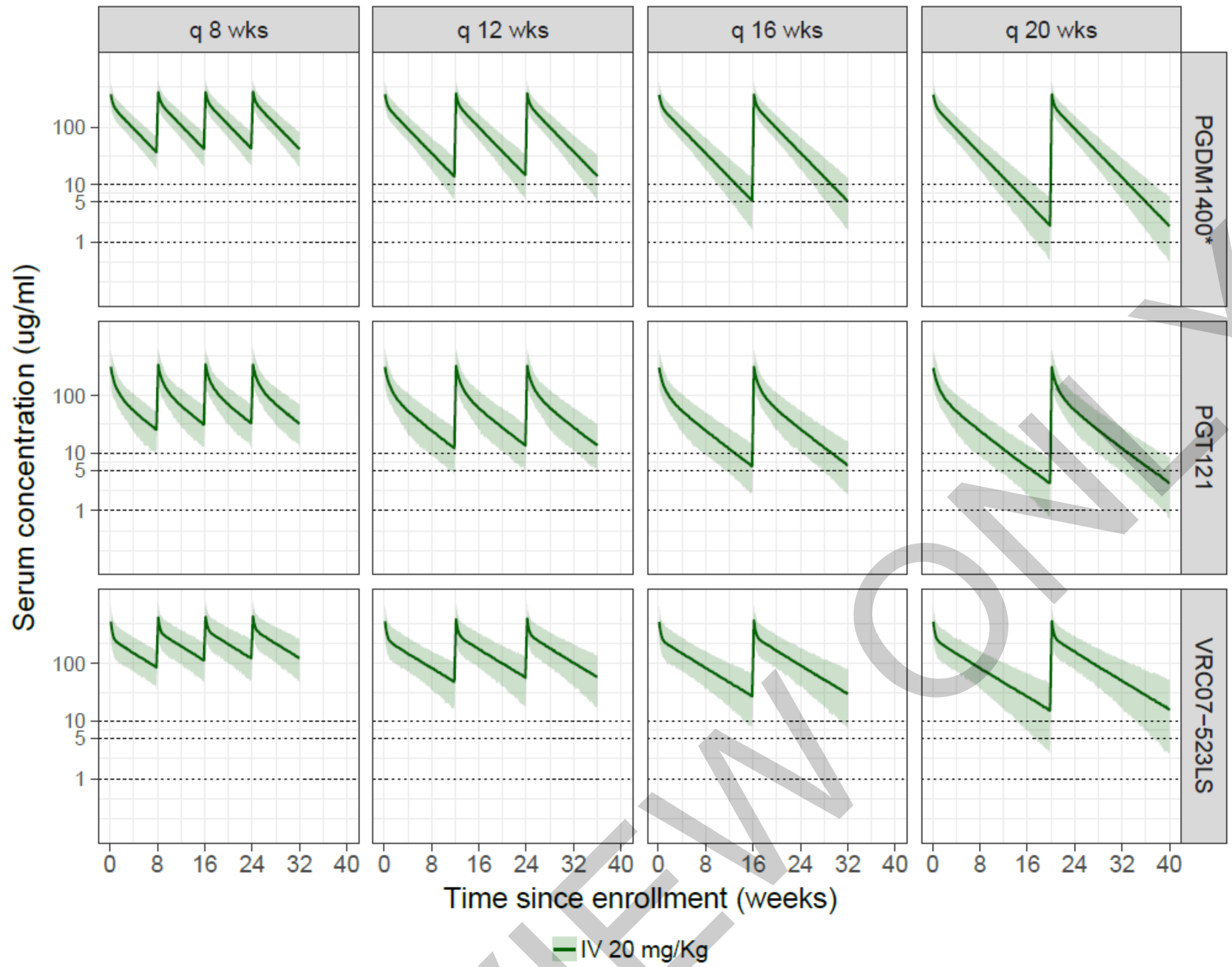

Figure 4-6 Predicted PGDM1400, PGT121 and VRC07-523LS concentration with multiple 8- to 20-weekly IV infusions at 20mg/kg. Shown are median (solid lines), 5th and 95th percentiles (shaded areas) of predicted drug levels with values truncated at half of the lower limit of quantification – 0.12 mcg/mL (PGDM1400), 0.25 mcg/mL (PGT121) or 1.1/2 mcg/mL (VRC07-523LS).

In these simulations, the body weights of the hypothetical trial participants were randomly drawn with replacement from the sample of body weights collected in the HVTN 104 study of VRC01 in US sites, some of which will be used in this study (39, 47). In addition, linear PK was assumed for the mAbs so that PK parameters estimated based on single-dose settings carried forward to multiple-dose settings. The PK parameters used in the simulations were estimated based on observed serum concentration data for PGDM1400 at 3 and 10 mg/kg following a single IV infusion, for PGT121 at 3, 10, and 30 mg/kg following a single IV infusion and at 3 mg/ kg following a single SC injection, and for VRC07-523LS at 5, 20, and 40 mg/kg following a single IV infusion, at 20 mg/ kg following three 12-weekly IV infusions, at 5mg/ kg following a single and at 5 mg/kg following three 12-weekly SC injections. The mean and variance of log-transformed serum concentrations were computed after each IV infusion. Specifically, at 16 weeks after the second IV infusion at a dose level of 20 mg/kg, the serum concentrations (mean ± standard deviation [SD]) were predicted to be 1.6 ± 0.5 for PGDM1400, 1.8 ± 0.5 for PGT121, and 3.4 ± 0.6 for VRC07-523LS. The chance of having a VRC07-523LS serum concentration > 1 mcg/mL at 48 weeks post the second IV infusion at 20 mg/kg is predicted to be about 24%.

FOR REVIEW ONLY

Given the IC_{50} and IC_{80} data of the mAbs, these PK simulation results suggest that the proposed study design should be able to attain desirable serum concentration levels over time that confer sufficient neutralization against diverse panels of viruses (see Section 4.1). Thus, based on currently available data, in Part B, a dosing interval of every 16 weeks was selected to have high probability of achieving a trough above 1 mcg/mL for all study mAbs. This threshold of 1 mcg/mL is the limit of detection of the currently used anti-idiotypic enzyme-linked immunosorbent assay (ELISA) for mAb serum concentrations.

4.6 Plans for future product development and testing

This trial aims to establish an initial safety, PK and neutralization profile for a triple-bnAb combination that is thought likely to advance to efficacy testing on the basis of early preclinical and clinical data, and informed by results from the first efficacy trials of a bnAb for HIV prevention, the AMP Studies. The approach of combining three bnAbs that bind and neutralize three independent epitopes on the HIV envelope protein is akin to triple-combination ART and appears to have the potential to make a comparable impact on the persistent HIV pandemic.

4.7 Preclinical studies

4.7.1 Preclinical studies of PGDM1400

4.7.1.1 GLP repeat dose toxicity study in rats.

In the repeat dose toxicity study, administration of PGDM1400 alone or in combination with PGT121 by once weekly subcutaneous or intravenous injection for one month (total of 5 injections) at 30 mg/kg/dose, and alone by once weekly intravenous injection at 300 mg/kg/dose, resulted in slightly higher plasma proteins in males, and a higher incidence of hemorrhage at the subcutaneous injection sites that had received PGDM1400. Neither of these findings was considered adverse, and they were not observed after a one-month recovery (treatment-free) period. No clinical signs or local reactions were noted at the injection sites that were considered to be related to the administration of PGDM1400 alone or in combination with PGT121. Body weight, food consumption, hematology, coagulation and urine volume and composition were unaffected by treatment with PGDM1400 alone or in combination. The NOAEL for intravenous administration was 300 mg/kg and for subcutaneous injection was 30 mg/kg, which were the highest dose levels administered.

4.7.1.2 Preclinical challenge study of PGDM1400

PGDM1400 was evaluated against a novel challenge stock, SHIV-325c, developed specifically for NHP challenge studies of PGDM1400, CAP256, and related variants and PGDM1400 demonstrated protection at low doses. As shown in Figure 4-7 below, rhesus macaques received a single IV infusion of PGDM1400 (n = 5 at 2 mg/kg; n = 5 at 0.4 mg/kg; n = 4 at 0.08 mg/kg) 24 hours

FOR PREVIEW ONLY

before high dose intrarectal challenge with SHIV-325c. All animals in the control group were infected between days 7 and 28. In the 2 mg/kg group, one of five animals receiving PGDM1400 became infected on Day 28; in the 0.4 mg/kg group, no animals became infected; in the 0.08 mg/kg group, three of four animals receiving PGDM1400 were infected. The average serum antibody concentrations in animals administered PGDM1400 at doses of 2, 0.4, and 0.08 mg/kg were 6.9, 2.5, and 0.22 mcg/mL, respectively, at the time of challenge. The half-life of PGDM1400 was calculated for the 2, 0.4, and 0.08 mg/kg groups to be 7.7 days, 6.7 days, and 6.3 days, respectively (28).

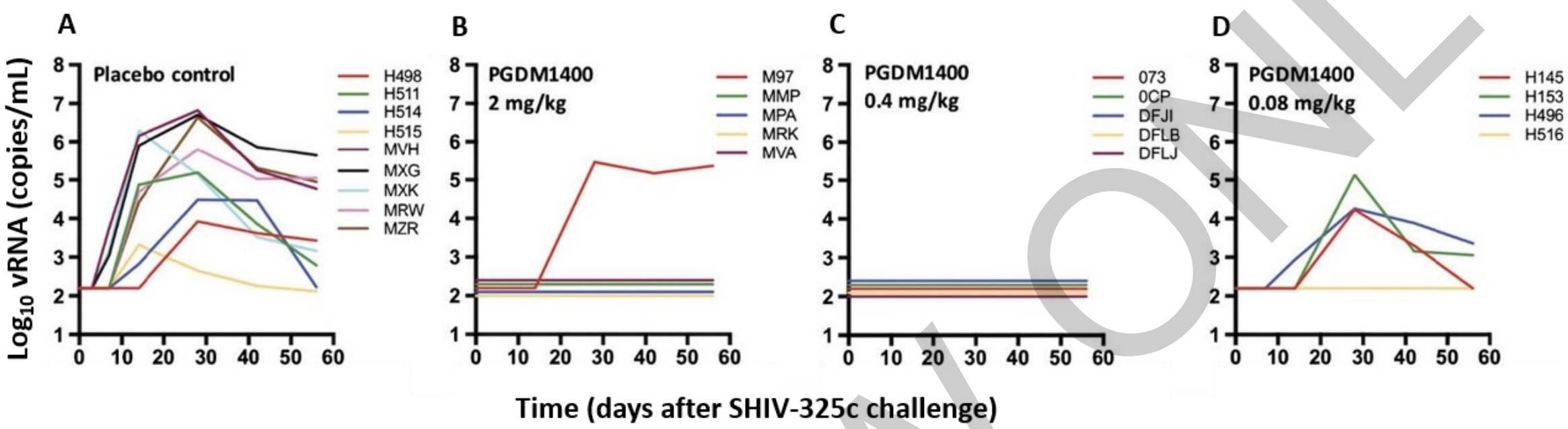

Figure 4-7 Protective efficacy of PGDM1400 against SHIV-325c in rhesus macaques. Plasma viral RNA (as log vRNA copies/mL) is shown for animals that received: (A) saline control; (B) PGDM1400 (2mg/kg); (C) PGDM1400 (0.4 mg/kg); (D) PGDM1400 (0.08 mg/kg). The assay sensitivity limit was >50 RNA copies/mL

4.7.2 Preclinical studies of PGDM1400LS

4.7.2.1 Preclinical study in rhesus macaques (Sanofi, VRC, BIDMC, Ragon Institute, Scripps, NIAID)

In 2017, L. Xu et al reported in Science (48) on the development of broadly neutralizing trispecific antibodies to HIV-1. Trispecific antibodies combine the specificities against 3 distinct epitopes on the HIV-1 envelope protein into a single molecule; one of these epitopes is the V2 binding site of PGDM1400. LS mutations were engineered into the Trispecific molecules as well as the parental antibodies, including PGDM1400. As shown in Figure 4-8B, plasma antibody levels in rhesus macaques administered 5 mg/kg IV of PGDM1400LS are comparable to that of VRC01LS which has a published half-life in humans of 71± 18 days (49). And as depicted in Figure 4-8C, three antibody specificities were required to prevent viremia in 100% of rhesus macaques challenged with a mixture of SHIV BaLP4 and SHIV 325c.

FOR REVIEW ONLY

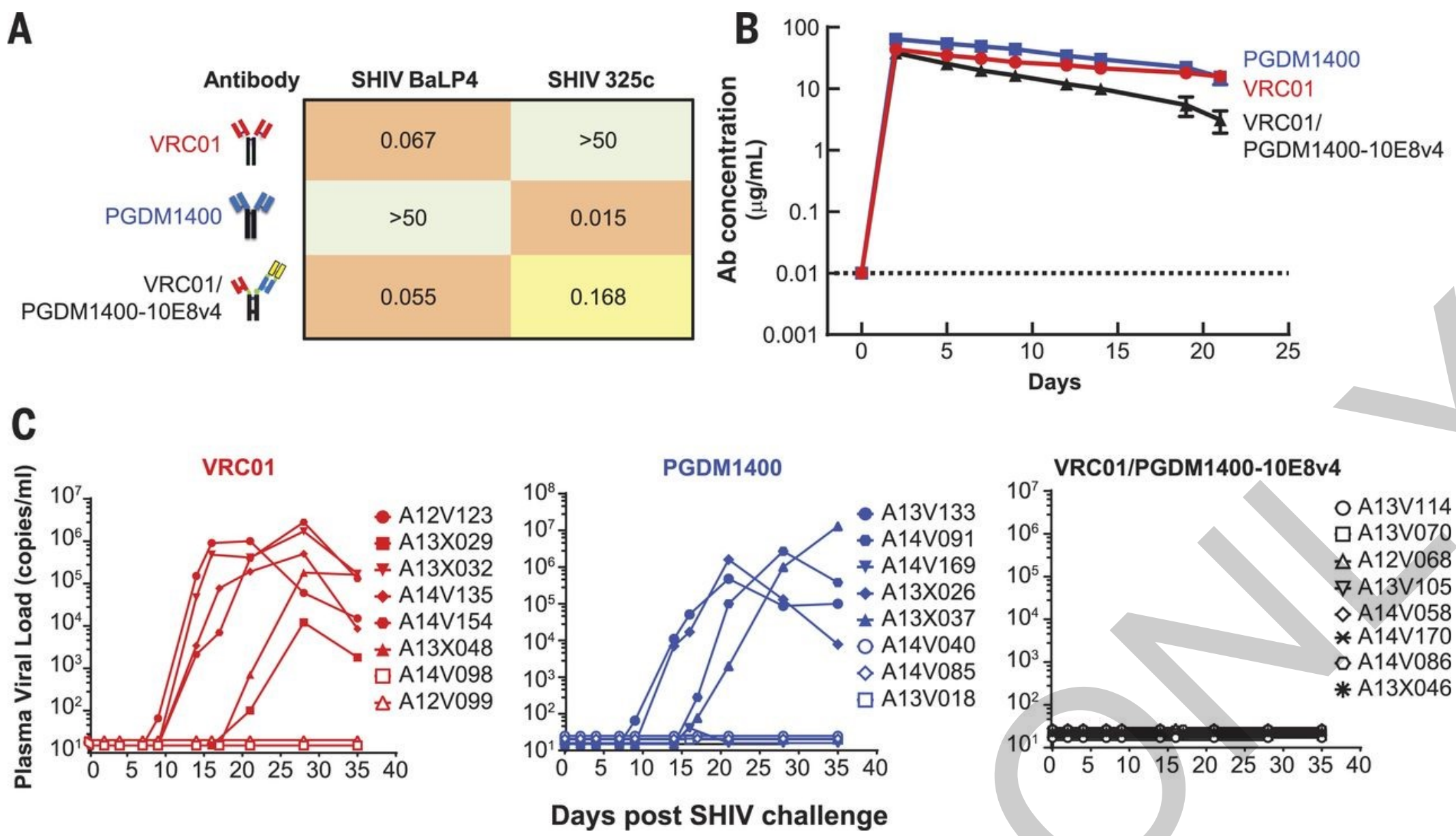

Figure 4-8 Trispecific and broad neutralizing antibody sensitivity of SHIVs, plasma antibody levels and viremia in rhesus macaques. All antibodies in the figure are LS variants. (A) The IC50 neutralizing titers (mcg/ml) of VRC01, PGDM1400, and VRC01/10E8v4-PGDM1400 against replication competent SHIV BaLP4 or SHIV 325c. (B) Plasma levels of VRC01, PGDM1400 and VRC01/PGDM1400-10E8v4 in rhesus macaques (n=8 on each arm, done in two separate experiments with 4 animals each). All animals were administered 5 mg/kg of the indicated antibody intravenously. Each data point represents the mean +/− SEM of the values from all 8 animals per group. (C) Plasma viral loads in rhesus macaques (n=8 per group) challenged with a mixture of SHIV BaLP4 and SHIV 325c, 5 days after intravenous administration of either VRC01, PGDM1400 or VRC01/PGDM1400-10E8v4. (Figure from Xu et al., (48)).

4.7.2.2 Pharmacokinetic study of PGDM1400LS in human neonatal Fc receptor (FcRn) transgenic mice

The VRC, NIAID, NIH evaluated the in vivo pharmacokinetic parameters of PGDM1400LS in human FcRn transgenic mice at a dose of 5 mg/kg via the IV route. The levels of antibody in the sera of these animals at various time points after administration were then quantified by using a HIV-1 trimer based ELISA (see Figure 4-9).

FOR REVIEW ONLY

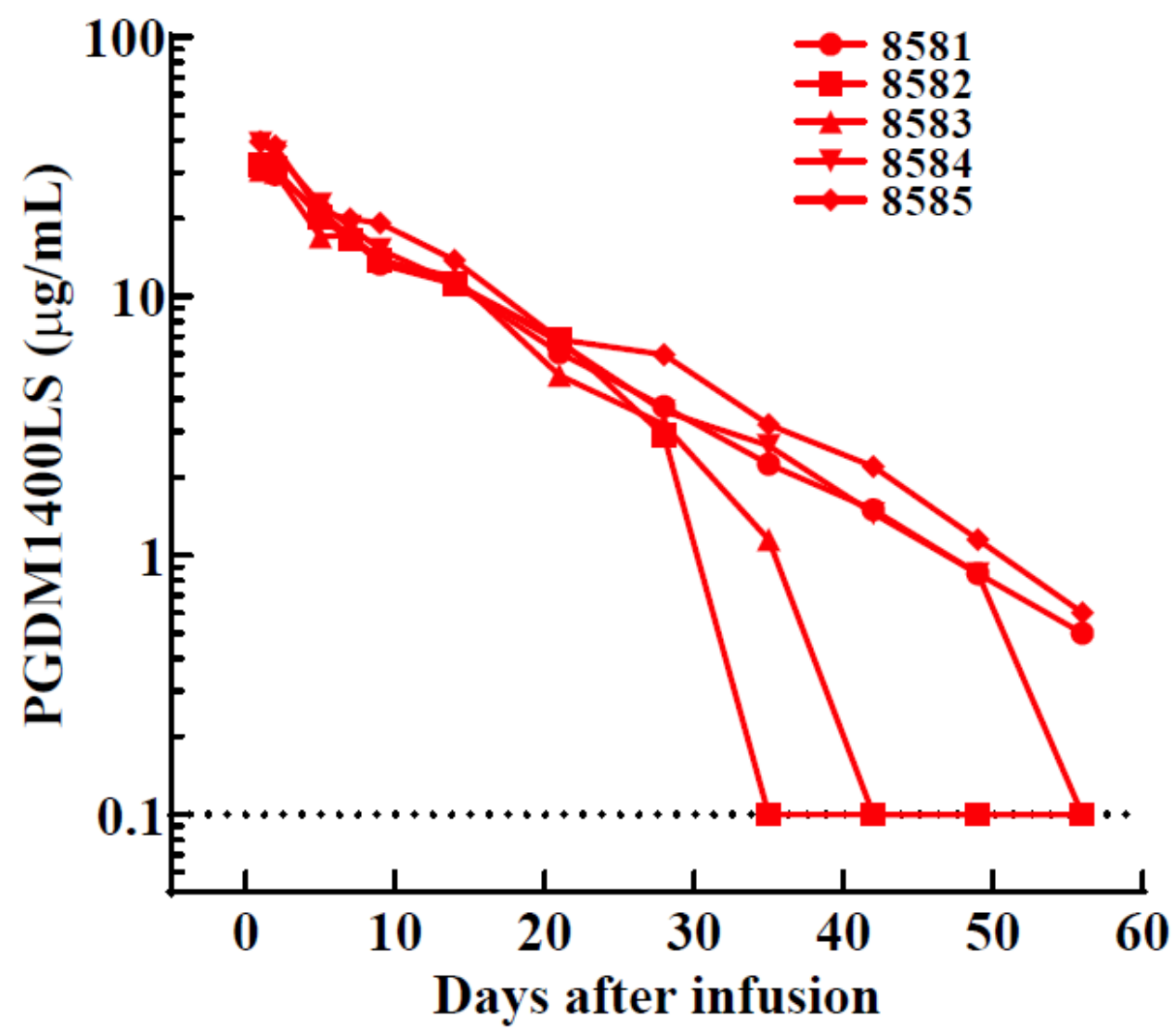

Figure 4-9 Sera levels of PGDM1400LS antibody in five human FcRn transgenic mice administered at 5 mg/kg via the IV route.

The pharmacokinetic parameters were calculated using a non-compartment model in WinNonLin software package and are summarized in Table 4-1. PGDM1400LS displayed a PK profile similar to other anti-HIV-1 antibodies in this animal model and the data provide support for the development of PGDM1400LS for clinical studies.

Table 4-1 In vivo pharmacokinetic parameters for PGDM1400LS in human FcRn transgenic mice administered at 5 mg/kg via the IV route.

Antibody	Animal ID	Half-life Day	AUC Day*µg/mL	Clearance mL/Day/kg
PGDM1400LS	8581	9.4	417	12.0
	8582	7.7	382	13.0
	8583	6.5	371	13.5
	8584	9.3	454	10.9
	8585	8.5	523	9.6
	Average	***8.3***	***429***	***11.8***
	Standard error	***0.5***	***25***	***0.6***

4.7.2.3 Binding affinity study of PGDM1400LS compared to parental PGDM1400

The VRC, NIAID, NIH compared the binding affinity of PGDM1400LS and PGDM1400. HIV Env Trimer 4571 binding to PGDM1400LS and the parental antibody PGDM1400 was measured by bio layer interferometry on a ForteBio OctetRED384. The binding affinity was measured and the equilibrium dissociation constant values were comparable (see Table 4-2)

FOR REVIEW ONLY

Table 4-2 Binding Affinity of PGDM1400LS and PGDM1400 to HIV Env Trimer 4571

Antibody	K_D (nM) ± standard error of the mean
PGDM1400LS	4.6 ± 0.9
PGDM1400	4.6 ± 0.8, 5.2 ± 0.6

4.7.2.4 In vitro neutralization study comparing PGDM1400LS with parental PGDM1400

The VRC, NIAID, NIH compared the in vitro neutralization activities of PGDM1400LS and PGDM1400 against a multiclade virus panel (n=3). The 80% inhibitory concentration (IC80) for PGDM1400LS and PGDM1400 were comparable (see Table 4-3). For additional in vitro neutralization data for PGDM1400 LS, please see the Investigator's Brochure (IB).

Table 4-3 Neutralization potency of PGDM1400LS and parental PGDM1400 in a 3-pseudovirus panel assay

Virus	Clade	IC80 (mcg/mL)		
		PGDM1400 LS (production run 5537)	PGDM1400 LS (production run 5515)	PGDM1400
MI369.A5	A	27.25	25.46	25.00
DU156.12	C	17.37	16.54	16.00
A03349MI.vrc4a	D	428.47	238.65	320.00

4.7.2.5 Autoreactivity evaluation of PGDM1400LS

The VRC, NIAID, NIH assessed potential reactivity to human epithelial type 2 (HEp-2) cells and to phospholipid cardiolipin. The reactivity to HEp-2 cells was assessed by immunofluorescence staining (see Figure 4-10) and potential binding to cardiolipin by ELISA (see Figure 4-11). 4E10 and VRC01LS bnAbs were used as positive and negative controls, respectively. The results showed that PGDM1400LS did not display detectable binding to either HEp-2 cells or cardiolipin, suggesting that PGDM1400LS does not exhibit detectable polyspecific autoreactivity.

FOR REVIEW ONLY

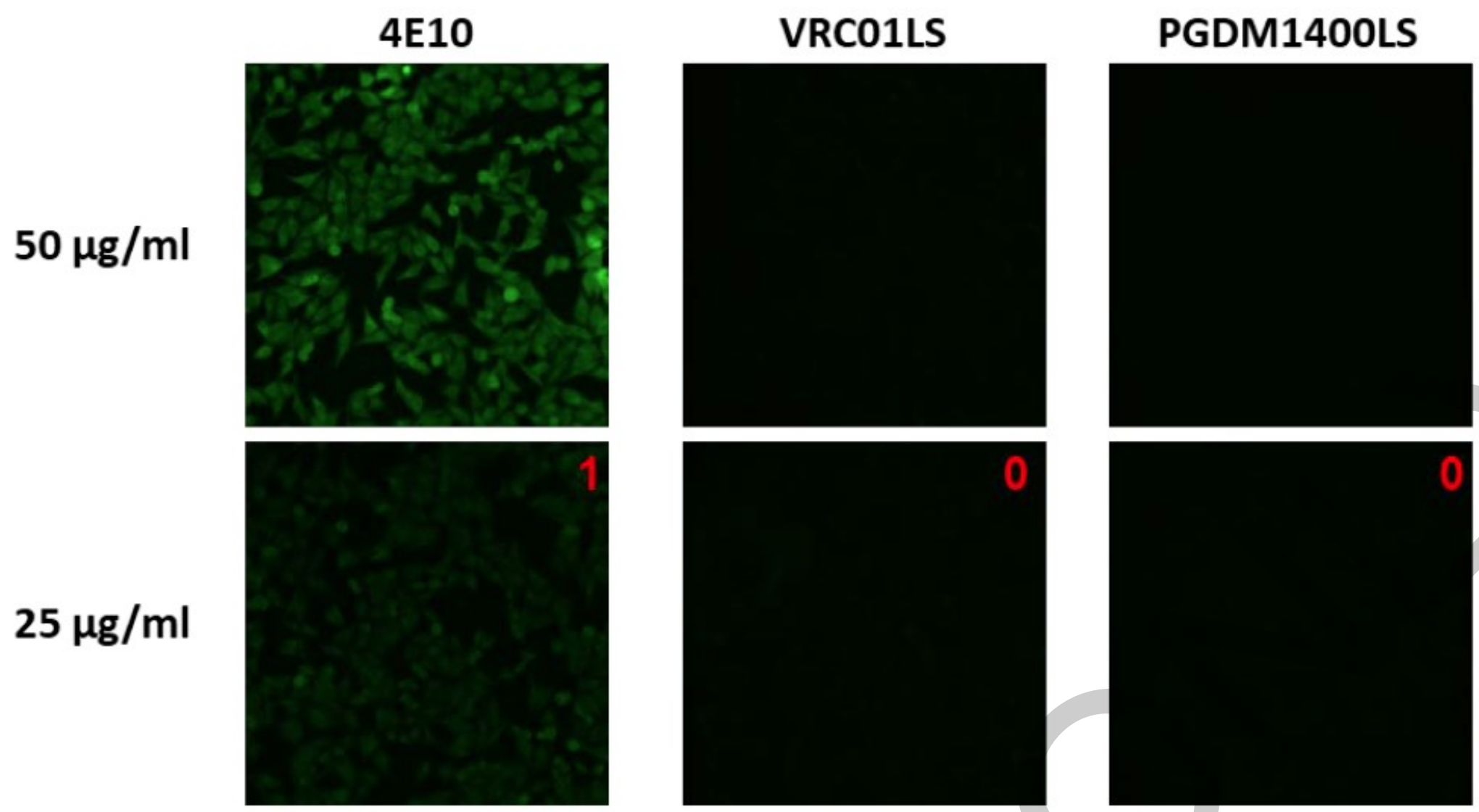

Figure 4-10 Representative images for the reactivity of 4E10, VRC01-LS and PGDM1400LS to HEp-2 cells by immunofluorescence staining using 25 or 50 mcg/mL of antibody. The tests on two separate days showed same results, thus only one was shown. Reactivity scores are shown as red inset numbers.

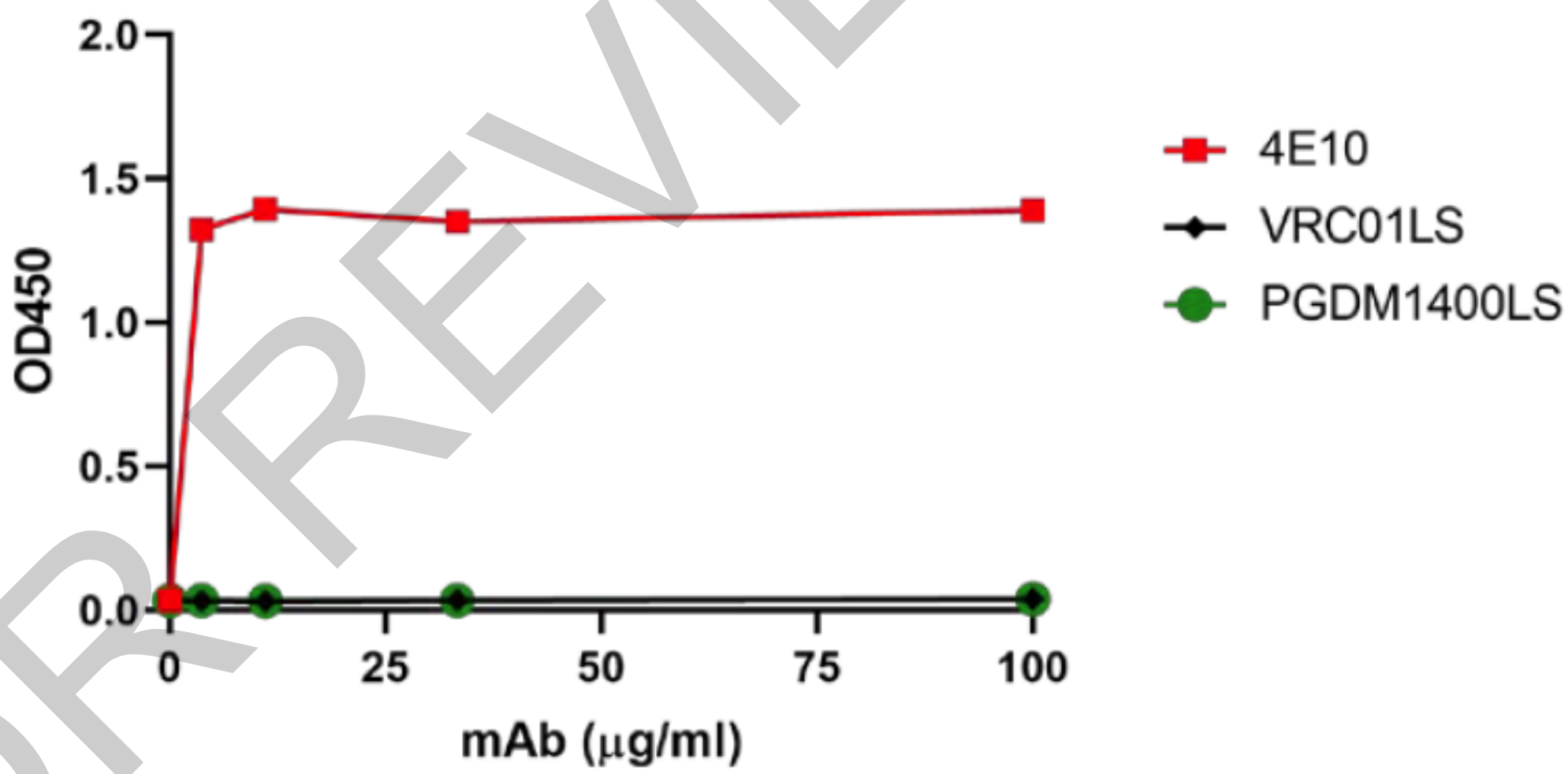

Figure 4-11 Antibody binding to cardiolipin by ELISA. 4E10, VRC01LS and PGDM1400LS were tested at 100, 33.3, 11.1 and 3.7 µg/mL and plotted with red squares, black diamonds and green dots, respectively.

4.7.3 Preclinical studies of PGT121.414.LS

Preclinical studies of PGT121.414.LS include: humanized FcRn mouse PK, NHP PK, repeat dose toxicity and Tissue Cross Reactivity as detailed below.

FOR REVIEW ONLY

4.7.3.1 Pharmacokinetic study of PGT121.414.LS in human neonatal Fc receptor (FcRn) transgenic mice

The VRC, NIAID, NIH evaluated the in vivo pharmacokinetic profile of PGT121.414.LS in three human FcRn transgenic mice (50-52). In this study, we tested PGT121.414.LS (lot #S-20190121-1) produced from a stably transfected CHO cell line. The antibody was given at a single bolus dose of 10 mg/kg via the intravenous route. The levels of antibody in the sera of these animals at various time points up to 28 days after administration were then quantitated by an anti-PGT121 idiotype based ELISA method. Figure 4-12 shows the sera levels of PGT121.414.LS in each animal, with levels maintained above 10 mcg/mL up to day 9 post infusion in all animals. After day 9, the sera levels of PGT121.414.LS steeply dropped to below the detection limit in 2 out of the 3 animals indicative of an anti-drug antibody (ADA) response against PGT121.414.LS in those animals. The third animal showed a longer persistence of the antibody in the sera with levels dropping below the detection limit at day 28 post infusion.

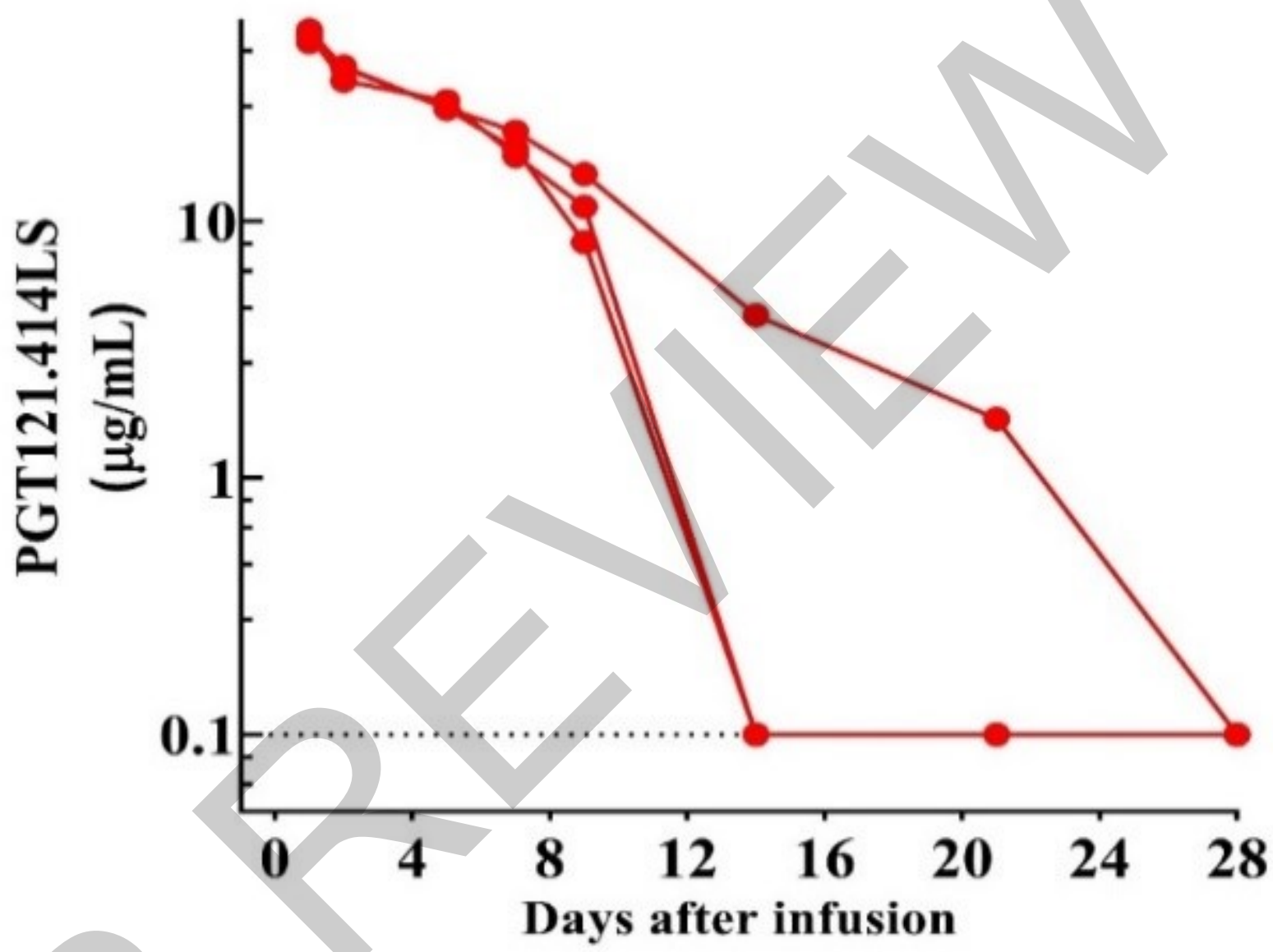

Figure 4-12 Sera levels of PGT121.414.LS antibody in three human FcRn transgenic mice administered 10 mg/kg of the antibody via the intravenous route.

The pharmacokinetic parameters were calculated using a non-compartment model in the WinNonLin software package and are presented in Table 4-4. The average half-life was calculated to be 3.61 days with a range of 2.53 to 4.59 days. The average AUC (Area Under the Curve) was calculated to be 332 Day mcg/mL with a range of 312 to 368 Day mcg/mL. The average clearance was calculated to be 15.13 mL/Day/kg with a range of 13.58 to 15.99 mL/Day/kg.

FOR REVIEW ONLY

Table 4-4 In vivo pharmacokinetic parameters for PGT121.414.LS in human FcRn transgenic mice

Antibody	Animal ID	Half-life Day	AUC Day*mcg/mL	Clearance mL/Day/kg
	2283	4.59	368	13.58
**PGT121.414LS**	2284	3.70	315	15.82
	2285	2.53	312	15.99
	Average	***3.61***	***332***	***15.13***
	Standard error	***0.46***	***14***	***0.60***

4.7.3.2 Pharmacokinetic study of PGT121.414.LS in rhesus macaques

The VRC evaluated the in vivo pharmacokinetic profile of PGT121.414.LS in four male and two female rhesus macaques. In this study, we tested PGT121.414.LS (lot #S-20190121-1) produced from a stably transfected CHO cell line. The antibody was given at a single bolus dose of 10 mg/kg via either the subcutaneous (n=3) or intravenous route (n=3). The levels of antibody in the sera of these animals at various time points up to 105 days after administration were then quantitated by an anti-PGT121 idiotype based ELISA method. Figure 4-13 shows the sera levels of PGT121.414.LS in each animal. The initial sera antibody levels were higher when the antibody was given IV compared to SC, but by day 2 the sera antibody levels were similar in all animals irrespective of route and followed similar distribution over time Also, sera antibody levels were maintained above 5mcg/mL for up to 105 days after dosing in all animals irrespective of route. In addition, none of the animals developed anti-drug antibody responses against PGT121.414.LS in this study.

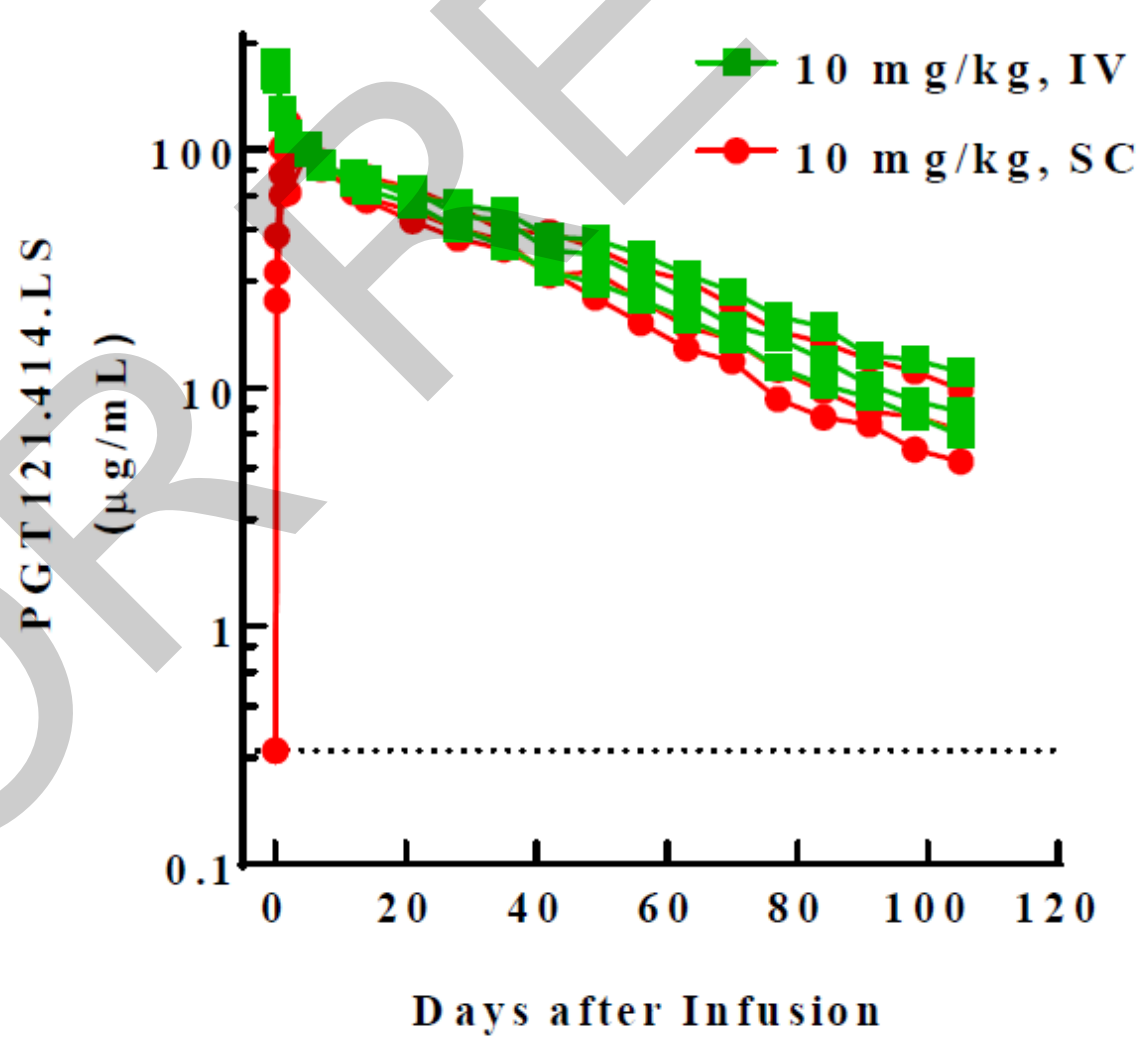

Figure 4-13 Sera levels of PGT121.414.LS antibody in six rhesus macaques administered at 10 mg/kg of the antibody via either the subcutaneous (SC) or intravenous (IV) route.

The pharmacokinetic parameters were calculated using a non-compartment model in the WinNonLin software package and are presented in Table 4-5. The average

FOR PREVIEW ONLY

half-life for PGT121.414.LS was 28.4 days for the IV route and 27.8 days for the SC route, which were very similar to each other, with a range of 26.6 days to 31.9 days. The AUC was slightly higher for the IV route (4710 day*mcg/mL) than for the SC route (3617 day*mcg/mL) due to the higher initial peak observed in the serum levels for the IV route compared to the SC route. The clearance was similar between the 2 routes (2.24 mL/day/kg for IV versus 2.61 mL/day/kg for SC).

Table 4-5 In vivo pharmacokinetic parameters for PGT121.414.LS in rhesus macaques

Route	Animal ID	Half-life (days)	AUC (day*mcg/mL)	Clearance (mL/day/kg)
**IV**	0DG	26.80	4221	2.22
**IV**	DGFH	26.65	3702	2.54
**IV**	DGKX	31.91	4586	1.96
	**Average**	**28.45**	**4170**	**2.24**
	**Standard error**	**1.73**	**257**	**0.17**
**SC**	09Z	30.13	4223	2.15
**SC**	DGDW	27.73	3353	2.78
**SC**	DGFK	25.53	3274	2.90
	**Average**	**27.80**	**3617**	**2.61**
	**Standard error**	**1.33**	**304**	**0.23**

Abbreviations: AUC, area under the curve; IV, intravenous; SC, subcutaneous.

4.7.3.3 GLP repeat dose IV or SC of PGT121.414.LS in rats

PGT121.414.LS was tested in a GLP-compliant study to determine the potential toxicity and toxicokinetics of the mAb in Sprague Dawley rats after three IV or SC dose administrations at 10-day intervals (on Days 1, 11, and 21) followed by a recovery period. No clinically significant findings were noted in the toxicology study. The study details and results are included in the IB.

4.7.3.4 PGT121.414.LS Tissue Cross Reactivity (TCR) study

PGT121.414.LS was tested in a GLP-compliant study to determine the potential cross reactivity of the mAb in human tissue cryosections. The TCR study was conducted using a panel of normal human tissue cryosections from 3 separate donors, according to recommendations in the Points to Consider in the Manufacture and Testing of Monoclonal Antibody Products for Human Use (CBER 1997) and consistent with ICH guidance S6(R1). No clinically significant findings were noted in the tissue cross reactivity study. The study details and results are included in the IB.

FOR REVIEW ONLY

4.7.3.5 4.5.2.5 PGT121.414.LS preclinical functional studies

PGT121.414.LS was recently assayed against a multiclade panel of 208 Env-pseudoviruses at the VRC (Table 4-6 below). As expected, it had less breadth than VRC07-523LS and VRC01 but was approximately 4-times more potent than VRC07-523LS and 10-times more potent than VRC01 against the viruses that were neutralized.

Table 4-6. Neutralization data of mAbs assayed against VITL/VRC Multiclade Panel

VITL/VRC Multiclade Panel

IC50	PGT121 .414 LS	PGT121	VRC01	VRC01.23	VRC07-523-LS	N6-LS	10-1074	PGDM 1400	10E8v4
# Viruses	208	208	208	208	208	208	208	208	208
% VS Neutralized									
IC50 <50ug/ml	58	65	90	96	96	98	63	80	98
IC50 <10ug/ml	55	64	89	96	96	97	63	79	98
IC50 <1.0ug/ml	50	56	72	94	92	95	60	75	71
IC50 <0.1ug/ml	44	44	17	73	53	63	42	60	15
For Sensitive Viruses Only:									
Median IC50	0.022	0.040	0.328	0.041	0.081	0.069	0.054	0.014	0.463
Geometric Mean	0.044	0.072	0.339	0.042	0.088	0.072	0.060	0.024	0.419
For All Viruses:									
Median IC50	0.904	0.352	0.392	0.042	0.086	0.071	0.204	0.043	0.468
Geometric Mean	0.868	0.691	0.548	0.055	0.116	0.081	0.724	0.107	0.470

IC80	PGT121 .414 LS	PGT121	VRC01	VRC01.23	VRC07-523-LS	N6-LS	10-1074	PGDM 1400	10E8v4
# Viruses	208	208	208	208	208	208	208	208	208
% VS Neutralized									
IC80 <50ug/ml	50	59	89	96	96	97	60	74	98
IC80 <10ug/ml	50	56	83	96	94	96	59	72	89
IC80 <1.0ug/ml	45	49	46	90	83	88	52	63	26
IC80 <0.1ug/ml	36	29	2	45	23	23	26	44	3
For Sensitive Viruses Only:									
Median IC80	0.051	0.099	0.959	0.107	0.238	0.221	0.126	0.047	2.31
Geometric Mean	0.076	0.154	1.06	0.120	0.257	0.231	0.157	0.069	1.91
For All Viruses:									
Median IC80	33.3	1.50	1.24	0.117	0.257	0.235	0.884	0.201	2.36
Geometric Mean	1.89	1.68	1.63	0.151	0.323	0.270	1.57	0.394	2.06

See IB for further details.

4.7.4 Preclinical studies of VRC07-523LS

VRC07-523LS has been assessed in several preclinical safety studies evaluating potential off-target binding, TCR, toxicity, and local tolerance.

VRC07-523LS was found to be 5- to 8-fold more potent than VRC01, with an IC50 < 50 mcg/mL against 96% of HIV-1 pseudoviruses representing the major circulating HIV-1 clades, and an IC50 < 1 mcg/mL against 92% of HIV-1 viruses tested. In addition, it displayed minimal levels of autoreactivity.

FOR REVIEW ONLY

In vivo proof-of-concept studies showed that VRC07-523LS is about 5-fold more potent than VRC01LS in Rhesus macaques and displays a longer half-life (9.8 days) than VRC07 (4.9 days) after a single dose of mAb at 10 mg/kg administered intravenously (11).

See the IB for further details.

4.8 Clinical trials of PGT121, PGT121.141LS, PGDM1400, VRC07-523LS alone and in combinations

Table 4-7 Summary of clinical studies

Study number/ status	Antibodies	Population(s)	Routes	ClinicalTrials.gov #	Protocol section
IAVI T001 completed	PGT121 FIH	HIV-<br>HIV+ on ART<br>HIV+ off ART	IV, SC	NCT02960581	4.8.1
IAVI T002 completed	PGDM1400 FIH, PGDM1400+PGT121, PGDM1400 + PGT121 + VRC07-523LS HIV+ off ART	HIV-<br>HIV+ off ART	IV	NCT03205917	4.8.2
IAVI T003 ongoing	PGT121 + VRC07-523LS, PGT121 + VRC07-523LS + PGDM1400, PGT121 + VRC07-523LS + PGDM1400 in HIV+ on ART	HIV-<br>HIV+ on ART	IV	NCT03721510	4.8.3
VRC605 Completed (53)	VRC07-523LS, FIH	HIV-	IV, SC	NCT03015181	4.8.4
HVTN 127/HPTN 087 ongoing	VRC07-523LS	HIV-	IV, SC	NCT03387150	4.8.5
HVTN 128 ongoing	VRC07-523LS	HIV-	IV	NCT03735849	4.8.6
HVTN 130/HPTN 089 ongoing	PGT121 + VRC07-523LS, PGDM1400 + VRC07-523LS, 10-1074 + VRC07-523LS, PGDM1400 + PGT121 + VRC07-523LS	HIV-	IV	NCT03928821	4.8.7
HVTN 136/HPTN 092 ongoing	PGT121.414LS FIH, PGT121.414LS + VRC07-523LS	HIV-	IV, SC	NCT04212091	4.8.8

FIH: first-in-human trial, IV:intravenous, SC: subcutaneous.

4.8.1 IAVI T001

IAVI T001 is a phase 1 randomized placebo-controlled clinical trial of the safety, pharmacokinetics, and antiviral activity of PGT121 in HIV-uninfected and HIV-infected adults. The study design is shown below in Table 4-8.

FOR REVIEW ONLY

Table 4-8: IAVI T001 study schema

	Group	Participants	Sub-Group	Regimen	N	Dose (mg/kg) - administration
Part 1[1]	1[3]	HIV-uninfected participants	1A	PGT121/Placebo	4/1 (6/2 if DLT[2])	3 IV
			1B	PGT121/Placebo	4/1 (6/2 if DLT)	10 IV
			1C	PGT121/Placebo	4/1 (6/2 if DLT)	30 IV
			1D	PGT121/Placebo	4/1 (6/2 if DLT)	3 SC
	2[3]	HIV-infected on ART, (<50 cp/ml)	2A	PGT121/Placebo	4/1 (6/2 if DLT[2])	3 IV
			2B	PGT121/Placebo	4/1 (6/2 if DLT)	10 IV
			2C	PGT121/Placebo	4/1 (6/2 if DLT)	30 IV
Safety Monitoring Committee Review[4]						
Part 2	3[5]	HIV-infected off ART (VL $2x10^3$ – $1x10^5$cp/ml)	3A	PGT121	6 (max 9)	30 IV
		HIV-Infected off ART (VL $1x10^2$ – $2x10^3$ cp/ml)	3D[6]	PGT121	6	30 IV

DLT, dose limiting toxicity; ART, antiretroviral therapy; cp, copies; ml, milliliter
Administration of PGT 121 will be by intravenous infusion (IV) or subcutaneous injection (SC)

The trial evaluated a single IV administration of PGT121 in healthy adults and in HIV-infected adults. Up to three escalating doses were tested, 3 mg/kg, 10 mg/kg and 30 mg/kg. Additionally, a single SC administration of PGT121 in healthy adults was tested at 3 mg/kg. A total of 48 volunteers were enrolled into the following dose subgroups:

- 1A (HIV-uninfected, 3 mg/kg IV, n=5),
- 1B (HIV-uninfected, 10 mg/kg IV, n=5),
- 1C (HIV-uninfected, 30 mg/kg IV, n=5),
- 1D (HIV-uninfected, 3 mg/kg SC, n=5);
- 2A (HIV-infected on ART, 3 mg/kg IV, n=5),
- 2B (HIV-infected on ART, 10 mg/kg IV, n=5),
- 2C (HIV-infected on ART, 30 mg/kg IV, n=5);
- 3A (HIV-infected not on ART, plasma HIV-RNA between 2,000 and 100,000 copies/mL,30 mg/kg IV, n=9), and
- 3B (HIV-infected not on ART, plasma HIV-RNA between 100 and 2,000 copies/mL, 30 mg/kg IV, n=4).

The most common *systemic* reactogenicity events reported based on the percentage of participants exhibiting the event across all groups were: headache 10/48 (20.8%), malaise 5/48 (10.4%), chills 4/48 (8.3%), nausea 1/48 (2%), arthralgia 1/48 (2%), and fever 1/48 (2%). The majority were mild (Grade 1); moderate (Grade 2) systemic reactions were reported for headache 4/48 (8.3%),

FOR REVIEW ONLY

malaise 2/48 (4.1%), nausea 1/48 (2%), and fever 1/48 (2%). No severe (Grade 3) events were reported during the study.

The most common *local* reactogenicity events reported based on the percentage of participants exhibiting the event across all groups were: tenderness 14/48 (29.1%), pain 7/48 (14.5%), erythema/skin discoloration 2/48 (4.1%) and swelling/hardening 2/48 (4.1%). Most were mild (Grade 1) l moderate (Grade 2) local reactions were reported for pain 1/48 (2%), tenderness 1/48 (2%) and erythema/skin discoloration 1/48 (2%). No severe (Grade 3) events were reported during the study.

Unsolicited adverse events (AEs) were adverse events reported in addition to the solicited reactogenicity events. A total of 39 unsolicited AEs were reported by the participants during the study, 34 non-serious adverse events in 21/48 (43.7%) participants who received investigational product and 5 non-serious adverse events in participants who had received placebo. The most common AEs reported based on percentage of participants exhibiting the event across all groups were: fatigue 4/48 (8.3%), headache 2/48 (4.1%), nasal congestion 2/48 (4.1%), upper respiratory tract infection 2/48 (4.1%), vessel puncture site bruising 2/48 (4.1%) and viral infection 2/48 (4.1%).

The majority were Grade 1 or 2 (mild or moderate), with one Grade 3 tonsillitis that was judged unrelated to study product. No adverse events were assessed as probably or definitely related to the study product. Two Grade 1 adverse events (headache and fatigue) and two Grade 2 adverse events (fatigue and gastroenteritis) were assessed as possibly related to study product.

One serious adverse event (SAE), a case of pre-patellar bursitis occurring 2 months after administration of study product, was assessed as not related. No deaths, potential immune mediating disease, or HIV infections were reported during the study. One pregnancy occurred during the study in an HIV-infected participant. The pregnancy was considered unremarkable and a healthy infant was delivered vis caesarian section at 36-weeks gestation.

The PGT121 elimination half-life in HIV-uninfected groups was 15.5 to 28.8 days and in HIV-infected groups was 8.2 to 28.9 days. The median AUC estimates from the HIV-uninfected groups were larger than the median AUC estimates from comparable HIV-infected groups (ie, 3 mg/kg HIV-uninfected vs. 3 mg/kg HIV-infected, etc.). In general, the median Cmax estimates from HIV-uninfected groups were similar to the median Cmax estimates from comparable HIV-infected groups.

4.8.2 IAVI T002

T002 is a phase 1, randomized, placebo-controlled clinical trial of the safety, pharmacokinetics, and antiviral activity of PGDM1400 alone or in combination with PGT121 in HIV-uninfected adults, and in combination with PGT121 and VRC07-523LS in HIV-infected adults. The study design is shown in Table 4-9.

FOR REVIEW ONLY

Table 4-9: IAVI T002 study schema

Participants	Group	Sub-Group	Regimen	N (Active/Placebo)	Dose (mg/kg)
HIV-uninfected participants	1	1A	PGDM1400 or Placebo	3/1	3 IV
HIV-uninfected participants	1	1B	PGDM1400 or Placebo	3/1	10 IV
HIV-uninfected participants	1	1C	PGDM1400 or Placebo	3/1	30 IV
HIV-uninfected participants	2	2A	PGDM1400 + PGT121 or Placebo	3/1	3 + 3 IV
HIV-uninfected participants	2	2B	PGDM1400 + PGT121 or Placebo	3/1	10 + 10 IV
HIV-uninfected participants	2	2C	PGDM1400 + PGT121 or Placebo	3/1	30 + 30 IV
HIV-infected off ART	3	3A	PGDM1400 + PGT121 + VRC07-523LS	4	20 + 20 + 20 IV
HIV-infected off ART	3	3B	PGDM1400 + PGT121	1	30 + 30 IV

As of January 2021, the study is completed and closed. A total of 29 individuals were enrolled, including 12 participants in group 1, 12 participants in group 2 and 4 participants in group 3. In groups 1 and 2 the MTD of 30 mg/kg for both products was confirmed. Overall, study product administration was safe and well-tolerated. Only grade 1 local reactogenicity was reported. Grade 1 and 2 systemic reactogenicity events included headache, myalgia, malaise and arthralgia. No Grade 3 or Grade 4 systemic symptoms were reported during the study. No AEs were assessed as probably or definitely related to the study product. No deaths, SAEs or potential immune-mediated disease adverse events were reported during the conduct of the study.

PGDM1400 concentrations have been measured in Groups 1A, 1B, and 1C (HIV-uninfected, 3, 10, and 30 mg/kg IV, respectively) and in Groups 2A, 2B and 2C (HIV-uninfected, 3, 10, and 30 mg/kg IV of each antibody, PGDM1400 and PGT121, respectively) by validated anti-idiotype BAMA-based PK assays through day 168 postinfusion (Figure 4-14). Among HIV-uninfected participants, the median (min, max) PGDM1400 elimination half-life estimate was 19.4 (15.3, 24.7) days, the population-level estimate of clearance (CL) was 0.191 (95% CI: 0.177, 0.206) L/day, the median (min, max) volume of distribution (Vd) was 5.67 (3.42, 8.04) L, and the median (min, max) dose- and weight-adjusted AUC was 371.74 (263.76, 507.73) day/L/Kg. There were no statistically significant differences in these parameters by PGDM1400 administration alone vs. co-administration with PGT121 (Group 1 vs. Group 2).

FOR REVIEW ONLY

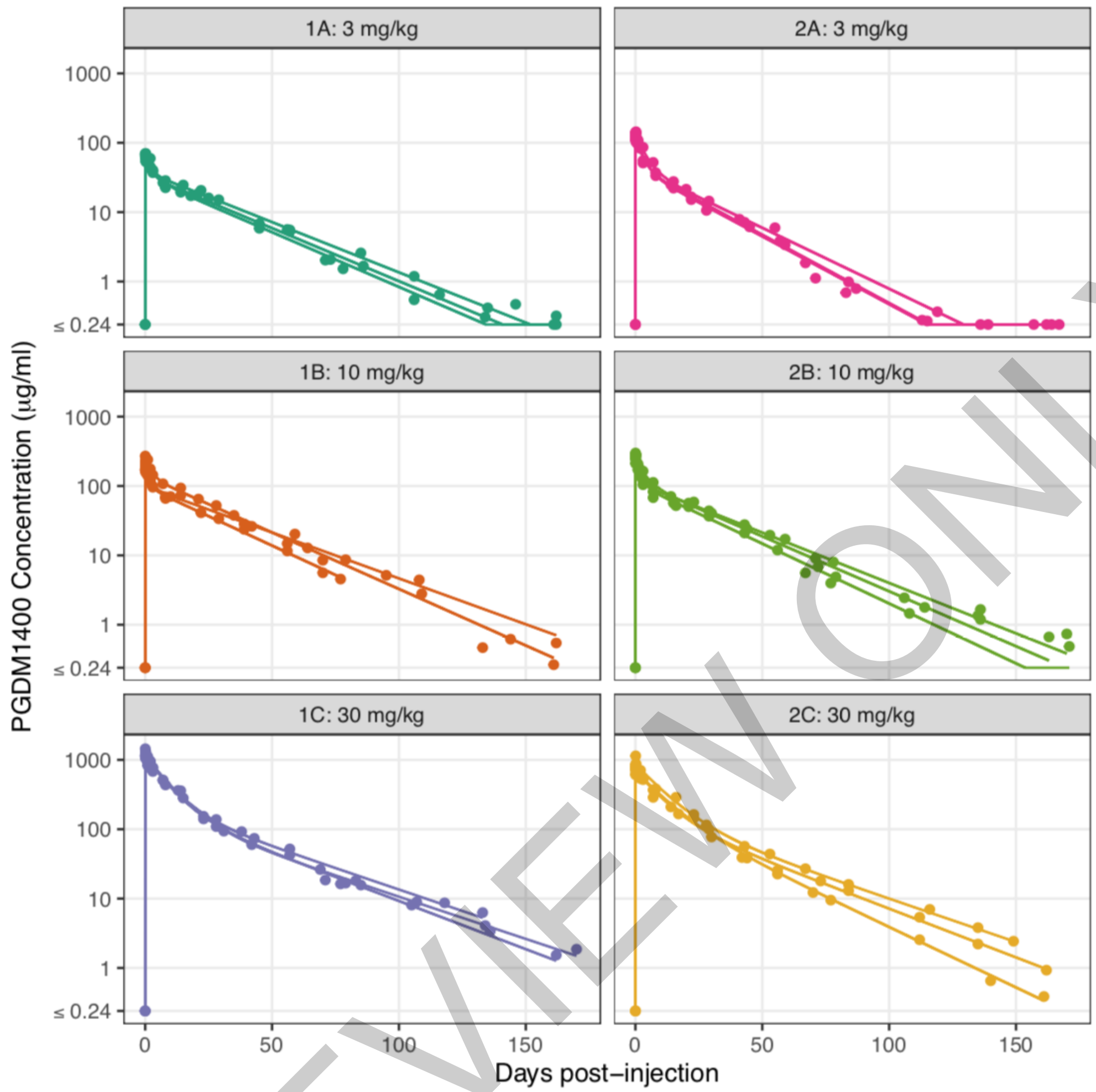

Figure 4-14 PGDM1400 binding antibody concentrations in Group 1A, 1B and 1C (HIV uninfected; 3, 10 and 30 mg/kg of PGDM1400), and in Group 2A, 2B and 2C (HIV uninfected; 3, 10 and 30 mg/kg of PGDM1400 and PGT121 each).

4.8.3 IAVI T003

IAVI T003 is a phase 1/2a, open-label clinical trial of the safety, tolerability, pharmacokinetics, and antiviral activity of PGT121, VRC07-523LS and PGDM1400 in HIV-uninfected and HIV-infected adults. The study design is shown below in Table 4-10.

FOR REVIEW ONLY

Table 4-10: IAVI T003 study schema

Group	Volunteer	Subgroup	Regimen	N	Dose (mg/kg)	Frequency	ATI
1	HIV-uninfected	1A	PGT121 +VRC07-523LS	3	30 + 30 IV	x1 (Day 0)	N/A
		PSRT review					
		1B	PGT121 +VRC07-523LS + PGDM1400	3	20 + 20 + 20 IV	x1 (Day 0)	N/A
SMC review							
2	HIV-infected on ART (VL <50 copies/mL)		PGT121 +VRC07-523LS + PGDM1400	12	20 + 20 + 20 IV	x3 (Days 0, 28, 56)<br>Optional: additional x3 (Days 84, 112, 140)	Yes
**Total**				**18**			

Notes: ART: Antiretroviral Therapy; ATI: Analytical Treatment Interruption (starting on Day 2 after participants complete their full course of ART for Day1 an after first IV infusion on Day 0); IV: intravenous; N/A: not applicable.

As of January 2021, Group 1A (PGT121 and VRC07-523LS, 30 mg/kg each, IV) and 1B (PGT121, VRC07-523LS and PGDM1400, 20 mg/kg each, IV) have been fully enrolled. Furthermore, PGT121, VRC07-523LS and PGDM1400, 20 mg/kg each, IV have been administered to 10 participants in Group 2, of which 1 participant has received 6 monthly infusions, 3 participants have received 3 monthly infusions and 6 participants have received between 1 and 3 monthly infusions, so far. There are still 2 open slots for enrollment. Study product administrations have been well tolerated overall, with no reported SAEs or potential immune-mediated disease.

4.8.4 VRC 605

VRC 605 is a phase 1, open-label, dose-escalation study of VRC07-523LS (NCT03015181) in healthy, HIV-uninfected adults to evaluate the safety and pharmacokinetics of 1 to 3 administrations of the antibody. The study is complete and results have been published (53) . The doses evaluated were a single administration of 1 mg/kg and 5 mg/kg IV and SC, 20 mg/kg and 40 mg/kg IV, and 3 administrations (q 12 weeks) of 5 mg/kg SC and 20 mg/kg IV VRC07-523LS (Table 4-11).

Study objectives included evaluating the safety and tolerability of the study regimen and the pharmacokinetics of each dose level, determining the presence or absence of detectable ADA to VRC07-523LS, and evaluating for evidence of functional activity of VRC07-523LS.

FOR REVIEW ONLY

Table 4-11 VRC 605 study schema

Group	Participants	Administration Schedule		
		Day 0	Week 12	Week 24
1	3	1 mg/kg IV		
2	3	5 mg/kg IV		
3	3	5 mg/kg SC		
4	3	20 mg/kg IV		
5	3	40 mg/kg IV		
6	5	5 mg/kg SC	5 mg/kg SC	5 mg/kg SC
7	5	20 mg/kg IV	20 mg/kg IV	20 mg/kg IV
Total	25			

IV = intravenous infusion
SC = subcutaneous injection

In VRC 605, a total of 41 administrations of VRC07-523LS occurred during the study. VRC07-523LS was safe and well tolerated, with no SAEs or dose-limiting toxicities. No anti-drug antibodies (ADA) were detected following IV or SC routes of administration (53).

PK analysis showed an elimination half-life of 38 days for IV groups and 33 days for subcutaneous groups (see Figure 4-15). VRC07-523LS containing sera showed equivalent or greater neutralization activity than sera from participants in previous trials receiving VRC01 or VRC01LS (53).

FOR REVIEW ONLY

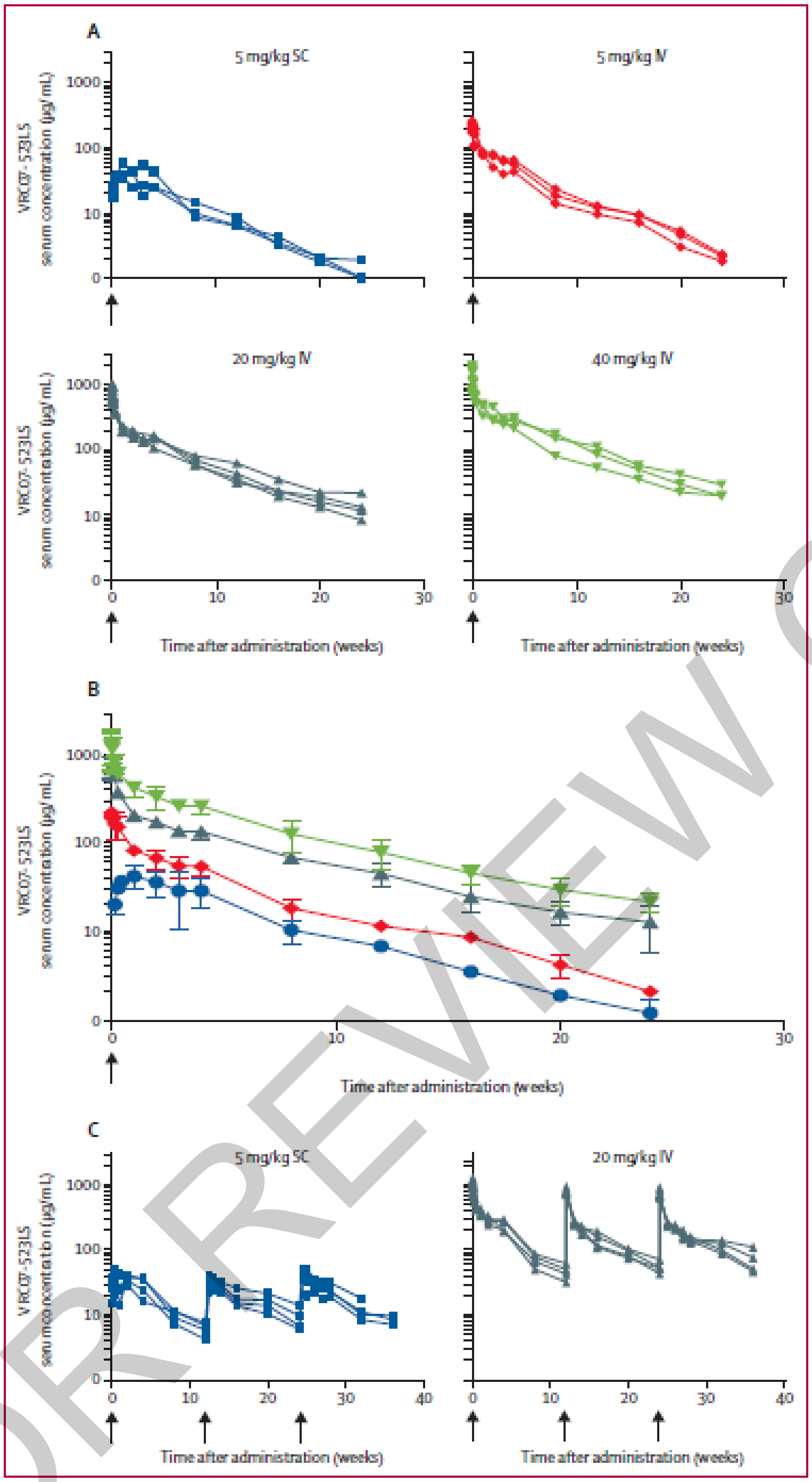

Figure 4-15 Serum concentrations of VRC07-523LS (A) Serum concentrations by individual participants, (B) Group geometric mean serum concentrations with error bars indicating the SD after a single administration, or (C) by individual participant after repeat administrations at weeks 0, 12, and 24. The upward pointing arrows indicate the time of VRC07-523LS administration. The participant in group 7 who only received a single dose of antibody was analyzed with group 4 participants. SC=subcutaneous. IV=intravenous. Figure from (53).

FOR REVIEW ONLY

4.8.5 HVTN 127/HPTN 087

HVTN 127/HPTN 087 (NCT03387150) is a randomized phase 1 clinical trial evaluating the safety and serum concentrations of VRC07-523LS administered in multiple doses via different routes to healthy, HIV-uninfected adults (Table 4-12).

Table 4-12 HVTN 127/HPTN 087 (Version 2.0) study schema

Group	N	Route	Dose	VRC07-523LS administration schedule				
				W0	W16	W32	W48	W64
1	20	IV	2.5 mg/kg	X	X	X	X	X
2	20	IV	5 mg/kg	X	X	X	X	X
3	20	IV	20 mg/kg	X	X	X	X	X
4	20	SC	2.5 mg/kg	X	X	X	X	X
5	20	SC	5 mg/kg	X	X	X	X	X
6	20	IM	2.5 mg/kg	X	X	X	X	X
	4		Placebo	X	X	X	X	X
**Total**	**124**							

IV = intravenous infusion
SC = subcutaneous injection
IM = intramuscular injection

HVTN 127/HPTN 087 enrolled 124 healthy, HIV-uninfected adult participants to receive five administrations of VRC07-523LS via IV, SC, or IM routes (4 IM participants receive placebo). A total of 440 administrations of VRC07-523LS via IV or SC occurred during the study; the IM group remains blinded and a total of 110 IM injections were administered.

The primary objectives of the study are to assess safety and tolerability of repeated IV, SC, or IM administrations of VRC07-523LS and to characterize serum concentration over time for different doses, schedules, and routes of administration. Additional objectives include building a population PK model of VRC07-523LS and determining whether ADAs emerge in response to repeated administrations of the antibody.

As of December 2020, three participants in Group 4 (2.5 mg/kg SC) experienced grade 3 SC injection site erythema or induration after their fifth administration including: (1) left arm erythema with a maximum erythema of 15x7 cm beginning two days after SC administration in the bilateral arms; (2) abdominal erythema and induration with a maximum erythema of 15x9.5 cm and a maximum induration of 14x8.5 cm beginning on the day of administration; and (3) abdominal erythema with a maximum measurement of 11x9 cm beginning one day after administration. One Grade 3 elevated alanine aminotransferase (ALT) deemed related to VRC07-523LS has been reported; the remaining AEs deemed

FOR REVIEW ONLY

related have been grade 1 (mild) or grade 2 (moderate). No related SAEs have been reported. Six mAb reactions have been reported in five participants, including: (1) grade 1 generalized pruritus in a participant in Group 2 (5 mg/kg IV) after her first infusion; (2) grade 1 generalized pruritus in a participant in Group 5 (5 mg/kg SC) after the second series of SC injections; (3) two infusion related reactions in a participant in Group 3 (20 mg/kg IV)- the first occurred after his first infusion and was grade 2 and characterized by chills, myalgia, arthralgia, malaise/fatigue, nausea and headache and the second occurred after his second infusion and was grade 1 and characterized by malaise/fatigue, chills and myalgia; (4) grade 1 infusion related reaction in a participant in Group 3 (20 mg/kg IV) and characterized by facial flushing, diaphoresis, myalgia, and arthralgia; and (5) grade 2 infusion related reaction in a participant in Group 3 (20 mg/kg IV) and characterized by facial flushing, asthenia, headache, pyrexia, pre-syncope, anxiety, fatigue/malaise, and warm sensation/sensation of feeling hot.

4.8.6 HVTN 128

HVTN 128 is a randomized, phase 1 clinical trial that evaluates the safety, tolerability and pharmacokinetics of VRC07-523LS in the sera and mucosae of healthy, HIV-uninfected adults (Table 4-13).

Table 4-13 HVTN 128 (Version 1.0) study schema

Group	N*	Route	VRC07-523LS Dose	Product administration schedule		
				D0	D112	D224
				W0	W16	W32
1	12	IV	10 mg/kg	X	X	X
2	12	IV	30 mg/kg	X	X	X
Total	24					

IV = intravenous infusion
N* = number of participants receiving at least one product infusion

HVTN 128 enrolled 24 participants to receive three IV infusions. A total of 66 administrations of VRC07-523LS occurred during the study. As of December 2020, no grade 3 or higher infusion site erythema or induration was reported; the only AEs deemed related to VRC07-523LS were mAb reactions detailed below. No related SAEs have been reported. Twelve mAb reactions have been reported in ten participants, including: (1) grade 1 facial flushing in a participant who enrolled into Group 2 (30 mg/kg); (2) grade 2 generalized pruritus in a participant in Group 2 (30 mg/kg); (3) grade 1 oropharyngeal itching in a participant who was randomized to Group 1 (10 mg/kg); (4) two grade 1 mAb reactions in a Group 1 (10 mg/kg) participant in whom the first was characterized by tachycardia, nausea, chills and headache after the first infusion and the second was characterized by nausea, vomiting, chills, muscle stiffness in her legs, arms

FOR REVIEW ONLY

and back after the third infusion; (5) grade 1 infusion related reaction characterized by non-exertional dyspnea and tachycardia in a participant in Group 2 (30 mg/kg); (6) grade 1 infusion related reaction characterized by headache, fatigue/malaise, and chills in a participant in Group 2 (30 mg/kg); (7) grade 1 infusion related reaction characterized by pyrexia, chills, fatigue/malaise, arthralgia, and myalgia in a participant in Group 2 (30 mg/kg); (8) two grade 1 mAb reactions in a in a participant in Group 1 (10 mg/kg), in whom the first was an infusion related reaction characterized by nausea, fatigue/malaise, chills, and myalgia that occurred after the first infusion, and the second reaction was chills after the third infusion; (9) grade 2 infusion related reaction characterized by hypotension, chills, nausea, fatigue/malaise, tachycardia and pyrexia in a participant in Group 2 (30 mg/kg); and (10) grade 1 infusion related reaction characterized by feeling cold, headache, mild rigors, headache, and generalized myalgia in a participant in Group 2 (30 mg/kg).

4.8.7 HVTN 130/HPTN 089

HVTN 130/HPTN 089 is a randomized, phase 1 clinical trial that will evaluate the safety, tolerability and serum concentrations of PGT121, PGDM1400, 10-1074 and VRC07-523LS when given sequentially in single and multiple doses via IV administration to healthy, HIV-uninfected adults (Table 4-14).

Table 4-14 HVTN 130/HPTN 089 (Version 1.0) study schema

Study arm	N	Dose	Route	M0	M4
Group 1	6	20+20 mg/kg	IV	PGT121 VRC07-523LS	—
Group 2	6	20+20 mg/kg	IV	PGDM1400 VRC07-523LS	—
Group 3	6	20+20 mg/kg	IV	10-1074 VRC07-523LS	—
Group 4*	9	20+20+20 mg/kg	IV	PGDM1400 PGT121 VRC07-523LS	PGDM1400 PGT121 VRC07-523LS
Total	27				

IV = intravenous infusion. The mAbs are infused sequentially in the order shown.
* Opening enrollment in Group 4 follows review of safety data for all participants in Groups 1-3.

A total of 33 administrations of PGT121, VRC07-523LS, 10-1074 and PGDM1400 occurred during the study. As of December 2020, no grade 3 or higher infusion site erythema or induration was reported; no grade 3 AEs deemed related to PGT121, PGDM1400, 10-1074 or VRC07 523LS were reported; no related SAEs have been reported. One mAb reaction was reported: a grade 1 infusion related reaction in a participant in Group 2 (20+20 mg/kg) characterized by chills, muscle pain in his upper back, joint pain in his wrists and headache.

FOR REVIEW ONLY

4.8.8 HVTN 136/HPTN 092

HVTN 136/HPTN 092 is the first clinical study of the PGT121.414.LS mAb. This phase 1, dose-escalation, open-label clinical trial is aiming to examine the safety, tolerability, dose, and pharmacokinetics of PGT121.414.LS with and without VRC07-523LS, a CD4 binding site mAb (see Table 4-15). The hypothesis is that PGT121.414.LS alone and paired with VRC07-523LS will be safe for administration to healthy adults by both the IV and SC routes.

In Part A of the study, PGT121.414.LS will be administered via IV infusion at 3, 10, or 30 mg/kg (Groups 1-3) or via SC infusion at 5 mg/kg (Group 4). Each group in Part A will have 3 participants. At each of 3 visits in Part B of the study, participants will receive consecutive administration of PGT121.414.LS followed by VRC07-523LS, at 20 mg/kg IV each per dose (Group 5) or 5 mg/kg SC each per dose (Group 6). Each group in Part B will have 10 participants, yielding a total sample size for Parts A and B of 32. Participants will be followed for 32 weeks after the last study product administration via IV infusion and 24 weeks after the last study product administration via SC infusion.

Table 4-15 HVTN 136/HPTN 092 schema

Study arm	Number	Dose	Route	Month 0 (Day 0)	Month 4 (Day 112)	Month 8 (Day 224)
**Part A**						
Group 1*	3	3 mg/kg	IV	PGT121.414.LS	—	—
Group 2[a*]	3	10 mg/kg	IV	PGT121.414.LS	—	—
Group 3[b*]	3	30 mg/kg	IV	PGT121.414.LS	—	—
Group 4[b*]	3	5 mg/kg	SC	PGT121.414.LS	—	—
**Part B**						
Group 5[c]	10	20 mg/kg + 20 mg/kg	IV	PGT121.414.LS + VRC07-523LS	PGT121.414.LS + VRC07-523LS	PGT121.414.LS + VRC07-523LS
Group 6[c]	10	5 mg/kg + 5 mg/kg	SC	PGT121.414.LS + VRC07-523LS	PGT121.414.LS + VRC07-523LS	PGT121.414.LS + VRC07-523LS
Total	32					

IV = intravenous infusion; SC = subcutaneous infusion.

A total of 9 administrations of PGT121.414.LS in Part A and no administrations of PGT121.414.LS with VRC07-523LS in Part B occurred during the study. As of May 2021, no Grade 3 or higher infusion site erythema or induration was reported; no AEs deemed related to PGT121.414.LS or VRC07-523LS were

FOR REVIEW ONLY

reported. No related SAEs have been reported. No mAb reactions have been reported. As this study is ongoing and directly relevant to this study, the protocol team will be informed of any additional, relevant HVTN 136/HPTN 092 data as it becomes available.

4.9 Potential risks of study products and administration

General Risks of Monoclonal Antibodies (mAbs): Overall, the side effects of mAbs are generally mild and can include fever, flushing, chills, rigors, nausea, vomiting, diarrhea, pain, pruritus, rash, urticaria, angioedema, headache, dizziness, shortness of breath, bronchospasm, tachycardia, hypotension, hypertension, and chest pain. There can also be a risk of infection from mAbs targeting human cytokines or human cell antigens, but this is not expected with a mAb targeting a viral antigen (54). Other uncommon potential side effects of mAbs include thrombocytopenia, autoimmune diseases, cancer, dermatitis, and cardiotoxicity (54).

Additional reactions such as tumor lysis syndrome and cytokine release syndrome have been previously described with chimeric and humanized Abs, usually with mAbs targeting human antigens. Cytokine release syndrome has been described with human mAbs targeting lymphocyte cell-surface antigens. Serious allergic reactions such as anaphylaxis, angioedema, bronchospasm, hypotension, and hypoxia are rare and often associated with mAbs targeting human proteins or with non-human mAbs. These mAb-related events typically occur within the first 24 hours of administration, though have not been observed with anti-HIV mAbs to date. Cytokine release syndrome typically occurs within the first few hours of administration, and usually with the first administration when the largest number of target cells expressing antigen are present. Reactions related to the rate of infusion have been described for several FDA-licensed mAbs. Cytokine release syndrome can be effectively managed by temporarily holding the administration, administering anti-histamines, and restarting the IV infusion at a slower rate (55).

More delayed allergic reactions may occur approximately 24 hours after administration, though can occur several days to a few weeks after administration, and can include serum sickness, which is characterized by urticaria, fever, lymphadenopathy, and arthralgia. This reaction is more likely to occur with chimeric Abs, and has not been observed with fully human mAbs.

Other potential side effects of mAbs include thrombocytopenia, autoimmune diseases, cancer, dermatitis, and cardiotoxicity (54).

Risk of Monoclonal Antibody-Associated Reactivity: There is a possibility that receipt of the study products will cause a reactive result on some currently available HIV test kits, especially if testing occurs close to study product administration timepoints (see Section 9.5.1).

FOR REVIEW ONLY

Risks of Blood Drawing: Blood drawing may cause pain and bruising and may, infrequently, cause a feeling of lightheadedness or fainting. Rarely, it may cause infection at the site where the blood is taken. Problems from use of an IV for blood drawing are generally mild and may include pain, bruising, minor swelling or bleeding at the IV site and, rarely, infection, vein inflammation (phlebitis), or blood clots. Blood drawing may also cause anemia.

Risks of IV Infusion: The placement of an IV catheter can allow for the development of bacteremia because of the contact between the catheter and unsterile skin during insertion. Risk of infection from IV infusion will be minimized through careful decontamination of skin prior to catheter placement and through the use of infection control practices during infusion. The risk of product contamination will be minimized through the use of aseptic technique during product preparation and administration.

Risks of SC Administration: SC administration can result in pain, nodule formation, and possible infection. The risk of infection from SC administration will be minimized through skin decontamination of the skin prior to needle insertion. The risk of product contamination will be the same as for IV administration and will be minimized through the use of aseptic technique during product preparation and administration.

Risks of study products:

Experience is accruing with PGT121.414.LS and its parental PGT121 mAb, PGDM1400 (the parental mAb for PGDM 1400LS) and VRC07523-LS mAbs alone and in the combinations mentioned above (Section 4.8). This study evaluating PGDM1400LS alone and in combination with PGT121.414.LS and VRC07-523LS will be a first-in-human for the LS-modified form of PGDM1400. Human experience with PGDM1400 is limited to the IAVI T002 and T003 and HVTN 130/HPTN 089 clinical trials.

As of January 2021, the IAVI T003 and HVTN 130/HPTN 089 clinical trials' accrual is ongoing, and there have been no related SAEs. To date, there have been no study safety pauses for AEs and product administrations have been generally well tolerated.

To date, the clinical trial safety experience with VRC01-class mAbs has been reassuring. In HVTN 104, IV administration of VRC01 was generally well-tolerated with mild pain and/or tenderness commonly reported at the site of the IV infusion. Mild-to-moderate systemic reactogenicity symptoms were reported by VRC01 recipients following at least 1 of the infusions, but there was no clear relationship with frequency or severity to the dose of VRC01 (39). SC administration of VRC01 was generally well-tolerated and associated with mild to moderate local pain and/or tenderness, but there was no difference in the frequency or severity between VRC01 recipients and placebo recipients. Erythema and/or induration at the SC injection sites were generally less than 25

FOR REVIEW ONLY

cm^2 whether VRC01 or placebo was injected (39). No hypersensitivity reactions or cytokine release syndrome symptoms were reported in HVTN 104 (39).

The efficacy trials HVTN 704/HPTN 085 and HVTN 703/HPTN 081 have accumulated significant additional VRC01 clinical experience. More than 40,000 infusions of 10 mg/kg and 30 mg/kg VRC01 have been given to more than 4,500 HIV-uninfected adults across both trials. While final safety analysis is ongoing, the safety profile is reassuring, thus far. In the combined trials, 40,642 IV infusions were administered and 209 AEs deemed related to study product were reported. The mean and median onset of these AEs was Day 0, the day of infusion. Of these AEs deemed related to study product, all ≥ grade 3 AEs and 95% of AEs of any grade were reported within three days post infusion. VRC01 was well-tolerated, with low rates of infusion-related reactions (IRRs): 1.7% of participants in HVTN 704/HPTN 085 and 4.8% of participants in HVTN 703/HPTN 081 experienced IRRs to VRC01/placebo. In the combined trials, 132 out of 160 IRRs developed in VRC01 recipients. IRR were typically mild or moderate (96.2% were deemed mild or moderate and 3.8% were deemed severe), successfully managed at the clinical research site, and resolved without sequelae.

Early-phase human experience with administration of VRC07-523LS has been reassuring in the VRC605 and HVTN 127/HPTN 087 trials (see Sections 4.8.4 and 4.8.5). The majority of participants have reported no local or systemic Solicited AEs and no Unsolicited AEs. Severe reactions associated with mAb administration, such as acute anaphylaxis, serum sickness, anti-drug antibodies, and increased risk of infections have not been observed to date in trials of VRC01-class mAbs, nor in trials of PGT121 or PGDM1400.

Risks of interference with common HIV tests:

An anti-HIV mAb is not likely to directly reduce or inhibit the assays used to detect HIV-1 infection.

FOR REVIEW ONLY

5 Objectives and endpoints

5.1 Primary objectives and endpoints

Primary objective 1:

To evaluate the safety and tolerability of PGDM1400LS when administered via intravenous (IV) or subcutaneous (SC) routes (Part A) and of PGDM1400LS, VRC07-523LS and PGT121.414.LS when administered in sequence IV or SC (Part B)

Primary endpoints 1:

- Local and systemic Solicited AEs, laboratory measures of safety, Unsolicited AEs, and SAEs
- Early discontinuation of administration and reason(s) for discontinuation and early study termination

Primary objective 2:

To evaluate the serum concentrations and pharmacokinetics of PGDM1400LS after a single administration (Part A) and of PGDM1400LS, VRC07-523LS, and PGT121.414.LS after each three-mAb administration (Part B)

Primary endpoint 2:

Serum concentrations of PGDM1400LS, VRC07-523LS and PGT121.414.LS at prespecified timepoints among participants who received all scheduled product administrations

Primary objective 3:

To evaluate the individual mAb-specific serum neutralizing activity after single product administration of PGDM1400LS (Part A) and after each three-mAb administration of PGDM1400LS, VRC07-523LS and PGT121.414.LS (Part B)

Primary endpoint 3:

Magnitude and breadth of neutralizing activity measured with Env pseudotyped viruses specific for either PGDM1400LS, VRC07-523LS or PGT121.414LS in TZM-bl cells at prespecified timepoints among participants who received all scheduled product administrations

FOR REVIEW ONLY

5.2 Secondary objectives and endpoints

Secondary objective 1:

To correlate serum concentrations of PGDM1400LS, VRC07-523LS and PGT121.414.LS with corresponding virus neutralization titers in serum

Secondary endpoints 1:

- Serum concentrations of PGDM1400LS, VRC07-523LS and PGT121.414.LS at prespecified timepoints for all participants in all groups regardless of how many product administrations and how much product they received

- Magnitude of serum neutralizing activity measured with mAb-specific Env-pseudotyped viruses in TZM-bl cells at prespecified timepoints for all participants in all groups regardless of how many product administrations and how much product they received

Secondary objective 2:

To determine whether the mAbs maintain their expected combined magnitude and breadth of serum neutralizing activity after each PGDM1400LS, VRC07-523LS and PGT121.414.LS three-mAb administration (Part B) as predicted by the known magnitude and breadth of neutralization of the corresponding mAb combinations as non-infused clinical products

Secondary endpoint 2:

Magnitude of neutralizing activity against a panel of Env-pseudotyped reference viruses that are sensitive to all three bnAbs in TZM-bl cells at selected timepoints for all participants in all groups regardless of how many product administrations and how much product they received

Secondary objective 3:

To determine whether anti-drug antibodies (ADA) are present

Secondary endpoint 3:

ADA titers in each group measured at prespecified timepoints for all participants in all groups regardless of how many product administrations and how much product they received

Secondary objective 4:

To evaluate the impact of body weight and other baseline covariates on the pharmacokinetics of PGDM1400LS, VRC07-523LS and PGT121.414.LS in both the weight-based and fixed-dose groups.

FOR REVIEW ONLY

Secondary endpoint 4:

Serum concentrations of PGDM1400LS, VRC07-523LS and PGT121.414.LS at prespecified timepoints

5.3 Exploratory objectives

Exploratory objective 1:

To determine whether any confirmed positive ADA samples have functional activity that impacts the neutralizing activity of PGDM1400LS, VRC07-523LS and PGT121.414.LS

Exploratory objective 2:

To further evaluate non-neutralizing anti-viral activities, additional assays (eg, ADCC, ADCP, virion capture) may be performed for activities that the PGDM1400LS, VRC07-523LS and PGT121.414.LS are shown to exhibit in vitro

Exploratory objective 3:

To conduct analyses related to predicting serum neutralization over time against a set of potentially exposing viruses in a future efficacy trial for ranking and down-selecting bnAb regimens

Exploratory objective 4:

To conduct analyses related to furthering the understanding of HIV, passive immunity, immunology, vaccines, and clinical trial conduct.

FOR REVIEW ONLY

6 Statistical considerations

6.1 Accrual and sample size calculations

Recruitment will target enrolling about 95 healthy, HIV-uninfected adult participants.

In Part A of the study, PGDM1400LS will be administered as a single IV infusion at 5, 20, or 40 mg/kg (Groups 1, 2, and 4) or as a single SC dose at 20 or 40 mg/kg (Groups 3 and 5) with n=3 participants per group. The trial will begin with enrollment in Group 1 only. Groups 2 and 3, and Groups 4 and 5, will be enrolled stepwise by two groups and randomized in a 1:1 ratio, respectively. Additional participants may be enrolled in each group to ensure the availability of early post-infusion safety data from at least 3 participants in each group.

In Part B of the study, participants will receive two doses of PGDM1400LS + VRC07-523LS + PGT121.414.LS at 20 or 40 mg/kg IV each per dose (Group 6 and 10) or 20 mg/kg SC each per dose (Group 7), or a fixed dose of 4.2 g IV (Group 8) or SC (Group 9). Groups 6, 7, 8, and 9 will begin enrollment simultaneously. Additional participants may be enrolled in each group to ensure the availability of early post-infusion safety data from at least 8 participants in Groups 6 through 9 together; or at least 3 participants in Group 10. All groups 1-10 are open-label. See also in Section 11.3.

To ensure balance across both sexes assigned at birth, participants of each sex assigned at birth should constitute approximately 40-60% of those enrolled in the trial (Part A and Part B combined). The study team will work closely with the sites to monitor enrollment progress to ensure appropriate balance. Since enrollment is concurrent with receiving the first study product administration, all participants will provide some safety data. However, for mAb concentrations and anti-viral functional activities, it is possible that data may be missing for various reasons, such as participants terminating from the study early, problems in shipping specimens, or high assay background. For this reason, the sample size calculations in Section 6.1.2 account for 20% enrolled participants having missing data for the primary lab endpoint at a given timepoint. As a reference, immunogenicity data from 17 phase 1 and 2 phase 2a HVTN vaccine trials, which began enrolling after June 2005 (data as of September 2014), indicate that 17% is a reasonable estimate for the rate of missing data at a given timepoint. In HVTN 104 (phase 1 trial of VRC01), approximately 15% of mAb concentration data were missing at the primary timepoints.

6.1.1 Sample size calculations for safety

The goal of the safety evaluation for this study is to identify safety concerns associated with product administration. The ability of the study to detect serious adverse events (SAEs) (see Section 11) can be expressed by the true event rate above which at least 1 SAE would likely be observed and the true event rate

FOR REVIEW ONLY

below which no events would likely be observed. Specifically, for each treatment group of size n = 3 in Part A, there is at least a 90% or more chance of observing at least 1 event if the true rate of such an event is 53.6% or more; and there is at least a 90% or more chance of observing no events if the true rate is 3.4% or less. For IV administration Groups 1, 2 and 4 combined n = 9 in Part A, there is at least a 90% or more chance of observing at least 1 event if the true rate of such an event is 22.6% or more; and there is at least a 90% or more chance of observing no events if the true rate is 1.2% or less. For SC administration Groups 3 and 5 combined n = 6 in Part A, there is at least a 90% or more chance of observing at least 1 event if the true rate of such an event is 31.9% or more; and there is at least a 90% or more chance of observing no events if the true rate is 1.7% or less. For all Groups 1-5 combined n = 15 in Part A, there is at least a 90% or more chance of observing at least 1 event if the true rate of such an event is 14.2% or more; and there is at least a 90% or more chance of observing no events if the true rate is 0.7% or less. For each group of size n=16 in Part B, there is 90% or more chance of observing at least 1 event if the true rate of such an event is 13.4% or more; and there is 90% or more chance of observing no events if the true rate is 0.7% or less. For IV administration Groups 6, 8 and 10 combined with n=48 in Part B, there is 90% or more chance of observing at least 1 event if the true rate of such an event is 4.7% or more; and there is 90% or more chance of observing no events if the true rate is 0.2% or less. For SC administration Groups 7 and 9 combined with n=32 in Part B, there is 90% or more chance of observing at least 1 event if the true rate of such an event is 6.9% or more; and there is 90% or more chance of observing no events if the true rate is 0.3% or less. For all Groups 6-10 combined n = 80 in Part B, there is at least a 90% or more chance of observing at least 1 event if the true rate of such an event is 2.8% or more; and there is at least a 90% or more chance of observing no events if the true rate is 0.1% or less. As a reference, in HVTN vaccine trials from April 2008 through March 2018, about 1.7% of participants who received placebos experienced an SAE.

Binomial probabilities of observing 0, 1 or more, and 2 or more events among arms of size 3 and 16 are presented in Table 6-1 for a range of possible true adverse event rates. These calculations provide a more complete picture of the sensitivity of the study design to identify potential safety problems with PGDM1400LS, VRC07-52310LS and PGT121.414.LS.

FOR REVIEW ONLY

Table 6-1 Probability of observing 0 events, 1 or more events, and 2 or more events, among groups of size 3, 9, 10, 15, 20, and 30 for different true event rates

Group Size	True event rate (%)	$Pr(0/n_1)$	$Pr(1+/n_1)$	$Pr(2+/n_1)$
3	1	0.97	0.03	<0.01
	4	0.88	0.12	<0.01
	10	0.73	0.27	0.03
	20	0.51	0.49	0.1
	30	0.34	0.66	0.22
	40	0.22	0.78	0.35
9	1	0.91	0.09	<0.01
	4	0.69	0.31	0.05
	10	0.39	0.61	0.23
	20	0.13	0.87	0.56
	30	0.04	0.96	0.8
	40	0.01	0.99	0.93
15	1	0.86	0.14	<0.01
	4	0.54	0.46	0.12
	10	0.21	0.79	0.45
	20	0.04	0.96	0.83
	30	<0.01	>0.99	0.96
	40	<0.01	>0.99	>0.99
16	1	0.85	0.15	0.01
	4	0.52	0.48	0.13
	10	0.19	0.81	0.49
	20	0.03	0.97	0.86
	30	<0.01	>0.99	0.97
	40	<0.01	>0.99	>0.99
48	1	0.62	0.38	0.08
	4	0.14	0.86	0.58
	10	<0.01	>0.99	0.96
	20	<0.01	>0.99	>0.99
	30	<0.01	>0.99	>0.99
	40	<0.01	>0.99	>0.99
80	1	0.45	0.55	0.19
	4	0.04	0.96	0.83
	10	<0.01	>0.99	>0.99
	20	<0.01	>0.99	>0.99
	30	<0.01	>0.99	>0.99
	40	<0.01	>0.99	>0.99
95	1	0.38	0.62	0.25
	4	0.02	0.98	0.9
	10	<0.01	>0.99	>0.99
	20	<0.01	>0.99	>0.99
	30	<0.01	>0.99	>0.99
	40	<0.01	>0.99	>0.99

FOR REVIEW ONLY

An alternative way of describing the statistical properties of the study design is in terms of the 95% confidence interval for the true rate of an adverse event based on the observed data. Table 6-2 shows the 2-sided 95% confidence intervals for the probability of an event based on a particular observed rate. Calculations are done using the score test method (56). If none of the 15 or 80 participants receiving an antibody treatment regimen in Part A or Part B experience a safety event, the 95% 2-sided upper confidence bound for the true rate of such events in the total treated population is 20.4% or 4.6%. For each individual antibody treatment arm n = 3 or 16 in Part A or Part B, the 2-sided upper confidence bound for this rate is 56.1% and 19.4%, respectively.

Table 6-2 Two-sided 95% confidence intervals for the probability of observing a safety event based on observing a particular rate of safety endpoints for arms of size 3, 15, 16, 80 and 95.

Observed event rate	95% Confidence interval (%)
0/3	(0.0, 56.1)
1/3	(6.2, 79.2)
2/3	(20.8, 93.8)
0/15	(0.0, 20.4)
1/15	(1.2, 29.8)
2/15	(3.7, 37.9)
0/16	(0.0, 19.4)
1/16	(1.1, 28.3)
2/16	(3.5, 36.0)
0/80	(0.0, 4.6)
1/80	(0.2, 6.8)
2/80	(0.7, 8.7)
0/95	(0.0, 3.9)
1/95	(0.2, 5.7)
2/95	(0.6, 7.3)

6.1.2 Sample size calculations for serum mAb concentrations

Primary Objective 2 of this study is to evaluate serum concentrations of PGDM1400LS, VRC07-523LS and PGT121.414.LS at several timepoints (ie, pharmacokinetics) following single or multiple IV or SC administrations. This objective is descriptive in nature, and will be accomplished by estimating the mean serum concentration of each mAb within each active arm at specific timepoints following product administration. The precision with which a true mean concentration can be estimated from observed data depends on the standard

FOR REVIEW ONLY

deviation (SD) of the measurements and the sample size. Table 6-3 displays two-sided 95% confidence intervals for the mean drug concentration for several values of the observed average drug concentration. The construction of these confidence intervals assumed sample sizes of n = 12 per arm, reflecting an attrition rate of 20% compared to a planned treatment group size of 16 participants. The calculations assumed that log-transformed serum concentrations are approximately normally distributed. To account for the small sample sizes, a *t*-distribution was used to construct CIs. For instance, with an observed mean $\log_e$ serum level of $\log_e$(10 mcg/mL) and assuming a standard deviation of 0.5 for their log-transformed values, a two-sided 95% confidence interval for the true mean drug concentration level is (7.3, 13.7) (in mcg/mL) with an effective sample size of 12 participants. Of note, in the HVTN104 trial of VRC01, SD smaller than 1.0 for the $\log_e$ -transformed serum concentrations of VRC01 were repeatedly observed over time following either IV infusions or SC injections of the study product (39, 47).

Table 6-3 Two-sided 95% confidence intervals based on observing a particular average $\log_e$ **mAb concentration in participants in any of the active arms, taking 20% attrition into consideration (n= 12)**

Observed average $\log_e$-concentration ($\log_e$ mcg/mL)	SD of $\log_e$-concentration ($\log_e$ mcg/mL)	95% confidence interval (mcg/mL)
		n = 12
$\log_e$(0.5)	0.5	(0.4, 0.7)
$\log_e$(1)		(0.7, 1.4)
$\log_e$(5)		(3.6, 6.9)
$\log_e$(10)		(7.3, 13.7)
$\log_e$(50)		(36.4, 68.7)
$\log_e$(100)		(72.8, 137.4)
$\log_e$(250)		(182.0, 343.5)
$\log_e$(500)		(363.9, 687.0)
$\log_e$(1000)		(727.8, 1373.9)
$\log_e$(0.5)	1	(0.3, 0.9)
$\log_e$(1)		(0.5, 1.9)
$\log_e$(5)		(2.6, 9.4)
$\log_e$(10)		(5.3, 18.9)
$\log_e$(50)		(26.5, 94.4)
$\log_e$(100)		(53.0, 188.8)
$\log_e$(250)		(132.4, 471.9)
$\log_e$(500)		(264.9, 943.9)
$\log_e$(1000)		(529.7, 1887.7)

6.1.3 Sample size calculations for serum neutralization activity

Primary Objective 3 of this study is to evaluate serum neutralization titers of PGDM1400LS, VRC07-523LS and PGT121.414.LS against a panel of Env-pseudotyped viruses (57) at several timepoints following IV or SC

FOR REVIEW ONLY

administrations. This objective is also descriptive in nature, and will be accomplished by estimating, within each mAb treatment group, the mean area-under-the-magnitude-breadth-curve (AUC-MB) (58) of serum neutralization titers of each mAB against the panel of viruses. The precision with which a true mean AUC-MB of neutralization titers can be estimated from observed data depends on the SD of the measurements and the sample size. Table 6-4 displays two-sided 95% confidence intervals for the mean AUC-MB of neutralization titer for several values of the observed average AUC-MB of ID50 or ID80 neutralization titer. The construction of these confidence intervals assumed sample sizes of n = 12 per group, reflecting an attrition rate of 20% compared to the planned treatment group size of 16 participants, respectively. The calculations assumed that $\log_e$-transformed neutralization titers are approximately normally distributed. To account for the small sample sizes, a t-distribution was used to construct CIs. For instance, with an observed mean $\log_e$ AUC-MB titer of $\log_e(50)$ and assuming a standard deviation of 0.5 for their log-transformed values, a two-sided 95% confidence interval for the true mean drug concentration level is (36.4, 68.7) with effective sample sizes of 12 participants, respectively. Of note, based on neutralization data against a global panel of 11 pseudoviruses in six participants in HVTN104, an SD of less than 1.0 was observed in the log-transformed AUC-MB of ID50 titers at various time-points post IV infusions of VRC01 (47).

Table 6-4 Two-sided 95% confidence intervals based on observing a particular average $\log_e$-AUC-MB neutralization titer in participants in any of the active arms, taking 20% attrition into consideration (n = 12)

Observed average $\log_e$-AUC-MB neutralization titer	SD of $\log_e$-AUC-MB neutralization titer	95% confidence interval
		n = 12
$\log_e(5)$		(3.6, 6.9)
$\log_e(20)$		(14.6, 27.5)
$\log_e(50)$		(36.4, 68.7)
$\log_e(100)$	0.5	(72.8, 137.4)
$\log_e(250)$		(182.0, 343.5)
$\log_e(500)$		(363.9, 687.0)
$\log_e(1000)$		(727.8, 1373.9)
$\log_e(5)$		(2.6, 9.4)
$\log_e(20)$		(10.6, 37.8)
$\log_e(50)$		(26.5, 94.4)
$\log_e(100)$	1.0	(53.0, 188.8)
$\log_e(250)$		(132.4, 471.9)
$\log_e(500)$		(264.9, 943.9)
$\log_e(1000)$		(529.7, 1887.7)

6.2 Randomization

There will be no randomization for Group 1 as they will be enrolled first. Contingent on safety data from Group 1, Groups 2 and 3 will be randomized and

FOR REVIEW ONLY

enrolled simultaneously. Contingent on safety data from Groups 2 and 3, Groups 4 and 5 will be randomized and enrolled simultaneously. Contingent on data from Part A, Groups 6, 7, 8, and 9 will be randomized in blocks to ensure balance across groups for simultaneous enrollment. There will be no randomization for Group 10. A participant's randomization assignment will be computer generated and provided to the CRS pharmacist through a web-based randomization system.

6.3 Blinding

Participants and CRS staff will be unblinded to participant treatment arm assignments. Laboratory Center staff will be unblinded to whether a sample is from Part A or Part B, but will remain blinded to treatment assignment within Part A or Part B during sample analysis.

6.4 Statistical analyses

This section describes the final study analyses, unblinded as to treatment arm assignment. All safety data from enrolled participants will be analyzed according to the initial randomization assignment regardless of how many study product administrations and how much study product they received. In the rare instance that a participant receives the wrong treatment at a specific study product administration time, the Statistical Analysis Plan (SAP) will address how to analyze the participant's safety data. Analyses of safety data are modified intent-to-treat (MITT) in that individuals who are randomized but not enrolled do not contribute data and hence are excluded. Because of the brief length of time between randomization and enrollment—typically no more than 4 working days—very few such individuals are expected. The primary analysis of mAb concentration and anti-viral functional activity data are per-protocol (PP) in that only individuals who receive the expected mAb at the expected dose level within the expected visit window contribute data. Secondary analysis will also involve the MITT cohort, and when necessary account for the actual specimen collection time, and the actual time and dose amount of each product administration.

Analyses for primary endpoints will be performed using SAS and R. Additional software may be used to perform non-compartmental PK and population PK analyses (eg, Monolix). All other descriptive and inferential statistical analyses will be performed using SAS, StatXact, or R statistical software.

No formal multiple comparison adjustments will be employed for multiple primary or secondary endpoints. However, multiplicity adjustments will be made for certain primary or secondary endpoint assays, as discussed below, when the assay endpoint is viewed as a collection of hypotheses (eg, testing multiple pseudo-viruses to determine a positive antiviral functional activity response). Unless otherwise noted, all statistical tests will be 2-sided and will be considered statistically significant if $p < 0.05$.

FOR REVIEW ONLY

6.4.1 Analysis variables

The analysis variables consist of baseline participant characteristics, safety, mAb concentration, mAb functionality, and ADA for primary- and secondary-objective analyses.

6.4.2 Baseline comparability

Treatment arms will be compared for baseline participant characteristics using descriptive statistics.

6.4.3 Safety/tolerability analysis

Since enrollment is concurrent with receiving the first study product administration, all participants will have received at least 1 product administration and therefore will provide some safety data.

6.4.3.1 Solicited AEs

The number and percentage of participants experiencing each type of Solicited AE sign or symptom (see Section 11.2.2) will be tabulated by severity and treatment arm and the percentages displayed graphically by arm. For a given sign or symptom, each participant's Solicited AEs will be counted once under the maximum severity for all injection visits. In addition, to the individual types of events, the maximum severity of local pain or tenderness, induration or erythema, and of systemic symptoms will be calculated. Kruskal-Wallis tests will be used to test for differences in severity between arms.

6.4.3.2 SAEs and Unsolicited AEs

Unsolicited AEs (see Section 11.2.2) will be summarized using MedDRA System Organ Class and preferred terms. Tables will show by treatment arm the number and percentage of participants experiencing an Unsolicited AE within a System Organ Class or within preferred term category by severity or by relationship to study product. For the calculations in these tables, a participant with multiple Unsolicited AEs within a category will be counted once under the maximum severity or the strongest recorded causal relationship to study product. Formal statistical testing comparing arms is not planned since interpretation of differences must rely heavily upon clinical judgment.

A listing of SAEs reported to the DAIDS Regulatory Support Center (RSC) Safety Office will provide details of the events including severity, relationship to study product, time between onset and last study product administration, and number of study product administrations received.

6.4.3.3 Local laboratory values

Box plots of local laboratory values will be generated for baseline values and for values measured during the course of the study by treatment arm and visit. Each

FOR REVIEW ONLY

box plot will show the first quartile, the median, and the third quartile. Outliers (values outside the box plot) will also be plotted. If appropriate, horizontal lines representing boundaries for abnormal values will be plotted.

For each local laboratory measure, summary statistics will be presented by treatment arm and timepoint, as well as changes from baseline for postenrollment values. In addition, the number (percentage) of participants with local laboratory values recorded as meeting Grade 1 AE criteria or above as specified in the Division of AIDS Table for Grading the Severity of Adult and Pediatric Adverse Events (see Section 9.8) will be tabulated by treatment arm for each poststudy product administration timepoint. Reportable clinical laboratory abnormalities without an associated clinical diagnosis will also be included in the tabulation of AEs described above.

6.4.3.4 Reasons for study product administration discontinuation and early study termination

The number and percentage of participants who discontinue study product administration and who terminate the study early will be tabulated by reason and treatment arm.

6.4.4 mAb concentration and PK analysis

6.4.4.1 Primary analyses of mAb concentrations

The primary analysis of serum concentration and PK of PGDM1400LS, VRC07-523LS and PGT121.414.LS (Primary Objective 2) will be restricted to participants who received all scheduled administrations per-protocol. Serum concentrations that fail the quality control of the assay, or from specimens collected outside of the visit window, or from HIV-infected participants postinfection may be excluded. The primary analysis of serum concentration will be descriptive and performed separately for each mAb.

A non-compartmental PK analysis will be performed on the concentration data. PK parameters may include, but are not limited to: area-under-the-curve (AUC), maximum concentration (C_{max}), time to C_{max} (T_{max}), clearance (CL), volume of distribution (Vd), terminal elimination rate constant (λz) and the terminal half-life ($T_{1/2}$). For SC Groups, the PK parameters will include AUC, C_{max}, T_{max}, clearance (CL/F, where F is the bioavailability via SC relative to IV), volume of distribution (Vd/F), λz and $T_{1/2}$. Data will be summarized by each dose group and overall for CL, Vd and $T_{1/2}$. The potential for non-linear PK among IV or SC recipients will be determined by comparing the dose-adjusted ratios for C_{max} and AUC within IV or SC dosing groups. Graphical displays of the data (eg, boxplots, scatterplots, histograms, spaghetti plots) will be generated to visually explore distributional properties of the data. These summary statistics and graphical displays may be produced for each treatment arm and each time point separately.

FOR REVIEW ONLY

6.4.5 Analysis of neutralization activity and correlation with serum concentrations

6.4.5.1 Primary analyses of neutralization magnitude-breadth curves

To address Primary objective 3, at each specified timepoint, the area-under-the-magnitude-breadth curve (AUC-MB) to a global panel of pseudoviruses (57) will be computed for each participant in the PP cohort with evaluable neutralization ID_{50} or ID_{80} data, as described in (58). Magnitude–Breadth (M-B) curves maybe employed to display individual- and group-level response breadth as a function of magnitude. Response breadth is defined as the percentage of viruses in the panel with neutralization titer above certain thresholds. Two choices are to compare the M-B curves among arms, as follows: a non-parametric Wilcoxon rank sum test on the subject-specific area-under-the M-B curve (AUC M-B) or a Kolmogorov-Smirnov type test on the 2 group-average M-B curves. Simulations can be used to obtain 2-sided p-values for the latter test. Second, a weighted-average score-like variable may be constructed to account for the correlations between virus isolates as an integrated magnitude of responses to multiple isolates. Similar group comparison methods described in the first approach may be adopted. Details of either approach will be described in the SAP.

6.4.5.2 Secondary analyses of correlations between serum concentrations and serum neutralization levels

To address Secondary objective 1, pharmacodynamics (PD) models based on either linear or non-linear mixed effects models will be performed to characterize the correlation between serum concentration (observed or popPK model-predicted) and serum neutralization against each virus or the AUC-MB of serum neutralization against the panel. Data from all enrolled participants will be analyzed regardless of how many administrations and how much mAb dose they received (MITT analysis). Similar to the popPK analysis, data from specimens collected outside of the visit window may be included in the PK/PD analyses that account for the actual specimen collection time, and the actual time and dose amount of each product administration. Since the exact date of HIV infection is unknown, any serum level data from blood draws 4 weeks prior to an infected participant's last seronegative sample and thereafter may be excluded. All data from HIV-infected participants who have no seronegative samples postenrollment may be excluded from the analysis.

6.4.5.3 Secondary analyses of neutralization magnitude-breadth curves

To address Secondary objective 2, at each specified timepoint, the area-under-the-magnitude-breadth curve (AUC-MB) to a panel of Env-pseudotyped reference viruses that are sensitive to all three bnAbs will be computed for all participants with evaluable neutralization ID50 or ID80 data, as described in (58). Magnitude–Breadth (M-B) curves may be employed to display individual- and group-level response breadth as a function of magnitude.

FOR REVIEW ONLY

6.4.6 Analysis of ADA and other non-neutralizing functional activities

For the analysis of ADA (Secondary objective 3), data from enrolled participants will be used regardless of how many administrations they received (MITT). For Exploratory objectives regarding mAb functionality, data from enrolled participants who received all scheduled administrations PP will be used. Assay results that are unreliable or from HIV-infected participants postinfection will be excluded. Additional exploratory analyses examining the impact of confirmed positive ADA on PK will be described in the SAP.

6.4.7 General approach

Univariate and bivariate descriptive analyses of continuous assay data (eg, Luminex-based serum concentrations) will be performed using mean, median, standard deviation, range, skewness, Spearman's and Pearson's correlation coefficients, for example. Graphical displays of the data based on appropriate techniques (eg, boxplots, histograms, kernel density estimates, probability plots, two- or three-dimensional scatterplots, spaghetti plots) will be generated to visually explore distributional properties of the data as well as potential pairwise associations. Statistics and graphical displays will be produced for each treatment arm across timepoints.

Comparisons of continuous assay data between treatment groups or timepoints will be primarily performed using nonparametric rank-based tests, the Wilcoxon rank-sum test, or Friedman nonparametric two-way analysis of variance (ANOVA). In the event the data appear normally distributed, the comparisons may be performed using appropriate parametric tests (eg, two-sample t-tests with unequal variances). Appropriate data transformations (eg, square-root, logarithmic) may be applied prior to testing hypotheses in order for key distributional assumptions [eg, normality, homoscedasticity (ie, constancy of variance) to be satisfied.

Analyses of categorical variables (eg, binary) will be conducted by constructing frequency tables. One such table will be produced for each treatment group and each timepoint. Crude response rates will be presented with their corresponding 95% confidence interval estimates calculated using the score test method (59). Associations between categorical variables will be assessed using Barnard's (2x2 tables), Fisher's exact or Chi-squared tests.

Analysis of longitudinal data may be performed using mixed effects models or generalized estimating equations (GEE). These approaches allow describing outcome responses over several timepoints while accounting for potential inter-subject heterogeneity. To achieve unbiased statistical estimation and inferences with nonparametric tests and generalized linear models fit by GEE methods, missing data need to be missing completely at random (MCAR). MCAR assumes that missingness does not depend on any observed or unobserved data (ie, the observed data is just a random sample of all the potential data). When missingness is negligible (eg, less than 20%), statistical methods (eg, nonparametric tests and

FOR REVIEW ONLY

GEE methods) based on the MCAR assumption can be used with limited impact on the analysis. When the frequency of missing data is more substantial, methods that require the MCAR assumption may give misleading results. In this situation, statistical analyses will be performed based on appropriate modeling assumptions and adjusted using weighting methods, or combined with imputation, under the assumption that the missing data are missing at random (MAR). MAR assumes that the probability of an observation being missing only depends on the observed responses or covariates. Thus, this assumption is less stringent than the MCAR assumption. Weighting adjustments (eg, weighted GEE) and imputation methods are valid under MAR. We will consider including any of the available baseline predictors of the missing outcomes as covariates in statistical models. Please see Little and Rubin (59), Chapters 1, 3, and 6] for elaborate definitions and examples of missing data mechanisms and Ibrahim et al (60) for a review of missing data methods in clinical studies.

Generalized linear models for response rates will use a binomial error distribution and for quantitative endpoints, a normal error distribution. We will assess repeated functional measurement using linear mixed effects models. If functional activity outcomes are left- and/or right- censored, we will use Hughes' (73) linear mixed effects models to accommodate censoring. In addition, exploratory analyses of repeated functional measurements may be done using weighted GEE (74) methods, which are valid under MAR. We will again consider including any of the available baseline predictors of the missing outcomes as covariates in statistical models.

6.4.8 Analyses prior to end of scheduled follow-up visits

Any analyses conducted prior to the end of the scheduled follow-up visits should not compromise the integrity of the trial in terms of participant retention or other study endpoint assessments.

6.4.8.1 Safety

During the course of the trial, unblinded analyses of safety data will be prepared approximately every 4 months for review by the SMB. Ad hoc safety reports may also be prepared for SMB review at the request of the HVTN 140/HPTN 101 PSRT.

FOR REVIEW ONLY

7 Selection and withdrawal of participants

Participants will be healthy, HIV-uninfected (seronegative) adults who comprehend the purpose of the study and have provided written informed consent. Volunteers will be recruited and screened; those determined to be eligible, based on the inclusion and exclusion criteria, will be enrolled in the study. Final eligibility determination will depend on information available at the time of enrollment, including results of screening laboratory tests, medical history, physical examinations, and answers to self-administered and/or interview questions.

Investigators should always use good clinical judgment in considering a volunteer's overall fitness for trial participation. Some volunteers may not be appropriate for enrollment even if they meet all inclusion/exclusion criteria. Medical, psychiatric, occupational, or other conditions may make evaluation of safety and/or other endpoints difficult, and some volunteers may be poor candidates for retention.

Determination of eligibility, taking into account all inclusion and exclusion criteria, must be made within 56 days prior to enrollment unless otherwise noted in Sections 7.1 and 7.2.

7.1 Inclusion criteria

General and Demographic Criteria

1. **Age** of 18 through 50 years

2. **Access to a participating CRS** and willingness to be followed for the planned duration of the study

3. Ability and willingness to provide **informed consent**

4. **Assessment of understanding**: volunteer demonstrates understanding of this study and completes a questionnaire prior to first study product administration with verbal demonstration of understanding of all questionnaire items answered incorrectly

5. **Agrees not to enroll in another study** of an investigational research agent until completion of the last required protocol clinic visit.

6. **Good general health** as shown by medical history, physical exam, and screening laboratory tests

HIV-Related Criteria:

7. Willingness to receive **HIV test results**

FOR REVIEW ONLY

8. Willingness to discuss **HIV infection risks** and amenable to HIV risk reduction counseling.

9. Assessed by the clinic staff as being at **"low risk" for HIV infection** and committed to maintaining behavior consistent with low risk of HIV exposure through the last required protocol clinic visit (see Appendix J and Appendix K).

Laboratory Inclusion Values

<u>Hemogram/Complete blood count (CBC)</u>

10. **Hemoglobin**

- ≥ 11.0 g/dL for volunteers who were assigned female sex at birth
- ≥ 13.0 g/dL for volunteers who were assigned male sex at birth and transgender males who have been on hormone therapy for more than 6 consecutive months
- ≥ 12.0 g/dL for transgender females who have been on hormone therapy for more than 6 consecutive months
- For transgender volunteers who have been on hormone therapy for less than 6 consecutive months, determine hemoglobin eligibility based on the sex assigned at birth

11. **White blood cell count** = 2,500 to 12,000 cells/mm3

12. **WBC differential** either within institutional normal range or with site clinician approval

13. **Platelets** = 125,000 to 550,000 cells/mm3

<u>Chemistry</u>

14. **Chemistry panel**: **alanine aminotransferase** (ALT) < 1.25 times the institutional upper limit of normal (ie, < 1.25 times the reference range upper limit) and **creatinine** < 1.1 times the institutional upper limit of normal (ie, <1.1 times the reference range upper limit)

<u>Virology</u>

15. **Negative HIV-1 and -2 blood test**: US volunteers must have a negative FDA-approved enzyme immunoassay (EIA) or chemiluminescent microparticle immunoassay (CMIA). Non-US sites may use locally available assays that have been approved by HVTN and HPTN Laboratory Operations

16. **Negative Hepatitis B surface antigen (HBsAg)**

FOR REVIEW ONLY

17. **Negative anti-Hepatitis C virus antibodies (anti-HCV)**, or negative HCV polymerase chain reaction (PCR) if the anti-HCV is positive

Urine

18. **Negative or trace urine protein**

Reproductive Status

19. **Volunteers who were assigned female sex at birth:** negative serum or urine beta human chorionic gonadotropin (β-HCG) pregnancy test(s) performed within 48 hours prior to initial study product administration. Persons who are NOT of reproductive potential due to having undergone total hysterectomy or bilateral oophorectomy (verified by medical records), are not required to undergo pregnancy testing.

20. **Reproductive status**

    A volunteer who was assigned female sex at birth must:

    - Agree to use effective contraception for sexual activity that could lead to pregnancy from at least 21 days prior to enrollment through the last required protocol visit. Effective contraception is defined as using one of the following methods:
        - Condoms (internal and external) with or without a spermicide,
        - Diaphragm or cervical cap with spermicide,
        - Intrauterine device (IUD),
        - Hormonal contraception,
        - Tubal ligation, or
        - Any other contraceptive method approved by the HVTN 140/HPTN 101 PSRT
        - Successful vasectomy in any partner assigned male sex at birth (considered successful if a volunteer reports that a male partner has [1] documentation of azoospermia by microscopy, or [2] a vasectomy more than 2 years ago with no resultant pregnancy despite sexual activity postvasectomy); or,
    - Not be of reproductive potential, such as having reached menopause (no menses for 1 year) or having undergone hysterectomy or bilateral oophorectomy; or,
    - Be sexually abstinent.

FOR REVIEW ONLY

21. **Volunteers who were assigned female sex at birth must also agree not to seek pregnancy through alternative methods**, such as artificial insemination or *in vitro* fertilization until after the last required protocol clinic visit

7.2 Exclusion criteria

General

1. **Weight** < 35kg or > 115 kg

2. **Blood products** received within 120 days before first study product administration, unless eligibility for earlier enrollment is determined by the HVTN 140/HPTN 101 PSRT

3. **Investigational research agents** received within 30 days before first study product administration

4. **Intent to participate in another study** of an investigational research agent or any other study that requires non-Network HIV antibody testing during the planned duration of the HVTN 140/HPTN 101 study

5. **Pregnant or breastfeeding**

Vaccines, Antibodies, and other Injections or Infusions

6. **HIV vaccine(s)** received in a prior HIV vaccine trial. Volunteers who have received control/placebo in an HIV vaccine trial are not excludedHVTN 140/HPTN 101.

7. **SARS-CoV-2 vaccine(s)** received within 7 days prior to HVTN 140/HPTN 101 enrollment or planned within 7 days after enrollment.

8. **Receipt of humanized or human mAbs,** whether licensed or investigational.

9. **Previous receipt of mAbs VRC01, VRC01LS, VRC07-523LS, PGDM1400, PGT121, PGT121.414.LS.**

Immune System

10. **Immunosuppressive medications** received within 30 days before first study product administration (Not exclusionary: [1] corticosteroid nasal spray; [2] inhaled corticosteroids; [3] topical corticosteroids for mild, uncomplicated dermatological condition; or [4] a single course of oral/parenteral prednisone or equivalent at doses < 20 mg/day and length of therapy < 14 days)

11. **Serious adverse reactions** to PGDM1400LS, VRC07-523LS, or PGT121.414.LS formulation components (see Section 8.2) including history of anaphylaxis and

FOR REVIEW ONLY

related symptoms such as hives, respiratory difficulty, angioedema, and/or abdominal pain.

12. **Immunoglobulin** received within 60 days before first study product administration (for mAb see criterion 8 above)

13. **Autoimmune disease** (Not excluded from participation: Volunteer with mild, stable and uncomplicated autoimmune disease that does not require immunosuppressive medication and that, in the judgment of the CRS investigator, is likely not subject to exacerbation and likely not to complicate Solicited and Unsolicited AE assessments.)

14. **Immunodeficiency**

Clinically significant medical conditions

15. **Clinically significant medical condition**, physical examination findings, clinically significant abnormal laboratory results, or past medical history with clinically significant implications for current health. A clinically significant condition or process includes but is not limited to:

    - Symptoms consistent with COVID-19 or known SARS-CoV-2 infection,
    - A process that would affect the immune response,
    - A process that would require medication that affects the immune response,
    - Any contraindication to repeated infusions, or blood draws, including inability to establish venous or subcutaneous access,
    - A condition that requires active medical intervention or monitoring to avert grave danger to the volunteer's health or well-being during the study period,
    - A condition or process (eg, chronic urticaria or recent injection or infusion with evidence of residual inflammation) for which signs or symptoms could be confused with reactions to the study product, or
    - Any condition specifically listed among the exclusion criteria.

16. **Any medical, psychiatric, or skin condition (eg, tattoos), or occupational responsibility** that, in the judgment of the investigator, would interfere with or serve as a contraindication to protocol adherence, assessment of safety or Solicited AEs, or a participant's ability to give informed consent.

17. **Psychiatric condition that precludes compliance with the protocol**. Specifically excluded are persons with psychoses within the past 3 years, ongoing risk for suicide, or history of suicide attempt or gesture within the past 3 years.

FOR REVIEW ONLY

18. **Current anti-tuberculosis (TB) therapy**

19. **Asthma** other than mild, well-controlled asthma. (Symptoms of asthma severity as defined in the most recent National Asthma Education and Prevention Program (NAEPP) Expert Panel report).

    Exclude a volunteer who:

    - Uses a short-acting rescue inhaler (typically a beta 2 agonist) daily, or
    - Uses moderate/high-dose, inhaled corticosteroids, or
    - In the past year has had either of the following:
        - Greater than 1 exacerbation of symptoms treated with oral/parenteral corticosteroids;
        - Emergency care, urgent care, hospitalization, or intubation for asthma.

20. **Diabetes mellitus** type 1 or type 2 (Not excluded: type 2 cases controlled with diet alone or a history of isolated gestational diabetes.)

21. **Hypertension**:

    - If a person has been found to have elevated blood pressure or hypertension during screening or previously, exclude for blood pressure that is not well controlled. Well-controlled blood pressure is defined in this protocol as consistently ≤ 140 mm Hg systolic and ≤ 90 mm Hg diastolic, with or without medication, with only isolated, brief instances of higher readings, which must be ≤ 150 mm Hg systolic and ≤ 100 mm Hg diastolic. For these volunteers, blood pressure must be ≤ 140 mm Hg systolic and ≤ 90 mm Hg diastolic at enrollment.

    - If a person has NOT been found to have elevated blood pressure or hypertension during screening or previously, exclude for systolic blood pressure ≥ 150 mm Hg at enrollment or diastolic blood pressure ≥ 100 mm Hg at enrollment.

22. **Bleeding disorder** diagnosed by a clinician (eg, factor deficiency, coagulopathy, or platelet disorder requiring special precautions)

23. **Malignancy** (Not excluded from participation: Volunteer who has had malignancy excised surgically and who, in the investigator's estimation, has a reasonable assurance of sustained cure, or who is unlikely to experience recurrence of malignancy during the period of the study)

24. **Seizure disorder:** History of seizure(s) within past 3 years. Also exclude if volunteer has used medications in order to prevent or treat seizure(s) at any time within the past 3 years.

FOR REVIEW ONLY

25. **Asplenia**: any condition resulting in the absence of a functional spleen

26. History of **generalized urticaria**, **angioedema**, or **anaphylaxis** (Not exclusionary: angioedema or anaphylaxis to a known trigger with at least 5 years since last reaction to demonstrate satisfactory avoidance of trigger).

7.3 Participant departure from study product administration schedule or withdrawal (Part B)

This section concerns an individual participant's departure from the study-product administration schedule. Pause rules for the trial are described in Section 11.4.

7.3.1 Delaying study product administrations for a participant (Part B only)

Under certain circumstances, a participant's scheduled study product administration will be delayed. Refer to the SSP for further guidance regarding which procedures to conduct in these instances. The factors to be considered in such a decision include but are not limited to the following:

- Within 7 days prior to study product administration
  - Receipt of systemic glucocorticoids (eg, prednisone or other glucocorticoids) or other immunomodulators (other than nonsteroidal anti-inflammatory drugs [NSAIDs])
- Within 7 days of study product administration (ie, *within 7 days prior to* study product administration, or *planned within 7 days after* study product administration)
  - Receipt of SARS CoV-2 vaccine
- Preinfusion abnormal vital signs or clinical symptoms that may mask assessment of study product reaction.
- Intercurrent illness that is assessed by CRS principal investigator (or designee) to require delaying study product administration. The investigator may consult the HVTN 140/HPTN 101 PSRT.
- Pregnancy: study product administration will be stopped while a participant is pregnant. If the participant is no longer pregnant (as defined by two consecutive negative tests) or breast-feeding and study product administration can be performed within an appropriate visit window, study product administration may resume with unanimous consent of the HVTN 140/HPTN 101 PSRT.

FOR REVIEW ONLY

7.3.2 Participant departure from study product administration schedule

Every effort should be made to follow the study product-administration schedule per the protocol. If a participant misses a study product administration and the visit window period for the study product administration has passed, that study product cannot be given. The participant should be asked to continue study visits. (see Sections 7.3.1 and 7.3.3).

7.3.3 Discontinuing study product administration for a participant

Under certain circumstances, an individual participant's study product administrations will be permanently discontinued. Specific events that will result in stopping a participant's study product-administration schedule include:

- Co-enrollment in a study with an investigational research agent (rare exceptions allowing for the continuation of study product administrations may be granted with the unanimous consent of the HVTN 140/HPTN 101 PSRT)
- Clinically significant condition (ie, a condition that affects the immune system or for which continued study product administrations and/or blood draws may pose additional risk), including but not limited to the following:
  - HIV infection
  - Any grade 4 local or systemic Solicited or Unsolicited adverse event (AE) that is subsequently considered to be related to study product administration
  - Grade 3 clinical AE that is subsequently considered to be related to study product administration with the exception of fever, vomiting, and subjective local and systemic symptoms. For grade 3 infusion site erythema and/or induration, upon review, the HVTN 140/HPTN 101 PSRT may allow continuation of study product administration
  - Any grade 3 or 4 lab abnormality confirmed by a repeated value that is subsequently considered to be related to study product;
  - SAE that is subsequently considered to be related to study product administration
  - Clinically significant hypersensitivity or mAb reaction including, but not limited to, type 1 hypersensitivity reaction, urticaria, or serum sickness associated with study product administration. Consultation with the HVTN 140/HPTN 101 PSRT is required prior to subsequent study product administrations following any hypersensitivity reaction associated with study product administration
- Investigator determination in consultation with Protocol Team leadership (eg, for repeated nonadherence to study staff instructions)

FOR REVIEW ONLY

Participants discontinuing study product for reasons other than HIV infection should be counseled on the importance of continuing with the study and strongly encouraged to participate in follow-up visits and protocol-related procedures per the protocol for the remainder of the trial, unless medically contraindicated (see HVTN 140/HPTN 101 SSP).

Participants diagnosed with HIV infection during the study should be encouraged to participate in follow-up visits as indicated in Section 9.12.

7.3.4 Participant termination from the study

Under certain circumstances, an individual participant may be terminated from participation in this study. Specific events that will result in early termination include:

- Participant refuses further participation,
- Participant relocates and remote follow-up or transfer to another CRS is not possible,
- CRS determines that the participant is lost to follow-up,
- Investigator decides, in consultation with Protocol Team leadership, to terminate participation (eg, if participant exhibits inappropriate behavior toward clinic staff), or
- Any condition where termination from the study is required by applicable regulations.

FOR REVIEW ONLY

8 Study product

CRS pharmacists should consult the Pharmacy Guidelines and Instructions for DAIDS Clinical Trials Networks for standard pharmacy operations. The protocol schema is shown in Table 1-1. See the IBs for further information about study products.

8.1 Study product regimen

The schedule of study product administration is shown in Section 1 and additional information is given below.

Part A

Group 1: PGDM1400LS 5 mg/kg to be administered via IV infusion at Month 0

Group 2: PGDM1400LS 20 mg/kg to be administered via IV infusion at Month 0

Group 3: PGDM1400LS 20 mg/kg to be administered via SC infusion at Month 0

Group 4: PGDM1400LS 40 mg/kg to be administered via IV infusion at Month 0

Group 5: PGDM1400LS 40 mg/kg to be administered via SC infusion at Month 0

Part B

Group 6: PGDM1400LS 20mg/kg + VRC07-523LS 20mg/kg + PGT121.414.LS 20 mg/kg to be administered via IV infusion sequentially in this order at Month 0 and Month 4

Group 7: PGDM1400LS 20mg/kg + VRC07-523LS 20mg/kg + PGT121.414.LS 20 mg/kg to be administered via SC infusion sequentially in this order at Month 0 and Month 4

Group 8: PGDM1400LS 1.4gram + VRC07-523LS 1.4gram + PGT121.414.LS 1.4gram to be administered via IV infusion sequentially in this order at Month 0 and Month 4

Group 9: PGDM1400LS 1.4gram + VRC07-523LS 1.4gram + PGT121.414.LS 1.4gram to be administered via SC infusion sequentially in this order at Month 0 and Month 4

Group 10: PGDM1400LS 40mg/kg + VRC07-523LS 40mg/kg + PGT121.414.LS 40 mg/kg to be administered via IV infusion sequentially in this order at Month 0 and Month 4

FOR PREVIEW ONLY

8.2 Study product formulation

8.2.1 PGDM1400LS

PGDM1400LS will be supplied as 10 mL single-use glass vials with a 4.75± 0.1 mL fill volume, at a concentration of 100 mg/mL. Upon thaw, each vial contains a clear, colorless to yellow solution for injection, which is essentially free from foreign particles, but some opaque or translucent particles may be present. The formulation buffer is composed of 10 mM Acetate, 9% w/v Sucrose, 0.01% w/v Polysorbate 80 at pH 5.2. It does not contain a preservative.

The product is stored frozen at -35°C to -15°C in a qualified, continuously monitored, temperature-controlled freezer until use. The study product is described in further detail in the IB.

8.2.2 VRC07-523LS

VRC07-523LS will be supplied as 10 mL single-use glass vials with a 6.25 ± 0.1mL fill volume and 3 mL single-use glass vials with a 2.25 mL ± 0.1 mL fill volume, at a concentration of 100 ± 10 mg/mL. Each vial contains a clear, colorless to yellow liquid, essentially free of visible particles; some opaque or translucent particles may be present. The formulation buffer is composed of 50mM histidine, 50 mM sodium chloride, 5% sucrose, and 2.5% sorbitol at pH 6.8. Vials do not contain a preservative.

VRC07-523LS product label designates the long-term storage as -35ºC to -15 ºC (-31 ºF to 5 ºF). The study product is described in further detail in the IB.

8.2.3 PGT121.414.LS

PGT121.414.LS will be supplied as 10 mL single-use glass vials with a 4.75 mL fill volume, at a concentration of 100 mg/mL. Each vial contains a clear, colorless to yellow, preservative free, sterile solution for injection. The formulation buffer is composed of acetate, sucrose, polysorbate 80 at pH of 5.2. PGT121.414.LS product label designates the long-term storage as -35°C to -15°C (-31°F to 5°F).

8.3 Preparation of study products

Prior to preparation of the first infusion (enrollment visit), a new prescription will be sent to the pharmacy. For participants randomized to a weight-based (mg/kg) dosing group, the prescription MUST contain the participant's weight based upon the participant's weight at the most recent visit where weight was measured (this includes screening). If this information is NOT on the prescription, the prescription will be returned to the clinic from the pharmacy to be completed appropriately prior to the pharmacist beginning preparation of study product. Subsequent visit weights (based upon the participant's weight at the most recent visit where weight was measured) must be communicated to the pharmacy in

FOR REVIEW ONLY

writing prior to the day of the visit. Any changes in weight of more than 10% (between the prior weight and the weight on the day of the infusion visit) will require an updated visit weight communication to the pharmacy in writing so that product can be prepared based on that weight change.

For Groups 8 and 9, the prescription will be written as the fixed dose per the randomized dose assignment.

For Groups 3, 5, 7 and 9, the prescription must also contain the number of infusion sites that will be used for each study product. The prescription may also need to contain additional information (eg, amount of overfill the syringe must contain to account for residual volume in the tubing), depending on the tubing and pump to be used for study product administration. See HVTN 140/HPTN 101 SSP for more details.

Pharmacists must follow appropriate aseptic technique and sterile preparation procedures as outlined in United States Pharmacopeia (USP) <797> Pharmaceutical Compounding – Sterile Preparations, utilizing a pharmacy biosafety cabinet/isolator or better. Local regulations and site institutional policies and procedures for use of personal protective equipment such as gloves, gowns, masks, and safety glasses, must be followed. Pharmacists should follow the requirements of their country, their institution, and their pharmacy regulatory authority regarding these procedures.

Any unused portion of study product will not be used for another participant. Any empty vials, unused portion of entered vials, or unused solution which contains study product should be discarded in a biohazard containment bag and incinerated or autoclaved in accordance with institutional or pharmacy policy.

8.3.1 PGDM1400LS

8.3.1.1 Thawing instructions

1. Remove PGDM1400LS vials from the freezer and thaw at controlled room temperature (maximum 27°C).

2. After thawing, the PGDM1400LS vials may be stored at 2°C to 8°C for up to 48 hours and/or at 15°C to 27°C for up to 24 hours. Product may not be stored in direct sunlight. If stored at 2°C to 8°C, vials must be equilibrated at controlled room temperature (15°C - 27°C) for a minimum of 30 minutes and may be held at room temperature for up to 8 hours prior to product preparation. PGDM1400LS vials should not be refrozen after thaw.

8.3.1.2 PGDM1400LS weight-based dose intravenous infusion preparation

1. Calculate the total dose (mg) of PGDM1400LS required based on the participant's weight (in kg). Obtain the minimum number of thawed

FOR REVIEW ONLY

PGDM1400LS vials, as well as an appropriately sized IV container (bag/glass bottle) containing 100 mL of 0.9% Sodium Chloride for Injection, USP, that will also permit the addition of the required calculated volume of PGDM1400LS.

2. Using an 18G needle, withdraw the calculated volume of PGDM1400LS into a sterile syringe.

3. Using aseptic technique, remove the air from the IV container and then add the withdrawn volume of PGDM1400LS to the IV container with 100 mL of 0.9% Sodium Chloride for Injection, USP. Record this as the study product preparation time.

4. Gently mix the prepared IV container. Ensure there are no visible particulates in the IV container.

5. After product preparation in IV bags, the prepared PGDM1400LS product may be stored at 2°C to 8°C up to 24 hours and/or at controlled room temperature (15°C - 27°C) for a maximum of 4 hours total including the infusion time. Product may not be stored in direct sunlight. If stored at 2°C to 8°C, prepared product must be equilibrated at controlled room temperature (15°C - 27°C) for a minimum of 30 minutes prior to product administration.

8.3.1.3 PGDM1400LS fixed-dose intravenous infusion

1. Obtain 4 vials of thawed PGDM1400LS vials, as well as an appropriately sized IV container (bag/glass bottle) containing 100 mL of 0.9% Sodium Chloride for Injection, USP, that will also permit the addition of the required calculated volume of PGDM1400LS.

2. Using an 18G needle, withdraw a total of 14 mL of PGDM1400LS into a sterile syringe.

3. Using aseptic technique, remove the air from the IV container and then add the withdrawn 14 mL of PGDM1400LS to the IV container with 100 mL of 0.9% Sodium Chloride for Injection, USP. Record this as the study product preparation time.

4. Gently mix the prepared IV container. Ensure there are no visible particulates in the IV container.

5. After product preparation in IV bags, the prepared PGDM1400LS product may be stored at 2°C to 8°C up to 24 hours and/or at controlled room temperature (15°C - 27°C) for a maximum of 4 hours total including the infusion time. Product may not be stored in direct sunlight. If stored at 2°C to 8°C, prepared product must be equilibrated at controlled room

FOR REVIEW ONLY

temperature (15°C - 27°C) for a minimum of 30 minutes prior to product administration.

8.3.1.4 PGDM1400LS weight-based dose subcutaneous infusion preparation

1. Calculate the total dose (mg) of PGDM1400LS required based on the participant's weight (in kg). Remove the minimum number of thawed PGDM1400LS vials from storage.

2. Using aseptic technique, withdraw the calculated volume of PGDM1400LS from the vial(s) into a syringe using a 5-micron filter needle (see HVTN 140/HPTN 101 SSP for needle/filter specifications and details). A new filter needle must be used for each vial. Add the additional volume of PGDM1400LS needed to accommodate for the residual volume left in the tubing after infusion. Discard the filter needle(s) prior to dispensing. Record this as the study product preparation time.

3. After preparation in syringes for SC administration, the prepared PGDM1400LS product may be stored at 2°C to 8°C up to 24 hours or at controlled room temperature (15°C - 27°C) up to 4 hours including administration time. Product may not be stored in direct sunlight. If stored at 2°C to 8°C, prepared product must be equilibrated at controlled room temperature (15°C - 27°C) for a minimum of 30 minutes prior to product administration.

8.3.1.5 PGDM1400LS fixed-dose subcutaneous infusion preparation

1. Remove 4 vials of thawed PGDM1400LS vials from storage.

2. Using aseptic technique, withdraw 14 mL of PGDM1400LS from the vial(s) into a syringe using a 5-micron filter needle (see HVTN 140/HPTN 101 SSP for needle/filter specifications and details). A new filter needle must be used for each container. Add the additional volume of PGDM1400LS needed to accommodate for the residual volume left in the tubing after infusion. Discard the filter needle prior to dispensing. Record this as the study product preparation time.

3. After preparation in syringes for SC administration, the prepared PGDM1400LS product may be stored at 2°C to 8°C up to 24 hours or at controlled room temperature (15°C - 27°C) up to 4 hours including administration time. Product may not be stored in direct sunlight. If stored at 2°C to 8°C, prepared product must be equilibrated at controlled room temperature (15°C - 27°C) for a minimum of 30 minutes prior to product administration.

FOR PREVIEW ONLY

8.3.2 VRC07-523LS

VRC07-523LS is a highly concentrated protein solution and may develop white, opaque to translucent particles after thawing.

8.3.2.1 Thawing instructions

1. Thaw vial(s) for a minimum of 1 hour at controlled room temperature (maximum 27°C) after removing from the freezer.

2. Keep the material at room temperature during the entire preparation period until use, up to the maximum storage times described in # 4 below.

3. Prior to preparation for administration, swirl vials for 30 seconds to resuspend any visible particles, avoid foaming. DO NOT SHAKE THE VIALS. If particles are observed, return the vials to 2°C to 8°C storage. If the particles re-dissolve within the maximum storage times described in # 4 below, they may be used for product preparation. If particles continue to be observed, do not use the vials.

4. Thawed vials may be stored for up to 24 hours at controlled room temperature (maximum 27°C) and/or up to 2 weeks (14 days) at 2°C to 8°C. Product may not be stored in direct sunlight. If stored at 2 ºC to 8 ºC, vials must be equilibrated at controlled room temperature (maximum 27 ºC) for a minimum of 30 minutes and may be held at room temperature for up to 8 hours prior to product preparation.

8.3.2.2 VRC07-523LS weight-based dose intravenous infusion preparation

1. Calculate the total dose (mg) of VRC07-523LS required based on participant's weight (in kg). Remove the minimum number of thawed particle free VRC07-523LS vials from storage, as well as an appropriately sized IV container (bag/glass bottle) containing 100 mL of 0.9% Sodium Chloride for Injection, USP, that will also permit the addition of the required calculated volume of VRC07-523LS.

2. Gently swirl thawed vials for 30 seconds, avoiding foaming. DO NOT SHAKE VIALS. Keep the vials upright at all times until ready to withdraw the contents. Do not invert the vials during inspection.

3. Observe vials for particles. If particles are observed, refer to the thawing instructions described above in Section 8.3.2.1.

4. Using aseptic technique, add the calculated volume of VRC07-523 LS to the IV container with 100 mL of Sodium Chloride for Injection, 0.9 USP. Record this as the study product preparation time.

5. The prepared VRC07-523LS IV container may be stored at 2°C to 8°C up to 48 hours or at controlled room temperature (maximum 27°C) for a

FOR REVIEW ONLY

maximum of 4 hours total including the infusion time. Product may not be stored in direct sunlight. If stored at 2°C to 8°C, prepared product must be equilibrated at controlled room temperature (maximum 27°C) for a minimum of 30 minutes prior to product administration.

8.3.2.3 VRC07-523LS fixed-dose intravenous infusion preparation

1. Remove either three 10 mL vial sizes or eight 3 mL vial sizes of thawed particle free VRC07-523LS from storage, as well as an appropriately sized IV container (bag/glass bottle) containing 100 mL of 0.9% Sodium Chloride for Injection, USP, that will also permit the addition of the required calculated volume of VRC07-523LS.

2. Gently swirl thawed vials for 30 seconds, avoiding foaming. DO NOT SHAKE VIALS. Keep the vials upright at all times until ready to withdraw the contents. Do not invert the vials during inspection.

3. Observe vials for particles. If particles are observed, refer to the thawing instructions described above in Section 8.3.2.1.

4. Using aseptic technique, add 14 mL of VRC07-523 LS to the IV container with 100 mL of 0.9% Sodium Chloride for Injection, USP. Record this as the study product preparation time.

5. The prepared VRC07-523LS IV container may be stored at 2°C to 8°C up to 48 hours or at controlled room temperature (maximum 27°C) for a maximum of 4 hours total including the infusion time. Product may not be stored in direct sunlight. If stored at 2°C to 8°C, prepared product must be equilibrated at controlled room temperature (maximum 27°C) for a minimum of 30 minutes prior to product administration.

8.3.2.4 VRC07-523LS weight-based dose subcutaneous infusion preparation

1. Calculate the total (mg) of VRC07-523LS required based on the participant's weight (in kg). Remove the minimum number of thawed particle-free VRC07-523LS vials from storage.

2. Gently swirl thawed vials for 30 seconds to avoid foaming. DO NOT SHAKE VIALS. Keep the vials upright until ready to withdraw the contents. Do not invert the vial during inspection.

3. Observe vials for particles. If particles are observed, refer to the thawing instructions described above in Section 8.3.2.1.

4. Using aseptic technique, withdraw the calculated volume of VRC07-523LS from the vial(s) into a syringe using a 5 micron filter needle (see HVTN 140/HPTN 101 SSP for needle/filter specifications and details). A

FOR REVIEW ONLY

new filter needle must be used for each vial. Discard the filter needle prior to dispensing. Record this as the study product preparation time.

5. The prepared VRC07-523LS syringe(s) may be stored at 2°C to 8°C for up to 24 hours or at controlled room temperature (maximum 27°C) for a maximum of 4 hours, including the administration time. Product may not be stored in direct sunlight. If stored at 2°C to 8°C, prepared product must be equilibrated at controlled room temperature (maximum 27°C) for a minimum of 30 minutes prior to product administration.

8.3.2.5 VRC07-523LS fixed-dose subcutaneous infusion preparation

1. Remove either three 10 mL vial sizes or eight 3 mL vial sizes of thawed particle free VRC07-523LS from storage.

2. Gently swirl thawed vials for 30 seconds, avoiding foaming. DO NOT SHAKE VIALS. Keep the vials upright at all times until ready to withdraw the contents. Do not invert the vials during inspection.

3. Observe vials for particles. If particles are observed, refer to the thawing instructions described above in Section 8.3.2.1.

4. Using aseptic technique, withdraw 14 mL of VRC07-523 LS into a syringe using a 5 micron filter needle (see HVTN 140/HPTN 101 SSP for needle/filter specifications and details). A new filter needle must be used for each vial. Add the additional volume of VRC07-523LS needed to accommodate for the residual volume left in the tubing after infusion. Discard the filter needle prior to dispensing. Record this as the study product preparation time.

5. The prepared VRC07-523LS syringe(s) may be stored at 2°C to 8°C for up to 24 hours or at controlled room temperature (maximum 27°C) for a maximum of 4 hours, including the administration time. Product may not be stored in direct sunlight. If stored at 2°C to 8°C, prepared product must be equilibrated at controlled room temperature (maximum 27°C) for a minimum of 30 minutes prior to product administration.

8.3.3 PGT121.414.LS

8.3.3.1 Thawing instructions

1. Thaw vial(s) at room temperature and hold for at least 30 minutes post-thaw (no ice crystals present). Vials must not be moved directly from the freezer to storage at 2°C to 8°C.

2. Keep the material at room temperature during the entire preparation period until use, up to the maximum storage times described in #4 below.

FOR PREVIEW ONLY

3. Prior to preparation for administration, swirl vials for 30 seconds to resuspend any visible particles, avoid foaming. DO NOT SHAKE THE VIALS. If some white to translucent particles continue to be observed, vials may be used for the preparation of the IV or SC product.

4. Thawed vials may be stored for up to 24 hours at room temperature (maximum 27°C). If vials are not used within that time, they may be refrigerated for up to 2 weeks (14 days) at 2°C to 8°C and should be used within 8 hours of any subsequent return to room temperature (maximum 27°C). Refrigerated product must be equilibrated at room temperature (maximum 27°C) for a minimum of 30 minutes prior to use.

8.3.3.2 PGT121.414.LS weight-based dose intravenous infusion preparation

1. Calculate the total dose (mg) of PGT121.414.LS required based on the participant's weight (in kg). Remove the minimum number of thawed PGT121.414.LS vials from storage, as well as an appropriately sized IV container (bag/glass bottle) containing 100 mL of 0.9% Sodium Chloride for Injection, USP, that will also permit the addition of the required calculated volume of PGT121.414.LS.

2. Gently swirl thawed vials for 30 seconds, avoiding foaming. DO NOT SHAKE VIALS. Keep the vials upright until ready to withdraw the contents. Do not invert the vials during inspection.

3. Using aseptic technique, remove the air from the IV container and then add the calculated volume of PGT121.414.LS to the IV container with 100 mL of Sodium Chloride for Injection, 0.9 % USP. Record this as the study product preparation time.

4. The prepared PGT121.414.LS product may be stored at 2°C to 8°C up to 24 hours or at room temperature (maximum 27°C) for a maximum of 4 hours, including the administration time. If stored at 2°C to 8°C, prepared product must be equilibrated at room temperature (maximum 27°C) for a minimum of 30 minutes prior to product administration.

8.3.3.3 PGT121.414.LS fixed-dose intravenous infusion preparation

1. Obtain 4 vials of thawed PGT121.414.LS vials, as well as an appropriately sized IV container (bag/glass bottle) containing 100 mL of 0.9% Sodium Chloride for Injection, USP, that will also permit the addition of the required calculated volume of PGT121.414.LS.

2. Using an 18G needle, withdraw a total of 14mL of PGT121.414.LS into a sterile syringe.

3. Using aseptic technique, remove the air from the IV container and then add the withdrawn 14 mL of PGT121.414.LS to the IV container with 100

FOR PREVIEW ONLY

mL of 0.9% Sodium Chloride for Injection, USP. Record this as the study product preparation time. Assign a 4-hour expiration from this time.

4. Gently mix the prepared IV container. Ensure there are no visible particulates in the IV container.

8.3.3.4 PGT121.414.LS weight-based dose subcutaneous infusion preparation

1. Calculate the total (mg) of PGT121.414.LS required based on the participant's weight (in kg). Remove the minimum number of thawed PGT121.414.LS vials from storage.

2. Gently swirl thawed vials for 30 seconds to avoid foaming. DO NOT SHAKE VIALS. Keep the vials upright until ready to withdraw the contents. Do not invert the vial during inspection.

3. Using aseptic technique, withdraw the calculated volume of PGT121.414.LS from the vial(s) into a syringe using a 5 micron filter needle (see HVTN 140/HPTN 101 SSP for needle/filter specifications and details). A new filter needle must be used for each vial. Add the additional volume of PGT121.414.LS needed to accommodate for the residual volume left in the tubing after infusion. Discard the filter needle prior to dispensing. Record this as the study product preparation time.

4. The prepared PGT121.414.LS product may be stored at 2°C to 8°C up to 24 hours or at room temperature (maximum 27°C) for a maximum of 4 hours, including the administration time. If stored at 2°C to 8°C, prepared product must be equilibrated at room temperature (maximum 27°C) for a minimum of 30 minutes prior to product administration.

8.3.3.5 PGT121.414.LS fixed-dose subcutaneous infusion preparation

1. Remove 4 vials of thawed PGT121.414.LS from storage.

2. Gently swirl thawed vials for 30 seconds to avoid foaming. DO NOT SHAKE VIALS. Keep the vials upright until ready to withdraw the contents. Do not invert the vial during inspection.

3. Using aseptic technique, withdraw 14 mL of PGT121.414.LS from the vial(s) into a syringe using a 5 micron filter needle (see HVTN 140/HPTN 101 SSP for needle/filter specifications and details). A new filter needle must be used for each vial. Add the additional volume of PGT121.414.LS needed to accommodate for the residual volume left in the tubing after infusion. Discard the filter needle prior to dispensing. Record this as the study product preparation time.

4. The prepared PGT121.414.LS product may be stored at 2°C to 8°C up to 24 hours or at room temperature (maximum 27°C) for a maximum of 4 hours, including the administration time. If stored at 2°C to 8°C, prepared

FOR REVIEW ONLY

product must be equilibrated at room temperature (maximum 27°C) for a minimum of 30 minutes prior to product administration.

8.3.3.6 Labeling of Study Product

Label the study product as follows:

- Participant identifier(s)
- Participant weight (in kg) for weight-based dosing
- Study product name
- Total dose (mg)
- Final volume (mL)
- Route (IV or SC)
- Beyond use date and time
- Any additional information required by jurisdiction

8.4 Administration

For weight-based dosing, the container prepared by the pharmacy will include the weight that was used for preparation of the study product. The clinician responsible for administration will check the container label and confirm that the participant identifier(s) is/are correct and that the weight listed on the container label is within 10% of the participant's current actual weight.

In Groups 6-10, three separate IV containers each containing one study product will be administered sequentially.

8.4.1 General considerations for subcutaneous infusion study product administration

Due to factors such as dose and volume, participant weight and body habitus, and participant tolerability and preference, the site clinicians and participants must decide whether to subcutaneously infuse each study product at 1, 2, or 3 anatomic locations simultaneously. In Part B, three mAbs are infused sequentially; thus in Part B there are a minimum of 3 and a maximum of 9 infusion sites. The HVTN 140/HPTN 101 SSP provides information about subcutaneous infusion sets, recommended infusion pumps and clinical considerations for study product administration.

FOR REVIEW ONLY

A suitable SC infusion set (tubing and needle(s)) should be affixed to the syringe prior to administration. The SC infusion sets are carefully and gently primed with the study product just to the end of the tubing leaving the needle dry. Dry needles are less painful. The syringe will be prepared with overfill to accommodate for the residual study product left in the tubing.

Refer to the HVTN 140/HPTN 101 SSP for further information on SC administration.

8.4.2 General considerations for intravenous infusion study product administration

For all IV infusions:

- A 1.2 micron in-line filter must be used for IV product administration. Filters must comply with the specifications described in the HVTN 140/ HPTN 101 SSP.
- Once the in-line filter is added to the tubing, prime the administration set with 0.9% Sodium Chloride for Injection, USP.
- Refer to the HVTN 140/HPTN 101 SSP for further information on IV administration.

8.4.3 PGDM1400LS (Intravenous Infusion)

PGDM1400LS will be administered IV over approximately 30 to 60 minutes.

8.4.4 PGDM1400LS (Subcutaneous Infusion)

PGDM1400LS will be administered via SC infusion at a rate of approximately 15 mL/hr per infusion site for the first infusion visit. The rate may be increased up to approximately 20 mL/hr per infusion site as tolerated by the participant for the second infusion visit in Part B.

8.4.5 VRC07-523LS (Intravenous Infusion)

VRC07-523LS will be administered IV over approximately 15 to 30 minutes or more as needed using a volumetric pump.

8.4.6 VR07-523LS (Subcutaneous Infusion)

VRC07-523LS will be administered via subcutaneous infusion at a rate of approximately 15 mL/hr for the first infusion visit. The rate may be increased up to approximately 20 mL/hr per infusion site as tolerated by the participant for the second infusion visit in Part B.

FOR REVIEW ONLY

8.4.7 PGT121.414.LS (Intravenous Infusion)

PGT121.414.LS will be administered IV over approximately 30 to 60 minutes.

8.4.8 PGT121.414.LS (Subcutaneous Infusion)

PGT121.414.LS will be administered via SC infusion at a rate of approximately 15 mL/hr for the first infusion visit. The rate may be increased up to approximately 20 mL/hr per infusion site as tolerated by the participant for the second infusion visit in Part B.

8.5 Acquisition of study products

PGDM1400LS is provided by DAIDS, NIAID, NIH, DHHS (Bethesda, MD, USA).

VRC07-523LS is provided by Dale and Betty Bumpers Vaccine Research Center (VRC), NIAID, NIH, DHHS (Bethesda, MD, USA).

PGT121.414.LS is provided by Dale and Betty Bumpers Vaccine Research Center (VRC), NIAID, NIH, DHHS (Bethesda, MD, USA).

Once a CRS is protocol registered, the pharmacist can obtain study products from the NIAID Clinical Research Products Management Center (CRPMC) by following the ordering procedures outlined in Pharmacy Guidelines and Instructions for DAIDS Clinical Trials Networks.

Filter needles, in-line filters, infusion sets, infusion pump, tubing, and 0.9% Sodium Chloride for Injection, USP will be locally sourced by the site. Refer to the study product considerations section of the HVTN 140/HPTN 101 SSP for product specific reference numbers. Should it be determined by the Networks or the HVTN 140/HPTN 101 team that specialized multiple leg subcutaneous infusion needle sets and specialty tubing that controls flow rate with constant pressure system pumps need to be supplied to the sites conducting this protocol, the Network LOCs will purchase the specialized infusion sets and they will be distributed by the CRPMC or via another distribution method.

8.6 Pharmacy records

The CRS pharmacist is required to maintain complete records of all study products. The pharmacist of record is responsible for maintaining randomization codes and randomization confirmation notices for each participant in a secure manner.

FOR REVIEW ONLY

8.7 Final disposition of study products

For US clinical research sites, all unused study products must be returned to the CRPMC after the study is completed or terminated unless otherwise instructed by the study sponsor. For non-US clinical research sites, all unused study products must be destroyed after the study is completed or terminated unless otherwise instructed by the study sponsor. The procedures are included in the Pharmacy Guidelines and Instructions for DAIDS Clinical Trials Networks.

FOR REVIEW ONLY

9 Clinical procedures

The schedule of clinical procedures is shown in Appendix H and Appendix I.

Procedures are in place so that study visits may be conducted remotely, such as via phone, text message, email or other electronic means, in lieu of, or in combination with, in-person visits at the clinical research site. Furthermore, some visit procedures may be conducted outside the CRS (eg, PK sampling, see HVTN 140/HPTN 101 SSP for additional details). Direct data entry or direct data capture of study data into the study database is allowed when capturing information from the participant. Study data may also be sourced from electronic or paper source documents prior to being entered into the study database (see HVTN 140/HPTN 101 SSP).

9.1 Informed consent

Informed consent is the process of working with participants so that they fully understand what will and may happen to them while participating in a research study. The informed consent form documents that a participant (1) has been informed about the potential risks, benefits, and alternatives to participation, and (2) is willing to participate in the study. Informed consent encompasses all written or verbal study information CRS staff provide to the participant, before and during the trial. CRS staff will obtain informed consent of participants according to HVTN and HPTN policies and procedures, as well as per the CRS's SOP on the informed consent process.

The informed consent process continues throughout the study. Key study concepts should be reviewed periodically with the participant and the review should be documented. At each study visit, CRS staff should consider reviewing the procedures and requirements for that visit and for the remaining visits. Additionally, if any new information is learned that might affect the participants' decisions to stay in the trial, this information will be shared with trial participants. If necessary, participants will be asked to sign revised informed consent forms as directed by the IRB/EC.

A CRS may employ recruitment efforts prior to the participant consenting. For example, some CRSs use a telephone script to prescreen people before they come to the clinic for a full screening visit. Participants must sign a screening or protocol-specific consent before any procedures are performed to determine eligibility. CRSs must submit recruitment and prescreening materials to their IRB/EC and any applicable RE for human subjects protection review and approval.

Note: As defined in the DAIDS Protocol Registration Manual, an RE is "Any entity/body that has the power to regulate which includes authorities that review submitted clinical data and those that conduct inspections. These are sometimes referred to as competent authorities. These are entities/bodies whose

FOR REVIEW ONLY

approval/authorization/acknowledgment of a clinical trial is required for conducting a clinical trial. Any organization whose approval is required prior to a CRS's participation in DAIDS funded and/or Sponsored Clinical Trial. Includes but not limited to approvals from state/national health systems and administrative bodies, drug agencies etc. (DAIDS adopted from ICH E6)." CRSs are responsible for complying with the requirements of their applicable REs.

9.1.1 Screening consent form

Without a general screening consent, screening for a specific study cannot take place until the CRS receives protocol registration from the DAIDS RSC Protocol Registration Office.

Some CRSs have approval from their IRB/EC and any applicable RE to use a general screening consent form that allows screening for an unspecified HIV prevention clinical trial. In this way, CRS staff can continually screen potential participants and, when needed, proceed quickly to obtain protocol-specific enrollment consent. Sites conducting general screening or prescreening approved by their IRB/EC and any applicable RE may use the results from this screening to determine eligibility for this protocol, provided the tests are conducted within the time periods specified in the eligibility criteria.

9.1.2 Protocol-specific consent forms

The protocol-specific consent forms describe the study products to be used and all aspects of protocol participation, including screening and enrollment procedures. A sample protocol-specific consent forms for the main study is located in Appendix A and Appendix B. A separate sample consent form for other uses of specimens is located in Appendix D.

Each CRS is responsible for developing a protocol-specific consent form(s) for local use, based on the sample protocol-specific consent forms in Appendix A, Appendix B, and Appendix D. The consent form(s) must be developed in accordance with requirements of the following:

- CRS's IRB/EC and any applicable REs,
- CRS's institution, and
- Elements of informed consent as described in Title 45, CFR Part 46 and Title 21 CFR, Part 50, and in ICH E6(R2) Good Clinical Practice: Consolidated Guidance 4.8.

Study sites are strongly encouraged to have their local CABs review their sites-specific consent forms. This review should include, but should not be limited to, issues of cultural competence, local language considerations, and the level of understandability.

FOR REVIEW ONLY

The sample informed consent form(s) include instructions for developing specific content.

Regarding protocol registration, sites should follow procedures outlined in the current version of the DAIDS Protocol Registration Manual.

9.1.3 Assessment of Understanding

Study staff is responsible for ensuring that participants fully understand the study before enrolling them. This process involves reviewing the informed consent form with the participant, allowing time for the participant to reflect on the procedures and issues presented, and answering all questions completely.

An Assessment of Understanding is used to document the participant's understanding of key concepts in this clinical trial. The participant must complete the Assessment of Understanding before enrollment. Staff may provide assistance in reading and understanding the questions and responses, if necessary. Participants must verbalize understanding of all questions answered incorrectly. This process and the participant's understanding of the key concepts should be recorded in source documentation at the site.

IRB/EC and any applicable RE may require that a participant has signed either a screening or protocol-specific consent document prior to administering the Assessment of Understanding. The consent process (including the use of the Assessment of Understanding) should be explained thoroughly to the IRB/EC and any applicable RE, whose recommendations should be followed.

9.2 Pre-enrollment procedures

Screening may occur over the course of several contacts/visits, up to and including before study product administration on day 0. All inclusion and exclusion criteria must be assessed within 56 days before enrollment, unless otherwise specified in the eligibility criteria (or below in this section).

After the appropriate informed consent has been obtained and before enrollment, the following procedures are performed:

- Medical history, documented in the case history record;
- Assessment of whether the volunteer is at low risk for HIV infection (see Appendix J and Appendix K);
- Complete physical examination, including height, weight, vital signs, and clinical assessments of head, ears, eyes, nose, and throat; neck; lymph nodes; heart; chest; abdomen; extremities; neurological function; and skin;

FOR PREVIEW ONLY

- Assessment of concomitant medications the volunteer is taking, including prescription and nonprescription drugs, vitamins, topical products, alternative/complementary medicines (eg, herbal and health food supplements), recreational drugs, vaccinations, and allergy shots;

- Laboratory tests including:
  - Screening HIV,
  - Hepatitis B surface antigen (HBsAg),
  - Anti-HCV Abs,
  - Syphilis,
  - Complete Blood Count (CBC) with differential,
  - Chemistry panel (alanine aminotransferase [ALT], creatinine),
  - Urine dipstick (urinalyses if indicated; see Section 9.7), and
  - Urine or serum pregnancy test (volunteers who were assigned female sex at birth); persons who are not of reproductive potential due to having undergone total hysterectomy with bilateral oophorectomy (verified by medical records) are not required to undergo pregnancy testing;

- Administration of behavioral risk assessment questionnaire;

- Obtaining volunteer demographics in compliance with the NIH Policy on Reporting Race and Ethnicity Data: Subjects in Clinical Research, Aug. 8, 2001 (available at http://grants.nih.gov/grants/guide/notice-files/NOT-OD-01-053.html);

- Counseling on HIV testing and risk reduction, performed in compliance with the US Centers for Disease Control and Prevention (CDC)'s current guidelines or other local guidelines for HIV counseling, testing, and referral as described in Section 9.5; and

- Discussion of reproductive status and pregnancy prevention. A pregnant or breastfeeding person may not be enrolled in this trial. Specific criteria and assessment of contraception and reproductive status are described in study inclusion criteria. Discussion of contraception includes advising a participant who was assigned female sex at birth and who reports no current sexual activity that could lead to that participant becoming pregnant to have a plan to begin adequate birth control. This plan would be put to use if, during the study, the participant becomes sexually active in a way that could lead to that participant becoming pregnant.

9.2.1 Use of screening results from another HVTN or HPTN study

If a participant screens for an HVTN or HPTN study at the same CRS but then does not join that study, screening results from that effort may be applied to the

FOR REVIEW ONLY

screening for this protocol, as long as the screening was done under participant consent, the participant has signed a consent form to begin screening for this study, and the tests were conducted within the time periods specified in the eligibility criteria (see Sections 7.1 and 7.2).

9.3 Enrollment and study product administration visits

Once a volunteer has consented to trial participation and is found to meet all eligibility criteria (see Sections 7.1 and 7.2), the CRS requests the randomization assignment via a Web-based randomization system. Enrollment is simultaneous with first study product administration. In general, the time interval between randomization and enrollment should not exceed 4 working days. However, circumstances may require a participant's enrollment visit to be changed. This may exceed the 4-day randomization time limit.

At **all study product administration visits**, the following procedures are performed **before study product administration**:

- Abbreviated physical examination, including weight, vital signs, and a symptom-directed evaluation by history and/or appropriate physical exam based on participant self-reported symptoms or complaints;
- Assessment of baseline Solicited AEs;
- Assessment of concomitant medications (as described in Section 9.2);
- Assessment of any new or unresolved Unsolicited AEs/intercurrent illnesses; and
- Clinical laboratory tests including:
  - CBC with differential;
  - Chemistry panel (alanine aminotransferase [ALT], aspartate aminotransferase [AST], alkaline phosphatase [Alk Phos] and creatinine); and
  - Urine or serum pregnancy test (for participants who were assigned female sex at birth). Persons who are NOT of reproductive potential due to having undergone hysterectomy or bilateral oophorectomy (verified by medical records), are not required to undergo pregnancy testing. For pregnant participants, see Section 9.11.

Following completion of all procedures in the preceding list, and if available results indicate that study product administration may proceed, study product administration is administered (see Sections 8.3 and 8.4).

FOR REVIEW ONLY

Administration of all infusions during a study product administration visit must be accomplished within 1 calendar day.

Immediately following study product administration, the participant remains in the clinic for observation for about 1 hour. See the HVTN 140/HPTN 101 SSP for details regarding study product administration visit procedures. Before leaving the clinic, the participant is given the Participant Diary and is instructed on how to complete it. The CRS will make arrangements to be in contact with the participant as described in Section 9.8.

The following procedures will be performed at **all study product administration visits**. These procedures may be performed **prior to or following study product administration**:

- Risk reduction counseling (as described in Section 9.5);

- For participants capable of becoming pregnant, contraception status assessment (as described in Sections 9.2 and 9.6). In persons who are confirmed pregnant, contraception status assessment is not required; and

- Assessment of new or unresolved social impacts (CRS staff will ask participant about the status of any unresolved social impacts and if s/he has experienced any new social impacts as a result of the trial participation).

The following procedures will be performed at all infusion visits following study product administration:

- Acceptability questionnaire (see Appendix H and Appendix I); and

- Drug concentrations/detection collection (see Appendix F and Appendix G).

Additional procedures will be performed at scheduled visits as specified in Appendix H and Appendix I:

- HIV infection assessment including pretest counseling and HIV testing. A subsequent follow-up contact is conducted to provide post-test counseling and to report results to participant;

- Confirm that participants received HIV test results from previous visit. If not, provide test results and post-test counseling as appropriate; and

- Specimen collection (should be completed per Appendix F and Appendix G,).

9.3.1 Managing Antibody reactions

Since PGDM1400LS, VRC07-523LS, and PGT121.414.LS are human mAbs, rather than murine or chimeric mAbs, that target a viral antigen, rather than human cell surface antigens, serious Ab reactions are expected to be rare.

FOR REVIEW ONLY

Nevertheless, participants will be closely monitored in the clinic during study product administration and during a post study-product-administration observation period.

CRS staff are trained to recognize suspected Ab reactions and to provide immediate medical care consistent with the HVTN 140/HPTN 101 SSP. Medications used to treat Ab reactions may include acetaminophen, antihistamines, and/or corticosteroids. CRSs are also equipped with additional emergency medical supplies to provide other immediate medical intervention, if indicated, and are near medical emergency services. Should the need arise, CRSs may transfer the participant, once stabilized, to a tertiary care center for further management.

The following procedures should be performed after an Ab reaction:

- Ab reaction clinical assessment; and
- Ab reaction blood collection.

For detailed management of Ab reactions see the HVTN 140/HPTN 101 SSP.

9.4 Follow-up visits

The following procedures are performed at **scheduled follow-up visits** as specified in Appendix H and Appendix I:

- Risk reduction counseling (as described in Section 9.5);
- For participants capable of becoming pregnant, contraception status assessment (as described in Sections 9.2 and 9.6). In persons who are confirmed pregnant, contraception status assessment is not required;
- Assessment of new or unresolved social impacts (CRS staff will ask participant about the status of any unresolved social impacts and if s/he has experienced any new social impacts as a result of the trial participation);
- Assessment of new or continuing concomitant medications (as described in Section 9.2);
- Assessment of new or unresolved AEs/intercurrent illnesses;
- Administration of the social impact assessment questionnaire (types of impacts assessed involve personal relationships, health insurance, life insurance, educational or employment opportunities, housing, immigration, or travel);

FOR REVIEW ONLY

- Administration of a questionnaire that asks the participant about any HIV testing they may have received outside of the study;
- HIV infection assessment including pretest counseling and HIV testing. A subsequent follow-up contact is conducted to provide post-test counseling and to report results to participant;
- Confirm that participants received HIV test results from previous visit. If not, provide test results and post-test counseling as appropriate;
- Abbreviated physical examination including weight, vital signs, and a symptom-directed evaluation by history and/or appropriate physical exam based on participant self-reported symptoms or complaints;
- Complete physical examination, including weight, vital signs, and clinical assessments of head, ears, eyes, nose, and throat; neck; lymph nodes; heart; chest; abdomen; extremities; neurological function; and skin;
- Specimen collection (should be completed per Appendix F and Appendix G,); and
- Clinical laboratory tests including:
  - CBC with differential;
  - Chemistry panel (alanine aminotransferase [ALT], aspartate aminotransferase [AST], alkaline phosphatase [Alk Phos] and creatinine);
  - Urine dipstick (urinalysis if appropriate; see Section 9.7); and
  - Urine or serum pregnancy test (for participants who were assigned female sex at birth). Persons who are NOT of reproductive potential due to having undergone hysterectomy or bilateral oophorectomy (verified by medical records), are not required to undergo pregnancy testing. During follow-up in persons who are confirmed pregnant, contraception status assessment is not required, unless clinically indicated.

9.4.1 Interim contacts

CRSs may report safety information obtained at a contact other than the regularly scheduled visits. These contacts are reported as interim visits.

9.5 HIV counseling and testing

HIV counseling will be performed in compliance with the CDC's guidelines or other local guidelines for HIV counseling and referral. HIV testing will be performed in accordance with the protocol-specific HIV testing algorithm following enrollment.

FOR REVIEW ONLY

Participants will be counseled routinely during the trial on the avoidance of HIV infection.

Potential participants identified during screening as having acquired HIV are not enrolled. Potential and enrolled participants identified as being HIV-infected will be referred for medical treatment, counseling, and management of the HIV infection. With respect to enrolled participants who acquire HIV during the study, see Section 9.12.

It is theoretically possible that an anti-HIV mAb may suppress viral replication, which can reduce the ability to detect HIV infection, even if a person has actually acquired HIV.

An anti-HIV mAb is not likely to directly affect the assays used to detect HIV-1 acquisition.

9.5.1 Monoclonal antibody-associated reactivity

Tests of human plasma containing anti-HIV mAbs have been conducted using a variety of commercially available HIV test kits. At high plasma concentrations, reactive or indeterminate results have been observed on some test kits. See the HVTN 140/HPTN 101 SSP for further detail. Thus, there is a possibility that receipt of the study product will cause a reactive result on some currently available HIV test kits, especially if testing occurs close to study product administration timepoints.

Study staff will advise study participants to confine their HIV testing while in the study to that provided through the CRS. Staff will also inform study participants of the likelihood of routine HIV testing being offered or performed outside the study CRS at emergency rooms, clinics, and medical offices, and will inform participants of their right to opt out of HIV testing outside the study site. CRS staff should inform study participants if local and/or state/regional policies and regulations permit medical providers to perform HIV testing without first informing patients. If this is the case, then CRS staff should advise study participants that they may decline testing preemptively. CRS staff should also inform participants if positive results must be reported to local public health authorities. CRS staff should provide participants with CRS contact information and should encourage participants to ask medical providers to contact the CRS. The CRS can verify that the participant is in an HIV mAb clinical trial and should only be tested at the study CRS.

9.6 Contraception status

Contraception status is assessed and documented at clinic visits indicated in Appendix H and Appendix I for a participant who was assigned female sex at birth and who is sexually active in a way that could cause that participant to become pregnant. Prior to enrollment and throughout the study, staff will ask

FOR REVIEW ONLY

participants to verbally confirm their use of adequate contraceptive methods. A participant who was assigned female sex at birth and is sexually active in a way that could cause that participant to become pregnant should be reminded at all scheduled clinic visits of the importance of using contraception and should be referred to specific counseling, information, and advice as needed. (Specific contraception requirements are listed in Section 7.1). This reminder should be documented in the participant's study record.

Self-reported infertility—including having reached menopause (no menses for 1 year) or having undergone hysterectomy, or bilateral oophorectomy—must be documented in the participant's study record.

9.7 Urine testing

Dipstick testing must include the following required elements: glucose, protein, and hemoglobin. The examination is performed on urine obtained by clean catch.

If the screening dipstick is transiently abnormal due to non-urinary bleeding (eg, menstruation) or infection, document this issue in the participant's source documentation. For infection, provide appropriate treatment and/or referral and document this in the participant's chart. Following resolution, repeat the dipstick and, if within the eligibility limits specified in the protocol, the participant may be enrolled.

Follow-up visit dipstick testing should be deferred if a participant is experiencing non-urinary bleeding (eg, menstruation) but should be performed as soon as possible. If a follow-up visit dipstick is abnormal due to a participant's non-urinary bleeding (eg, menstruation) document in the comment section of the case report form (CRF)' and repeat the dipstick once the participant is no longer experiencing non-urinary bleeding. A micro-urinalysis is not required. If a follow-up visit dipstick or micro-urinalysis is abnormal due to infection, provide appropriate treatment and/or referral and document this in the participant's source documentation. See the Urinalysis Sample Collection, Interpretation, Management, and Reporting section of the Ab Manual of Operations (MOP) for further details.

9.8 Assessments of Solicited AEs

For all participants, baseline assessments are performed before, and Solicited AE assessments are performed after each study product administration. All Solicited AEs are graded according to the Division of AIDS Table for Grading the Severity of Adult and Pediatric Adverse Events, Corrected Version 2.1, July 2017, except as noted in Section 11.2.2. See HVTN 140/HPTN 101 SSP for further details.

The Solicited AE assessment period is 3 full days following each study product administration per the assessment schedule shown in Table 9-1. Participants are

FOR REVIEW ONLY

instructed to record symptoms using a Participant Diary. The CRS staff and the participant will be in contact after the 3-day Solicited AE assessment period, or sooner if indicated. In general, a participant who self-reports any post study product administration reactions greater than mild is seen by a clinician within 48 hours after onset, unless the reaction is improving and/or has completely resolved. Clinic staff will follow new or unresolved Solicited AEs present at day 3 to resolution.

Solicited AEs are reported using CRFs that correspond to the time of assessment in Table 9-1. Solicited AE assessments include assessments of systemic and local symptoms, and study product-related lesions. Events not listed on a CRF, or with an onset after the Solicited AE assessment period (day of study product administration and 3 full days after), or those meeting SAE/Unsolicited AEs requiring expedited reporting according to DAIDS criteria, are recorded on an AE Log CRF.

Table 9-1 Schedule of Solicited AE assessments

Day	Time	Performed by
0[a]	Baseline: before study product administration	CRS clinician
	Early: 25-60 minutes after study product administration	CRS clinician
	Between early assessment and 11:59pm day 0	CRS clinician or participant
1-3[b]	Between 12:00am and 11:59pm on the respective day	CRS clinician or participant

[a] Day of study product administration

[b] New or unresolved Solicited AEs present on day 3 are followed until resolution

9.8.1 Assessment of systemic and local symptoms

Systemic symptoms to be assessed as Solicited AEs in this trial include increased body temperature, malaise and/or fatigue, myalgia, headache, chills, arthralgia, nausea, urticaria, non-exertional dyspnea, non-exertional tachycardia (assessed by CRS staff, not by the participant), generalized pruritus, facial flushing, and unexplained diaphoresis. Local symptoms include pain and/or tenderness at the infusion site. Additionally, in participants receiving SC injection, local pruritus will be assessed. The daily maximum severity reached for each symptom during the assessment period is reported.

Body temperature is measured by oral or infrared thermometry. All temperatures must be measured by non-axillary thermometry. This includes temperatures taken in the clinic, as well as temperatures taken by participants during the Solicited AE period.

Temperature is reported in degrees Celsius. If temperature is measured in Fahrenheit, the conversion to Celsius should be documented in the participant's chart note. A measurement is taken once daily during the assessment period and should be repeated if participant is feeling feverish.

FOR REVIEW ONLY

9.8.2 Assessment of IV or SC infusion site

Typical infusion site reactions are erythema/redness and induration/swelling. The maximum diameter measurement for all infusion site reactions is recorded.

All infusion site reactions are monitored until resolution. Reactions with areas with diameters ≥ 5 cm are followed daily; otherwise, the frequency of follow-up is based on clinician judgment. See HVTN140/HPTN101 SSP for details.

9.9 Visit windows and missed visits

Visit windows are shown in Appendix L. The procedures for documenting missed visits and out of window visits are described in HVTN140/HPTN101 SSP.

If a participant misses a scheduled visit, the CRS staff should either attempt to bring the participant in to the CRS or conduct the visit remotely as soon as possible to complete the required safety assessments and other procedures. See the SSP for more details.

If a missed visit required study product administration or if study product administration must be permanently discontinued, please refer to Section 7.3.2 and Section 7.3.3 for resolution.

9.10 Early termination visit

In the event of early participant termination, CRS staff should attempt to complete the following assessments, as appropriate: a final physical examination, clinical laboratory tests (including urine dipstick, CBC with differential, and chemistry panel (see Section 7.1), pregnancy testing (note: for persons who are confirmed pregnant, pregnancy testing is not required, unless clinically indicated), social impact assessment, and HIV test. For participants who have a confirmed diagnosis of HIV infection, see Section 9.12.

9.11 Pregnancy

If a participant becomes pregnant during the course of the study, no more infusions of study product will be given, but remaining visits and study procedures should be completed unless medically contraindicated or applicable regulations require termination from the study. During follow-up in persons who are confirmed pregnant, pregnancy testing is not required, unless clinically indicated. If the participant terminates from the study prior to the pregnancy outcome, the CRS should make every effort to keep in touch with the participant in order to ascertain the pregnancy outcome. Pregnancies and pregnancy outcomes will be reported. If the participant is no longer pregnant, refer to Section 7.3.1.

FOR REVIEW ONLY

See Pregnancy Management and Reporting section of the Ab MOP for further details.

9.12 HIV infection during the study

If a participant becomes HIV-infected during the course of the study, no additional study product will be administered. Participants will be encouraged to continue scheduled study visits for up to 24 weeks following their last study product administration. Follow-up duration for participants diagnosed with HIV infection may be adjusted in consultation with the CRS investigator and the HVTN 140/HPTN 101 PSRT (eg, to avoid interference with participant initiation of HIV treatment). At post-infection follow-up visits, only specimens required for protocol-specified safety laboratory tests, urinalysis, and pregnancy tests will be collected (note: for persons who are confirmed pregnant, pregnancy testing is not required, unless clinically indicated); in addition, some clinic procedures may be modified or discontinued (see Appendix F and Appendix G). These individuals may also be referred to appropriate ongoing clinical trials or observational studies.

See the HIV Infection section in the Ab MOP for further details.

FOR REVIEW ONLY

10 Laboratory

10.1 CRS laboratory procedures

The HVTN 140/HPTN 101 Site Lab Instructions and SSP provide further guidelines for operational issues concerning the clinical and processing laboratories. These documents include guidelines for general specimen collection, special considerations for phlebotomy, specimen labeling, whole blood processing, HIV screening/diagnostic testing, and general screening and safety testing.

Tube types for blood collection are specified in Appendix F and Appendix G. For tests performed locally, the local lab may assign appropriate tube types.

In specific situations, the blood collection tubes may be redirected to another laboratory or may require study-specific processing techniques. In these cases, laboratory special instructions will be posted on the protocol-specific section of the HVTN website.

Of note, all assays described below are performed as research assays and are not approved for use in medical care. Results from these assays are not made available to participants or medical professionals to guide treatment decisions.

10.2 Total blood volume

Required blood volumes per visit are shown in Appendix F and Appendix G. Not shown is any additional blood volume that would be required if a safety lab needs to be repeated, or if a serum pregnancy test needs to be performed. The additional blood volume would likely be minimal. The total blood volume drawn for each participant will not exceed 500 mL in any 56-day (8-week) period.

10.3 PGDM1400LS, VRC07-523LS, and PGT121.414.LS concentrations

PGDM1400LS, VRC07-523LS and PGT121.414.LS concentrations will be measured in serum collected at prespecified timepoints. A quantitative immunoassay will be used to determine the concentration of each mAb. Ultra-sensitive bead-based analyses enable a broad dynamic range and higher sensitivity (eg, for the anti-idiotype mAb, 5C9, the lower limit of quantification is approximately 50 pg/mL). The operational sensitivity of the quantitative assays will be determined for the clinical grade PGDM1400LS, VRC07-523LS and PGT121.414.LS used for this study. For multiplexed PK measurements, interference testing will be included as part of the qualification/validation. The mAb concentrations may be normalized relative to total protein.

FOR REVIEW ONLY

10.4 Neutralizing antibody assay

HIV-1–specific nAb assays will be performed on serum samples from study participants taken at post administration timepoint(s) and at baseline. The TZM-bl assay will test neutralization of mAb-specific viruses (one virus per mAb). The assay will also test the neutralization of a panel of viruses that exhibit a range of known sensitivities to PGDM1400LS, VRC07-523LS and PGT121.414.LS. The viruses will be selected from a global and/or clade-specific panel (57, 61) and may be modified by site-directed mutagenesis to be capable of measuring the activity of each mAb individually.

10.5 ADA detection assays

A tiered testing approach will be used to identify and characterize ADAs that may arise (62, 63). Anti-PGDM1400LS, -VRC07-523LS, and -PGT121.414.LS antibody detection assays (screening, confirmatory, and/or titration) will be performed on serum samples from study participants at indicated timepoints. Samples will be evaluated with a sensitive screening assay in Tier 1. Samples showing positive responses in the screening assay will be evaluated in a confirmatory assay of specificity. Specific or Tier 2 positive responses will be characterized by titration (Tier 3).

10.6 ADA functional assay

A functional ADA assay will be used to characterize any positive activity that is observed in the ADA detection assays. Functional activity will measure a reduction in PGDM1400LS, VRC07-523LS and/or PGT121.414.LS neutralizing activity against a qualified virus in the TZM-bl assay.

10.7 Antibody reaction assays

To investigate Ab reactions, serum samples collected after the onset of reaction may be tested to measure levels of certain markers [eg, tryptase, complement components (C3 and C4), and cytokines]. ADA detection and functional assays, as described above, may be performed on serum samples taken prior to the study product administration associated with the reaction. Refer to the HVTN 140/HPTN 101 SSP for more information.

10.8 HVTN Laboratory Center assay portfolio

Additional assays may be performed per the HVTN Laboratory Center assay portfolio, which includes immune assessments such as those for cellular, humoral, and innate immune responses, and host genetics. The assay portfolio will be

FOR REVIEW ONLY

updated periodically to include new assays and adjust qualification levels of existing assays.

10.9 Exploratory studies

Samples may be used for other testing and research related to furthering the understanding of HIV immunology, antibody mediated prevention, or vaccines. In addition, samples may be used to perform additional assays to support standardization and validation of existing or newly developed methods.

10.10 Specimen storage and other use of specimens

The Networks store specimens from all study participants indefinitely, unless a participant requests that specimens be destroyed or if destruction or a time limit for storage is required by IRB/EC, or RE.

Other use of specimens is defined as studies not covered by the protocol or the informed consent forms for the main study (see Appendix A and Appendix B).

This research may relate to HIV, vaccines, antibodies, the immune system, and other diseases. This could include genetic testing and, potentially, genome-wide studies. This research is done only to the extent authorized in each study site's informed consent form, or as otherwise authorized under applicable law. Other research on specimens ("other use") will occur only after review and approval by the HVTN, the HPTN, the IRB/EC of the researcher requesting the specimens, and IRBs/ECs/REs of the CRS's if required.

As part of consenting for the study, participants document their initial decision to allow or not allow their specimens to be used in other research, and they may change their decision at any time. The participant's initial decision about other use of their specimens, and any later change to that decision, is recorded by their CRS in a Web-based tool that documents their current decisions for other use of their specimens. The Networks will only allow other research to be done on specimens from participants who allow such use.

CRSs must notify HVTN Regulatory Affairs if institutional or local governmental requirements pose a conflict with or impose restrictions on specimen storage or other use of specimens.

10.11 Biohazard containment

As the transmission of HIV and other blood-borne pathogens can occur through contact with contaminated needles, blood, and blood products, appropriate precautions will be employed by all personnel in the drawing of blood and

FOR REVIEW ONLY

shipping and handling of all specimens for this study, as currently recommended by the CDC and the NIH or other applicable agencies.

All dangerous goods materials, including Biological Substances, Category A or Category B, must be transported according to instructions detailed in the International Air Transport Association Dangerous Goods Regulations.

FOR REVIEW ONLY

11 Safety monitoring and safety review

11.1 Safety monitoring and oversight

11.1.1 HVTN 140/HPTN 101 PSRT

The HVTN 140/HPTN 101 PSRT is composed of the following members:

- DAIDS medical officer representatives
- Protocol chairs
- Protocol Team leaders
- Core medical monitor
- Clinical safety specialist
- Regional medical liaison

The clinician members of the HVTN 140/HPTN 101 PSRT are responsible for decisions related to participant safety.

The Protocol Team clinic coordinator, clinical data manager, study product developer representative, clinical trial manager, clinical research manager, and others may also be included in HVTN 140/HPTN 101 PSRT meetings.

11.1.2 HVTN SMB

The SMB is a multidisciplinary group consisting of biostatisticians, clinicians, and experts in HIV vaccine and drug research that, collectively, has experience in the conduct and monitoring of vaccine, mAb, and other drug trials. Members of the SMB are not directly affiliated with the protocols under review.

The SMB reviews safety data approximately every 4 months. The reviews consist of evaluation of cumulative Solicited AEs, Unsolicited AEs, laboratory safety data, and individual reports of adverse events requiring expedited reporting to DAIDS. The SMB conducts additional special reviews at the request of the HVTN 140/HPTN 101 PSRT.

Study sites will receive SMB summary minutes and are responsible for forwarding them to their IRB/EC and any applicable RE.

11.1.3 Roles and responsibilities in safety monitoring

The roles and responsibilities of the SDMC in relation to safety monitoring include:

FOR REVIEW ONLY

- Maintaining a central database management system for clinical data; and
- Providing reports of clinical data to appropriate groups such as the HVTN 140/HPTN 101 PSRT and HVTN SMB (see Section 11.1.2).

The roles and responsibilities of the HVTN CSS or HVTN Core designee in relation to safety monitoring include:

- Daily monitoring of clinical data for events that meet the safety pause and HVTN 140/HPTN 101 PSRT AE review criteria (see Section 11.4);
- Notifying CRSs and other groups when safety pauses or planned holds are instituted and lifted (see Sections 11.3 and 11.4);
- Querying CRSs for additional information regarding reported clinical data; and
- Providing support to the HVTN 140/HPTN 101 PSRT.

11.2 Safety reporting

11.2.1 Submission of safety forms to SDMC.

CRS staff must submit all safety forms (eg, Solicited AEs, Unsolicited AEs, urinalysis, local lab results, and concomitant medications) before the end of the next business day, excluding federal or bank holidays. The forms should not be held in anticipation of additional information at a later date. If additional information is received at a later date, the forms should be updated and resubmitted before the end of the next business day after receiving the new information. For the case of a longer CRS holiday closure, CRS staff must submit the data by the end of the 5th day (local time) after receiving the information even if this day is a holiday.

For example: If the CRS becomes aware of an AE on Thursday (Day 0, the first day), the CRS must submit the data by the end of the next business day, on Friday (Day 1). If there is a longer CRS holiday closure, then this AE must be reported no later than the end of day, Monday (Day 4). If Monday is a holiday as well, all safety forms still need to be submitted by the end of Monday (Day 4).

11.2.2 AE reporting

An AE is any untoward medical occurrence in a clinical investigation participant administered a study product/procedure(s) and which does not necessarily have a causal relationship with this treatment. An AE can therefore be any unfavorable and unintended sign (including an abnormal laboratory finding), symptom, or disease temporally associated with the use of an investigational study product/procedure(s), whether or not related to the investigational study

FOR REVIEW ONLY

product/procedure(s). AEs include both solicited AEs and unsolicited AEs. Solicited AEs are a subset of AEs that are defined per protocol and specifically asked about for 3 full days after each study infusion. Unsolicited AEs include all other AEs that do not fit the definition of a solicited AE. See the SSP for further detail regarding Solicited and Unsolicited AEs.

The Unsolicited AE reporting period for this study comprises the entire study period for each individual participant (from study enrollment until study completion or discontinuation of the study).

All AEs are graded according to the Division of AIDS (DAIDS) Table for Grading the Severity of Adult and Pediatric Adverse Events, Corrected Version 2.1, July 2017, available on the RSC website at https://rsc.niaid.nih.gov/clinical-research-sites/daids-adverse-event-grading-tables, except:

- Unintentional Weight Loss is required to be reported as an AE only if it is considered to be potentially deleterious to the participant's health (see HVTN 140/HPTN 101 SSP);

- Infusion Site Erythema or Redness and Infusion Site Induration or Swelling will not consider surface area and interference with usual social and functional activities such that:
  - Grade 1 is: 2.5 to < 5 cm in diameter;
  - Grade 2 is: ≥ 5 to < 10 cm in diameter;
  - Grade 3 is: ≥ 10 cm in diameter OR Ulceration OR Secondary infection OR Phlebitis OR Sterile abscess OR Drainage;
  - Grade 4 is: Potentially life-threatening consequences (eg, abscess, exfoliative dermatitis, necrosis involving dermis or deeper tissue);

- Creatinine is required to be reported as an AE only if it is gradable per the increase from local lab ULN/reference range parameter. Do not grade elevated creatinine based on the change from the baseline parameter.

- Creatinine clearance or eGFR is required to be reported based only on the reported value or if dialysis is needed. Do not grade Creatinine clearance or eGFR based on the change from the baseline parameter.

- Ab reactions not represented in the DAIDS Table to be graded per the "infusion related reaction" row from the Common Terminology Criteria for Adverse Events (CTCAE) from the US DHHS (Version 5.0. Published November 27, 2017, available at https://ctep.cancer.gov/protocoldevelopment/electronic_applications/docs/ctcae_v5_quick_reference_5x7.pdf (see also HVTN 140/HPTN 101 SSP).

All AEs are reported to the SDMC on the appropriate CRF. Clinic staff should evaluate every AE to determine if: (1) the AE meets the requirements for

FOR REVIEW ONLY

expedited reporting to DAIDS (see Section 11.2.3); (2) if the AE meets the criteria for a safety pause/prompt AE review (see Section 11.4).

Sites are expected to notify HVTN clinical safety staff of any serious safety concern requiring their attention (Table 11-2). Telephone numbers and email addresses are found on the protocol home page on the HVTN Members' site (https://members.hvtn.org/protocols/hvtn140-hptn101). Concerns requiring immediate attention should be communicated by calling the clinical safety phone.

In the case of email notification, clinical safety staff will reply within one business day. Serious events that meet pause rule criteria will be addressed immediately (as outlined in Table 11-2) . If email service is not available, the CRS should notify clinical safety staff of the event by telephone, and then submit CRFs.

In addition, CRS investigators are required to submit AE information in accordance with IRB/EC and any applicable RE requirements.

11.2.3 Expedited reporting of adverse events to DAIDS

Requirements, definitions and methods for expedited reporting of AEs are outlined in Version 2.0 (January 2010) of the *Manual for Expedited Reporting of Adverse Events* to DAIDS (DAIDS EAE Manual), which is available on the DAIDS RSC website at https://rsc.niaid.nih.gov/clinical-research-sites/manual-expedited-reporting-adverse-events-daids. The SAE Reporting Category, as defined in Version 2.0 of DAIDS EAE Manual, will be used for this study.

The internet-based DAIDS Adverse Experience Reporting System (DAERS) must be used for expedited AE (EAE) reporting to DAIDS. In the event of system outages or technical difficulties, expedited AE reports may be submitted via the DAIDS EAE form. This form is available on the DAIDS RSC website at https://rsc.niaid.nih.gov/clinical-research-sites/paper-eae-reporting.

For questions about DAERS, please contact CRMSsupport@niaid.nih.gov or from within the DAERS application itself.

For questions about EAE reporting, please contact the DAIDS RSC Safety Office at (DAIDSRSCSafetyOffice@tech-res.com).

The study products for which expedited reporting are required are:

- PGDM1400LS
- PGT121.414.LS
- VRC07-523LS

FOR REVIEW ONLY

While the participant is in the study, from enrollment to the end of trial participation for that participant, the SAE Reporting Category will be used.

After the end of trial participation for that participant, unless otherwise noted, only Suspected, Unexpected Serious Adverse Reactions (SUSARs) as defined in Version 2.0 of the DAIDS EAE Manual must be reported to DAIDS, if the study staff become aware of the events.

The NIAID/DAIDS will report all unexpected SAEs related to the study products observed in this clinical trial to the FDA in accordance with 21 CFR 312.32 (IND Safety Reports). In addition, the NIAID/DAIDS or designee(s) will prepare and file expedited reports to other appropriate regulatory authorities within the timelines required by pertinent national regulatory agencies.

CRS Investigators of Record (IoRs)/designees will submit AE information and any other relevant safety information to their IRBs/ECs in accordance with IRB/EC requirements.

In some cases, the PSRT or CRS may believe unblinding of the CRS PI and participant would be appropriate to facilitate the clinical management of an AE or SAE. The Ab MOP specifies procedures for emergency unblinding, and for early unblinding for medical reasons.

11.3 Safety reviews

11.3.1 Safety Considerations for Part A dose escalation

The HVTN 140/HPTN 101 PSRT will monitor participant safety data throughout the study period as described in this section. The PSRT will review safety data for all groups to determine if enrollment may commence in subsequent groups for dose escalation. In addition, the HVTN 140/HPTN 101 PSRT will consider all available safety data on the antibodies in this study, including from ongoing and concurrent clinical trials (eg, HVTN 136/HPTN 092), in determining the appropriateness of opening HVTN 140/HPTN 101 groups to enrollment throughout the course of the trial.

The trial will begin with enrollment in Part A Group 1 only. Groups 2 and 3 will be enrolled simultaneously; then groups 4 and 5 will be enrolled simultaneously. Additional participants may be enrolled in each group to ensure the availability of safety data from at least 3 participants in each group.

Enrollment will start with Group 1 and will be restricted to a maximum of 1 participant per day across all participating CRSs until a total of 3 participants have been enrolled. Enrollment will then be held. The HVTN 140/HPTN 101 PSRT will review all cumulative safety data reported, including Group 1 data through the Day 6 visit post study product administration. Upon PSRT

FOR REVIEW ONLY

determination that it is safe to proceed, enrollment in both Group 2 and Group 3 will begin (see Table 11-1).

If any ≥ Grade 3 AE(s) deemed related to study product are reported in Group 1, the HVTN SMB will perform an additional unblinded review of Group 1 safety data to make the final determination based on safety for proceeding to Groups 2 and 3 enrollment.

Groups 2 and 3 will be enrolled simultaneously and will be restricted to a maximum of 1 participant per day in either group across all participating CRSs until a total of 6 participants have been enrolled. Enrollment will then be held. The HVTN 140/HPTN 101 PSRT will review all cumulative safety data reported, including Groups 2 and 3 data through the Day 6 visit post study product administration. Upon PSRT determination that it is safe to proceed, enrollment in both Group 4 and Group 5 will begin (see Table 11-1).

If any ≥ Grade 3 AE(s) deemed related to study product are reported in Group 2 or 3, the HVTN SMB will perform an additional unblinded review of Groups 2 and 3 safety data to make the final determination based on safety for proceeding to Groups 4 and 5 enrollment.

Groups 4 and 5 will be enrolled simultaneously and will be restricted to a maximum of 1 participant per day in either group across all participating CRSs until a total of 6 participants have been enrolled. Enrollment will then be held.

11.3.2 Safety evaluation for moving from Part A to Part B

In addition to monitoring participant safety throughout the study period, the HVTN 140/HPTN 101 PSRT will review all cumulative safety data available from Groups 1 through 5 reported through the Day 6 visit after study product administration. Based on the assessment of this safety data and all available safety data on the antibodies in this study, including from ongoing and concurrent clinical trials, the HVTN 140/HPTN 101 PSRT will make a decision regarding the appropriateness of moving to Part B. Additional participants may be enrolled in each group in Part A to ensure the availability of safety data from at least 3 participants in each group. If any ≥ Grade 3 AE(s) deemed related to study product are reported in Part A, the HVTN SMB will perform an additional unblinded review of this safety data to make the final determination based on safety for proceeding to Part B.

11.3.3 Safety Considerations for Part B dose escalation

Part B will begin with enrollment in Groups 6, 7, 8 and 9 simultaneously. Group 10 will enroll last. Additional participants may be enrolled in each group to ensure the availability of safety data from at least 8 participants in Groups 6 through 9 together; or at least 3 participants in Group 10.

FOR REVIEW ONLY

Enrollment in Part B begins with simultaneous enrollment into Groups 6 through 9 and will be restricted to a maximum of 1 participant per day in any group across all participating CRSs for the first 8 participants (at least 2 participants in each group). Enrollment will then be held. The HVTN 140/HPTN 101 PSRT will review all cumulative safety data available, including Groups 6 through 9 reported through the Day 6 visit post study product administration. Upon PSRT determination that it is safe to proceed, enrollment of the remaining participants in Groups 6 through 9 will reopen to enrollment without restrictions and enrollment into Group 10 will begin (see Table 11-1). If any ≥ Grade 3 AE(s) deemed related to study product have been reported in Groups 6 through 9, the HVTN SMB will perform an additional unblinded review of Groups 6 through 9 safety data to make the final determination based on safety for continuing enrollment and subsequent product administrations in Groups 6 through 9 without restrictions and proceeding to Group 10 enrollment.

Group 10 will be restricted to a maximum of 1 participant per day across all participating CRSs for the first 3 participants. Enrollment will then be held. The HVTN 140/HPTN 101 PSRT will review all cumulative safety data reported, including Group 10 data through the Day 6 visit post study product administration. Upon PSRT determination that it is safe to proceed, enrollment of the remaining participants in Group 10 will reopen to enrollment without restrictions (see Table 11-1). If any ≥ Grade 3 AE(s) deemed related to study product have been reported in Group 10, the HVTN SMB will perform an additional unblinded review of Group 10 safety data to make the final determination based on safety for continuing enrollment and subsequent product administrations in Group 10 without restrictions.

Table 11-1 Summary of planned safety holds and safety data reviews for dose escalations

Planned Safety Hold #	Group(s)	Timepoint/Data Reviewed	Action
**Part A**[a]			
1	1	Begins after the 3rd participant is enrolled (infused) and the solicited AE assessment data through the Day 6 visit is reported.<br>Review of all cumulative safety data available for the 3 participants in Group 1 up to and including the solicited AE assessment data reported at the Day 6 visit after the study product administration.	The PSRT (+/- SMB) will decide the appropriateness of beginning enrollment in Groups 2 and 3 based on these safety data.
2	2 & 3	Begins after the 6th participant is enrolled (infused) in either Group 2 or 3 and the solicited AE assessment data through the Day 6 visit is reported (3 participants in Group 2 and 3 participants in Group 3).<br>Review of all cumulative safety data available, including the first 6 participants in Groups 2 and 3 up to and including the solicited AE assessment data reported at the Day 6 visit after the study product administration.	The PSRT (+/- SMB) will decide the appropriateness of beginning enrollment in Groups 4 and 5 based on these safety data.

FOR REVIEW ONLY

Planned Safety Hold #	Group(s)	Timepoint/Data Reviewed	Action
3	4 & 5	Begins after the 6th participant is enrolled (infused) and the solicited AE assessment data through the Day 6 visit is reported (3 participants each in Groups 4 and 5).<br>Review of all cumulative safety data available for all participants in Part A and the first 6 participants in Groups 4 and 5 up to and including the solicited AE assessment data reported at the Day 6 visit after the product administration.	The PSRT (+/- SMB) will decide the appropriateness of beginning enrollment in Part B based on all available safety data from all participants included in Part A.
**Part B[a]**			
4	6, 7, 8 and 9	Begins after the 8th participant is enrolled (infused) and the solicited AE assessment data through the Day 6 visit is reported (at least 2 participants in Groups 6 through Group 9).<br>Review of all cumulative safety data available, including the first 8 participants in Groups 6 through Group 9 up to and including the solicited AE assessment data reported at the Day 6 visit after the first study product administration.	The PSRT (+/- SMB) will decide the appropriateness of continuing enrollment and subsequent product administrations in Groups 6 through 9 without restrictions, and beginning enrollment into Group 10 based on all available safety data.
5	10	Begins after the 3rd Group 10 participant is enrolled (infused) and the solicited AE assessment data through the Day 6 visit is reported.<br>Review of all cumulative safety data available, including the first 3 participants in Group 10 up to and including the solicited AE assessment data reported at the Day 6 visit after the first study product administration.	The PSRT (+/- SMB) will decide the appropriateness of continuing enrollment into Group 10 without restrictions based on these safety data.

[a]Additional participants may be enrolled in each group to ensure the availability of safety data from at least 3 participants in each group.

11.4 Safety pause and prompt PSRT AE review

When a trial is placed on safety pause, all enrollment and study product administration with the product related to the event that triggered the pause will be held until further notice. The AEs that will lead to a safety pause or prompt HVTN 140/HPTN 101 PSRT AE review are summarized in Table 11-2. Study product administrations may be suspended for safety concerns other than those described in the table, or before pause rules are met, if, in the judgment of the HVTN 140/HPTN 101 PSRT, participant safety may be threatened. Criteria for an individual participant's departure from the schedule of study product administrations are listed in Section 7.3.

FOR REVIEW ONLY

Table 11-2 AE notification and safety pause/AE review rules

Event and relationship to study products	Severity	CRS action[a]	HVTN LOC action [b]
SAE, related	Grade 5 or Grade 4	Phone immediately, email and submit forms immediately	Immediate pause
SAE, not related	Grade 5	Phone immediately, email and submit forms immediately	Immediate PSRT notification
SAE, related	Grade 3, 2, or 1	Email and submit forms immediately	Immediate PSRT notification and prompt PSRT AE review to consider pause
AE[c], related	Grade 4 or 3	Email and submit forms immediately	Immediate PSRT notification and prompt PSRT AE review to consider pause

[a] Phone numbers and email addresses are found on the Protocol home page on the HVTN Members' site (https://members.hvtn.org/protocols/hvtn140-hptn101).

b HVTN CSS or HVTN Core designee

c Does not include subjective Solicited AEs (infusion site pain and/or tenderness, fatigue/malaise, myalgia, arthralgia, chills, headache, nausea (unless IV rehydration required), non-exertional dyspnea, generalized pruritus, local pruritus [SC infusion only], facial flushing, and unexplained diaphoresis).

For all safety pauses, HVTN LOC notifies the HVTN 140/HPTN 101 PSRT, HVTN Regulatory Affairs, DAIDS Pharmaceutical Affairs Branch (PAB), DAIDS Regulatory Affairs Branch (RAB), DAIDS Safety and Pharmacovigilance Team (SPT), and participating CRSs. When an immediate safety pause is triggered, HVTN LOC notifies the SMB.

Once a trial is paused, the HVTN 140/HPTN 101 PSRT reviews safety data and decides whether the pause can be lifted or permanent discontinuation of study product administration is appropriate, consulting the SMB if necessary. HVTN LOC notifies the participating CRSs, HVTN Regulatory Affairs, DAIDS PAB, DAIDS RAB, and DAIDS SPT of the decision regarding resumption or discontinuation of study product administrations. Based on the HVTN 140/HPTN 101 PSRT assessment, DAIDS RAB notifies the FDA as needed.

If an immediate HVTN 140/HPTN 101 PSRT notification or prompt HVTN 140/HPTN 101 PSRT AE review is triggered, HVTN Core notifies the HVTN 140/HPTN 101 PSRT as soon as possible during working hours (local time)—or, if the information was received during off hours, by the morning of the next workday. If a prompt HVTN 140/HPTN 101 PSRT AE review cannot be completed within 72 hours of notification (excluding weekends and US federal holidays), an automatic safety pause occurs.

The HVTN and HPTN require that each CRS submit to its IRB/EC and any applicable RE protocol-related safety information (such as IND safety reports, notification of study-product holds due to the pause rules, unanticipated problems involving risks to participants or others, and notification of other unplanned safety pauses). CRSs must also follow all applicable RE reporting requirements.

FOR REVIEW ONLY

In addition, all other AEs are reviewed routinely by the HVTN 140/HPTN 101 PSRT (see Section 11.5.2).

11.5 Review of cumulative safety data

Routine safety review occurs at the start of enrollment and then throughout the study.

Reviews proceed from a standardized set of protocol-specific safety data reports. These reports are produced by the SDMC and include queries to the CRSs. Events are tracked by internal reports until resolution.

11.5.1 Daily review

Daily safety reviews are routinely conducted by HVTN Core for events requiring expedited reporting to DAIDS, and events that meet safety pause criteria or prompt HVTN 140/HPTN 101 PSRT AE review criteria.

11.5.2 Weekly review

During the study product administration phase of the trial, the HVTN 140/HPTN 101 PSRT reviews clinical safety reports on a weekly basis and conducts calls to review the data as appropriate. Following the visit 8 weeks post-final study product administration, less frequent reporting and safety reviews may be conducted at the discretion of the HVTN 140/HPTN 101 PSRT. HVTN LOC reviews reports of clinical and laboratory AEs. Events identified during the review that are considered questionable, inconsistent, or unexplained are referred to the CRS clinic coordinator for verification.

11.6 Study termination

NIAID reserves the right to terminate or curtail a clinical study for any reason, including but not limited to the following (reference: https://grants.nih.gov/grants/guide/rfa-files/RFA-AI-12-012.html):

- risk to subject safety
- the scientific question is no longer relevant or the objectives will not be met (ie, slow accrual)
- failure to comply with GCP, U.S. Federal regulations, or Terms and Conditions of Award
- occurrence of unforeseen drug safety issues or data from preclinical studies indicate a presence of unanticipated toxicity

FOR REVIEW ONLY

- risks that cannot be adequately quantified
- ethical concerns raised by the local community or local medical care/health care authorities
- failure to remedy deficiencies identified through site monitoring
- substandard data
- reaching a major study endpoint substantially before schedule with persuasive statistical significance.

This study may also be terminated early by the determination of the HVTN 140/HPTN 101 PSRT, FDA, a pertinent national regulatory authority, NIH, Office for Human Research Protections (OHRP), or study product developer(s). In addition, the conduct of this study at an individual CRS may be terminated by the determination of the IRB/EC and any applicable RE.

FOR REVIEW ONLY

12 Protocol conduct

This protocol and all actions and activities connected with it will be conducted in compliance with the principles of GCP (ICH E6(R2)), and according to DAIDS, HVTN and HPTN policies and procedures as specified in the network-specific Manuals of Operations, DAIDS Clinical Research Policies, and Standard Procedures Documents including procedures for the following:

- Protocol registration, activation, and implementation;
- Informed consent, screening, and enrollment;
- Study participant reimbursement;
- Clinical and safety assessments;
- Safety monitoring and reporting;
- Data collection, documentation, transfer, and storage;
- Participant confidentiality;
- Study follow-up and close-out;
- Quality control;
- Protocol monitoring (on-site or remote) and compliance;
- Advocacy and assistance to participants regarding negative social impacts associated with the trial;
- Risk reduction counseling;
- Specimen collection, processing, and analysis;
- Exploratory and ancillary studies and sub-studies, and
- Destruction of specimens.

DAIDS, HVTN and HPTN policies and procedures are available for review by any IRB/EC/RE upon request.

Any policies or procedures that vary from DAIDS, HVTN, or HPTN standards or require additional instructions (eg, instructions for randomization specific to this study) will be described in the HVTN 140/HPTN 101 SSP.

FOR REVIEW ONLY

12.1 Social impacts

It is possible that participants' involvement in the study could result in social impacts. Social harms are negative social impact events and social benefits are positive social impact events that a participant reports as affecting them as a result of being involved in a research study. It is not the researcher's opinion of how they perceive an event has affected a participant. For example, a participant's involvement in the study could become known to others, and a social harm may result (eg, because participants could be perceived as being HIV infected or at "high risk" for HIV infection). Participants could be treated unfairly or could have problems being accepted by their families and/or communities. Alternatively, a social benefit may result (eg, a participant could feel good helping others).

Social impacts will be collected and reported on CRFs during scheduled visits (see Appendix H and Appendix I). Social harms are tabulated by the SDMC and are subjected to descriptive analysis. The goal is to reduce their incidence and enhance the ability of study staff to mitigate them when possible. Summary tables of social impact events will be generated weekly, and made available for review by the protocol chairs, protocol team leader, and the designated NIAID representative. A social harm that is reported by the participant and judged by the IoR/designee to be serious or unexpected will be reported to the responsible site's IRB at least annually, or according to their individual requirements.

In the event that a participant reports a social harm, every effort will be made by study staff to provide appropriate care and counseling to the participant as necessary, and/or referral to appropriate resources for the safety and wellbeing of the participant. If CRS staff have questions regarding ways to assist a participant dealing with a social impact, a designated NIAID or Network Core representative can be contacted. While maintaining participant confidentiality, study sites may engage their CAB in exploring the social context surrounding instances of social harms to minimize the potential occurrence of such an impact.

12.2 Emergency communication with study participants

As in all clinical research, this study may generate a need to reach participants quickly to avoid imminent harm, or to report study findings that may otherwise concern their health or welfare.

When such communication is needed, the CRS will request that its IRB/EC and any applicable RE expedite review of the message. If this review cannot be completed in a timeframe consistent with the urgency of the required communication, the CRS can contact the participant without IRB/EC approval if such communication is necessary to avoid imminent harm to the study participant. The CRS must notify the IRB/EC and any applicable RE of the matter as soon as possible.

FOR REVIEW ONLY

13 Version history

The Protocol Team may modify the original version of the protocol. Modifications are made to Network protocols via clarification memos, letters of amendment, or full protocol amendments.

The version history of, and modifications to, Protocol HVTN 140/HPTN 101 are described below.

Protocol history and modifications

Date: June 23, 2021

Protocol version: 1.0
Protocol modification:

Original protocol

FOR REVIEW ONLY

14 Document references (other than literature citations)

Other documents referred to in this protocol, and containing information relevant to the conduct of this study, include:

- Assessment of Understanding. Accessible through the HVTN protocol-specific website.
- Current Centers for Disease Control (CDC) Guidelines.
  - Revised Recommendations for HIV Testing of Adults, Adolescents, and Pregnant Women in Health-Care Settings. Available at http://www.cdc.gov/mmwr/PDF/rr/rr5514.pdf.
  - Revised Guidelines for HIV Counseling, Testing, and Referral. Available at http://www.cdc.gov/mmwr/preview/mmwrhtml/rr5019a1.htm
- Division of AIDS (DAIDS) Clinical Research Policies and Standard Procedures Documents. Available at https://www.niaid.nih.gov/research/daids-clinical-research-policies-standard-procedures
- Division of AIDS Protocol Registration Manual. Available at https://www.niaid.nih.gov/sites/default/files/prmanual.pdf
- Division of AIDS Table for Grading the Severity of Adult and Pediatric Adverse Events. Corrected Version 2.1, July 2017. Available at https://rsc.niaid.nih.gov/clinical-research-sites/daids-adverse-event-grading-tables
- The Manual for Expedited Reporting of Adverse Events to DAIDS. Version 2.0, January 2010. Available at https://rsc.niaid.nih.gov/clinical-research-sites/manual-expedited-reporting-adverse-events-daids
- Common Terminology Criteria for Adverse Events (CTCAE), Version 5.0. Published November 27, 2017. Available at https://ctep.cancer.gov/protocoldevelopment/electronic_applications/docs/ctcae_v5_quick_reference_5x7.pdf
- HVTN Certificate of Confidentiality. Accessible through the HVTN website.
- HPTN Certificate of Confidentiality.
- HVTN 140/HPTN 101 Special Instructions. Accessible through the HVTN protocol-specific website.

FOR REVIEW ONLY

- HVTN 140/HPTN 101 Study Specific Procedures. Accessible through the HVTN protocol-specific website.

- HVTN 140/HPTN 101 Site Lab Instructions. Accessible through the HVTN protocol-specific website.

- Ab Manual of Operations. Accessible through the HVTN website.

- Dangerous Goods Regulations (updated annually), International Air Transport Association. Available for purchase at https://www.iata.org/publications/dgr/Pages/index.aspx

- Lab assay algorithm (available upon request)

- International Council on Harmonisation of Technical Requirements for Pharmaceuticals for Human Use (ICH) E6, Guideline for Good Clinical Practice: Section 4.8, Informed consent of trial subjects. Available at http://www.ich.org/products/guidelines/efficacy/article/efficacy-guidelines.html

- Participants' Bill of Rights and Responsibilities. Accessible through the HVTN website.

- National Institutes of Health (NIH) Policy on Reporting Race and Ethnicity Data: Subjects in Clinical Research. Available at https://grants.nih.gov/grants/guide/notice-files/NOT-OD-01-053.html

- Pharmacy Guidelines and Instructions for DAIDS Clinical Trials Networks, July 2008.

- Requirements for Source Documentation in DAIDS Funded and/or Sponsored Clinical Trials. Available at https://www.niaid.nih.gov/sites/default/files/score-source-documentation-requirements.pdf

- Title 21, Code of Federal Regulations, Part 50. Available at http://www.accessdata.fda.gov/scripts/cdrh/cfdocs/cfcfr/CFRSearch.cfm?CFRPart=50

- Title 45, Code of Federal Regulations, Part 46. Available at https://www.hhs.gov/ohrp/regulations-and-policy/regulations/45-cfr-46/index.html

- The Protection of Personal Information Act (POPIA, South Africa, 2013). Available at https://www.gov.za/sites/default/files/gcis_document/201409/3706726-11act4of2013protectionofpersonalinforcorrect.pdf

FOR REVIEW ONLY

See Section 16 for literature cited in the background and statistics sections of this protocol.

FOR REVIEW ONLY

15 Acronyms and abbreviations

Ab	antibody
ADA	anti-drug antibodies
ADCC	antibody dependent cell mediated cytotoxicity
ADCP	antibody dependent cellular phagocytosis
AE	adverse event
Alk Phos	alkaline phosphatase
ALT	alanine aminotransferase
AMP	Antibody Mediated Prevention
ANOVA	analysis of variance
ART	antiretroviral therapy
AST	aspartate aminotransferase
AUC	area-under-the-curve
AUC-MB	area-under-the-magnitude-breadth curve
BAMA	binding antibody multiplex assay
β-HCG	beta human chorionic gonadotropin
BIDMC	Beth Israel Deaconess Medical Center
bnAb	broadly neutralizing HIV-1 antibody
CAB	Community Advisory Board
CAVD	Collaboration for AIDS Vaccine Discovery
CBC	complete blood count
CDC	US Centers for Disease Control and Prevention
CFR	Code of Federal Regulations
cGMP	current Good Manufacturing Practice
CL	clearance
Cmax	maximum concentration
CMIA	chemiluminescent microparticle immunoassay
CRF	case report form
CRPMC	NIAID Clinical Research Products Management Center
CRS	clinical research site
CSS	Clinical Safety Specialist
CTCAE	Common Terminology Criteria for Adverse Events
DAERS	DAIDS Adverse Experience Reporting System
DAIDS	Division of AIDS (US NIH)
DHHS	US Department of Health and Human Services
EAE	adverse events requiring expedited reporting to DAIDS
EC	Ethics Committee
EIA	enzyme immunoassay
ELISA	enzyme-linked immunosorbent assay

FOR REVIEW ONLY

Env	HIV envelope protein
Fc	Fragment crystallizable
FcRn	Fc-receptor
FDA	US Food and Drug Administration
FIH	first-in-human
Fred Hutch	Fred Hutchinson Cancer Research Center
GCP	Good Clinical Practice
GEE	generalized estimating equation
GLP	Good Laboratory Practice
HBsAg	hepatitis B surface antigen
HCV	hepatitis C virus
HPTN	HIV Prevention Trials Network
HVTN	HIV Vaccine Trials Network
IAVI	International AIDS Vaccine Initiative
IB	Investigator's Brochure
IC	inhibitory concentration
ICF	informed consent form
ICH	International Council on Harmonisation of Technical Requirements for Pharmaceuticals for Human Use
IgG	immunoglobulin G
IM	intramuscular
IND	Investigational New Drug
IoR	Investigator of Record
IRB	Institutional Review Board
IUD	intrauterine device
IV	intravenous
LOC	Leadership Operations Center
mAb	monoclonal antibody
MAR	missing at random
M-B	Magnitude-Breadth
MCAR	missing completely at random
MITT	modified intent-to-treat
MOP	Manual of Operations
MTD	maximum tolerated dose
nAb	neutralizing antibody
NAEPP	National Asthma Education and Prevention Program
NHP	nonhuman primate
NIAID	National Institute of Allergy and Infectious Diseases (US NIH)
NIH	US National Institutes of Health

FOR REVIEW ONLY

NOAEL	no-observed-adverse-effect-level
NSAID	non-steroidal anti-inflammatory drugs
OHRP	US Office for Human Research Protections
PAB	DAIDS Pharmaceutical Affairs Branch
PCR	polymerase chain reaction
PD	pharmacodynamics
PK	pharmacokinetic
POPIA	Protection of Personal Information Act
popPK	population PK (pharmacokinetic)
PP	per-protocol
PrEP	pre-exposure prophylaxis
PSRT	Protocol Safety Review Team
RAB	DAIDS Regulatory Affairs Branch
RE	regulatory entity
RSC	DAIDS Regulatory Support Center
RSV	respiratory syncytial virus
SAE	serious adverse event
SAP	Statistical Analysis Plan
SC	subcutaneous
SCHARP	Statistical Center for HIV/AIDS Research and Prevention
SD	standard deviation
SDMC	statistical and data management center
SHIV	simian-human immunodeficiency virus
SMB	Safety Monitoring Board
SOP	standard operating procedure
SPT	DAIDS Safety and Pharmacovigilance Team
SSP	Study Specific Procedures
SUSAR	sudden unexpected serious adverse reaction
TB	tuberculosis
TCR	tissue cross reactivity
Tmax	time to Cmax
USP	United States Pharmacopeia
UW-VSL	University of Washington Virology Specialty Laboratory
VCMP	Vaccine Clinical Materials Program
Vd	volume of distribution
VRC	Vaccine Research Center (NIAID)
VRP	Vaccine Research Program
VTRB	Vaccine Translational Research Branch

FOR REVIEW ONLY

16 Literature cited

1. UNAIDS. Ethical considerations in biomedical HIV prevention trials. 2007 7/2007.

2. The National Commission for the Protection of Human Subjects of Biomedical and Behavioral Research. The Belmont Report: Ethical Principles and Guidelines for the Protection of Human Subjects of Research. 1979 4/18/1979.

3. Council for International Organizations of Medical Sciences (CIOMS). International ethical guidelines for biomedical research involving human subjects. Bull Med Ethics. 2002(182):17-23.

4. UNAIDS. UNAIDS Data 2018 2018 [Available from: http://www.unaids.org/en/resources/documents/2018/unaids-data-2018.

5. Centers for Disease Control and Prevention. HIV Surveillance Report, Volume 28 2016 [updated 2016. Available from: https://www.cdc.gov/hiv/pdf/library/reports/surveillance/cdc-hiv-surveillance-report-2016-vol-28.pdf.

6. Cohen YZ, Caskey M. Broadly neutralizing antibodies for treatment and prevention of HIV-1 infection. Curr Opin HIV AIDS. 2018;13(4):366-73.

7. Caskey M, Klein F, Lorenzi JC, Seaman MS, West AP, Jr., Buckley N, et al. Viraemia suppressed in HIV-1-infected humans by broadly neutralizing antibody 3BNC117. Nature. 2015.

8. Barouch DH, Whitney JB, Moldt B, Klein F, Oliveira TY, Liu J, et al. Therapeutic efficacy of potent neutralizing HIV-1-specific monoclonal antibodies in SHIV-infected rhesus monkeys. Nature. 2013;503(7475):224-8.

9. Hessell AJ, Poignard P, Hunter M, Hangartner L, Tehrani DM, Bleeker WK, et al. Effective, low-titer antibody protection against low-dose repeated mucosal SHIV challenge in macaques. Nat Med. 2009;15(8):951-4.

10. Moldt B, Rakasz EG, Schultz N, Chan-Hui PY, Swiderek K, Weisgrau KL, et al. Highly potent HIV-specific antibody neutralization in vitro translates into effective protection against mucosal SHIV challenge in vivo. Proc Natl Acad Sci U S A. 2012;109(46):18921-5.

11. Rudicell RS, Kwon YD, Ko SY, Pegu A, Louder MK, Georgiev IS, et al. Enhanced potency of a broadly neutralizing HIV-1 antibody in vitro improves protection against lentiviral infection in vivo. J Virol. 2014;88(21):12669-82.

FOR REVIEW ONLY

12. Mascola JR, Lewis MG, Stiegler G, Harris D, VanCott TC, Hayes D, et al. Protection of Macaques against pathogenic simian/human immunodeficiency virus 89.6PD by passive transfer of neutralizing antibodies. J Virol. 1999;73(5):4009-18.

13. Parren PW, Marx PA, Hessell AJ, Luckay A, Harouse J, Cheng-Mayer C, et al. Antibody protects macaques against vaginal challenge with a pathogenic R5 simian/human immunodeficiency virus at serum levels giving complete neutralization in vitro. J Virol. 2001;75(17):8340-7.

14. Pegu A, Yang ZY, Boyington JC, Wu L, Ko SY, Schmidt SD, et al. Neutralizing antibodies to HIV-1 envelope protect more effectively in vivo than those to the CD4 receptor. Sci Transl Med. 2014;6(243):243ra88.

15. Lynch RM, Boritz E, Coates EE, DeZure A, Madden P, Costner P, et al. Virologic effects of broadly neutralizing antibody VRC01 administration during chronic HIV-1 infection. Sci Transl Med. 2015;7(319):319ra206.

16. Saunders KO, Pegu A, Georgiev IS, Zeng M, Joyce MG, Yang ZY, et al. Sustained Delivery of a Broadly Neutralizing Antibody in Nonhuman Primates Confers Long-Term Protection against Simian/Human Immunodeficiency Virus Infection. J Virol. 2015;89(11):5895-903.

17. Bar KJ, Sneller MC, Harrison LJ, Justement JS, Overton ET, Petrone ME, et al. Effect of HIV Antibody VRC01 on Viral Rebound after Treatment Interruption. N Engl J Med. 2016;375(21):2037-50.

18. Scheid JF, Horwitz JA, Bar-On Y, Kreider EF, Lu CL, Lorenzi JC, et al. HIV-1 antibody 3BNC117 suppresses viral rebound in humans during treatment interruption. Nature. 2016;535(7613):556-60.

19. Caskey M, Schoofs T, Gruell H, Settler A, Karagounis T, Kreider EF, et al. Antibody 10-1074 suppresses viremia in HIV-1-infected individuals. Nat Med. 2017;23(2):185-91.

20. Bar-On Y, Gruell H, Schoofs T, Pai JA, Nogueira L, Butler AL, et al. Safety and antiviral activity of combination HIV-1 broadly neutralizing antibodies in viremic individuals. Nat Med. 2018.

21. Mendoza P, Gruell H, Nogueira L, Pai JA, Butler AL, Millard K, et al. Combination therapy with anti-HIV-1 antibodies maintains viral suppression. Nature. 2018;561(7724):479-84.

22. Hessell AJ, Rakasz EG, Poignard P, Hangartner L, Landucci G, Forthal DN, et al. Broadly neutralizing human anti-HIV antibody 2G12 is effective in protection against mucosal SHIV challenge even at low serum neutralizing titers. PLoS Pathog. 2009;5(5):e1000433.

FOR REVIEW ONLY

23. Walker LM, Huber M, Doores KJ, Falkowska E, Pejchal R, Julien JP, et al. Broad neutralization coverage of HIV by multiple highly potent antibodies. Nature. 2011;477(7365):466-70.

24. Stephenson KE, Barouch DH. Broadly Neutralizing Antibodies for HIV Eradication. Curr HIV/AIDS Rep. 2016;13(1):31-7.

25. Haynes BF, McElrath MJ. Progress in HIV-1 vaccine development. Curr Opin HIV AIDS. 2013;8(4):326-32.

26. Burton DR, Mascola JR. Antibody responses to envelope glycoproteins in HIV-1 infection. Nat Immunol. 2015;16(6):571-6.

27. Sok D, van Gils MJ, Pauthner M, Julien JP, Saye-Francisco KL, Hsueh J, et al. Recombinant HIV envelope trimer selects for quaternary-dependent antibodies targeting the trimer apex. Proc Natl Acad Sci U S A. 2014;111(49):17624-9.

28. Julg B, Liu PT, Wagh K, Fischer WM, Abbink P, Mercado NB, et al. Protection against a mixed SHIV challenge by a broadly neutralizing antibody cocktail. Sci Transl Med. 2017;9(408).

29. Scheid JF, Mouquet H, Ueberheide B, Diskin R, Klein F, Oliveira TY, et al. Sequence and structural convergence of broad and potent HIV antibodies that mimic CD4 binding. Science. 2011;333(6049):1633-7.

30. Zalevsky J, Chamberlain AK, Horton HM, Karki S, Leung IW, Sproule TJ, et al. Enhanced antibody half-life improves in vivo activity. Nat Biotechnol. 2010;28(2):157-9.

31. Ko SY, Pegu A, Rudicell RS, Yang ZY, Joyce MG, Chen X, et al. Enhanced neonatal Fc receptor function improves protection against primate SHIV infection. Nature. 2014;514(7524):642-5.

32. Julien JP, Sok D, Khayat R, Lee JH, Doores KJ, Walker LM, et al. Broadly neutralizing antibody PGT121 allosterically modulates CD4 binding via recognition of the HIV-1 gp120 V3 base and multiple surrounding glycans. PLoS Pathog. 2013;9(5):e1003342.

33. Sok D, Doores KJ, Briney B, Le KM, Saye-Francisco KL, Ramos A, et al. Promiscuous glycan site recognition by antibodies to the high-mannose patch of gp120 broadens neutralization of HIV. Sci Transl Med. 2014;6(236):236ra63.

34. Jardine J, Julien JP, Menis S, Ota T, Kalyuzhniy O, McGuire A, et al. Rational HIV immunogen design to target specific germline B cell receptors. Science. 2013;340(6133):711-6.

FOR REVIEW ONLY

35. Wu X, Yang ZY, Li Y, Hogerkorp CM, Schief WR, Seaman MS, et al. Rational design of envelope identifies broadly neutralizing human monoclonal antibodies to HIV-1. Science. 2010;329(5993):856-61.

36. Wu X, Wang C, O'Dell S, Li Y, Keele BF, Yang Z, et al. Selection pressure on HIV-1 envelope by broadly neutralizing antibodies to the conserved CD4-binding site. J Virol. 2012;86(10):5844-56.

37. Pegu A, Borate B, Huang Y, Pauthner MG, Hessell AJ, Julg B, et al. A Meta-analysis of Passive Immunization Studies Shows that Serum-Neutralizing Antibody Titer Associates with Protection against SHIV Challenge. Cell Host Microbe. 2019;26(3):336-46 e3.

38. Corey L, Gilbert P, Juraska M, Montefiori D, Morris L, Karuna S, et al. Two Randomized Trials of Neutralizing Antibodies to Prevent HIV-1 Acquisition. N Engl J Med. 2021;384(11):1003-14.

39. Huang Y, Zhang L, Ledgerwood J, Grunenberg N, Bailer R, Isaacs A, et al. Population pharmacokinetics analysis of VRC01, an HIV-1 broadly neutralizing monoclonal antibody, in healthy adults. MAbs. 2017:1-9.

40. Bajaj G, Suryawanshi S, Roy A, Gupta M. Evaluation of covariate effects on pharmacokinetics of monoclonal antibodies in oncology. Br J Clin Pharmacol. 2019;85(9):2045-58.

41. Davda JP, Dodds MG, Gibbs MA, Wisdom W, Gibbs J. A model-based meta-analysis of monoclonal antibody pharmacokinetics to guide optimal first-in-human study design. MAbs. 2014;6(4):1094-102.

42. Huang Y, Naidoo L, Zhang L, Carpp LN, Rudnicki E, Randhawa A, et al. Pharmacokinetics and predicted neutralisation coverage of VRC01 in HIV-uninfected participants of the Antibody Mediated Prevention (AMP) trials. EBioMedicine. 2021;64:103203.

43. Li J, Nikanjam M, Cunningham CK, McFarland EJ, Coates EE, Houser KV, et al. Model Informed Development of VRC01 in Newborn Infants Using a Population Pharmacokinetics Approach. Clin Pharmacol Ther. 2021;109(1):184-92.

44. Wang DD, Zhang S, Zhao H, Men AY, Parivar K. Fixed dosing versus body size-based dosing of monoclonal antibodies in adult clinical trials. J Clin Pharmacol. 2009;49(9):1012-24.

45. Bai S, Jorga K, Xin Y, Jin D, Zheng Y, Damico-Beyer LA, et al. A guide to rational dosing of monoclonal antibodies. Clin Pharmacokinet. 2012;51(2):119-35.

FOR REVIEW ONLY

46. Davies A, Berge C, Boehnke A, Dadabhoy A, Lugtenburg P, Rule S, et al. Subcutaneous Rituximab for the Treatment of B-Cell Hematologic Malignancies: A Review of the Scientific Rationale and Clinical Development. Adv Ther. 2017;34(10):2210-31.

47. Mayer KH, Seaton KE, Huang Y, Grunenberg N, Isaacs A, Allen M, et al. Safety, pharmacokinetics, and immunological activities of multiple intravenous or subcutaneous doses of an anti-HIV monoclonal antibody, VRC01, administered to HIV-uninfected adults: Results of a phase 1 randomized trial. PLoS Med. 2017;14(11):e1002435.

48. Xu L, Pegu A, Rao E, Doria-Rose N, Beninga J, McKee K, et al. Trispecific broadly neutralizing HIV antibodies mediate potent SHIV protection in macaques. Science. 2017;358(6359):85-90.

49. Gaudinski MR, Coates EE, Houser KV, Chen GL, Yamshchikov G, Saunders JG, et al. Safety and pharmacokinetics of the Fc-modified HIV-1 human monoclonal antibody VRC01LS: A Phase 1 open-label clinical trial in healthy adults. PLoS Med. 2018;15(1):e1002493.

50. Betts A, Keunecke A, van Steeg TJ, van der Graaf PH, Avery LB, Jones H, et al. Linear pharmacokinetic parameters for monoclonal antibodies are similar within a species and across different pharmacological targets: A comparison between human, cynomolgus monkey and hFcRn Tg32 transgenic mouse using a population-modeling approach. MAbs. 2018;10(5):751-64.

51. Petkova SB, Akilesh S, Sproule TJ, Christianson GJ, Al Khabbaz H, Brown AC, et al. Enhanced half-life of genetically engineered human IgG1 antibodies in a humanized FcRn mouse model: potential application in humorally mediated autoimmune disease. Int Immunol. 2006;18(12):1759-69.

52. Roopenian DC, Christianson GJ, Sproule TJ. Human FcRn transgenic mice for pharmacokinetic evaluation of therapeutic antibodies. Methods Mol Biol. 2010;602:93-104.

53. Gaudinski MR, Houser KV, Doria-Rose NA, Chen GL, Rothwell RSS, Berkowitz N, et al. Safety and pharmacokinetics of broadly neutralising human monoclonal antibody VRC07-523LS in healthy adults: a phase 1 dose-escalation clinical trial. Lancet HIV. 2019;6(10):e667-e79.

54. Hansel TT, Kropshofer H, Singer T, Mitchell JA, George AJ. The safety and side effects of monoclonal antibodies. Nat Rev Drug Discov. 2010;9(4):325-38.

55. Vogel WH. Infusion reactions: diagnosis, assessment, and management. Clin J Oncol Nurs. 2010;14(2):E10-E21.

FOR REVIEW ONLY

56. Agresti A, Coull BA. Approximate is better than "exact" for interval estimation of binomial proportions. Am Stat. 1998;52(2):119-26.

57. DeCamp A, Hraber P, Bailer RT, Seaman MS, Ochsenbauer C, Kappes J, et al. Global panel of HIV-1 Env reference strains for standardized assessments of vaccine-elicited neutralizing antibodies. J Virol. 2014;88(5):2489-507.

58. Huang Y, Gilbert P, Montefiori D, Self S. Simultaneous evaluation of the magnitude and breadth of a left- and right-censored multivariate response, with application to HIV vaccine development. Statistics in Biopharmaceutical Research. 2009;1:81-91.

59. Little RJA, Rubin DB. Statistical Analysis with Missing Data. 2nd ed. New York, NY: John Wiley & Sons; 2002.

60. Ibrahim JG, Chu H, Chen MH. Missing data in clinical studies: issues and methods. J Clin Oncol. 2012;30(26):3297-303.

61. Seaman M, Janes H, Hawkins N, Grandpre L, Devoy C, Giri A, et al. Tiered Categorization of a Diverse Panel of HIV-1 Env Pseudoviruses for Assessment of Neutralizing Antibodies. J Virol. 2010;84(3):1439-52.

62. Bharadwaj P, Riekofski C, Lin S, Seaman MS, Garber DA, Montefiori D, et al. Implementation of a three-tiered approach to identify and characterize anti-drug antibodies raised against HIV-specific broadly neutralizing antibodies. J Immunol Methods. 2020;479:112764.

63. Cohen YZ, Butler AL, Millard K, Witmer-Pack M, Levin R, Unson-O'Brien C, et al. Safety, pharmacokinetics, and immunogenicity of the combination of the broadly neutralizing anti-HIV-1 antibodies 3BNC117 and 10-1074 in healthy adults: A randomized, phase 1 study. PLoS One. 2019;14(8):e0219142.

FOR REVIEW ONLY

Appendix A Sample informed consent form for Part A

Title: A phase 1 dose-escalation clinical trial to evaluate the safety, tolerability, and pharmacokinetics of PGDM1400LS alone and in combination with VRC07-523LS and PGT121.414.LS in healthy, HIV-uninfected adult participants

Protocol number: HVTN 140/HPTN 101

Site: [Insert site name]

Thank you for your interest in our research study. Please read this consent form or ask someone to read it to you. If you decide to join the study, we will ask you to sign or make your mark on this form. We will offer you a copy to keep. We will ask you questions to see if we have explained everything clearly. You can also ask us questions about the study.

Research is not the same as treatment or medical care. The purpose of a research study is to answer scientific questions.

Key Information

These are some of the things you should know about this study:

- The purpose of this part of the study is to understand how the body's immune system responds to a new lab-made antibody against HIV. We also want to see if the way the antibody is given affects the immune response.We will also look at whether the antibody is safe to give to people and does not make them too uncomfortable.
- If you choose to join, we will ask you to come to several study visits over about 6 months. At one of these visits you will get the study antibody in a vein (by IV infusion) or under the skin (by subcutaneous infusion, called SC). We will take blood from you at each study visit to see how your body responds to the study antibody and how much antibody is in your blood. We will test you for HIV and other sexually transmitted infections (STIs) and pregnancy (if applicable). We will ask you to complete questionnaires and you will have physical exams.
- Some general risks of antibodies include fever, chills, shaking, nausea, vomiting, pain, headache, dizziness, fatigue, flushing, diarrhea, trouble breathing, high or low blood pressure, itchiness and rash. There may be other side effects that we don't yet know about, even serious ones. Because this is the first time this study antibody is being given to people, we do not know what all of the risks may be. We think that the risks will be similar to these general risks.
- There is no direct benefit to you from being in the study.
- Whether to take part in this study is your choice. You do not have to take part in the study and you are free to stop at any time.

FOR REVIEW ONLY

- The rest of this form provides a more complete description of this study. Please read it carefully.

About the study

The HIV Vaccine Trials Network (HVTN), the HIV Prevention Trials Network (HPTN), and [Insert site name] are doing a study to test a combination of different antibodies against HIV. HIV is the virus that causes AIDS. Antibodies are made by the body as one way to respond to or fight infection. Researchers can also make antibodies in laboratories and give them to people by infusions into a vein or under the skin. We will tell you more about these procedures below. Antibodies have been used successfully to prevent or treat other health problems, such as COVID-19 caused by SARS-CoV-2 infection, and respiratory infections in babies caused by RSV (respiratory syncytial virus).

About 95 people will take part in this study at multiple clinics. The researcher in charge of this study at this clinic is [Insert name of site PI]. The US National Institutes of Health (NIH) is paying for the study.

There are 2 parts of this study, Part A and Part B. About 15 people will take part in Part A of this study to test one study antibody. After we see the results from Part A, we will decide whether or not to do Part B of the study, that will test a combination of 3 antibodies. If we decide to do Part B, 80 more people will join.

You are being invited to join Part A of the study.

1. **We are doing this part of the study to answer several questions.**

   - Is the new study antibody safe to give to people by itself at different doses?
   - Are people able to take the new study antibody without becoming too uncomfortable?
   - How do people's immune systems respond to the new study antibody? (Your immune system protects you from disease.)
   - Does the method of giving the new study antibody change the body's response?
   - How much of the antibody remains in the body as time passes?

2. **The study antibody cannot give you HIV.**

   The study antibody is not made from actual HIV. It is impossible for the study antibody to give you HIV. Also, it cannot cause you to give HIV to someone else.

FOR REVIEW ONLY

We do not know if the study antibody will decrease, increase, or not change your risk of getting HIV if you are exposed to the virus.

3. **This study antibody is experimental.**

The study antibody is called PGDM1400LS. From here on, we will call it the study antibody. It is an experimental HIV prevention product. That means we do not know if it will be safe to use in people, or if it will work to prevent HIV infection. This antibody is used only in research studies.

The antibody was developed by the National Institute of Allergy and Infectious Diseases (NIAID).

This study antibody has not been given to people before. An earlier version of the study antibody called PGDM1400 has been given to 53 people in 3 other studies. One of these studies is still ongoing, but so far there have been no safety concerns. Nine people have been given PGDM1400 alone and 44 have been given PGDM1400 in combination with other antibodies. The new version of the study antibody used in this study has been changed so that it will last longer in the body.

Antibodies given to a person usually do not last in the body more than a few months. One of the goals of this study is to see how long this antibody will stay in the body. We don't know yet how long it will last, but we think it may be several months.

Risks of the study antibody:

This section lists the side effects we know about. There may be others that we don't yet know about, even serious ones. We will tell you if we learn about any new side effects.

Because this is the first time the antibody is being given to people, we do not know what all of the risks may be. We think that the risks will be similar to the general risks described below.

General risks of antibodies:

Other kinds of antibodies used for other health problems have caused fever, chills, itchiness, rash, hives, redness in cheeks and neck, lip or face swelling, nausea, vomiting, pain, diarrhea, fatigue, flushing, headache, dizziness, shaking, trouble breathing, high or low blood pressure, racing heartbeat, and chest pain. These reactions may be related to how an antibody is made, what it targets, or how fast the antibody product is given. At times the side effects include reactions at the injection site (pain, redness, bruising, swelling, hardening). Most side effects happen within the first 24 hours.

Rarely, some antibodies for other diseases have caused 2 kinds of side effects that may be life-threatening:

FOR REVIEW ONLY

- Anaphylaxis – a physical reaction that may include hives or rash, mouth and face swelling, low blood pressure, and trouble breathing, possibly leading to low blood oxygen. This may occur soon after getting an antibody.

- Serum Sickness – a physical reaction that includes hives or rash, fever, big lymph nodes, muscle and joint pain, chest discomfort and shortness of breath. This may occur several days to a few weeks after getting an antibody.

These rare side effects have not been seen in other studies with similar experimental antibodies against HIV. Please tell us if you have ever had a reaction similar to anaphylaxis or serum sickness.

Rarely, antibodies approved for treatment of other diseases have been linked to other effects like cancer, increased bleeding, increased infections, heart and liver toxicity, and problems with blood clotting and immune response. These rare side effects and reactions have not been seen in other studies with similar experimental antibodies against HIV.

Joining the study

4. **It is completely up to you whether or not to join the study.**

Take your time in deciding. If it helps, talk to people you trust, such as your doctor, friends or family. If you decide not to join this study, or if you leave it after you have joined, your other care at this clinic and the benefits or rights you would normally have will not be affected.

If you join this study, you may not be allowed to join some other HIV prevention studies now or in the future. You cannot be in this study while you are in another study where you get a study product. Being in more than one study may not be safe. *Remainder of paragraph for South African sites.* We check to make sure that you are not in more than one study by taking your fingerprint on an electronic system. This information is only accessed by a few members of the study team using a secure password.

You should not donate blood or tissue during the study.

If you choose not to join this study, you may be able to join another study.

Site: Remove item 5 if you use a separate screening consent that covers these procedures.

5. **If you want to join the study, we will screen you to see if you are eligible.**

Screening involves a physical exam, HIV test and health history. A physical exam may include, but is not limited to:

- Checking your weight, temperature and blood pressure

FOR REVIEW ONLY

- Looking in your mouth and throat
- Checking your veins to see how easy it might be to start an infusion
- Looking at your skin
- Listening to your heart and lungs
- Feeling your abdomen (stomach and liver)
- Asking you about any symptoms of COVID-19 that you may have
- Asking you about vaccines you have gotten recently

We will also do blood and urine tests. These tests tell us about some aspects of your health, such as how healthy your kidneys, liver, and immune system are. We will ask you about medications you are taking. We will ask you about behaviors that might put you at risk for getting HIV.

If you were assigned female sex at birth, we will test you for pregnancy. If you have had your uterus or ovaries removed (a hysterectomy or oophorectomy), and this is verified by medical records, you are not required to have a pregnancy test.

We will review the screening results with you. The screening results may show you are not eligible to join the study, even if you want to.

Site: adapt the following section so it is applicable to the care available at your site

6. **If we find that you have a health problem during screening or during the study, we will tell you about the care that we can give here for free.**

   For the care that we cannot give, we will explain how we will help you get care elsewhere. For health problems that are unrelated to the study, we will not pay for care.

7. **If you were assigned female sex at birth and could become pregnant, you must agree to use birth control to join this study.**

Site: If you want to include Appendix C, Approved birth control methods (for sample informed consent form), in this consent form, paste it below and delete paragraph below.

You should not become pregnant during the study because we do not know how the study antibody could affect the developing baby. You must agree to use effective birth control from 21 days before your infusion until your last required clinic visit. We will talk to you about effective birth control methods. They are listed on a handout that we will give to you.

FOR REVIEW ONLY

Being in the study

If you meet the study requirements and want to join, here is what will happen:

8. **You will come to the clinic for scheduled visits about [#] times over [Insert period of time].**

Site: Insert number of visits and range of visit lengths. (There is site-specific variation in screening protocols and in the number of possible follow-up visits between protocol-mandated visits.)

Most of the visits will be 1-2 months apart. After you get the study antibody, we will ask you to come to the clinic for follow up visits 3 days later and 6 days later to draw your blood. We will do this so that we can look at how your body responds to the study product. Visits can last from [#] to [#] hours.

Site: Please adjust the first sentence in the paragraph below to reflect whether you assess COVID-19 symptoms by phone/email/text message prior to the scheduled visit, or if symptom assessment occurs at the visit itself. You can also add a description of how symptoms are monitored upon arrival at your location, such as temperature screening in the building lobby, etc., so that participants know what to expect.

You may have to come for more visits if you have a lab or health issue.

We may contact you after the study ends (for example, to tell you about the study results).

9. **We will give you [Site: Insert compensation] for each study visit you complete.**

This amount is to cover the costs of [Site: Insert text]

Site: Insert any costs to participants (eg, birth control costs for participants who could become pregnant).

US sites: Include the following paragraph. You can remove the box around the text.

Payments you receive for being in the study may be taxable. We may need to ask you for your Social Security number for tax reasons.

You do not have to pay anything to be in this study.

FOR REVIEW ONLY

10. **We will give you the study antibody by IV infusion or SC infusion at your enrollment visit.**

For the IV infusion, we will use a sterile needle to place a small plastic tube into a vein in your arm or hand. The small plastic tube is connected to a small bag of fluid that contains the study antibody. A pump controls how fast the fluid drips from the bag, through the tube, and into your vein. The IV infusion will take about 1 hour.

When getting a subcutaneous (SC) infusion, sterile needles are put under the skin on your abdomen, arm, or thigh. The dose of the antibody will be divided and given in 2 or 3 locations at the same time. A pump is used to control how fast the study antibody is injected through the needles. The infusion will take between 15-90 minutes depending on which group you are in and depending on your weight.

If you have any symptoms while you are getting the study antibody, tell the study staff. Slowing or stopping the flow rate may help improve the symptoms.

11. **We will give you the study antibody at one visit.**

You will be in one of 5 groups. You will get 1 infusion during the study as shown in the table below.

People in Group 1 will be enrolled first, and the safety will be reviewed before enrollment starts in Group 2 and 3. Safety for Groups 2 and 3 will be reviewed before enrollment begins in Groups 4 and 5. Enrollment in Groups 2 and 3, and Groups 4 and 5 is completely random, like flipping a coin. We have no say in how people are assigned to these groups. Neither do you.

				Infusion schedule:
Group	Number of participants	Dose	How it is given	Enrollment Visit
1	3	lower	IV	PGDM1400LS
2	3	medium	IV	PGDM1400LS
3	3	medium	SC	PGDM1400LS
4	3	higher	IV	PGDM1400LS
5	3	higher	SC	PGDM1400LS

You will have to wait in the clinic for about 1 hour after the infusion to see if there are any problems. We will collect a blood sample about 1 hour after the infusion. Then for that night and for 3 more days, you will need to keep track in a diary of how you are feeling and if you have any symptoms. *Site: Customize the next sentence based on how you collect reactogenicity information.* We will review the diary with you during the visits after you get an infusion. You will turn

FOR REVIEW ONLY

in the diary by the Day 6 visit. Contact the clinic staff if you have any issues or concerns after getting an infusion. If you have a problem, we will continue to check on you until it goes away.

12. **In addition to giving you the study antibody we will:**

- Do regular HIV testing, as well as counseling on your results and on how to avoid getting HIV
- Do physical exams
- Do pregnancy tests if you were assigned female sex at birth
- Ask questions about your health, including medications you may be taking, including PrEP
- Ask questions about any personal problems or benefits you may have from being in the study
- Ask questions about your experience getting infusions
- Take urine and blood samples.

When we take blood, the amount will depend on the lab tests we need to do. It will be some amount between 25 mL and 105 mL (a little less than 2 tablespoons to about 1/2 cup). Your body will make new blood to replace the blood we take out.

Site: You may want to add a sentence to the end of the previous paragraph contextualizing the blood volumes described (eg, "To compare, people who donate blood in the US can give a total of about 500 mL in an 8-week period."). Modify the example for cultural relevance and alter blood volumes as necessary.

Site: Insert Appendix E, Table of procedures (for informed consent form) in this section or distribute it as a separate sheet if it is helpful to your study participants. You are not required to do either.

We will be looking for side effects. We will review the results of these procedures and tests with you at your next visit, or sooner if necessary. If any of the results are important to your health, we will tell you.

Some procedures may be done remotely, such as by phone, text message, email or video calls. This could happen instead of or in combination with in-person visits at the study clinic. Some study procedures might also take place in other locations, such as outdoor tents or mobile clinics. The staff will tell you more about the options they plan to use to protect everyone's safety.

FOR REVIEW ONLY

13. **Getting a COVID-19 vaccine while you are in this study is allowed.**

    To be sure that we can answer the study questions, we will not give the study infusion within 7 days before or after you get a COVID-19 vaccine. If you are planning to get a COVID-19 vaccine, please tell us so that we can schedule your study visits with this in mind.

14. **We will counsel you about protecting yourself from HIV.**

    We will ask you personal questions about your HIV risk factors such as sexual behavior, alcohol, and drug use. We will talk with you about ways to keep your risk of acquiring HIV low.

15. **We will test your samples.**

    We will send your samples (without your name) to labs approved by the HVTN and HPTN for this study, which are located in the United States and South Africa. In rare cases, some of your samples may be sent to labs approved by the HVTN and HPTN in other countries for research related to this study.

    Researchers may also do genetic testing related to this study on your samples. Your genes are passed to you from your birth parents. They affect how you look and how your body works. The differences in people's genes can help explain why some people get a disease while others do not. The genetic testing will only involve some of your genes, not all of your genes (your genome). The researchers will study only the genes related to the immune system and HIV and those that affect how people get HIV.

    These tests done on your samples are for research purposes, not to check your health. The labs will not give the results to you or this clinic because their tests are not approved for use in making health care decisions. These labs are only approved to do research tests.

    When your samples are no longer needed for this study, the HVTN and HPTN will continue to store them.

Site: Delete next section if using separate consent for use of samples and information in other studies

16. **When samples are no longer needed for this study, the HVTN and HPTN want to use them in other studies and share them with other researchers.**

    The HVTN and HPTN call these samples "extra samples". The HVTN and HPTN will only allow your extra samples to be used in other studies if you agree to this. You will mark your decision at the end of this form. If you have any questions, please ask.

FOR REVIEW ONLY

Do I have to agree? No. You are free to say yes or no, or to change your mind after you sign this form. At your request, we will destroy all extra samples that we have. Your decision will not affect your being in this study or have any negative consequences here.

Where are the samples stored? Extra samples are stored in a secure central place called a repository. *[Site: choose one of the following two sentences. African sites should choose the sentence referencing the repository in South Africa. All other sites should choose the sentence referencing the repository in the United States.]* Your samples will be stored in the HVTN repository in South Africa. Your samples will be stored in the HVTN repository in the United States.

How long will the samples be stored? There is no limit on how long your extra samples will be stored. *[Site: Revise the previous sentence to insert limits if your regulatory authority imposes them.]*

Will I be paid for the use of my samples? No. Also, a researcher may make a new scientific discovery or product based on the use of your samples. If this happens, there is no plan to share any money with you. The researcher is not likely to ever know who you are.

Will I benefit from allowing my samples to be used in other studies? Probably not. Results from these other studies are not given to you, this clinic, or your doctor. They are not part of your medical record. The studies are only being done for research purposes.

Will the HVTN or HPTN sell my samples and information? No, but the HVTN and HPTN may share your samples with HVTN, HPTN, or other researchers. Once we share your samples and information, we may not be able to get them back.

How do other researchers get my samples and information? When a researcher wants to use your samples and information, their research plan must be approved by the HVTN and HPTN. Also, the researcher's institutional review board (IRB) or ethics committee (EC) will review their plan. *[Site: If review by your institution's IRB/EC/RE is also required, insert a sentence stating this.]* IRBs/ECs protect the rights and well-being of people in research. If the research plan is approved, the HVTN will send your samples to the researcher's location.

What information is shared with HVTN, HPTN, or other researchers? The samples and information will be labeled with a code number. The key to the code will stay at this clinic. It will not be shared with the HVTN, HPTN, other researchers, or with anyone else who does not need to know your name. Your name will not be part of the information. However, some information that we share may be personal, such as your race, ethnicity, sex, health information from the study, and HIV status. We may share information about the study antibody you received and how your body responded to the studyantibody.

FOR REVIEW ONLY

What kind of studies might be done with my extra samples and information? The studies will be related to HIV, vaccines, antibodies, the immune system, and other diseases.

Researchers may also do genetic testing on your samples. If you agree, your samples could also be used for genome wide studies. In these studies, researchers will look at all of your genes (your genome). The researchers compare the genomes of many people, looking for common patterns of genes that could help them understand diseases. The researchers may put the information from the genome-wide studies into a protected database so that other researchers can access it, but your name and other personal information will not be included. Usually, no one would be able to look at your genome and link it to you as a person. However, if another database exists that also has information on your genome and your name, someone might be able to compare the databases and identify you. If others found out, it could lead to discrimination or other problems. The risk of this is very small. There may be other unknown risks.

Who will have access to my information in studies using my extra samples?

People who may see your information are:

- Researchers who use your extra samples and information for other research
- Government agencies that fund or monitor the research using your extra samples and information
- Any regulatory agency that reviews clinical trials
- The researcher's Institutional Review Board or Ethics Committee
- The people who work with the researcher

All of these people will do their best to protect your information. The results of any new studies that use your extra samples and information may be published. No publication will use your name or identify you personally.

17. **We will do our best to protect your private information.**

US sites: Check HIPAA authorization for conflicts with this section.

Your study records and samples will be kept in a secure location. We will label all of your samples and most of your records with a code number, not your name or other personal information. However, it is possible to identify you, if necessary. We will not share your name with the lab that does the tests on your samples, or with anyone else who does not need to know your name.

FOR REVIEW ONLY

Clinic staff will have access to your study records. Your records may also be reviewed by groups who watch over this study to see that we are protecting your rights, keeping you safe, and following the study plan. These groups include:

- The US National Institutes of Health, people who work for them, and their study monitors,
- The US Food and Drug Administration,
- Any regulatory agency that reviews clinical trials,
- *Non-US Sites must include; US sites may but are not required to include*:[Insert name of local IRB/EC] ,
- [Insert name of local and/or national regulatory authority as appropriate],
- The HVTN, HPTN and people who work for them,
- The Safety Monitoring Board and
- The US Office for Human Research Protections.

All reviewers will take steps to keep your records private.

We cannot guarantee absolute privacy. If you are found to have a medical condition that we are required to report by law, then some of your information may be shared. At this clinic, we have to report the following information:

Site: Include any public health or legal reporting requirements, including SARS-CoV-2/COVID-19. Bulleted examples should include all appropriate cases (reportable communicable disease, risk of harm to self or others, etc.). If your site does not have public health or legal reporting requirements, you may delete the last sentence in the paragraph above, along with the bullets below.

- [Item 1]
- [Item 2]
- [Item 3]

US sites: Include the following boxed text. You can remove the box around the text.

We have a Certificate of Confidentiality from the US government, to help protect your privacy. With the certificate, we do not have to release information about you to someone who is not connected to the study, such as the courts or police. Sometimes we can't use the certificate. Since the US government funds this research, we cannot withhold information from it. Also, you can still release information about yourself and your study participation to others.

FOR REVIEW ONLY

The results of this study may be published. No publication will use your name or identify you personally.

We may share information from the study with other researchers. We will not share your name or information that can identify you.

A description of this clinical trial will be available on http://www.ClinicalTrials.gov. This website will not include information that can identify you. At most, the website will include a summary of the results. You can search this website at any time.

18. **If you become pregnant or acquire HIV, we may ask you to stay in the study to complete other study procedures.**

    If you become pregnant, we will encourage you to stay in the study if you choose. We will discuss your study options with you. If you leave the study while you are still pregnant, we will contact you after your due date to ask some questions about your pregnancy and delivery.

    If you get HIV while in the study, we will also take fewer samples, and we will help you get care and support. We will encourage you to stay in the study for up to 6 months after your last infusion or last scheduled protocol clinic visit whichever comes first. We will discuss your study options with you. We will counsel you about having HIV and about telling your partner(s). *Site: Modify the following sentence as appropriate.* We will not provide or pay for any of your HIV care directly.

19. **We may take you out of the study at any time**.

    We may take you out of the study if:

    - you do not follow instructions,
    - we think that staying in the study might harm you,
    - the study is stopped for any reason.

Other Risks

20. **There are other risks to being in this study.**

    This section describes the other risks and restrictions we know about. There may also be unknown risks, even serious ones. We will tell you if we learn anything new that may affect your willingness to stay in the study.

    Risks of giving blood:

FOR REVIEW ONLY

Giving blood can cause bruising, pain, fainting, soreness, redness, swelling, itching, bleeding, and infection (rarely). Giving blood can cause a low blood cell count (anemia), making your feel tired.

Risks of SC infusion procedures:

Getting a SC infusion may cause bruising, pain, fainting, soreness, redness, swelling, itching, a sore, bleeding, blood clots, forming a bump under the skin, and (rarely) muscle damage, or infection where you got the SC infusion.

Risks of IV infusion procedures:

Getting an IV may cause stinging, discomfort, pain, soreness, redness, bruising, itching, rash and swelling where the needle goes into the skin. Rarely, needle sticks can result in a blood clot or infection.

Personal problems/discrimination:

Some people who join HVTN and HPTN studies report personal problems or discrimination because of joining an HIV prevention study. Family or friends may worry, get upset or angry, or assume that you have HIV or are at high risk and treat you unfairly as a result. Rarely, a person has lost a job because the study took too much time away from work, or because their employer thought they had HIV.

HIV testing:

HIV antibody tests are the usual way to test for HIV infections. We are still learning how HIV tests perform when people are given monoclonal antibodies. We have used several common HIV antibody tests to test samples of blood containing antibodies similar to the study antibody. We found that very high levels of these similar antibodies can cause positive or uncertain results on some HIV tests. You may have such high levels for a short time after you get the study antibody. This means that for a few days after getting the study antibody, some HIV tests might say you have HIV, even if you don't.

For this reason, you should plan to get HIV tests only at this clinic during the study. Our tests can truly detect HIV. They can also tell if someone does not have HIV. We do not expect you to have any problems with HIV testing after the study ends.

Embarrassment/anxiety:

You may feel embarrassed when we ask about your HIV risks, such as having sex and using drugs. Also, waiting for your HIV test results or other health test results could make you feel anxious. You could feel worried if your test results show that you have HIV. If you feel embarrassed or anxious, please tell us and we will try to help you.

FOR PREVIEW ONLY

Risks of disclosure of your personal information:

We will take several steps to protect your personal information. Although the risk is very low, it is possible that your personal information could be given to someone who should not have it. If that happened, you could face discrimination, stress, and embarrassment. We can tell you more about how we will protect your personal information if you would like it.

Risks of genetic testing:

It is unlikely, but the genetic tests done on your samples could show you may be at risk for certain diseases. If others found out, it could lead to discrimination or other problems. However, it is almost impossible for you or others to know your test results from the genetic testing. The results are not part of your study records and are not given to you.

U.S. Sites, include the following paragraph In the very unlikely event that your genetic information becomes linked to your name, a federal law called the Genetic Information Nondiscrimination Act (GINA) helps protect you. GINA keeps health insurance companies and employers from seeing results of genetic testing when deciding about giving you health insurance or offering you work. GINA does not help or protect you against discrimination by companies that sell life, disability or long-term care insurance.

Unknown risks:

We do not know if the study antibody will increase, decrease, or not change your risk of getting HIV if you are exposed. If you get HIV, we do not know how the study antibody might affect your HIV infection or how long it takes to develop AIDS.

We do not know if getting this study antibody will affect how you respond to any future approved HIV antibody or vaccine. Currently, no HIV antibody or vaccine has been approved for use.

We do not know how the study antibody will affect a pregnant participant or a developing baby.

Benefits

21. **The study may not benefit you.**

We do not expect the study antibody to benefit you in any way. However, being in the study might still help you in some ways. The counseling that you get as part of the study may help you avoid getting HIV. The lab tests and physical exams that you get while in this study might detect health problems you don't yet know about.

FOR PREVIEW ONLY

When asked, most study participants say that participating in a study made them feel good about helping others, increased their knowledge about HIV, and improved their self-esteem.

This study may help in the search for an antibody or vaccine to prevent HIV. However, if the study antibody or a vaccine later become approved and sold, there are no plans to share any money with you.

Your rights and responsibilities

22. **If you join the study, you have rights and responsibilities.**

You have many rights that we will respect. You also have responsibilities. We list these in the Bill of Rights and Responsibilities for HIV Research. We will give you a copy of it.

Leaving the study

23. **Tell us if you decide to leave the study.**

You are free to leave the study at any time and for any reason. Your care at this clinic and your legal rights will not be affected, but it is important for you to let us know.

Previously collected information about you will remain in the study records and will be included in the analysis of results. Your information can not be removed from the study records.

We may ask you to come back to the clinic one last time for a physical exam, and we may ask to take some blood and urine samples. We may also ask about any personal problems or benefits you have experienced from being in the study. We believe these steps are important to protecting your health, but it is up to you whether to complete them.

Injuries

Sites: Approval from HVTN Regulatory Affairs (at vtn.core.reg@hvtn.org) is needed for any change (other than those that the instructions specifically request or those previously approved by HVTN Regulatory Affairs) to the boxed text. You can remove the box around the text.

24. **If you get sick or injured during the study, contact us immediately.**

2 paragraphs below for all sites.

Your health is important to us. *(Sites: adjust the following 2 sentences if applicable to the care available at your site)* We will tell you about the care that

FOR REVIEW ONLY

we can give here. For the care that we cannot provide, we will explain how we will help you get care elsewhere.

If you become sick or injured in this study, the HVTN has a process to decide if it is related to the study product and/or procedures. If it is, we call it a study-related injury. There are funds to pay for treatment of study-related injuries if certain conditions are met.

Next paragraph for non-US sites.

Sites: adjust the language in this paragraph so it is applicable to your site. Note that the ABPI guidelines apply to South Africa only. In this study, our clinic has insurance to cover your medical treatment in the case of a study-related injury. We will follow the Association of the British Pharmaceutical Industry guidelines for payment of study-related injury. We can give you a copy of these guidelines. In rare cases, the insurance funds may not be enough.

In this situation, the HVTN has limited funds to pay medical costs that it determines are reasonable. *(Sites: insert locale- appropriate medical insurance language in the following sentence)* If the injury is not study related, then you and/or your health insurance will be responsible for treatment costs.

Next paragraph for US sites.

The HVTN has limited funds to pay medical costs that it determines are reasonable. *(Sites: insert locale- appropriate medical insurance language in the following sentence)* If the injury is not study related, then you and/or your health insurance will be responsible for treatment costs.

2 paragraphs below for all sites.

Some injuries are not physical. For example, you might be harmed emotionally by being in an HIV prevention study. Or you might lose wages because you cannot go to work. However, there are no funds to pay for these kinds of injuries, even if they are study related.

You may disagree with the decision about whether your injury is study related. If you wish, the HVTN will ask independent experts to review the decision. You always have the right to use the court system if you are not satisfied.

FOR REVIEW ONLY

Questions

25. **If you have questions or problems at any time during your participation in this study, use the following important contacts.**

If you have questions about this study, contact
[name or title and telephone number of the investigator or other study staff].

If you have any symptoms that you think may be related to this study, contact
[name or title and telephone number of the investigator or other study staff].

This study has been reviewed and approved by a committee called the
[name of local IRB/EC]. If you have questions about your rights as a research participant, or problems or concerns about how you are being treated in this study, contact [name or title and telephone number of person on IRB/EC] , at the committee.

Paragraph for South African sites. The study has been structured in accordance with the Declaration of Helsinki (last updated October 2013) which deals with the recommendations guiding doctors in biomedical research involving human participants, the Ethics in Health Research: Principles, Structures and Processes Second Edition 2015, and Guidelines for Good Practice in the Conduct of Clinical Trials in Human Participants in South Africa. We can provide you with copies of these guidelines if you wish to review them. In addition, the recent Protection of Personal Information Act (POPIA) ensures that all South African institutions conduct themselves in a responsible manner when collecting, processing, storing and sharing another entity’s personal information by holding them accountable should they abuse or compromise your personal information in any way.If you want to leave this study, contact
[name or title and telephone number of the investigator or other study staff].

Remainder of section for South African sites only.

You can reach a study staff member 24 hours a day at [telephone number].

If you have questions about this trial you should first discuss them with your doctor or the ethics committee (contact details as provided on this form). After you have consulted your doctor or the ethics committee and if they have not provided you with answers to your satisfaction, you should write to the South African Health Products Regulatory Authority (SAHPRA) at:

The Chief Executive Officer
Dr Boitumelo Semete-Makokotlela
South African Health Products Regulatory Authority Private Bag X828
PRETORIA
0001
Tel: (012) 501 0410
e-mail: Boitumelo.Semete@sahpra.org.za

FOR PREVIEW ONLY

Your permissions and signature

Site: Delete this section if using a separate consent for use of samples and information in other studies

26. **In Section 16 of this form, we told you about possible other uses of your extra samples and information, outside this study. Please choose only one of the options below and write your initials or make your mark in the box next to it. Whatever you choose, the HVTN and HPTN keep track of your decision about how your samples and information can be used. You can change your mind after signing this form.**

☐ I allow my extra samples and information to be used for other studies related to HIV, vaccines, antibodies, the immune system, and other diseases. This may include genetic testing.

OR

☐ I agree to the option above *and* also to allow my extra samples and information to be used in genome wide studies.

OR

☐ I do not allow my extra samples to be used in any other studies. This includes not allowing genetic testing or genome wide studies.

27. **If you agree to join this study, you will need to sign or make your mark below. Before you sign or make your mark on this consent form, make sure of the following:**

- You have read this consent form, or someone has read it to you.
- You feel that you understand what the study is about and what will happen to you if you join. You understand what the possible risks and benefits are.
- You have had your questions answered and know that you can ask more.
- You agree to join this study.

You will not be giving up any of your rights by signing this consent form.

FOR REVIEW ONLY

Participant's name (print)	Participant's signature or mark	Date	Time
Clinic staff conducting consent discussion (print)	Clinic staff signature	Date	Time

For participants who are unable to read or write, a witness should complete the signature block below:

Witness's name (print)	Witness's signature	Date	Time

*Witness is impartial and was present for the entire discussion of this consent form.

FOR REVIEW ONLY

Appendix B Sample informed consent form for Part B

Title: A phase 1 dose-escalation clinical trial to evaluate the safety, tolerability, and pharmacokinetics of PGDM1400LS alone and in combination with VRC07-523LS and PGT121.414.LS in healthy, HIV-uninfected adult participants
Protocol number: HVTN 140/HPTN 101
Site: [Insert site name]

Thank you for your interest in our research study. Please read this consent form or ask someone to read it to you. If you decide to join the study, we will ask you to sign or make your mark on this form. We will offer you a copy to keep. We will ask you questions to see if we have explained everything clearly. You can also ask us questions about the study.

Research is not the same as treatment or medical care. The purpose of a research study is to answer scientific questions.

Key Information

These are some of the things you should know about this study:

- The purpose of this part of the study is to understand how the body's immune system responds to lab-made antibodies against HIV when they are given in combination at different doses. We also want to see if the way the antibodies is given affects the immune response.
- We also want to see if the antibodies are safe to give to people and do not make them too uncomfortable.
- If you choose to join, we will ask you to come to several study visits over about 10 months. At 2 of these visits you will get the study antibodies in a vein (by IV infusion) or under the skin (by subcutaneous infusion, called SC). We will take blood from you at each study visit to see how your body responds to the study antibodies and how much of the antibody combination remains in your blood. We will test you for HIV and other sexually transmitted infections (STIs) and pregnancy (if applicable). We will ask you to complete questionnaires and you will have physical exams.
- Some general risks of antibodies include fever, chills, shaking, nausea, vomiting, pain, diarrhea, headache, dizziness, fatigue, flushing, trouble breathing, high or low blood pressure, itchiness and rash. There may be other side effects that we don't yet know about, even serious ones. Because this is the first time that one of the study antibodies is being given to people, and the first time this combination of antibodies is being given to people, we do not know what all of the risks may be. We think that the risks will be similar to these general risks.
- There is no direct benefit to you from being in the study.

FOR REVIEW ONLY

- Whether to take part in this study is your choice. You do not have to take part in the study and you are free to stop at any time.
- The rest of this form provides a more complete description of this study. Please read it carefully.

About the study

The HIV Vaccine Trials Network (HVTN), the HIV Prevention Trials Network (HPTN), and [Insert site name] are doing a study to test a combination of antibodies against HIV. HIV is the virus that causes AIDS. Antibodies are made by the body as one way to respond to or fight infection. Researchers can also make antibodies in laboratories and give them to people by infusion into a vein or under the skin. We will tell you more about these procedures below. Antibodies have been used successfully to prevent or treat other health problems, such as COVID-19 caused by the SARS-CoV-2 virus, and respiratory infections in babies caused by RSV (respiratory syncytial virus).

About 95 people will take part in this study at multiple sites. The researcher in charge of this study at this clinic is [Insert name of site PI]. The US National Institutes of Health (NIH) is paying for the study.

There are 2 parts of this study, Part A and Part B. In Part A, we gave different doses of one of the study antibodies to 15 people. People in Part A got the study antibody one time. Part A was the first time that antibody has been tested in people. The results of Part A show that it is safe to move ahead with Part B of the study. In Part B, we will give about 80 more people a combination of three study antibodies, including the antibody tested in Part A.

You are being invited to join part B of the study.

1. **We are doing this part of the study to answer several questions.**

   - Are the study antibodies safe to give to people together at different doses?
   - Are people able to take the study antibodies without becoming too uncomfortable?
   - How do people’s immune systems respond to the study antibodies? (Your immune system protects you from disease.)
   - Does the method of giving the study antibodies change the body’s response?
   - How much of the antibodies remain in the body as time passes?

FOR REVIEW ONLY

2. **The study antibodies cannot give you HIV.**

   The study antibodies are not made from actual HIV. It is impossible for the study antibodies to give you HIV. Also, they cannot cause you to give HIV to someone else. We do not know if the study antibodies will decrease, increase, or not change your risk of getting HIV if you are exposed to the virus.

3. **These study antibodies are experimental.**

   The study antibodies are called PGDM1400LS, VRC07-523LS, and PGT121.414.LS. From here on, we will call them the study antibodies. They are experimental HIV prevention products. That means we do not know if they will be safe to use in people, or if they will work to prevent HIV infection. These antibodies are used only in research studies.

   The 3 study antibodies were developed by researchers with the National Institute of Allergy and Infectious Diseases (NIAID) and the Collaboration for AIDS Vaccine Discovery. Researchers at Beth Israel Deaconess Medical Center in Boston, MA were also involved in developing PGT121.414.LS.

   The study antibody PGDM1400LS has only been given to the 15 people who were enrolled in Part A. A review of the safety information from these participants shows that it is safe to continue with Part B of the study. An earlier version of the study antibody called PGDM1400 has been given to 53 people in 3 other studies. One of these studies is still ongoing, but so far there have been no safety concerns. Nine people have been given PGDM1400 alone and 44 have in combination with other antibodies. PGDM1400LS is a new version of PGDM1400 and has been changed so that it will last longer in the body.

   The other 2 antibodies have been given to 231 people in 6 studies. VRC07-523LS has been given alone to 173 people and in combination with other antibodies to 49 people. PGT121.414.LS has only been given to 9 people. An earlier version of the study antibody called PGT121 has been given alone to 41 people and in combination with other antibodies to 47 people, in 4 studies. PGT121.414.LS is a new version of PGT121 and has been changed so that it will last longer in the body.

   Antibodies given to a person usually do not last in the body more than a few months. One of the goals of this study is to see how long these study antibodies will stay in the body. We don't know yet how long they will last, but we think it may be several months.

   Risks of the study antibodies:

   This section lists the side effects we know about. There may be others that we don't yet know about, even serious ones. We will tell you if we learn about any new side effects. Based on what we have seen so far in ongoing studies, we think that the risks will be similar to the general risks described below.

FOR PREVIEW ONLY

General risks of antibodies:

Other kinds of antibodies used for other health problems have caused fever, chills, itchiness, rash, hives, redness in cheeks and neck, lip or face swelling, nausea, vomiting, diarrhea, pain, headache, fatigue, flushing, dizziness, shaking, trouble breathing, high or low blood pressure, racing heartbeat, and chest pain. These reactions may be related to how an antibody is made, what it targets, or how fast the antibody product is given. At times the side effects include reactions at injection site (pain, redness, bruising, swelling, hardening). Most side effects happen within the first 24 hours.

Rarely, some antibodies for other diseases have caused 2 kinds of side effects that may be life-threatening:

- Anaphylaxis – a physical reaction that may include hives or rash, mouth and face swelling, low blood pressure, and trouble breathing, possibly leading to low blood oxygen. This may occur soon after getting an antibody.

- Serum Sickness – a physical reaction that includes hives or rash, fever, big lymph nodes, muscle and joint pain, chest discomfort and shortness of breath. This may occur several days to a few weeks after getting an antibody.

These rare side effects have not been seen in other studies with similar experimental antibodies against HIV. Please tell us if you have ever had a reaction similar to anaphylaxis or serum sickness.

Rarely, antibodies approved for treatment of other diseases have been linked to other effects like cancer, increased bleeding, increased infections, heart and liver toxicity, and problems with blood clotting and immune response. These rare side effects and reactions have not been seen in other studies with these study antibodies or similar experimental antibodies against HIV.

Joining the study

4. **It is completely up to you whether or not to join the study.**

Take your time in deciding. If it helps, talk to people you trust, such as your doctor, friends or family. If you decide not to join this study, or if you leave it after you have joined, your other care at this clinic and the benefits or rights you would normally have will not be affected.

If you join this study, you may not be allowed to join some other HIV prevention studies now or in the future. You cannot be in this study while you are in another study where you get a study product. Being in more than one study may not be safe. *Site: Remainder of paragraph for South African sites.* We check to make sure that you are not in more than one study by taking your fingerprint on an electronic system. This information is only accessed by a few members of the study team using a secure password.

FOR REVIEW ONLY

You should not donate blood or tissue during the study.

If you choose not to join this study, you may be able to join another study.

Site: Remove item 5 if you use a separate screening consent that covers these procedures.

5. **If you want to join the study, we will screen you to see if you are eligible.**

   Screening involves a physical exam, HIV test and health history. A physical exam may include, but is not limited to:

   - Checking your weight, temperature and blood pressure
   - Looking in your mouth and throat
   - Checking your veins to see how easy it might be to start an infusion
   - Looking at your skin
   - Listening to your heart and lungs
   - Feeling your abdomen (stomach and liver)
   - Asking you about any symptoms of COVID-19 that you may have
   - Asking you about vaccines you have gotten recently.

   We will also do blood and urine tests. These tests tell us about some aspects of your health, such as how healthy your kidneys, liver, and immune system are. We will ask you about medications you are taking. We will ask you about behaviors that might put you at risk for getting HIV.

   If you were assigned female sex at birth, we will test you for pregnancy. If you have had your uterus or ovaries removed (a hysterectomy or oophorectomy), and this is verified by medical records, you are not required to have a pregnancy test.

   We will review the screening results with you. The screening results may show you are not eligible to join the study, even if you want to.

Site: adapt the following section so it is applicable to the care available at your site

6. **If we find that you have a health problem during screening or during the study, we will tell you about the care that we can give here for free.**

   For the care that we cannot give, we will explain how we will help you get care elsewhere. For health problems that are unrelated to the study, we will not pay for care.

FOR REVIEW ONLY

7. **If you were assigned female sex at birth and could become pregnant, you must agree to use birth control to join this study.**

Site: If you want to include Appendix C, Approved birth control methods (for sample informed consent form), in this consent form, paste it below and delete paragraph below.

You should not become pregnant during the study because we do not know how the study antibodies could affect the developing baby. You must agree to use effective birth control from 21 days before your first infusion until your last required clinic visit. We will talk to you about effective birth control methods. They are listed on a handout that we will give to you.

Being in the study

If you meet the study requirements and want to join, here is what will happen:

8. **You will come to the clinic for scheduled visits about [#] times over [Insert period of time].**

Site: Insert number of visits and range of visit lengths. (There is site-specific variation in screening protocols and in the number of possible follow-up visits between protocol-mandated visits.)

Most of the visits will be 1-2 months apart. After you get the study antibodies, we will also ask you to come to the clinic for follow-up visits about 3 and 6 days after the first infusion to take your blood. We will do this so that we can look at how your body responds to the study products. Visits can last from [#] to [#] hours.

Site:Please adjust the first sentence in the paragraph below to reflect whether you assess COVID-19 symptoms by phone/email/text message prior to the scheduled visit, or if symptom assessment occurs at the visit itself. You can also add a description of how symptoms are monitored upon arrival at your location, such as temperature screening in the building lobby, etc., so that participants know what to expect.

You may have to come for more visits if you have a lab or health issue.

We may contact you after the study ends (for example, to tell you about the study results).

9. **We will give you [Site: Insert compensation] for each study visit you complete.**

This amount is to cover the costs of [Site: Insert text]

Site: Insert any costs to participants (eg, birth control costs for participants who could become pregnant).

FOR REVIEW ONLY

US sites: Include the following paragraph. You can remove the box around the text.

Payments you receive for being in the study may be taxable. We may need to ask you for your Social Security number for tax reasons.

You do not have to pay anything to be in this study.

10. **We will give you the study antibodies by IV infusion or SC infusion.**

For the IV infusion, we will use a sterile needle to place a small plastic tube into a vein in your arm or hand. The small plastic tube is connected to a small bag of fluid that contains the study antibody. A pump controls how fast the fluid drips from the bag, through the tube, and into your vein. Each antibody will be given in a separate infusion, one after the other. If you have any symptoms while you are getting the study antibodies, tell the study staff. Slowing or stopping the flow rate may help improve the symptom.

At the first visit, the IV infusions will take about one hour for each study antibody (total of about 3 hours). The second IV infusion visit will take about 15 - 30 minutes for each study antibody (total of about 1 1/2 hours).

When getting a subcutaneous (SC) infusion, sterile needles are put under the skin on your abdomen, arm, or thigh. The dose of each antibody will be divided and given in 2 or 3 locations at the same time. A pump is used to control how fast the study antibody is injected through the needles. Each antibody will be given separately, one after the other, and you'll be able to get up and move around in between infusions. The infusion of the three study antibodies will take between 45 minutes – 2 hours depending on which group you are in and depending on your weight.

The highest dose of antibodies given to some people in this study is more than the highest dose of these antibodies given in previous studies. However, it is less than the amount of other licensed antibodies given to treat other illnesses.

11. **We will give you the study antibodies on a schedule.**

You will be in either group 6, 7, 8, 9 or 10. (Groups 1-5 were in Part A of the study.) You will get 2 infusions during the study as shown in the table below. The difference between the groups is the dose of study antibodies being given and the way they are being given. Some people will get a fixed dose of antibodies and some people will get a dose of antibodies based on their body weight.

Because this is the first time the antibodies are being given to people in this combination, the study has planned a pause when safety information will be reviewed to decide if it is safe to continue the study. The pause will be after the first 8 participants in Groups 6-9, with two participants per group, have had their first infusion. If the safety review of these participants shows it is safe to continue, then enrollment will resume for the rest of the participants in Groups 6-9 as well

FOR REVIEW ONLY

as Group 10. You have an equal chance of being in Groups 6-9. Whether you are enrolled in Groups 6-9 or Group 10 will depend on when you join the study.

				Infusion schedule	
Group	Number of participants	Dose	How it is given	Enrollment Visit	4 months later
6	16	medium dose weight based	IV	PGDM1400LS + VRC07-523LS + PGT121.414.LS	PGDM1400LS + VRC07-523LS + PGT121.414.LS
7	16	medium dose weight based	SC	PGDM1400LS + VRC07-523LS + PGT121.414.LS	PGDM1400LS + VRC07-523LS + PGT121.414.LS
8	16	medium dose fixed	IV	PGDM1400LS + VRC07-523LS + PGT121.414.LS	PGDM1400LS + VRC07-523LS + PGT121.414.LS
9	16	medium dose fixed	SC	PGDM1400LS + VRC07-523LS + PGT121.414.LS	PGDM1400LS + VRC07-523LS + PGT121.414.LS
10	16	higher dose weight based	IV	PGDM1400LS + VRC07-523LS + PGT121.414.LS	PGDM1400LS + VRC07-523LS + PGT121.414.LS

You will have to wait in the clinic for about 1 hour after your last infusion to see if there are any problems. We will collect a blood sample about 1 hour after the infusion. Then for that night and for 3 more days, you will need to keep track in a diary about how you are feeling and if you have any symptoms. *Site: Customize the next sentence based on how you collect reactogenicity information.* We will review the diary with you during the 2 visits after you get an infusion. You will turn in the diary by the Day 6 visit after the first infusion. We will contact you about 4 days after the second infusion to collect the information from your diary. Contact the clinic staff if you have any issues or concerns after getting an infusion. If you have a problem, we will continue to check on you until it goes away.

12. **In addition to giving you the study antibodies we will:**

- Do regular HIV testing, as well as counseling on your results and on how to avoid getting HIV

FOR REVIEW ONLY

- Do physical exams
- Do pregnancy tests if you were assigned female sex at birth
- Ask questions about your health, including medications you may be taking, including PrEP
- Asking you about any symptoms of COVID-19 that you may have
- Ask questions about any personal problems or benefits you may have from being in the study
- Ask questions about your experience getting infusions/injections
- Take urine and blood samples.

When we take blood, the amount will depend on the lab tests we need to do. It will be some amount between 25 mL and 105 mL (a little less than 2 tablespoons to about 1/2 cup). Your body will make new blood to replace the blood we take out.

Site: You may want to add a sentence to the end of the previous paragraph contextualizing the blood volumes described (eg, "To compare, people who donate blood in the US can give a total of about 500 mL in an 8-week period."). Modify the example for cultural relevance and alter blood volumes as necessary.

Site: Insert Appendix E, Table of procedures (for informed consent form) in this section or distribute it as a separate sheet if it is helpful to your study participants. You are not required to do either.

We will be looking for side effects. We will review the results of these procedures and tests with you at your next visit, or sooner if necessary. If any of the results are important to your health, we will tell you.

Some procedures may be done remotely, such as by phone, text message, email or video calls. This could happen instead of or in combination with in-person visits at the study clinic. Some study procedures might also take place in other locations, such as outdoor tents or mobile clinics. The staff will tell you more about the options they plan to use to protect everyone's safety.

13. **Getting a COVID-19 vaccine while you are in this study is allowed.**

To be sure that we can answer the study questions, we will not give the study infusion within 7 days before or after youget a COVID-19 vaccine. If you are planning to get a COVID-19 vaccine, please tell us so that we can schedule your study visits with this in mind.

FOR REVIEW ONLY

14. **We will counsel you about protecting yourself from HIV.**

    We will ask you personal questions about your HIV risk factors such as sexual behavior, alcohol, and drug use. We will talk with you about ways to keep your risk of acquiring HIV low.

15. **We will test your samples.**

    We will send your samples (without your name) to labs approved by the HVTN and HPTN for this study, which are located in the United States and South Africa. In rare cases, some of your samples may be sent to labs approved by the HVTN and HPTN in other countries for research related to this study.

    Researchers may also do genetic testing related to this study on your samples. Your genes are passed to you from your birth parents. They affect how you look and how your body works. The differences in people's genes can help explain why some people get a disease while others do not. The genetic testing will only involve some of your genes, not all of your genes (your genome). The researchers will study only the genes related to the immune system and HIV and those that affect how people get HIV.

    These tests done on your samples are for research purposes, not to check your health. The labs will not give the results to you or this clinic because their tests are not approved for use in making health care decisions. These labs are only approved to do research tests.

    When your samples are no longer needed for this study, the HVTN and HPTN will continue to store them.

Site: Delete next section if using separate consent for use of samples and information in other studies

16. **When samples are no longer needed for this study, the HVTN and HPTN want to use them in other studies and share them with other researchers.**

    The HVTN and HPTN call these samples "extra samples". The HVTN and HPTN will only allow your extra samples to be used in other studies if you agree to this. You will mark your decision at the end of this form. If you have any questions, please ask.

    Do I have to agree? No. You are free to say yes or no, or to change your mind after you sign this form. At your request, we will destroy all extra samples that we have. Your decision will not affect your being in this study or have any negative consequences here.

    Where are the samples stored? Extra samples are stored in a secure central place called a repository. *[Site: choose one of the following two sentences. African sites should choose the sentence referencing the repository in South Africa. All other*

FOR REVIEW ONLY

sites should choose the sentence referencing the repository in the United States.] Your samples will be stored in the HVTN repository in South Africa. Your samples will be stored in the HVTN repository in the United States.

How long will the samples be stored? There is no limit on how long your extra samples will be stored. *[Site: Revise the previous sentence to insert limits if your regulatory authority imposes them.]*

Will I be paid for the use of my samples? No. Also, a researcher may make a new scientific discovery or product based on the use of your samples. If this happens, there is no plan to share any money with you. The researcher is not likely to ever know who you are.

Will I benefit from allowing my samples to be used in other studies? Probably not. Results from these other studies are not given to you, this clinic, or your doctor. They are not part of your medical record. The studies are only being done for research purposes.

Will the HVTN or HPTN sell my samples and information? No, but the HVTN and HPTN may share your samples with HVTN, HPTN, or other researchers. Once we share your samples and information, we may not be able to get them back.

How do other researchers get my samples and information? When a researcher wants to use your samples and information, their research plan must be approved by the HVTN and HPTN. Also, the researcher's institutional review board (IRB) or ethics committee (EC) will review their plan. *[Site: If review by your institution's IRB/EC/RE is also required, insert a sentence stating this.]* IRBs/ECs protect the rights and well-being of people in research. If the research plan is approved, the HVTN will send your samples to the researcher's location.

What information is shared with HVTN, HPTN, or other researchers? The samples and information will be labeled with a code number. The key to the code will stay at this clinic. It will not be shared with the HVTN, other researchers, or with anyone else who does not need to know your name. Your name will not be part of the information. However, some information that we share may be personal, such as your race, ethnicity, sex, health information from the study, and HIV status. We may share information about the study antibodies you received and how your body responded to the studyantibodies.

What kind of studies might be done with my extra samples and information? The studies will be related to HIV, vaccines, antibodies, the immune system, and other diseases.

Researchers may also do genetic testing on your samples. If you agree, your samples could also be used for genome wide studies. In these studies, researchers will look at all of your genes (your genome). The researchers compare the genomes of many people, looking for common patterns of genes that could help

FOR PREVIEW ONLY

them understand diseases. The researchers may put the information from the genome-wide studies into a protected database so that other researchers can access it, but your name and other personal information will not be included. Usually, no one would be able to look at your genome and link it to you as a person. However, if another database exists that also has information on your genome and your name, someone might be able to compare the databases and identify you. If others found out, it could lead to discrimination or other problems. The risk of this is very small. There may be other unknown risks.

Who will have access to my information in studies using my extra samples?

People who may see your information are:

- Researchers who use your extra samples and information for other research
- Government agencies that fund or monitor the research using your extra samples and information
- Any regulatory agency that reviews clinical trials
- The researcher's Institutional Review Board or Ethics Committee
- The people who work with the researcher

All of these people will do their best to protect your information. The results of any new studies that use your extra samples and information may be published. No publication will use your name or identify you personally.

17. **We will do our best to protect your private information.**

US sites: Check HIPAA authorization for conflicts with this section.

Your study records and samples will be kept in a secure location. We will label all of your samples and most of your records with a code number, not your name or other personal information. However, it is possible to identify you, if necessary. We will not share your name with the lab that does the tests on your samples, or with anyone else who does not need to know your name.

Clinic staff will have access to your study records. Your records may also be reviewed by groups who watch over this study to see that we are protecting your rights, keeping you safe, and following the study plan. These groups include:

- The US National Institutes of Health, people who work for them, and their study monitors,
- The US Food and Drug Administration,
- Any regulatory agency that reviews clinical trials,

FOR REVIEW ONLY

- *Non-US Sites must include; US sites may but are not required to include:*[Insert name of local IRB/EC] ,
- [Insert name of local and/or national regulatory authority as appropriate],
- The HVTN, HPTN and people who work for them,
- The Safety Monitoring Board and
- The US Office for Human Research Protections.

All reviewers will take steps to keep your records private.

We cannot guarantee absolute privacy. If you are found to have a medical condition that we are required to report by law, then some of your information may be shared. At this clinic, we have to report the following information:

Site: Include any public health or legal reporting requirements, including SARS-CoV-2/COVID-19. Bulleted examples should include all appropriate cases (reportable communicable disease, risk of harm to self or others, etc.). If your site does not have public health or legal reporting requirements, you may delete the last sentence in the paragraph above, along with the bullets below.

- [Item 1]
- [Item 2]
- [Item 3]

US sites: Include the following boxed text. You can remove the box around the text.

We have a Certificate of Confidentiality from the US government, to help protect your privacy. With the certificate, we do not have to release information about you to someone who is not connected to the study, such as the courts or police. Sometimes we can't use the certificate. Since the US government funds this research, we cannot withhold information from it. Also, you can still release information about yourself and your study participation to others.

The results of this study may be published. No publication will use your name or identify you personally.

We may share information from the study with other researchers. We will not share your name or information that can identify you.

A description of this clinical trial will be available on http://www.ClinicalTrials.gov. This website will not include information that can identify you. At most, the website will include a summary of the results. You can search this website at any time.

FOR PREVIEW ONLY

18. **There are several reasons why we may stop your infusions. We may stop them even if you were scheduled for more infusions.**

If we stop your infusions, we may ask you to stay in the study to complete other study procedures.

We will stop your infusions if you become pregnant. We will encourage you to stay in the study if you choose. We will discuss your study options with you. If you leave the study while you are still pregnant, we will contact you after your due date to ask some questions about your pregnancy and delivery.

We will stop your infusions if you enroll in a different research study where you get another study product. We will encourage you to stay in the study if you choose.

We will stop your infusions if you acquire HIV. We will also take fewer samples, and we will help you get care and support. We will encourage you to stay in the study for up to 16 weeks after your last infusion or last scheduled protocol clinic visit, whichever comes first. We will discuss your study options with you. We will counsel you about having HIV and about telling your partner(s). *Site: Modify the following sentence as appropriate.* We will not provide or pay for any of your HIV care directly.

In the unlikely event that you have a bad reaction to a study infusion, we may stop your infusions.

19. We may take you out of the study at any time.

We may take you out of the study if:

- you do not follow instructions,
- we think that staying in the study might harm you,
- the study is stopped for any reason.

Other Risks

20. **There are other risks to being in this study.**

This section describes the other risks and restrictions we know about. There may also be unknown risks, even serious ones. We will tell you if we learn anything new that may affect your willingness to stay in the study.

Risks of giving blood:

FOR REVIEW ONLY

Giving blood can cause bruising, pain, fainting, soreness, redness, swelling, itching, bleeding, and infection (rarely). Giving blood can cause a low blood cell count (anemia), making your feel tired.

Risks of SC infusion procedures:

Getting a SC infusion may cause bruising, pain, fainting, soreness, redness, swelling, itching, a sore, bleeding, blood clots, forming a bump under the skin, and (rarely) muscle damage, or infection where you got the SC infusion.

Risks of IV infusion procedures:

Getting an IV may cause stinging, discomfort, pain, soreness, redness, bruising, itching, rash and swelling where the needle goes into the skin. Rarely, needle sticks can result in a blood clot or infection.

Personal problems/discrimination:

Some people who join HVTN and HPTN studies report personal problems or discrimination because of joining an HIV prevention study. Family or friends may worry, get upset or angry, or assume that you have HIV or are at high risk and treat you unfairly as a result. Rarely, a person has lost a job because the study took too much time away from work, or because their employer thought they had HIV.

HIV testing:

HIV antibody tests are the usual way to test for HIV infections. We are still learning how HIV tests perform when people are given monoclonal antibodies. We have used several common HIV antibody tests to test samples of blood containing antibodies similar to the study antibodies. We found that very high levels of these similar antibodies can cause positive or uncertain results on some HIV tests. You may have such high levels for a short time after you get the study antibodies. This means that for a few days after getting the study antibodies, some HIV tests might say you have HIV, even if you don't.

For this reason, you should plan to get HIV tests only at this clinic during the study. Our tests can truly detect HIV. They can also tell if someone does not have HIV. We do not expect you to have any problems with HIV testing after the study ends.

Embarrassment/anxiety:

You may feel embarrassed when we ask about your HIV risks, such as having sex and using drugs. Also, waiting for your HIV test results or other health test results could make you feel anxious. You could feel worried if your test results show that you have HIV. If you feel embarrassed or anxious, please tell us and we will try to help you.

FOR PREVIEW ONLY

Risks of disclosure of your personal information:

We will take several steps to protect your personal information. Although the risk is very low, it is possible that your personal information could be given to someone who should not have it. If that happened, you could face discrimination, stress, and embarrassment. We can tell you more about how we will protect your personal information if you would like it.

Risks of genetic testing:

It is unlikely, but the genetic tests done on your samples could show you may be at risk for certain diseases. If others found out, it could lead to discrimination or other problems. However, it is almost impossible for you or others to know your test results from the genetic testing. The results are not part of your study records and are not given to you.

U.S. Sites, include the following paragraph In the very unlikely event that your genetic information becomes linked to your name, a federal law called the Genetic Information Nondiscrimination Act (GINA) helps protect you. GINA keeps health insurance companies and employers from seeing results of genetic testing when deciding about giving you health insurance or offering you work. GINA does not help or protect you against discrimination by companies that sell life, disability or long-term care insurance.

Unknown risks:

We do not know if the study antibodies will increase, decrease, or not change your risk of getting HIV if you are exposed. If you get HIV, we do not know how the study antibodies might affect your HIV infection or how long it takes to develop AIDS.

We do not know if getting these study antibodies will affect how you respond to any future approved HIV antibody or vaccine. Currently, no HIV antibody or vaccine has been approved for use.

We do not know how the study antibodies will affect a pregnant participant or a developing baby.

Benefits

21. **The study may not benefit you.**

We do not expect the study antibodies to benefit you in any way. However, being in the study might still help you in some ways. The counseling that you get as part of the study may help you avoid getting HIV. The lab tests and physical exams that you get while in this study might detect health problems you don't yet know about.

FOR PREVIEW ONLY

When asked, most study participants say that participating in a study made them feel good about helping others, increased their knowledge about HIV, and improved their self-esteem.

This study may help in the search for an antibody or vaccine to prevent HIV. However, if the study antibodies or a vaccine later become approved and sold, there are no plans to share any money with you.

Your rights and responsibilities

22. **If you join the study, you have rights and responsibilities.**

    You have many rights that we will respect. You also have responsibilities. We list these in the Bill of Rights and Responsibilities for Research. We will give you a copy of it.

Leaving the study

23. **Tell us if you decide to leave the study.**

    You are free to leave the study at any time and for any reason. Your care at this clinic and your legal rights will not be affected, but it is important for you to let us know.

    Previously collected information about you will remain in the study records and will be included in the analysis of results. Your information can not be removed from the study records.

    We may ask you to come back to the clinic one last time for a physical exam, and we may ask to take some blood and urine samples. We may also ask about any personal problems or benefits you have experienced from being in the study. We believe these steps are important to protecting your health, but it is up to you whether to complete them.

Injuries

Sites: Approval from HVTN Regulatory Affairs (at vtn.core.reg@hvtn.org) is needed for any change (other than those that the instructions specifically request or those previously approved by HVTN Regulatory Affairs) to the boxed text. You can remove the box around the text.

24. **If you get sick or injured during the study, contact us immediately.**

2 paragraphs below for all sites.

Your health is important to us. *(Sites: adjust the following 2 sentences if applicable to the care available at your site)* We will tell you about the care that

FOR REVIEW ONLY

we can give here. For the care that we cannot provide, we will explain how we will help you get care elsewhere.

If you become sick or injured in this study, the HVTN has a process to decide if it is related to the [study product(s)] and/or procedures. If it is, we call it a study-related injury. There are funds to pay for treatment of study-related injuries if certain conditions are met.

Next paragraph for non-US sites.

Sites: adjust the language in this paragraph so it is applicable to your site. Note that the ABPI guidelines apply to South Africa only. In this study, our clinic has insurance to cover your medical treatment in the case of a study-related injury. We will follow the Association of the British Pharmaceutical Industry guidelines for payment of study-related injury. We can give you a copy of these guidelines. In rare cases, the insurance funds may not be enough.

In this situation, the HVTN has limited funds to pay medical costs that it determines are reasonable. *(Sites: insert locale- appropriate medical insurance language in the following sentence)* If the injury is not study related, then you and/or your health insurance will be responsible for treatment costs.

Next paragraph for US .

The HVTN has limited funds to pay medical costs that it determines are reasonable. *(Sites: insert locale- appropriate medical insurance language in the following sentence)* If the injury is not study related, then you and/or your health insurance will be responsible for treatment costs.

2 paragraphs below for all sites.

Some injuries are not physical. For example, you might be harmed emotionally by being in an HIV prevention study. Or you might lose wages because you cannot go to work. However, there are no funds to pay for these kinds of injuries, even if they are study related.

You may disagree with the decision about whether your injury is study related. If you wish, the HVTN will ask independent experts to review the decision. You always have the right to use the court system if you are not satisfied.

FOR REVIEW ONLY

Questions

25. **If you have questions or problems at any time during your participation in this study, use the following important contacts.**

If you have questions about this study, contact
[name or title and telephone number of the investigator or other study staff].

If you have any symptoms that you think may be related to this study, contact
[name or title and telephone number of the investigator or other study staff].

This study has been reviewed and approved by a committee called the [name of local IRB/EC]. If you have questions about your rights as a research participant, or problems or concerns about how you are being treated in this study, contact [name or title and telephone number of person on IRB/EC] , at the committee.

US sites include if this sIRB will be used: This study has been reviewed and approved by a committee called the Fred Hutchinson Cancer Research Center Institutional Review Board. If you have questions about your rights as a research participant, or problems with or concerns about how you are being treated in this study, contact the Director at 206-667-5900 or irodirector@fredhutch.org.

Paragraph for South African sites. The study has been structured in accordance with the Declaration of Helsinki (last updated October 2013) which deals with the recommendations guiding doctors in biomedical research involving human participants, the Ethics in Health Research: Principles, Structures and Processes Second Edition 2015, and Guidelines for Good Practice in the Conduct of Clinical Trials in Human Participants in South Africa. We can provide you with copies of these guidelines if you wish to review them. In addition, the recent Protection of Personal Information Act (POPIA) ensures that all South African institutions conducts themselves in a responsible manner when collecting, processing, storing and sharing another entity's personal information by holding them accountable should they abuse or compromise your personal information in any way.If you want to leave this study, contact
[name or title and telephone number of the investigator or other study staff].

Remainder of section for South African sites only.

You can reach a study staff member 24 hours a day at [telephone number].

If you have questions about this trial you should first discuss them with your doctor or the ethics committee (contact details as provided on this form). After you have consulted your doctor or the ethics committee and if they have not provided you with answers to your satisfaction, you should write to the South African Health Products Regulatory Authority (SAHPRA) at:

FOR REVIEW ONLY

The Chief Executive Officer
Dr Boitumelo Semete-Makokotlela
South African Health Products Regulatory Authority Private Bag X828
PRETORIA
0001
Tel: (012) 501 0410
e-mail: Boitumelo.Semete@sahpra.org.za

Your permissions and signature

Site: Delete this section if using a separate consent for use of samples and information in other studies

26. **In Section 16 of this form, we told you about possible other uses of your extra samples and information, outside this study. Please choose only one of the options below and write your initials or make your mark in the box next to it. Whatever you choose, the HVTN and HPTN keep track of your decision about how your samples and information can be used. You can change your mind after signing this form.**

☐ I allow my extra samples and information to be used for other studies related to HIV, vaccines, antibodies, the immune system, and other diseases. This may include genetic testing.

OR

☐ I agree to the option above *and* also to allow my extra samples and information to be used in genome wide studies.

OR

☐ I do not allow my extra samples to be used in any other studies. This includes not allowing genetic testing or genome wide studies.

27. **If you agree to join this study, you will need to sign or make your mark below. Before you sign or make your mark on this consent form, make sure of the following:**

- You have read this consent form, or someone has read it to you.
- You feel that you understand what the study is about and what will happen to you if you join. You understand what the possible risks and benefits are.
- You have had your questions answered and know that you can ask more.
- You agree to join this study.

FOR PREVIEW ONLY

You will not be giving up any of your rights by signing this consent form.

Participant's name (print)	Participant's signature or mark	Date	Time
Clinic staff conducting consent discussion (print)	Clinic staff signature	Date	Time

For participants who are unable to read or write, a witness should complete the signature block below:

Witness's name (print)	Witness's signature	Date	Time

*Witness is impartial and was present for the entire discussion of this consent form.

FOR REVIEW ONLY

Appendix C Approved birth control methods for persons assigned female sex at birth (for sample informed consent form)

Site: Any change to the following boxed text requires approval from HVTN Regulatory Affairs at vtn.core.reg@hvtn.org. You can remove the box around the text.

You should not become pregnant during the study because we do not know how the study antibodies could affect the developing baby.

You must agree to use effective birth control from 21 days before your first infusion until last scheduled protocol clinic visit.

Effective birth control means using one of the following methods every time you have sex:

- Birth control drugs that prevent pregnancy—given by pills, shots, patches, vaginal rings, or inserts under the skin;
- Internal or external condoms, with or without a cream or gel that kills sperm;
- Diaphragm or cervical cap with a cream or gel that kills sperm;
- Intrauterine device (IUD); or
- Any other contraceptive method approved by the researchers.

You do not have to use birth control if:

- You are only having sex with a partner or partners who have had a vasectomy. (We will ask you some questions to confirm that the vasectomy was successful.);
- You have reached menopause, with no menstrual periods for one year;
- You have had a hysterectomy (your uterus removed);
- You have had your ovaries removed;
- You have a tubal ligation (your "tubes tied") or confirmed successful placement of a product that blocks the fallopian tubes;
- You are having sex only with a partner(s) assigned female sex at birth;
- You only have oral sex; or,
- You are sexually abstinent (no sex at all).

FOR REVIEW ONLY

Remember: If you are having sex, internal and external condoms (also known as male and female condoms) are the only birth control methods that also provide protection against HIV and other sexually transmitted infections.

If you join the study, we will test you for pregnancy at some visits, including before each studyinfusion.

FOR REVIEW ONLY

Appendix D Sample consent form for use of samples and information in other studies

Title: A phase 1 dose-escalation clinical trial to evaluate the safety, tolerability, and pharmacokinetics of PGDM1400LS alone and in combination with VRC07-523LS and PGT121.414.LS in healthy, HIV-uninfected adult participants<br>Protocol number: HVTN 140/HPTN 101<br>Site: [Insert site name]

When samples are no longer needed for this study, the HVTN and HPTN want to use them in other studies and share them with other researchers. The HVTN and HTPN call these samples called "extra samples." The HVTN and HPTN will only allow your extra samples to be used in other studies if you agree to this. You will mark your decision at the end of this form. If you have any questions, please ask.

1. Do I have to agree?

No. You are free to say yes or no, or to change your mind after you sign this form. At your request, we will destroy all extra samples that we have. Your decision will not affect your being in this study or have any negative consequences here.

2. Where are the samples stored?

Extra samples are stored in a secure central place called a repository. *[Site: choose one of the following two sentences. African sites should choose the sentence referencing the repository in South Africa. All other sites should choose the sentence referencing the repository in the United States.]* Your samples will be stored in the HVTN repository in South Africa. Your samples will be stored in the HVTN repository in the United States.

3. How long will the samples be stored?

There is no limit on how long your extra samples will be stored. *[Site: Revise the previous sentence to insert limits if your regulatory authority imposes them.]*

4. Will I be paid for the use of my samples?

No. Also, a researcher may make a new scientific discovery or product based on the use of your samples. If this happens, there is no plan to share any money with you. The researcher is not likely to ever know who you are.

5. Will I benefit from allowing my samples to be used in other studies?

Probably not. Results from these other studies are not given to you, this clinic, or your doctor. They are not part of your medical record. The studies are only being done for research purposes.

FOR REVIEW ONLY

6. **Will the HVTN or HPTN sell my samples and information?**

   No, but the HVTN and HPTN may share your samples with HVTN, HPTN, or other researchers. Once we share your samples and information, we may not be able to get them back.

7. **How do other researchers get my samples and information?**

   When a researcher wants to use your samples and information, their research plan must be approved by the HVTN and HPTN. Also, the researcher's institutional review board (IRB) or ethics committee (EC) will review their plan. *[Site: If review by your institution's IRB/EC/RE is also required, insert a sentence stating this.]* IRBs/ECs protect the rights and well-being of people in research. If the research plan is approved, the HVTN will send your samples to the researcher's location.

8. **What information is shared with HVTN, HPTN, or other researchers?**

   The samples and information will be labeled with a code number. Your name will not be part of the information. However, some information that we share may be personal, such as your race, ethnicity, gender, health information from the study, and HIV status. We may share information about the study antibodies you received and how your body responded to the studyantibodies.

9. **What kind of studies might be done with my extra samples and information?**

   The studies will be related to HIV, vaccines, antibodies, the immune system, and other diseases.

   Researchers may also do genetic testing on your samples.

   If you agree, your samples could also be used for genome wide studies. In these studies, researchers will look at all of your genes (your genome). The researchers compare the genomes of many people, looking for common patterns of genes that could help them understand diseases. The researchers may put the information from the genome-wide studies into a protected database so that other researchers can access it, but your name and other personal information will not be included. Usually, no one would be able to look at your genome and link it to you as a person. However, if another database exists that also has information on your genome and your name, someone might be able to compare the databases and identify you. If others found out, it could lead to discrimination or other problems. The risk of this is very small. There may be other unknown risks.

10. **What are the risks of genetic testing?**

    It is unlikely, but the genetic tests done on your samples could show you may be at risk for certain diseases. If others found out, it could lead to discrimination or other problems. However, it is almost impossible for you or others to know your

FOR REVIEW ONLY

test results from the genetic testing. The results are not part of your study records and are not given to you.

US Sites, include the following paragraph

In the very unlikely event that your genetic information becomes linked to your name, a federal law called the Genetic Information Nondiscrimination Act (GINA) helps protect you. GINA keeps health insurance companies and employers from seeing results of genetic testing when deciding about giving you health insurance or offering you work. GINA does not help or protect you against discrimination by companies that sell life, disability or long-term care insurance.

11. Who will have access to my information in studies using my extra samples?

People who may see your information are:

- Researchers who use your extra samples and information for other research
- Government agencies that fund or monitor the research using your extra samples and information
- Any regulatory agency that reviews clinical trials
- The researcher's Institutional Review Board or Ethics Committee
- The people who work with the researcher

All of these people will do their best to protect your information. The results of any new studies that use your extra samples and information may be published. No publication will use your name or identify you personally.

Questions

12. If you have questions or problems about allowing your samples and information to be used in other studies, use the following important contacts.

If you have questions about the use of your samples or information or if you want to change your mind about their use, contact
[name or title and telephone number of the investigator or other study staff].

If you think you may have been harmed because of studies using your samples or information, contact
[name or title and telephone number of the investigator or other study staff].

If you have questions about your rights as a research participant, contact
[name or title and telephone number of person on IRB/EC .

FOR REVIEW ONLY

Paragraph for South African sites. The study has been structured in accordance with the Declaration of Helsinki (last updated October 2013) which deals with the recommendations guiding doctors in biomedical research involving human participants, the Ethics in Health Research: Principles, Structures and Processes Second Edition 2015, and Guidelines for Good Practice in the Conduct of Clinical Trials in Human Participants in South Africa. We can provide you with copies of these guidelines if you wish to review them.

Remainder of section for South African sites only.

You can reach a study staff member 24 hours a day at [telephone number].

If you have questions about this trial you should first discuss them with your doctor or the ethics committee (contact details as provided on this form). After you have consulted your doctor or the ethics committee and if they have not provided you with answers to your satisfaction, you should write to the South African Health Products Regulatory Authority (SAHPRA) at:

The Chief Executive Officer
Dr Boitumelo Semete-Makokotlela
South African Health Products Regulatory Authority Private Bag X828
PRETORIA
0001
Tel: (012) 501 0410
e-mail: Boitumelo.Semete@sahpra.org.za

FOR REVIEW ONLY

13. **Please choose only one of the options below and write your initials or make your mark in the box next to it. Whatever you choose, the HVTN and HPTN keep track of your choice about how your samples and information can be used. You can change your mind after signing this form.**

☐ I allow my extra samples and information to be used for other studies related to HIV, vaccines, antibodies, the immune system, and other diseases. This may include genetic testing.

OR

☐ I agree to the option above *and* also to allow my extra samples and information to be used in genome wide studies.

OR

☐ I do not allow my extra samples to be used in any other studies. This includes not allowing genetic testing or genome wide studies.

Participant's name (print)	Participant's signature or mark	Date	Time
Clinic staff conducting consent discussion (print)	Clinic staff signature	Date	Time

For participants who are unable to read or write, a witness should complete the signature block below:

Witness's name (print)	Witness's signature	Date	Time

*Witness is impartial and was present for the entire discussion of this consent form.

FOR REVIEW ONLY

Appendix E Table of procedures (for sample informed consent form)

**For Part A**	Time after enrollment infusion visit							
Procedure	Screening visit(s)	Infusion visit	3 days	6 days	1 month	2 months	4 months	6 months
Infusion		√						
Medical history	√							
Complete physical	√							√
Brief physical		√	√	√	√	√	√	
Urine test	√					√		√
Blood drawn	√	√	√	√	√	√	√	√
Pregnancy test (participants assigned female sex at birth)*	√	√						√
HIV testing and pretest counseling	√						√	√
Risk reduction counseling	√	√			√	√	√	√
Interview/questionnaire	√	√			√	√	√	√

Not shown in this table is a time after all study participants have completed their last scheduled visit when you can find out what products you received.

* Persons who had a hysterectomy (removal of the uterus) or removal of both ovaries (verified by medical records), are not required to have a pregnancy test.

FOR REVIEW ONLY

**For Part B**		Time after first infusion visit									
Procedure	Screening visit(s)	First infusion visit	3 days	6 days	1 month	2 months	4 months	4 months + 4 days	6 months	8 months	10 months
Infusion		√					√				
Medical history	√										
Complete physical	√										√
Brief physical		√	√	√	√	√	√		√	√	
Urine test	√					√			√		√
Blood drawn	√	√	√	√	√	√	√		√	√	√
Pregnancy test (participants assigned female sex at birth)*	√	√					√				√
HIV testing and pretest counseling	√						√			√	√
Risk reduction counseling	√	√			√	√	√		√	√	√
Interview/questionnaire	√	√		√	√	√	√	√	√	√	√

Not shown in this table is a time after all study participants have completed their last scheduled visit when you can find out what products you received.

* Persons who had a hysterectomy (removal of the uterus) or removal of both ovaries (verified by medical records), are not required to have a pregnancy test.

FOR REVIEW ONLY

Appendix F Laboratory procedures for Part A

				Visit:	1	2	3	4	5	6	7	8	9
				Day:	Screening visit[3]	D0	D3	D6	D28	D56	D112	D116	D168
				Week:		W0			W4	W8	W16		W24
						**Study Product Administration**							
**Procedure**	**Ship to[1]**	**Assay location[2]**	**Tube Type[4]**	**Tube size (vol. capacity)[4]**		**PGDM1400LS**							
BLOOD COLLECTION													
Screening/Diagnostic													
Screening HIV test	Local lab	Local lab	EDTA	5mL	5	—	—	—	—	—	—		—
HBsAg/anti-HCV	Local lab	Local lab	SST	5mL	5	—	—	—	—	—	—		—
Syphilis[9]	Local lab	Local lab	SST	5mL	5	—	—	—	—	—	—		—
HIV diagnostics[7]	UW-VSL / HSML-NICD	UW-VSL / HSML-NICD	EDTA	10mL	—	—	—	—	—	—	—		10
Safety labs[10]													
CBC/ Differential	Local lab	Local lab	EDTA	5mL	5	5	—	—	—	5	—		5
Chemistry Panel[5]	Local lab	Local lab	SST	5mL	5	5	—	—	—	5	—		5
Drug concentrations/detection													
PGDM1400LS concentration	CSR	HVTN Labs	SST	8.5mL	—	y	y	y	y	y	y		y
Humoral assays													
HIV-1 neutralizing Ab	CSR	HVTN Labs	SST	8.5mL	—	y	y	y	y	y	y		y
Non-neutralizing antiviral assays	CSR	HVTN Labs	SST	8.5mL	—	y	y	y	y	y	y		y
Anti-Drug Antibody (ADA)													
ADA detection assays (screening, confirmatory, titration)	CSR	HVTN Labs	SST	8.5mL	—	y	—	—	—	—	—		y
ADA functional assay	CSR	HVTN Labs	SST	8.5mL	—	y	—	—	—	—	—		y
Ab Reaction[11]													
Tryptase / C3 and C4 Complement / Cytokines	CSR	ARUP	SST	8.5mL	—	See footnote 12	—	—	See footnote 12				See footnote 12
ADA detection assays (screening, confirmatory, titration)	CSR	HVTN Labs	SST	8.5mL	—	See footnote y	—	—	—	—	—		—
ADA functional assay	CSR	HVTN Labs	SST	8.5mL	—	See footnote y	—	—	—	—	—		—
STORAGE													
Serum	CSR	—	SST	8.5mL	—	76.5[14]	42.5	42.5	42.5	42.5	42.5		42.5
**Visit total**					**25**	**86.5**	**42.5**	**42.5**	**42.5**	**52.5**	**42.5**		**62.5**
**56-Day total[13]**					**25**	**112**	**154.0**	**197**	**239**	**292**	**95**		**105**
**URINE COLLECTION[10]**													
Urine dipstick[8]	Local lab	Local lab			X	—	—	—	—	X	—		X
Pregnancy test[6]	Local lab	Local lab			X	X[15]	—	—	—	—	—		X

FOR REVIEW ONLY

FOR REVIEW ONLY

Footnotes for Appendix F

Greyed-out column does not apply to Part A.

[1]CSR = central specimen repository; UW-VSL = University of Washington Virology Specialty Laboratory (Seattle, Washington, USA); HSML-NICD = HIV Sero-Molecular Laboratory-National Institute for Communicable Diseases (Johannesburg, South Africa)

[2]HVTN Laboratories include: Duke University Medical Center (Durham, North Carolina, USA); South African Immunology Laboratory-National Institute for Communicable Diseases (SAIL-NICD, Johannesburg, South Africa); Dartmouth College (Hanover, New Hampshire, USA).Non-HVTN laboratories: ARUP Laboratories (Salt Lake City, Utah, USA).

[3]Screening may occur over the course of several contacts/visits up to and including day 0 prior to study product administration.

[4]Local labs may assign appropriate alternative specimen type or tube types for locally performed tests.

[5]Chemistry panels are defined in Section 9.2 (pre-enrollment), and Sections 9.3 and 9.4 (enrollment and follow-up).

[6]For participants assigned female sex at birth, pregnancy test must be performed on urine or blood specimens on the day of study product administration with negative results received prior to administration. Persons who are NOT of reproductive potential due to having undergone hysterectomy or bilateral oophorectomy (verified by medical records), are not required to undergo pregnancy testing.

[7]At an early termination visit for a withdrawn or terminated participant who is not HIV-infected (see Section 9.10), blood should be drawn for HIV diagnostic testing, as shown for visit 15 above. If a participant has a confirmed diagnosis of HIV infection, do not collect blood for HIV diagnostic testing (see Section 9.12).

[8]And microscopy if needed.

[9]Syphilis testing will be done by serology.

[10]For participants with confirmed diagnosis of HIV infection, only specimens required for protocol-specified safety laboratory tests, urinalysis and pregnancy tests will be collected.

[11]To investigate Ab administration-related clinical reactions, assays may be performed on serum samples taken prior to the study product administration associated with the reaction and collected after the onset of reaction. Refer to the SSP for more information.

[12]SST blood will be collected at specific time points after the onset of any Ab reaction. Refer to the SSP for more information.

[13]The 56-day total blood volume does not include up to 34mL SST blood collected for any Ab reaction; however, the 56-day limit is not exceeded at any visit by the possible collection of SST blood for an Ab reaction.

[14]Of this volume, 8.5mL of SST blood will be **collected post study product administration** (see SSP for details).

[15] Pregnancy test at enrollment does not need to be performed if negative results are received from screening pregnancy test conducted within 48 hours prior to study product administration.

y = SST blood collected for serum storage will also cover specimen needs for drug concentrations, HIV-1 neutralizing Ab assays, non-neutralizing antiviral assays, and ADA detection and functional assays (including for any Ab reactions); no separate blood draw is needed.

Appendix G Laboratory procedures for Part B

Procedure	Ship to[1]	Assay location[2]	Tube Type[4]	Tube size (vol. capacity)[4]		Study Product Administration #1<br>PGDM1400LS + VRC07-523LS + PGT121.414.LS					Study Product Administration #2<br>PGDM1400LS + VRC07-523LS + PGT121.414.LS				
BLOOD COLLECTION															
Screening/Diagnostic															
Screening HIV test	Local lab	Local lab	EDTA	5mL	5	—	—	—	—	—	—	—	—	—	—
HBsAg/anti-HCV	Local lab	Local lab	SST	5mL	5	—	—	—	—	—	—	—	—	—	—
Syphilis[9]	Local lab	Local lab	SST	5mL	5	—	—	—	—	—	—	—	—	—	—
HIV diagnostics[7]	UW-VSL / HSML-NICD	UW-VSL / HSML-NICD	EDTA	10mL	—	—	—	—	—	—	10	—	—	—	10
Safety labs[10]															
CBC/ Differential	Local lab	Local lab	EDTA	5mL	5	5	—	—	—	5	5	—	5	—	5
Chemistry Panel[5]	Local lab	Local lab	SST	5mL	5	5	—	—	—	5	5	—	5	—	5
Drug concentrations/detection															
PGDM1400LS, VRC07-523LS, PGT121.414.LS concentrations	CSR	HVTN Labs	SST	8.5mL	—	y	y	y	y	y	y	—	y	y	y
Humoral assays															
HIV-1 neutralizing Ab	CSR	HVTN Labs	SST	8.5mL	—	y	y	y	y	y	y	—	y	y	y
Non-neutralizing antiviral assays	CSR	HVTN Labs	SST	8.5mL	—	y	y	y	y	y	y	—	y	y	y
Anti-Drug Antibody (ADA)															
ADA detection assays (screening, confirmatory, titration)	CSR	HVTN Labs	SST	8.5mL	—	y	—	—	—	—	y	—	—	—	y
ADA functional assay	CSR	HVTN Labs	SST	8.5mL	—	y	—	—	—	—	y	—	—	—	y
Ab Reaction[11]															
Tryptase / C3 and C4 Complement / Cytokines	CSR	ARUP	SST	8.5mL	—	See footnote 12	—	—	See footnote 12			—	See footnote 12		
ADA detection assays (screening, confirmatory, titration)	CSR	HVTN Labs	SST	8.5mL	—	See footnote y	—	—	—	—	See footnote y	—	—	—	—
ADA functional assay	CSR	HVTN Labs	SST	8.5mL	—	See footnote y	—	—	—	—	See footnote y	—	—	—	—
STORAGE															
Serum	CSR	—	SST	8.5mL	—	76.5[14]	42.5	42.5	42.5	42.5	51[14]	—	42.5	42.5	42.5
**Visit total**					**25**	**86.5**	**42.5**	**42.5**	**42.5**	**52.5**	**71**	**0**	**52.5**	**42.5**	**62.5**
**56-Day total**[13]					**25**	**112**	**154**	**197**	**239**	**292**	**71**	**71**	**124**	**95**	**105**
**URINE COLLECTION**[10]															
Urine dipstick[8]	Local lab	Local lab			X	—	—	—	—	X	—	—	X	—	X
Pregnancy test[6]	Local lab	Local lab			X	X[15]	—	—	—	—	X	—	—	—	X

FOR REVIEW ONLY

FOR REVIEW ONLY

Footnotes for Appendix G

[1]CSR = central specimen repository; UW-VSL = University of Washington Virology Specialty Laboratory (Seattle, Washington, USA); HSML-NICD = HIV Sero-Molecular Laboratory-National Institute for Communicable Diseases (Johannesburg, South Africa)

[2]HVTN Laboratories include: Duke University Medical Center (Durham, North Carolina, USA); South African Immunology Laboratory-National Institute for Communicable Diseases (SAIL-NICD, Johannesburg, South Africa); Dartmouth College (Hanover, New Hampshire, USA). Non-HVTN laboratories: ARUP Laboratories (Salt Lake City, Utah, USA)

[3]Screening may occur over the course of several contacts/visits up to and including day 0 prior to study product administration.

[4]Local labs may assign appropriate alternative specimen type or tube types for locally performed tests.

[5]Chemistry panels are defined in Section 9.2 (pre-enrollment), and Sections 9.3 and 9.4 (enrollment and follow-up).

[6]For participants assigned female sex at birth, pregnancy test must be performed on urine or blood specimens on the day of study product administration with negative results received prior to administration. Persons who are NOT of reproductive potential due to having undergone hysterectomy or bilateral oophorectomy (verified by medical records), are not required to undergo pregnancy testing.

[7]At an early termination visit for a withdrawn or terminated participant who is not HIV-infected (see Section 9.10), blood should be drawn for HIV diagnostic testing, as shown for visit 20 above. If a participant has a confirmed diagnosis of HIV infection, do not collect blood for HIV diagnostic testing (see Section 9.12).

[8]And microscopy if needed.

[9]Syphilis testing will be done by serology.

[10]For participants with confirmed diagnosis of HIV infection, only specimens required for protocol-specified safety laboratory tests, urinalysis and pregnancy tests will be collected.

[11]To investigate Ab administration-related clinical reactions, assays may be performed on serum samples taken prior to the study product administration associated with the reaction and collected after the onset of reaction. Refer to the SSP for more information.

[12]SST blood will be collected at specific time points after the onset of any Ab reaction. Refer to the SSP for more information.

[13]The 56-day total blood volume does not include up to 51mL SST blood collected for any Ab reaction; however, the 56-day limit is not exceeded at any visit by the possible collection of SST blood for an Ab reaction.

[14]Of this volume, 8.5mL of SST blood will be **collected post study product administration (**see SSP for details).

[15]Pregnancy test at enrollment does not need to be performed if negative results are received from screening pregnancy test conducted within 48 hours prior to study product administration .

[16]Phone contact only. No specimen collection at this visit.

y = SST blood collected for serum storage will also cover specimen needs for drug concentrations, HIV-1 neutralizing Ab assays, non-neutralizing antiviral assays, and ADA detection and functional assays (including for any Ab reactions); no separate blood draw is needed.

Appendix H Procedures at HVTN CRS for Part A

Visit	01[1]	02[2]	03	04	05	06	07	08	09	Post
Day:		D0	D3	D6	D28	D56	D112	D116	D168	
Week:		W0	W0	W0	W4	W8	W16	W16	W24	
Procedure	Scr	Inf								
**Study procedures**										
Signed screening consent (if used)	X	—	—	—	—	—	—		—	—
Assessment of understanding	X	—	—	—	—	—	—		—	—
Signed protocol consent	X	—	—	—	—	—	—		—	—
Medical history	X	—	—	—	—	—	—		—	—
Complete physical exam	X	—	—	—	—	—	—		X	—
Confirm eligibility, obtain demographics, randomize	X	—	—	—	—	—	—		—	—
**Infusion**	—	X	—	—	—	—	—		—	—
Solicited AE assessment[3]	—	X	X[3]	X[3]	—	—	—		—	—
Abbreviated physical exam	—	X	X	X	X	X	X		—	—
Risk reduction counseling[4]	X	X	—	—	X	X	X		X	—
Contraception status assessment[5]	X	X	—	—	X	X	X		X	—
Social impact assessment	—	X	—	—	X	X	X		X	—
Behavioral risk assessment questionnaire[6]	X	—	—	—	—	—	—		X	—
Social impact assessment questionnaire	—	—	—	—	—	X	—		X	—
Acceptability questionnaire	—	X	—	—	—	—	—		—	—
Outside testing questionnaire	—	—	—	—	—	—	—		X	
Concomitant medications	X	X	X	X	X	X	X		X	—
Intercurrent illness/Unsolicited AE assessment	—	X	X	X	X	X	X		X	—
HIV infection assessment[7]	X	—	—	—	—	—	X		X	—
Confirm HIV test results provided to participant	—	X	—	—	—	—	—		X	X
**Specimen collection** [8]	X	X	X	X	X	X	X		X	—

FOR REVIEW ONLY

Greyed-out column does not apply to Part A.

Footnotes for Appendix H

[1] Screening may occur over the course of several contacts/visits up to and including day 0 prior to study product administration.

[2] Specimens collected at Day 0 may be obtained within the 14 days prior to study product administration, except for a pregnancy test, which must be performed on urine or blood specimens within 48 hours prior to study product administration with negative results received prior to study product administration.

[3] Solicited AE assessments are performed daily for at least 3 full days following study product administration. CRS staff will review and reconcile the diary with the participant and then report Solicited AEs. Participant diary reconciliation may happen as the data is available (see the HVTN 140/HPTN 101 SSP).

[4] Includes transmission risk reduction counseling for HIV-infected participants.

[5] Contraception status assessment is required only for participants who were assigned female sex at birth and who are capable of becoming pregnant.

[6] Not applicable to HIV-infected participants.

[7] Includes pretest counseling and HIV testing. A subsequent follow-up contact is conducted to provide post-test counseling and to report results to participant. Not applicable for participants diagnosed with HIV infection.

[8] For specimen collection requirements, see Appendix F.

FOR REVIEW ONLY

Appendix I Procedures at HVTN CRS for Part B

Visit	01[1]	02[2]	03	04	05	06	07	08	09	10	11	Post
**Day:**		D0	D3	D6	D28	D56	D112	D116	D168	D224	D280	
**Week:**		W0	W0	W0	W4	W8	W16	W16	W24	W32	W40	
**Procedure**	Scr	Inf 1					Inf 2	Phone contact				
**Study procedures**												
Signed screening consent (if used)	X	—	—	—	—	—	—		—	—	—	—
Assessment of understanding	X	—	—	—	—	—	—		—	—	—	—
Signed protocol consent	X	—	—	—	—	—	—		—	—	—	—
Medical history	X	—	—	—	—	—	—		—	—	—	—
Complete physical exam	X	—	—	—	—	—	—		—	—	X	—
Confirm eligibility, obtain demographics, randomize	X	—	—	—	—	—	—		—	—	—	—
**Infusion**	—	X	—	—	—	—	X		—	—	—	—
Solicited AE assessment[3]	—	X	X[3]	X[3]	—	—	X[3]	X[3]	X[3]	—	—	—
Abbreviated physical exam	—	X	X	X	X	X	X		X	X	—	—
Risk reduction counseling[4]	X	X	—	—	X	X	X		X	X	X	—
Contraception status assessment[5]	X	X	—	—	X	X	X		X	X	X	—
Social impact assessment	—	X	—	—	X	X	X		X	X	X	—
Behavioral risk assessment questionnaire[6]	X	—	—	—	—	—	—		—	—	X	—
Social impact assessment questionnaire	—	—	—	—	—	X	—		X	—	X	—
Acceptability questionnaire	—	X	—	—	—	—	X		—	—	—	—
Outside testing questionnaire	—	—	—	—	—	—	—		—	—	X	
Concomitant medications	X	X	X	X	X	X	X		X	X	X	—
Intercurrent illness/Unsolicited AE assessment	—	X	X	X	X	X	X		X	X	X	—
HIV infection assessment[7]	X	—	—	—	—	—	X		—	X	X	—
Confirm HIV test results provided to participant	—	X	—	—	—	—	—		X	—	X	X
**Specimen collection** [8]	X	X	X	X	X	X	X		X	X	X	—

FOR REVIEW ONLY

FOR REVIEW ONLY

Footnotes for Appendix I

[1] Screening may occur over the course of several contacts/visits up to and including day 0 prior to study product administration.

[2] Specimens collected at Day 0 may be obtained within the 14 days prior to study product administration, except for a pregnancy test, which must be performed on urine or blood specimens within 48 hours prior to study product administration with negative results received prior to study product administration.

[3] Solicited AE assessments are performed daily for at least 3 full days following study product administration. CRS staff will review and reconcile the diary with the participant and then report Solicited AEs. Participant diary reconciliation may happen as the data is available (see the HVTN 140/HPTN 101 SSP). For Part B participants' second infusion solicited AE data collection: remote documentation of the participant diary may occur after the solicited AE assessment period and the next clinic visit (Visit 9), ideally within 2 weeks post-infusion (see the HVTN 140/HPTN 101 SSP).

[4] Includes transmission risk reduction counseling for HIV-infected participants.

[5] Contraception status assessment is required only for participants who were assigned female sex at birth and who are capable of becoming pregnant.

[6] Not applicable to HIV-infected participants.

[7] Includes pretest counseling and HIV testing. A subsequent follow-up contact is conducted to provide post-test counseling and to report results to participant. Not applicable for participants diagnosed with HIV infection.

[8] For specimen collection requirements, see Appendix G.

Appendix J HVTN low risk guidelines for the US

The following are intended as guidelines for the investigator to help identify potential vaccine trial participants at "low risk" for HIV infection. These guidelines are based on behaviors within the last 6-12 months prior to enrollment; however, it may be appropriate to consider a person's behavior over a longer period of time than specified to assess the person's likelihood of maintaining low risk behavior. *Some volunteers may not be appropriate for enrollment even if they meet these guidelines.* These guidelines should be supplemented and interpreted with local epidemiologic information about HIV prevalence in your area and community networks. The investigator may review the risk level of any volunteer with the site PI and/or the Protocol Safety Review Team.

A volunteer may be appropriate for inclusion if he/she/they meets these guidelines:

A. For US volunteers NOT on stable Pre-exposure prophylaxis (PrEP)

1. SEXUAL BEHAVIORS

In the **last 12 months** did not:

- Have oral, vaginal or anal intercourse with an HIV-infected partner, or a partner who uses injection drugs
- Give or receive money, drugs, gifts or services in exchange for oral, vaginal or anal sex

AND

In the **last 6 months** has abstained from penile/anal or penile/vaginal intercourse, OR

In the **last 6 months**:

- Had 4 or fewer partners of the opposite birth sex for vaginal and/or anal intercourse, OR

Is an MSM (person born male with partner(s) born male) who, in the **last 12 months**:

- Had 2 or fewer MSM partners for anal intercourse and had no unprotected anal sex with MSM, OR
- Had unprotected anal intercourse with only 1 MSM partner, within a monogamous relationship lasting at least 12 months (during which neither partner had any other partners). If the monogamous relationship ended, the volunteer may then have had protected anal intercourse with 1 other MSM partner (total 2 or fewer partners in the last 12 months).

Is a transgender person, regardless of the point on the transition spectrum, having sex with men (born male) and/or other transgender persons, who in the **last 12 months**:

- Had 2 or fewer partners for anal or vaginal intercourse, and had no unprotected anal or vaginal sex, OR
- Had unprotected anal or vaginal intercourse sex with 1 partner only within a monogamous relationship lasting at least 12 months (during which neither partner had any other partners). If the monogamous relationship ended, may

FOR REVIEW ONLY

then have had protected anal or vaginal sex with 1 other partner (total 2 or fewer partners in the last 12 months).

AND
Uses or intends to use condoms in situations which may include penile/anal or

penile/vaginal intercourse with new partners of unknown HIV status, occasional partners,

partners outside a primary relationship, and/or partners known to have other partners.

2. NON-SEXUAL BEHAVIORS
In the **last 12 months** did not:

- Inject drugs or other substances without a prescription
- Use cocaine, methamphetamine, or excessive alcohol, which in the investigator's judgment, rendered the participant at greater than low risk for acquiring HIV infection.
  The investigator's judgment should consider local epidemiologic information about HIV prevalence in the area and community networks.

A volunteer is NOT appropriate for inclusion if he/she:

Acquired an STI (ie, new infection) in the **last 12 months**:

- Syphilis
- Gonorrhea
- Non-gonococcal urethritis
- Herpes Simplex Virus type 2 (HSV2)
- Chlamydia
- Pelvic inflammatory disease (PID)
- Trichomonas
- Mucopurulent cervicitis
- Epididymitis
- Proctitis
- Lymphogranuloma venereum
- Chancroid
- Hepatitis B

B. For US volunteers on Pre-exposure prophylaxis (PrEP)

1. PrEP ASSESSMENT

- Reports equal to or greater than six months of protective PrEP use
- Commits to maintaining protective PrEP use throughout trial
- Participant reports equal to or greater than 70% when asked the following:

"*Thinking about the past 4 weeks, what percent of the time were you able to take all your PrEP medications?"*

FOR PREVIEW ONLY

2. SEXUAL BEHAVIORS

Persons stably taking PrEP as described above for 6 months or longer are considered low risk of HIV infection, regardless of any sexual behavior that might otherwise be associated with high risk of HIV exposure.

2. NON-SEXUAL BEHAVIORS

In the **last 12 months** did not:

- Inject drugs or other substances without a prescription
- Use cocaine, methamphetamine, or excessive alcohol, which in the investigator's judgment, rendered the participant at greater than low risk for acquiring HIV infection.

The investigator's judgment should consider local epidemiologic information about HIV prevalence in the area and community networks.

FOR REVIEW ONLY

Appendix K HVTN low risk guidelines for Southern Africa

The following are intended as guidelines for the investigator to help identify potential vaccine trial participants at "low risk" for HIV infection. These guidelines are based on behaviors within the last 12 months prior to enrollment; however, it may be appropriate to consider a person's behavior over a longer period of time than specified to assess the person's likelihood of maintaining low risk behavior. Some volunteers may not be appropriate for enrollment even if they meet these guidelines. These guidelines should be supplemented and interpreted with local epidemiologic information about HIV prevalence in your area and community networks. The investigator may review the risk level of any volunteer with the site PI and/or the Protocol Safety Review Team.

Assessment of sexual behaviors

Consider whether a volunteer would be appropriate for inclusion if, within 12 months prior to enrollment, the person:

- Abstained from penile/vaginal and penile/anal intercourse, or
- Was in a mutually monogamous relationship with a partner with a known HIV-uninfected status, or
- Had one partner believed to be HIV-uninfected with whom he/she regularly used condoms for penile/vaginal and penile/anal intercourse.

Exclude a volunteer if:

Within the 12 months prior to enrollment: a history of newly acquired syphilis, gonorrhea, chlamydia, trichomoniasis, active HSV lesions, chancroid, pelvic inflammatory disease (PID), genital sores or ulcers, cervicitis, genital warts of the labia minora, vagina, or cervix, or any other symptomatic genital warts.

FOR REVIEW ONLY

Appendix L Visit windows

Part A

Visit Number	Visit Type	Lower Allowable Window	Lower Target Day	Target Day	Upper Target Day	Upper Allowable Window
01.0	Screening	-56	-		-	-
02.0	**Enrollment[1]** **Infusion**	-	-	0	-	-
03.0	3 days post infusion	-1	-	3	-	+1
04.0	6 days post infusion	-1	-	6	-	+2
05.0	4 weeks post infusion	-7	-3	28	+3	+7
06.0	8 weeks post infusion	-7	-3	56	+3	+7
07.0	16 weeks post infusion	-7	-3	112	+3	+7
08.0						
09.0	24 weeks post infusion	-7	-3	168	+3	+7

Greyed-out visit does not apply to Part A.

Part B

Visit Number	Visit Type	Lower Allowable Window	Lower Target Day	Target Day	Upper Target Day	Upper Allowable Window
01.0	Screening	-56	-		-	-
02.0	**Enrollment** **Infusion #1**	-	-	0	-	-
03.0	3 days post infusion #1	-1	-	3	-	+1
04.0	6 days post infusion #1	-1	-	6	-	+2
05.0	4 weeks post infusion #1	-7	-3	28	+3	+7
06.0	8 weeks post infusion #1	-7	-3	56	+3	+7
07.0	**Infusion #2**	-14	-7	112	+7	+14
08.0	4 days post infusion #2	-	-	116	+7	-
09.0	8 weeks post infusion #2	-7	-3	168	+3	+7
10.0	16 weeks post infusion #2	-7	-3	224	+3	+7
11.0	24 weeks post infusion #2	-7	-3	280	+3	+7

FOR REVIEW ONLY

Appendix M Protocol Signature Page

A phase 1 dose-escalation clinical trial to evaluate the safety, tolerability, and pharmacokinetics of PGDM1400LS alone and in combination with VRC07-523LS and PGT121.414.LS in healthy, HIV-uninfected adult participants

I will conduct the study in accordance with the provisions of this protocol and all applicable protocol-related documents. I agree to conduct this study in compliance with United States (U.S.) Health and Human Service regulations (45 CFR 46); applicable U.S. Food and Drug Administration regulations; standards of the International Council for Harmonisation of Technical Requirements for Pharmaceuticals for Human Use (ICH) Guideline for Good Clinical Practice (E6(R2)); Institutional Review Board/Ethics Committee determinations; all applicable in-country, state, and local laws and regulations; and other applicable requirements (eg, U.S. National Institutes of Health, Division of AIDS) and institutional policies

Investigator of Record Name (print)	Investigator of Record Signature	Date

DAIDS Protocol Number: HVTN 140/HPTN 101

DAIDS Protocol Version: Version 1.0

Protocol Date: June 23, 2021

FOR REVIEW ONLY
